# Supplementary material for: Is type-D personality trait(s) or state? An examination of type-D temporal stability in older Israeli adults in the community
Source: PeerJ. 2016 Feb 9;4:e1690. doi: 10.7717/peerj.1690 (PMC4756746; doi:10.7717/peerj.1690)
Supplement: Supplemental Information 1 [file peerj-04-1690-s001.docx]

FREQUENCIES VARIABLES= fam_stat_p3 BIRTH_year_P3 age_p3 gender_p3 education_p3

/STATISTICS=STDDEV MINIMUM MAXIMUM MEAN MEDIAN MODE

/ORDER=ANALYSIS.

DATASET ACTIVATE DataSet2.

FREQUENCIES VARIABLES=ds_soc1 ds_neg1 typed1 ds_soc_P3 ds_neg_P3 typed_P3

/STATISTICS=STDDEV MINIMUM MAXIMUM MEAN MEDIAN MODE

/ORDER=ANALYSIS.

CORRELATIONS ds_soc1 ds_neg1 with ds_soc_P3 ds_neg_P3 .

DATASET ACTIVATE DataSet1.

CROSSTABS

/TABLES=gender_p3 BY typed_P3

/FORMAT=AVALUE TABLES

/STATISTICS=CHISQ

/CELLS=COUNT EXPECTED

/COUNT ROUND CELL.

DATASET ACTIVATE DataSet1.

CROSSTABS

/TABLES=typed1 BY typed_P3

/FORMAT=AVALUE TABLES

/STATISTICS=CHISQ

/CELLS=COUNT EXPECTED

/COUNT ROUND CELL.

LOGISTIC REGRESSION VARIABLES typed_P3

/method=enter gender_p3 age_p3

/method=enter ds_soc1 ds_neg1

/METHOD=ENTER NS1 HA1 RD1 PS1 SD1 CO1 ST1

/METHOD=ENTER tas_total1

/METHOD=ENTER cvd

/CRITERIA=PIN(.05) POUT(.10) ITERATE(20) CUT(.5).

DATASET ACTIVATE DataSet1.

FREQUENCIES VARIABLES=Gender_P3 Fam_stat_P3 age_p3 Education_P3

/STATISTICS=STDDEV MINIMUM MAXIMUM MEAN MEDIAN MODE

/ORDER=ANALYSIS.

compute d1_product=ds_soc1*ds_neg1.

compute d2_product=ds_soc_P3*ds_neg_P3.

DATASET ACTIVATE DataSet1.

FREQUENCIES VARIABLES=d1_product d2_product

/STATISTICS=STDDEV VARIANCE MINIMUM MAXIMUM MEAN MEDIAN MODE

/HISTOGRAM NORMAL

/ORDER=ANALYSIS.

compute d1_oness=0.

if (d1_product ge 132)d1_oness=1.

FREQUENCIES VARIABLES=d1_oness

/STATISTICS=STDDEV VARIANCE MINIMUM MAXIMUM MEAN MEDIAN MODE

/HISTOGRAM NORMAL

/ORDER=ANALYSIS.

CROSSTABS

/TABLES=d1_oness BY typed1

/FORMAT=AVALUE TABLES

/STATISTICS=CHISQ

/CELLS=COUNT ROW COLUMN TOTAL

/COUNT ROUND CELL.

compute d2_oness=0.

if (d2_product ge 132)d2_oness=1.

FREQUENCIES VARIABLES=d2_oness

/STATISTICS=STDDEV VARIANCE MINIMUM MAXIMUM MEAN MEDIAN MODE

/HISTOGRAM NORMAL

/ORDER=ANALYSIS.

CROSSTABS

/TABLES=d2_oness BY typed_P3

/FORMAT=AVALUE TABLES

/STATISTICS=CHISQ

/CELLS=COUNT ROW COLUMN TOTAL

/COUNT ROUND CELL.

CROSSTABS

/TABLES=d1_oness BY d2_oness

/FORMAT=AVALUE TABLES

/STATISTICS=CHISQ

/CELLS=COUNT ROW COLUMN TOTAL

/COUNT ROUND CELL.

**************

compute d1150_oness=0.

if (d1_product ge 150)d1150_oness=1.

FREQUENCIES VARIABLES=d1150_oness

/STATISTICS=STDDEV VARIANCE MINIMUM MAXIMUM MEAN MEDIAN MODE

/HISTOGRAM NORMAL

/ORDER=ANALYSIS.

CROSSTABS

/TABLES=d1150_oness BY typed1

/FORMAT=AVALUE TABLES

/STATISTICS=CHISQ

/CELLS=COUNT ROW COLUMN TOTAL

/COUNT ROUND CELL.

compute d2150_oness=0.

if (d2_product ge 150)d2150_oness=1.

FREQUENCIES VARIABLES=d2150_oness

/STATISTICS=STDDEV VARIANCE MINIMUM MAXIMUM MEAN MEDIAN MODE

/HISTOGRAM NORMAL

/ORDER=ANALYSIS.

CROSSTABS

/TABLES=d2150_oness BY typed_P3

/FORMAT=AVALUE TABLES

/STATISTICS=CHISQ

/CELLS=COUNT ROW COLUMN TOTAL

/COUNT ROUND CELL.

CROSSTABS

/TABLES=d1150_oness BY d2150_oness

/FORMAT=AVALUE TABLES

/STATISTICS=CHISQ

/CELLS=COUNT ROW COLUMN TOTAL

/COUNT ROUND CELL.
