# Supplementary material for: Is type-D personality trait(s) or state? An examination of type-D temporal stability in older Israeli adults in the community
Source: PeerJ. 2016 Feb 9;4:e1690. doi: 10.7717/peerj.1690 (PMC4756746; doi:10.7717/peerj.1690)
Supplement: Supplemental Information 2 [file peerj-04-1690-s002.pdf]

NO	Gender_P3	Fam_stat_P3	Birth_year_P3	YEAR_P3	Education_P3	Tas_1_P3_a
1,045	1	2	1,949	1	18	0.00
1,076	2	2	1,947	1	14	3.00
1,080	2	2	1,949	1	21	0.00
1,118	1	2	1,937	3	12	0.00
1,133	1	2	1,949	1	18	#NULL!
1,148	1	2	1,962	2	15	0.00
1,149	2	2	1,963	1	15	0.00
1,151	1	2	1,934	1	14	0.00
1,153	2	2	1,953	1	16	0.00
1,163	2	5	1,938	3	12	0.00
1,164	2	2	1,944	1	12	0.00
1,171	2	2	1,943	1	12	1.00
1,178	2	2	1,960	1	20	1.00
1,184	1	2	1,944	3	16	0.00
1,198	1	3	1,930	1	15	1.00
1,200	2	2	1,960	1	18	#NULL!
1,201	2	3	1,942	2	20	1.00
1,210	2	2	1,940	1	12	0.00
1,211	1	2	1,925	1	13	1.00
1,219	2	3	1,940	1	12	1.00
1,222	1	2	1,961	1	16	0.00
1,223	2	2	1,944	1	18	1.00
1,225	1	2	1,953	1	15	0.00
1,226	1	2	1,966	1	15	0.00
1,239	2	2	1,948	1	20	1.00
1,241	2	2	1,947	1	16	1.00
1,242	2	2	1,950	1	16	0.00
1,243	1	2	1,961	1	16	1.00
1,244	1	2	1,944	2	16	1.00
1,249	2	2	1,951	1	17	0.00
1,251	2	2	1,944	1	12	1.00
1,253	1	5	1,938	1	18	0.00
1,255	2	1	1,957	1	15	1.00
1,257	2	3	1,949	1	15	3.00
1,259	2	5	1,928	3	17	#NULL!
1,690	2	2	1,951	3	22	#NULL!
1,990	2	2	1,964	3	15	#NULL!
1,136	1	5	1,922	3	6	#NULL!
1,261	2	2	1,944	2	17	0.00
1,268	2	2	1,950	1	15	1.00
1,271	2	5	1,917	1	9	1.00
1,278	2	5	1,932	1	16	1.00
1,279	2	2	1,948	1	20	1.00
1,285	2	2	1,943	1	12	#NULL!
1,292	2	2	1,963	3	18	1.00
1,293	1	2	1,966	1	18	0.00
1,300	2	2	1,948	1	17	0.00
1,303	1	1	1,953	1	15	#NULL!
1,306	1	2	1,954	1	15	0.00
1,309	2	2	1,952	1	13	0.00
1,310	2	2	1,944	1	18	1.00
1,311	2	2	1,946	1	15	#NULL!
1,313	2	2	1,946	2	12	1.00
1,315	2	5	1,940	1	14	1.00
1,323	2	1	1,950	1	12	#NULL!

1,325	2	2	1,943	1	12	0.00
1,329	2	2	1,949	1	17	0.00
1,330	1	2	1,963	1	18	0.00
1,331	2	2	1,944	1	12	0.00
1,336	2	5	1,944	1	18	1.00
1,337	2	3	1,945	1	15	#NULL!
1,346	1	2	1,943	1	15	0.00
1,347	1	4	1,947	1	15	0.00
1,352	2	3	1,966	2	17	3.00
1,353	1	5	1,934	1	15	1.00
1,354	1	2	1,936	1	11	0.00
1,355	2	2	1,937	1	12	2.00
1,356	2	2	1,935	1	17	1.00
1,357	1	2	1,935	1	13	1.00
1,358	2	2	1,950	1	14	1.00
1,359	2	2	1,966	1	16	2.00
1,362	1	3	1,952	1	14	0.00
1,365	2	2	1,955	1	15	0.00
1,373	1	2	1,956	1	16	1.00
1,375	2	2	1,959	1	15	1.00
1,378	1	2	1,957	1	10	0.00
1,379	1	2	1,947	2	15	0.00
1,387	2	2	1,965	1	15	#NULL!
1,388	1	2	1,956	2	16	0.00
1,389	2	2	1,952	1	20	0.00
1,390	1	2	1,951	1	14	1.00
1,393	1	2	1,951	1	17	1.00
1,394	2	3	1,960	1	13	0.00
1,399	1	2	1,934	1	15	1.00
1,401	2	2	1,961	1	16	0.00
1,402	1	2	1,957	1	12	0.00
1,407	1	2	1,954	1	15	1.00
1,409	2	3	1,959	2	16	0.00
1,415	2	2	1,953	1	16	2.00
1,418	1	2	1,951	2	23	0.00
1,421	2	2	1,963	3	15	1.00
1,432	1	2	1,955	1	16	1.00
1,437	2	2	1,964	3	21	4.00
1,438	1	2	1,963	1	21	#NULL!
1,441	1	2	1,942	1	15	1.00
1,445	1	3	1,945	1	14	2.00
1,447	1	2	1,953	2	15	2.00
1,448	2	2	1,953	1	21	0.00
1,450	2	1	1,942	1	18	1.00
1,452	2	4	1,958	1	14	2.00
1,455	1	2	1,937	1	16	1.00
1,457	2	2	1,952	1	18	1.00
1,462	2	4	1,960	1	15	1.00
1,463	2	2	1,965	3	14	0.00
1,464	1	2	1,962	2	14	1.00
1,468	2	2	1,939	1	15	#NULL!
1,472	2	3	1,962	1	12	0.00
1,476	1	2	1,950	1	13	1.00
1,479	2	2	1,963	2	15	1.00
1,480	2	#NULL!	1,951	2	13	#NULL!
1,485	2	3	1,948	1	18	1.00

1,487	2	3	1,948	1	14	1.00
1,488	1	5	1,940	1	16	1.00
1,489	1	2	1,964	1	18	0.00
1,496	1	2	1,950	1	23	#NULL!
1,497	2	2	1,949	1	15	1.00
1,498	2	2	1,925	1	15	1.00
1,504	1	2	1,948	1	14	1.00
1,505	2	2	1,965	2	14	0.00
1,506	1	2	1,961	2	15	#NULL!
1,511	2	2	1,965	1	12	1.00
1,512	1	2	1,954	1	19	0.00
1,513	2	2	1,951	1	18	0.00
1,514	1	2	1,943	1	17	1.00
1,523	1	2	1,958	2	17	0.00
1,528	1	2	1,955	1	16	0.00
1,532	1	3	1,936	1	15	#NULL!
1,534	2	3	1,950	2	16	1.00
1,536	2	2	1,954	1	23	#NULL!
1,545	1	2	1,939	1	18	0.00
1,546	2	2	1,946	2	12	1.00
1,549	2	3	1,953	1	20	#NULL!
1,550	1	2	1,963	1	12	0.00
1,556	2	2	1,953	1	16	#NULL!
1,564	1	2	1,966	1	16	0.00
1,580	2	5	1,948	1	12	0.00
1,582	1	2	1,966	2	15	3.00
1,583	1	2	1,953	2	15	0.00
1,584	2	5	1,955	1	17	1.00
1,587	2	3	1,945	1	18	#NULL!
1,598	2	3	1,957	1	14	#NULL!
1,599	2	3	1,956	1	14	1.00
1,606	2	3	1,967	1	16	1.00
1,607	2	5	1,942	1	16	3.00
1,614	2	2	1,946	1	12	1.00
1,615	1	2	1,939	1	12	1.00
1,616	2	2	1,939	1	14	0.00
1,620	2	2	1,934	1	15	#NULL!
1,623	2	2	1,957	1	13	0.00
1,626	1	2	1,947	1	19	2.00
1,627	1	2	1,954	1	20	#NULL!
1,632	1	2	1,941	1	12	2.00
1,634	2	2	1,960	1	16	2.00
1,636	2	2	1,965	1	14	1.00
1,637	1	2	1,957	1	18	1.00
1,638	2	3	1,945	1	17	1.00
1,641	2	2	1,945	1	17	0.00
1,645	2	2	1,965	3	12	3.00
1,650	1	2	1,936	2	12	1.00
1,655	2	2	1,957	1	16	1.00
1,671	2	2	1,954	2	15	1.00
1,674	1	3	1,938	1	15	0.00
1,677	1	2	1,943	1	20	#NULL!
1,685	2	2	1,959	1	19	0.00
1,687	1	2	1,955	1	16	1.00
1,689	1	2	1,958	1	17	1.00
1,696	2	3	1,959	1	18	1.00

1,698	2	3	1,964	3	19	0.00
1,702	2	2	1,962	1	13	0.00
1,705	1	2	1,957	1	12	0.00
1,706	2	3	1,943	1	19	1.00
1,710	1	3	1,957	1	15	#NULL!
1,712	2	2	1,942	1	12	0.00
1,714	1	3	1,956	1	16	1.00
1,715	2	2	1,964	2	15	0.00
1,719	2	1	1,943	1	20	0.00
1,721	2	3	1,946	1	15	3.00
1,723	2	5	1,935	1	10	1.00
1,730	1	3	1,964	1	14	0.00
1,738	2	2	1,953	1	15	1.00
1,741	2	2	1,961	1	16	3.00
1,744	2	3	1,951	1	15	2.00
1,745	1	3	1,945	1	16	#NULL!
1,747	2	2	1,943	2	12	1.00
1,748	1	2	1,943	1	19	0.00
1,749	1	3	1,944	1	14	3.00
1,754	2	5	1,935	1	18	#NULL!
1,755	2	1	1,937	1	15	0.00
1,756	2	4	1,945	1	20	#NULL!
1,759	1	2	1,963	1	22	0.00
1,762	1	3	1,960	2	15	1.00
1,772	1	2	1,943	1	16	0.00
1,775	2	2	1,944	1	25	1.00
1,777	2	2	1,958	1	22	2.00
1,778	2	2	1,952	1	14	0.00
1,779	2	2	1,943	1	13	1.00
1,790	1	2	1,959	1	15	1.00
1,791	1	2	1,966	2	16	1.00
1,792	1	2	1,932	1	19	#NULL!
1,794	1	2	1,933	1	20	1.00
1,796	2	2	1,951	1	15	#NULL!
1,803	1	3	1,951	1	13	1.00
1,805	2	5	1,937	1	14	1.00
1,813	1	2	1,946	1	15	#NULL!
1,816	1	2	1,967	1	15	3.00
1,824	1	2	1,955	2	17	2.00
1,826	2	3	1,942	1	18	1.00
1,831	2	2	1,934	2	15	#NULL!
1,833	2	2	1,953	1	16	1.00
1,835	2	5	1,944	1	12	1.00
1,837	2	2	1,935	3	22	0.00
1,839	2	2	1,953	1	15	1.00
1,841	2	2	1,957	1	14	#NULL!
1,842	2	3	1,952	2	21	0.00
1,848	1	5	1,937	1	12	1.00
1,849	1	2	1,967	1	15	1.00
1,857	2	3	1,958	1	19	#NULL!
1,858	2	3	1,953	2	15	1.00
1,860	1	4	1,949	1	18	1.00
1,863	1	2	1,939	1	20	1.00
1,865	1	3	1,961	1	13	0.00
1,870	2	3	1,955	1	15	#NULL!
1,873	2	2	1,962	2	20	1.00

1,877	2	2	1,942	1	12	1.00
1,886	1	2	1,955	1	16	2.00
1,890	1	2	1,962	1	14	1.00
1,891	1	2	1,941	1	13	1.00
1,892	1	3	1,961	1	14	2.00
1,895	2	3	1,944	1	19	1.00
1,897	2	2	1,946	1	25	0.00
1,898	1	2	1,935	1	24	#NULL!
1,902	2	2	1,956	1	15	1.00
1,907	2	3	1,957	1	13	3.00
1,910	2	2	1,956	1	15	2.00
1,911	2	2	1,936	1	14	#NULL!
1,912	1	3	1,953	1	15	#NULL!
1,915	1	3	1,958	1	14	1.00
1,924	1	2	1,965	1	17	1.00
1,925	1	2	1,948	1	16	1.00
1,926	2	2	1,956	1	12	1.00
1,934	1	2	1,958	1	14	1.00
1,938	1	2	1,927	1	12	999.00
1,940	1	2	1,960	2	16	0.00
1,949	2	3	1,953	1	18	0.00
1,952	2	3	1,957	1	16	1.00
1,953	2	2	1,949	3	17	0.00
1,957	2	3	1,956	1	11	3.00
1,961	2	2	1,953	2	15	2.00
1,966	2	2	1,946	1	16	2.00
1,968	1	2	1,959	1	21	2.00
1,969	2	2	1,962	1	18	0.00
1,970	2	2	1,949	2	20	0.00
1,971	2	2	1,940	3	12	1.00
1,973	2	2	1,964	1	12	2.00
1,974	1	3	1,942	2	15	2.00
1,978	2	2	1,946	3	17	0.00
1,980	1	3	1,939	1	17	1.00
1,986	1	2	1,950	1	17	0.00
1,987	2	2	1,949	1	16	#NULL!
1,989	1	2	1,936	1	15	1.00
1,992	2	2	1,946	1	18	0.00
1,993	2	3	1,942	1	16	1.00
1,994	2	2	1,947	1	14	1.00
1,996	2	2	1,938	1	16	0.00
1,999	1	2	1,954	2	14	2.00
2,009	2	3	1,935	1	12	1.00
2,019	2	5	1,944	1	18	1.00
2,020	1	2	1,956	1	17	1.00
2,022	2	5	1,926	1	15	#NULL!
3,001	2	5	1,932	1	14	2.00
3,002	1	2	1,946	1	17	0.00
3,004	2	2	1,958	1	17	1.00
3,006	2	2	1,959	1	25	1.00
3,010	2	5	1,951	2	18	1.00
3,012	2	5	1,940	1	16	1.00
3,019	1	2	1,936	1	16	1.00
3,021	2	3	1,951	1	12	#NULL!
3,022	2	2	1,952	2	15	3.00
3,025	2	2	1,947	1	16	1.00

3,027	1	2	1,950	2	12	0.00
3,030	1	2	1,953	1	24	1.00
3,037	1	2	1,940	1	16	0.00
3,038	2	3	1,945	1	12	1.00
3,049	1	3	1,962	2	16	#NULL!
3,051	1	2	1,959	1	12	#NULL!
3,056	1	2	1,953	1	15	0.00
3,063	1	3	1,946	1	19	3.00
4,044	1	3	1,964	1	15	0.00
7,000	1	5	1,942	1	12	0.00
7,001	1	2	1,940	1	19	0.00
7,002	1	2	1,940	1	16	0.00
7,003	1	2	1,956	1	22	0.00
7,004	1	2	1,943	1	18	1.00
10,011	2	2	1,951	1	21	0.00
10,027	2	2	1,960	1	13	0.00
11,000	2	5	1,937	3	12	4.00
11,001	1	2	1,957	3	12	1.00

Tas_2_P3_a	Tas_3_P3_a	Tas_4_P3_a	Tas_5_P3_a	Tas_6_P3_a	Tas_7_P3_a	Tas_8_P3_a	Tas_9_P3_a
1.00	0.00	2.000	1.00	0.00	0.00	1.00	0.00
4.00	3.00	3.000	4.00	4.00	3.00	1.00	3.00
1.00	0.00	3.000	1.00	1.00	2.00	1.00	2.00
0.00	0.00	4.000	3.00	2.00	1.00	0.00	0.00
#NULL!	#NULL!	#NULL!	#NULL!	#NULL!	#NULL!	#NULL!	#NULL!
0.00	0.00	4.000	3.00	0.00	0.00	3.00	0.00
2.00	0.00	1.000	2.00	0.00	0.00	1.00	0.00
0.00	0.00	3.000	3.00	1.00	1.00	1.00	0.00
1.00	1.00	3.000	3.00	1.00	1.00	3.00	1.00
0.00	4.00	4.000	4.00	3.00	0.00	0.00	0.00
0.00	0.00	3.000	3.00	0.00	1.00	1.00	1.00
1.00	1.00	3.000	2.00	1.00	1.00	2.00	1.00
1.00	0.00	3.000	3.00	1.00	0.00	2.00	1.00
0.00	0.00	3.000	3.00	2.00	1.00	1.00	1.00
0.00	2.00	3.000	3.00	0.00	1.00	1.00	2.00
#NULL!	#NULL!	#NULL!	#NULL!	#NULL!	#NULL!	#NULL!	#NULL!
1.00	1.00	3.000	3.00	2.00	1.00	2.00	1.00
3.00	0.00	0.000	0.00	0.00	0.00	2.00	0.00
1.00	1.00	4.000	4.00	4.00	4.00	999.00	1.00
3.00	3.00	0.000	0.00	1.00	0.00	1.00	3.00
1.00	1.00	3.000	4.00	0.00	0.00	2.00	1.00
0.00	0.00	4.000	4.00	1.00	0.00	2.00	0.00
0.00	0.00	1.000	3.00	0.00	0.00	0.00	0.00
1.00	1.00	2.000	2.00	2.00	2.00	2.00	2.00
1.00	4.00	3.000	1.00	1.00	2.00	3.00	1.00
1.00	0.00	4.000	3.00	3.00	1.00	1.00	0.00
0.00	0.00	4.000	4.00	2.00	0.00	4.00	3.00
1.00	1.00	4.000	3.00	0.00	1.00	4.00	1.00
1.00	0.00	2.000	2.00	1.00	0.00	1.00	1.00
0.00	0.00	3.000	4.00	0.00	0.00	0.00	0.00
1.00	1.00	2.000	0.00	1.00	1.00	1.00	2.00
0.00	0.00	3.000	2.00	0.00	0.00	2.00	1.00
3.00	0.00	3.000	3.00	1.00	0.00	1.00	1.00
3.00	0.00	3.000	2.00	1.00	0.00	4.00	3.00
#NULL!	#NULL!	#NULL!	#NULL!	#NULL!	#NULL!	#NULL!	#NULL!
#NULL!	#NULL!	#NULL!	#NULL!	#NULL!	#NULL!	#NULL!	#NULL!
#NULL!	#NULL!	#NULL!	#NULL!	#NULL!	#NULL!	#NULL!	#NULL!
#NULL!	#NULL!	#NULL!	#NULL!	#NULL!	#NULL!	#NULL!	#NULL!
0.00	0.00	0.000	1.00	0.00	0.00	0.00	0.00
2.00	0.00	2.000	3.00	1.00	0.00	1.00	1.00
2.00	3.00	2.000	2.00	3.00	2.00	4.00	1.00
0.00	1.00	3.000	4.00	0.00	2.00	1.00	0.00
1.00	3.00	4.000	3.00	1.00	3.00	1.00	1.00
#NULL!	#NULL!	#NULL!	#NULL!	#NULL!	#NULL!	#NULL!	#NULL!
1.00	4.00	4.000	4.00	1.00	4.00	4.00	4.00
0.00	0.00	4.000	0.00	2.00	0.00	0.00	0.00
0.00	0.00	3.000	3.00	0.00	0.00	0.00	0.00
#NULL!	#NULL!	#NULL!	#NULL!	#NULL!	#NULL!	#NULL!	#NULL!
0.00	1.00	3.000	4.00	0.00	0.00	1.00	0.00
0.00	0.00	4.000	3.00	0.00	0.00	2.00	0.00
0.00	3.00	2.000	3.00	0.00	0.00	1.00	0.00
#NULL!	#NULL!	#NULL!	#NULL!	#NULL!	#NULL!	#NULL!	#NULL!
1.00	0.00	4.000	3.00	3.00	2.00	1.00	0.00
0.00	2.00	4.000	3.00	1.00	2.00	0.00	1.00
#NULL!	#NULL!	#NULL!	#NULL!	#NULL!	#NULL!	#NULL!	#NULL!

3.00	0.00	2.000	3.00	2.00	0.00	2.00	1.00
1.00	0.00	0.000	0.00	1.00	1.00	0.00	0.00
0.00	0.00	0.000	2.00	0.00	0.00	0.00	0.00
0.00	0.00	0.000	0.00	0.00	0.00	0.00	0.00
1.00	0.00	3.000	3.00	1.00	1.00	1.00	1.00
#NULL!	#NULL!	#NULL!	#NULL!	#NULL!	#NULL!	#NULL!	#NULL!
0.00	0.00	4.000	4.00	0.00	0.00	3.00	0.00
0.00	0.00	4.000	3.00	1.00	0.00	1.00	0.00
1.00	1.00	3.000	3.00	2.00	3.00	2.00	1.00
1.00	2.00	1.000	1.00	1.00	2.00	2.00	2.00
0.00	0.00	4.000	2.00	1.00	0.00	3.00	0.00
2.00	1.00	3.000	0.00	0.00	0.00	0.00	0.00
0.00	0.00	1.000	4.00	0.00	0.00	2.00	0.00
2.00	1.00	2.000	2.00	2.00	1.00	2.00	1.00
2.00	0.00	2.000	2.00	2.00	1.00	0.00	2.00
1.00	0.00	3.000	3.00	1.00	1.00	4.00	1.00
0.00	0.00	3.000	2.00	1.00	0.00	2.00	1.00
0.00	0.00	3.000	3.00	3.00	1.00	1.00	1.00
1.00	1.00	3.000	2.00	1.00	1.00	2.00	1.00
2.00	0.00	2.000	1.00	1.00	0.00	3.00	1.00
0.00	0.00	4.000	0.00	0.00	0.00	4.00	0.00
1.00	1.00	1.000	3.00	0.00	1.00	3.00	2.00
#NULL!	#NULL!	#NULL!	#NULL!	#NULL!	#NULL!	#NULL!	#NULL!
0.00	3.00	0.000	2.00	0.00	0.00	0.00	0.00
4.00	0.00	0.000	0.00	0.00	0.00	0.00	0.00
1.00	1.00	4.000	2.00	3.00	1.00	3.00	1.00
1.00	1.00	3.000	3.00	1.00	1.00	1.00	1.00
0.00	3.00	0.000	2.00	0.00	0.00	0.00	0.00
1.00	1.00	1.000	1.00	1.00	1.00	2.00	1.00
1.00	3.00	3.000	2.00	1.00	1.00	2.00	1.00
0.00	0.00	4.000	3.00	0.00	0.00	3.00	0.00
1.00	1.00	3.000	4.00	1.00	0.00	1.00	1.00
0.00	0.00	0.000	3.00	0.00	0.00	0.00	0.00
1.00	1.00	3.000	1.00	1.00	1.00	3.00	3.00
0.00	0.00	4.000	2.00	0.00	0.00	0.00	0.00
1.00	1.00	4.000	3.00	1.00	1.00	1.00	1.00
1.00	1.00	3.000	3.00	1.00	1.00	1.00	1.00
3.00	1.00	2.000	3.00	3.00	1.00	2.00	2.00
#NULL!	#NULL!	#NULL!	#NULL!	#NULL!	#NULL!	#NULL!	#NULL!
1.00	1.00	2.000	3.00	1.00	1.00	3.00	1.00
1.00	2.00	3.000	2.00	1.00	2.00	2.00	1.00
1.00	1.00	2.000	1.00	1.00	1.00	2.00	1.00
3.00	2.00	3.000	3.00	0.00	0.00	3.00	0.00
1.00	1.00	1.000	4.00	1.00	1.00	1.00	1.00
2.00	2.00	2.000	3.00	2.00	2.00	1.00	1.00
1.00	1.00	4.000	3.00	0.00	3.00	2.00	0.00
1.00	2.00	3.000	3.00	3.00	1.00	1.00	1.00
1.00	3.00	2.000	1.00	1.00	1.00	2.00	1.00
0.00	0.00	4.000	3.00	1.00	1.00	3.00	0.00
2.00	1.00	3.000	1.00	1.00	1.00	2.00	1.00
#NULL!	#NULL!	#NULL!	#NULL!	#NULL!	#NULL!	#NULL!	#NULL!
2.00	0.00	3.000	3.00	0.00	0.00	1.00	0.00
1.00	1.00	3.000	2.00	1.00	2.00	3.00	2.00
2.00	2.00	3.000	1.00	1.00	0.00	2.00	1.00
#NULL!	#NULL!	#NULL!	#NULL!	#NULL!	#NULL!	#NULL!	#NULL!
1.00	1.00	3.000	2.00	1.00	0.00	1.00	1.00

1.00	1.00	1.000	2.00	0.00	1.00	3.00	1.00
1.00	2.00	3.000	3.00	2.00	1.00	2.00	1.00
0.00	0.00	4.000	3.00	1.00	0.00	3.00	0.00
#NULL!	#NULL!	#NULL!	#NULL!	#NULL!	#NULL!	#NULL!	#NULL!
1.00	1.00	3.000	2.00	1.00	1.00	1.00	1.00
1.00	0.00	4.000	4.00	0.00	0.00	0.00	0.00
3.00	2.00	1.000	3.00	1.00	1.00	1.00	1.00
0.00	0.00	4.000	4.00	1.00	0.00	1.00	0.00
#NULL!	#NULL!	#NULL!	#NULL!	#NULL!	#NULL!	#NULL!	#NULL!
1.00	3.00	1.000	4.00	1.00	1.00	4.00	3.00
0.00	0.00	3.000	3.00	0.00	0.00	0.00	0.00
1.00	0.00	3.000	3.00	0.00	0.00	0.00	0.00
1.00	0.00	3.000	1.00	1.00	0.00	1.00	1.00
1.00	1.00	3.000	3.00	2.00	1.00	1.00	1.00
0.00	0.00	4.000	3.00	0.00	0.00	0.00	#NULL!
#NULL!	#NULL!	#NULL!	#NULL!	#NULL!	#NULL!	#NULL!	#NULL!
1.00	0.00	2.000	3.00	0.00	0.00	1.00	1.00
#NULL!	#NULL!	#NULL!	#NULL!	#NULL!	#NULL!	#NULL!	#NULL!
0.00	0.00	4.000	4.00	0.00	0.00	0.00	0.00
1.00	1.00	3.000	2.00	2.00	3.00	3.00	1.00
#NULL!	#NULL!	#NULL!	#NULL!	#NULL!	#NULL!	#NULL!	#NULL!
0.00	1.00	2.000	2.00	0.00	0.00	0.00	0.00
#NULL!	#NULL!	#NULL!	#NULL!	#NULL!	#NULL!	#NULL!	#NULL!
0.00	2.00	3.000	1.00	3.00	2.00	0.00	1.00
3.00	0.00	2.000	3.00	3.00	1.00	1.00	3.00
2.00	0.00	2.000	3.00	0.00	0.00	0.00	0.00
0.00	0.00	3.000	1.00	3.00	0.00	3.00	3.00
1.00	0.00	3.000	3.00	1.00	1.00	3.00	1.00
#NULL!	#NULL!	#NULL!	#NULL!	#NULL!	#NULL!	#NULL!	#NULL!
#NULL!	#NULL!	#NULL!	#NULL!	#NULL!	#NULL!	#NULL!	#NULL!
1.00	0.00	3.000	2.00	1.00	0.00	999.00	1.00
1.00	1.00	3.000	3.00	1.00	0.00	1.00	0.00
1.00	1.00	2.000	2.00	999.00	2.00	2.00	2.00
2.00	0.00	1.000	2.00	2.00	1.00	2.00	2.00
2.00	2.00	2.000	3.00	1.00	1.00	3.00	2.00
0.00	0.00	4.000	3.00	0.00	0.00	2.00	0.00
#NULL!	#NULL!	#NULL!	#NULL!	#NULL!	#NULL!	#NULL!	#NULL!
2.00	0.00	2.000	3.00	1.00	1.00	3.00	1.00
1.00	0.00	1.000	4.00	1.00	1.00	#NULL!	1.00
#NULL!	#NULL!	#NULL!	#NULL!	#NULL!	#NULL!	#NULL!	#NULL!
1.00	2.00	2.000	4.00	4.00	2.00	4.00	3.00
2.00	0.00	4.000	3.00	3.00	1.00	2.00	3.00
1.00	0.00	3.000	3.00	2.00	1.00	1.00	3.00
1.00	1.00	3.000	3.00	2.00	1.00	2.00	1.00
1.00	3.00	3.000	3.00	0.00	1.00	0.00	1.00
0.00	0.00	4.000	4.00	0.00	0.00	0.00	0.00
3.00	1.00	3.000	4.00	2.00	1.00	1.00	3.00
1.00	4.00	3.000	3.00	0.00	0.00	0.00	0.00
2.00	1.00	3.000	4.00	0.00	1.00	2.00	1.00
2.00	3.00	4.000	4.00	1.00	1.00	1.00	1.00
0.00	0.00	3.000	1.00	0.00	0.00	1.00	0.00
#NULL!	#NULL!	#NULL!	#NULL!	#NULL!	#NULL!	#NULL!	#NULL!
0.00	2.00	0.000	2.00	0.00	1.00	1.00	0.00
1.00	0.00	3.000	3.00	1.00	0.00	1.00	1.00
1.00	1.00	3.000	3.00	2.00	2.00	0.00	2.00
1.00	3.00	4.000	3.00	1.00	3.00	1.00	1.00

2.00	0.00	3.000	4.00	3.00	2.00	1.00	3.00
0.00	0.00	4.000	3.00	0.00	0.00	3.00	0.00
0.00	3.00	4.000	4.00	0.00	2.00	0.00	0.00
1.00	0.00	1.000	3.00	1.00	1.00	1.00	1.00
#NULL!	#NULL!	#NULL!	#NULL!	#NULL!	#NULL!	#NULL!	#NULL!
0.00	0.00	4.000	4.00	0.00	1.00	1.00	0.00
3.00	1.00	4.000	1.00	4.00	0.00	1.00	1.00
0.00	0.00	4.000	4.00	0.00	0.00	0.00	0.00
0.00	4.00	3.000	1.00	0.00	4.00	1.00	0.00
3.00	2.00	3.000	3.00	3.00	2.00	3.00	2.00
3.00	3.00	3.000	3.00	3.00	3.00	2.00	3.00
0.00	0.00	3.000	2.00	0.00	0.00	3.00	0.00
3.00	1.00	1.000	4.00	1.00	2.00	4.00	1.00
1.00	1.00	3.000	3.00	2.00	1.00	2.00	2.00
2.00	3.00	3.000	3.00	3.00	3.00	3.00	1.00
#NULL!	#NULL!	#NULL!	#NULL!	#NULL!	#NULL!	#NULL!	#NULL!
1.00	2.00	3.000	2.00	2.00	1.00	2.00	1.00
0.00	0.00	4.000	3.00	1.00	0.00	1.00	1.00
3.00	2.00	1.000	4.00	2.00	3.00	3.00	2.00
#NULL!	#NULL!	#NULL!	#NULL!	#NULL!	#NULL!	#NULL!	#NULL!
0.00	0.00	4.000	3.00	0.00	0.00	0.00	0.00
#NULL!	#NULL!	#NULL!	#NULL!	#NULL!	#NULL!	#NULL!	#NULL!
0.00	0.00	3.000	3.00	0.00	1.00	1.00	1.00
1.00	3.00	3.000	3.00	0.00	0.00	2.00	0.00
0.00	0.00	4.000	3.00	0.00	0.00	2.00	0.00
1.00	1.00	4.000	2.00	0.00	0.00	2.00	1.00
3.00	3.00	3.000	3.00	3.00	2.00	1.00	3.00
0.00	0.00	4.000	1.00	0.00	0.00	3.00	0.00
2.00	2.00	1.000	3.00	1.00	2.00	1.00	2.00
0.00	3.00	3.000	2.00	1.00	2.00	3.00	1.00
0.00	1.00	0.000	3.00	1.00	2.00	3.00	1.00
#NULL!	#NULL!	#NULL!	#NULL!	#NULL!	#NULL!	#NULL!	#NULL!
1.00	4.00	1.000	4.00	3.00	3.00	1.00	1.00
#NULL!	#NULL!	#NULL!	#NULL!	#NULL!	#NULL!	#NULL!	#NULL!
2.00	0.00	0.000	2.00	2.00	1.00	3.00	2.00
1.00	2.00	3.000	3.00	1.00	1.00	1.00	1.00
#NULL!	#NULL!	#NULL!	#NULL!	#NULL!	#NULL!	#NULL!	#NULL!
3.00	0.00	2.000	1.00	3.00	0.00	3.00	3.00
3.00	1.00	3.000	3.00	2.00	2.00	1.00	3.00
1.00	2.00	3.000	3.00	0.00	0.00	1.00	1.00
#NULL!	#NULL!	#NULL!	#NULL!	#NULL!	#NULL!	#NULL!	#NULL!
1.00	1.00	3.000	2.00	2.00	1.00	2.00	1.00
2.00	0.00	2.000	0.00	0.00	0.00	2.00	0.00
0.00	0.00	3.000	3.00	0.00	0.00	0.00	0.00
1.00	3.00	3.000	3.00	0.00	1.00	0.00	1.00
#NULL!	#NULL!	#NULL!	#NULL!	#NULL!	#NULL!	#NULL!	#NULL!
0.00	0.00	4.000	1.00	0.00	0.00	3.00	0.00
1.00	0.00	3.000	3.00	1.00	0.00	0.00	0.00
1.00	1.00	1.000	1.00	1.00	1.00	1.00	2.00
#NULL!	#NULL!	#NULL!	#NULL!	#NULL!	#NULL!	#NULL!	#NULL!
1.00	1.00	4.000	2.00	0.00	0.00	1.00	0.00
1.00	1.00	1.000	2.00	1.00	1.00	1.00	1.00
1.00	0.00	3.000	2.00	3.00	1.00	1.00	2.00
0.00	3.00	4.000	1.00	0.00	4.00	1.00	2.00
#NULL!	#NULL!	#NULL!	#NULL!	#NULL!	#NULL!	#NULL!	#NULL!
1.00	1.00	4.000	2.00	1.00	1.00	1.00	1.00

1.00	1.00	1.000	1.00	1.00	2.00	1.00	1.00
1.00	2.00	3.000	3.00	1.00	2.00	2.00	2.00
2.00	1.00	2.000	3.00	1.00	0.00	1.00	0.00
2.00	3.00	2.000	3.00	3.00	3.00	1.00	3.00
2.00	1.00	3.000	2.00	2.00	1.00	2.00	1.00
1.00	0.00	2.000	2.00	1.00	0.00	0.00	1.00
0.00	2.00	4.000	3.00	0.00	0.00	0.00	1.00
#NULL!	#NULL!	#NULL!	#NULL!	#NULL!	#NULL!	#NULL!	#NULL!
1.00	2.00	3.000	2.00	1.00	2.00	1.00	1.00
2.00	1.00	2.000	2.00	2.00	2.00	3.00	2.00
1.00	0.00	3.000	1.00	0.00	0.00	1.00	1.00
#NULL!	#NULL!	#NULL!	#NULL!	#NULL!	#NULL!	#NULL!	#NULL!
#NULL!	#NULL!	#NULL!	#NULL!	#NULL!	#NULL!	#NULL!	#NULL!
1.00	2.00	3.000	2.00	1.00	3.00	1.00	1.00
1.00	2.00	2.000	2.00	1.00	1.00	2.00	2.00
1.00	1.00	3.000	4.00	3.00	1.00	0.00	1.00
2.00	2.00	1.000	2.00	0.00	1.00	1.00	2.00
2.00	0.00	2.000	1.00	1.00	0.00	2.00	1.00
999.00	3.00	4.000	4.00	1.00	1.00	4.00	1.00
0.00	0.00	4.000	4.00	2.00	0.00	0.00	999.00
0.00	2.00	4.000	2.00	0.00	2.00	2.00	0.00
1.00	3.00	3.000	3.00	1.00	1.00	1.00	1.00
0.00	0.00	4.000	3.00	2.00	0.00	4.00	0.00
4.00	3.00	3.000	3.00	4.00	3.00	999.00	3.00
2.00	3.00	2.000	2.00	1.00	1.00	1.00	2.00
2.00	1.00	0.000	0.00	1.00	1.00	3.00	2.00
3.00	1.00	1.000	2.00	1.00	0.00	1.00	2.00
0.00	0.00	2.000	1.00	2.00	0.00	0.00	0.00
0.00	0.00	4.000	4.00	0.00	0.00	0.00	0.00
1.00	1.00	4.000	4.00	1.00	1.00	1.00	1.00
4.00	1.00	1.000	3.00	2.00	2.00	2.00	2.00
0.00	3.00	3.000	4.00	3.00	0.00	0.00	0.00
1.00	1.00	2.000	2.00	1.00	1.00	1.00	1.00
3.00	0.00	1.000	2.00	1.00	0.00	2.00	0.00
0.00	0.00	0.000	1.00	0.00	0.00	0.00	0.00
#NULL!	#NULL!	#NULL!	#NULL!	#NULL!	#NULL!	#NULL!	#NULL!
1.00	0.00	1.000	1.00	1.00	0.00	2.00	1.00
0.00	0.00	4.000	0.00	0.00	0.00	0.00	3.00
1.00	0.00	3.000	3.00	1.00	0.00	1.00	2.00
1.00	1.00	4.000	3.00	1.00	0.00	0.00	1.00
0.00	0.00	0.000	1.00	2.00	0.00	1.00	1.00
3.00	0.00	1.000	1.00	0.00	0.00	3.00	3.00
1.00	1.00	2.000	2.00	2.00	1.00	3.00	2.00
1.00	1.00	3.000	3.00	0.00	1.00	0.00	1.00
2.00	1.00	3.000	3.00	0.00	1.00	1.00	1.00
#NULL!	#NULL!	#NULL!	#NULL!	#NULL!	#NULL!	#NULL!	#NULL!
2.00	1.00	1.000	1.00	1.00	2.00	3.00	1.00
0.00	2.00	2.000	3.00	3.00	2.00	1.00	1.00
1.00	1.00	3.000	4.00	1.00	0.00	0.00	1.00
2.00	1.00	3.000	2.00	1.00	0.00	1.00	1.00
1.00	1.00	3.000	4.00	2.00	1.00	1.00	1.00
1.00	0.00	3.000	3.00	4.00	0.00	0.00	3.00
1.00	1.00	2.000	1.00	1.00	2.00	1.00	2.00
#NULL!	#NULL!	#NULL!	#NULL!	#NULL!	#NULL!	#NULL!	#NULL!
4.00	4.00	1.000	2.00	3.00	3.00	2.00	2.00
2.00	0.00	2.000	1.00	1.00	0.00	2.00	1.00

0.00	1.00	3.000	2.00	1.00	0.00	0.00	1.00
1.00	1.00	3.000	2.00	1.00	1.00	1.00	2.00
0.00	0.00	3.000	4.00	0.00	0.00	0.00	0.00
0.00	0.00	3.000	4.00	0.00	0.00	0.00	0.00
#NULL!	#NULL!	#NULL!	#NULL!	#NULL!	#NULL!	#NULL!	#NULL!
#NULL!	#NULL!	#NULL!	#NULL!	#NULL!	#NULL!	#NULL!	#NULL!
0.00	0.00	4.000	4.00	0.00	0.00	0.00	0.00
1.00	1.00	2.000	2.00	3.00	1.00	2.00	2.00
0.00	4.00	4.000	4.00	0.00	3.00	3.00	1.00
0.00	0.00	0.000	0.00	0.00	0.00	0.00	0.00
0.00	0.00	3.000	0.00	1.00	1.00	3.00	0.00
1.00	1.00	4.000	3.00	1.00	0.00	0.00	3.00
0.00	0.00	3.000	3.00	2.00	0.00	0.00	0.00
1.00	3.00	2.000	4.00	2.00	1.00	1.00	3.00
0.00	0.00	4.000	4.00	1.00	0.00	1.00	0.00
0.00	2.00	2.000	3.00	1.00	1.00	1.00	1.00
4.00	1.00	2.000	2.00	4.00	4.00	2.00	4.00
1.00	1.00	3.000	2.00	1.00	0.00	1.00	1.00

Tas_10_P3_a	Tas_11_P3_a	Tas_12_P3_a	Tas_13_P3_a	Tas_14_P3_a	Tas_15_P3_a	Tas_16_P3_a	Tas_17_P3_a
2.00	0.00	3.00	0.00	0.00	3.00	1.00	3.00
4.00	999.00	0.00	0.00	1.00	0.00	0.00	0.00
3.00	1.00	1.00	1.00	1.00	2.00	1.00	1.00
4.00	0.00	0.00	0.00	0.00	3.00	3.00	0.00
#NULL!	#NULL!	#NULL!	#NULL!	#NULL!	#NULL!	#NULL!	#NULL!
4.00	0.00	0.00	0.00	0.00	3.00	#NULL!	0.00
2.00	1.00	0.00	0.00	0.00	2.00	3.00	1.00
3.00	1.00	1.00	0.00	0.00	3.00	3.00	1.00
4.00	1.00	1.00	1.00	1.00	1.00	3.00	0.00
4.00	4.00	4.00	0.00	0.00	4.00	1.00	0.00
4.00	0.00	1.00	1.00	0.00	2.00	1.00	1.00
3.00	1.00	1.00	1.00	1.00	1.00	2.00	1.00
3.00	0.00	1.00	0.00	0.00	1.00	1.00	0.00
3.00	1.00	1.00	1.00	1.00	2.00	3.00	1.00
3.00	2.00	1.00	0.00	1.00	3.00	4.00	3.00
#NULL!	#NULL!	#NULL!	#NULL!	#NULL!	#NULL!	#NULL!	#NULL!
3.00	1.00	1.00	1.00	1.00	2.00	1.00	1.00
2.00	0.00	0.00	0.00	0.00	0.00	2.00	4.00
999.00	0.00	0.00	999.00	1.00	4.00	4.00	4.00
3.00	3.00	3.00	2.00	1.00	3.00	2.00	4.00
3.00	0.00	1.00	0.00	0.00	2.00	2.00	1.00
4.00	0.00	1.00	0.00	0.00	0.00	0.00	0.00
2.00	1.00	2.00	1.00	0.00	1.00	2.00	2.00
2.00	2.00	2.00	2.00	2.00	3.00	3.00	2.00
4.00	0.00	1.00	0.00	0.00	1.00	0.00	1.00
4.00	1.00	3.00	1.00	0.00	1.00	1.00	1.00
3.00	1.00	3.00	0.00	4.00	0.00	0.00	0.00
4.00	1.00	0.00	0.00	0.00	2.00	4.00	1.00
3.00	1.00	1.00	0.00	0.00	2.00	2.00	2.00
0.00	4.00	0.00	0.00	0.00	0.00	2.00	3.00
3.00	0.00	0.00	0.00	0.00	2.00	1.00	1.00
3.00	1.00	2.00	0.00	0.00	1.00	2.00	2.00
3.00	1.00	1.00	1.00	1.00	2.00	1.00	1.00
1.00	1.00	0.00	0.00	0.00	4.00	1.00	4.00
#NULL!	#NULL!	#NULL!	#NULL!	#NULL!	#NULL!	#NULL!	#NULL!
#NULL!	#NULL!	#NULL!	#NULL!	#NULL!	#NULL!	#NULL!	#NULL!
#NULL!	#NULL!	#NULL!	#NULL!	#NULL!	#NULL!	#NULL!	#NULL!
#NULL!	#NULL!	#NULL!	#NULL!	#NULL!	#NULL!	#NULL!	#NULL!
4.00	1.00	1.00	1.00	1.00	2.00	1.00	1.00
4.00	1.00	1.00	1.00	1.00	1.00	1.00	1.00
4.00	3.00	0.00	4.00	0.00	2.00	3.00	2.00
3.00	1.00	2.00	0.00	1.00	2.00	2.00	1.00
4.00	0.00	0.00	0.00	1.00	1.00	1.00	0.00
#NULL!	#NULL!	#NULL!	#NULL!	#NULL!	#NULL!	#NULL!	#NULL!
4.00	2.00	1.00	0.00	0.00	0.00	2.00	0.00
3.00	1.00	1.00	0.00	0.00	0.00	0.00	0.00
4.00	0.00	0.00	0.00	0.00	0.00	0.00	0.00
#NULL!	#NULL!	#NULL!	#NULL!	#NULL!	#NULL!	#NULL!	#NULL!
4.00	1.00	0.00	0.00	0.00	1.00	4.00	0.00
4.00	1.00	1.00	0.00	0.00	1.00	2.00	3.00
3.00	1.00	2.00	0.00	1.00	1.00	0.00	1.00
#NULL!	#NULL!	#NULL!	#NULL!	#NULL!	#NULL!	#NULL!	#NULL!
4.00	0.00	3.00	0.00	0.00	0.00	0.00	0.00
4.00	1.00	1.00	0.00	1.00	1.00	0.00	0.00
#NULL!	#NULL!	#NULL!	#NULL!	#NULL!	#NULL!	#NULL!	#NULL!

2.00	1.00	0.00	0.00	0.00	3.00	3.00	4.00
4.00	0.00	3.00	1.00	0.00	1.00	2.00	4.00
3.00	0.00	0.00	0.00	0.00	0.00	3.00	0.00
4.00	0.00	3.00	0.00	0.00	0.00	0.00	0.00
4.00	1.00	0.00	1.00	0.00	0.00	0.00	0.00
#NULL!	#NULL!	#NULL!	#NULL!	#NULL!	#NULL!	#NULL!	#NULL!
3.00	3.00	2.00	0.00	0.00	4.00	2.00	4.00
3.00	0.00	1.00	0.00	0.00	2.00	2.00	0.00
3.00	1.00	2.00	1.00	4.00	0.00	4.00	3.00
2.00	1.00	1.00	2.00	2.00	3.00	3.00	3.00
3.00	0.00	1.00	0.00	0.00	2.00	4.00	1.00
3.00	3.00	0.00	0.00	0.00	0.00	0.00	0.00
4.00	0.00	0.00	0.00	0.00	0.00	0.00	0.00
2.00	2.00	1.00	1.00	2.00	3.00	2.00	3.00
2.00	2.00	1.00	1.00	1.00	1.00	1.00	1.00
4.00	1.00	0.00	0.00	0.00	0.00	0.00	0.00
3.00	1.00	3.00	0.00	1.00	1.00	3.00	1.00
3.00	1.00	2.00	1.00	1.00	2.00	1.00	1.00
2.00	1.00	1.00	999.00	999.00	4.00	1.00	1.00
3.00	2.00	1.00	1.00	1.00	1.00	2.00	1.00
4.00	0.00	0.00	0.00	0.00	0.00	4.00	0.00
3.00	1.00	4.00	0.00	0.00	3.00	0.00	4.00
#NULL!	#NULL!	#NULL!	#NULL!	#NULL!	#NULL!	#NULL!	#NULL!
0.00	0.00	0.00	0.00	0.00	4.00	4.00	4.00
4.00	0.00	0.00	2.00	2.00	2.00	0.00	0.00
2.00	1.00	1.00	1.00	1.00	2.00	2.00	1.00
2.00	2.00	1.00	1.00	0.00	3.00	3.00	3.00
4.00	0.00	2.00	0.00	1.00	1.00	1.00	0.00
2.00	1.00	2.00	1.00	1.00	3.00	2.00	2.00
3.00	1.00	1.00	1.00	1.00	1.00	1.00	2.00
4.00	0.00	1.00	0.00	0.00	2.00	1.00	0.00
4.00	1.00	0.00	1.00	0.00	2.00	0.00	0.00
3.00	0.00	4.00	0.00	0.00	0.00	0.00	0.00
3.00	1.00	1.00	1.00	1.00	1.00	1.00	1.00
4.00	0.00	2.00	0.00	1.00	3.00	2.00	2.00
4.00	1.00	1.00	1.00	1.00	1.00	1.00	1.00
1.00	1.00	1.00	1.00	1.00	1.00	3.00	1.00
3.00	3.00	1.00	2.00	1.00	3.00	2.00	1.00
#NULL!	#NULL!	#NULL!	#NULL!	#NULL!	#NULL!	#NULL!	#NULL!
3.00	2.00	1.00	1.00	1.00	3.00	3.00	3.00
4.00	1.00	1.00	1.00	0.00	2.00	2.00	1.00
2.00	1.00	1.00	1.00	0.00	2.00	3.00	3.00
3.00	0.00	1.00	0.00	1.00	2.00	0.00	3.00
4.00	1.00	1.00	1.00	1.00	1.00	1.00	1.00
3.00	2.00	0.00	3.00	1.00	2.00	2.00	2.00
0.00	0.00	0.00	0.00	0.00	1.00	0.00	0.00
2.00	1.00	2.00	1.00	1.00	2.00	2.00	3.00
3.00	1.00	1.00	1.00	1.00	1.00	2.00	2.00
3.00	0.00	3.00	0.00	0.00	0.00	1.00	0.00
2.00	2.00	3.00	2.00	1.00	2.00	3.00	2.00
#NULL!	#NULL!	#NULL!	#NULL!	#NULL!	#NULL!	#NULL!	#NULL!
3.00	0.00	0.00	0.00	0.00	2.00	2.00	1.00
0.00	1.00	1.00	1.00	1.00	3.00	3.00	1.00
4.00	1.00	1.00	1.00	2.00	1.00	1.00	3.00
#NULL!	#NULL!	#NULL!	#NULL!	#NULL!	#NULL!	#NULL!	#NULL!
2.00	1.00	0.00	0.00	0.00	0.00	1.00	1.00

3.00	1.00	1.00	1.00	0.00	1.00	3.00	0.00
3.00	1.00	2.00	0.00	1.00	2.00	1.00	1.00
2.00	0.00	0.00	0.00	0.00	1.00	2.00	0.00
#NULL!	#NULL!	#NULL!	#NULL!	#NULL!	#NULL!	#NULL!	#NULL!
3.00	1.00	1.00	1.00	1.00	1.00	1.00	1.00
3.00	1.00	1.00	1.00	1.00	1.00	1.00	1.00
3.00	3.00	3.00	1.00	1.00	3.00	3.00	3.00
3.00	0.00	0.00	0.00	0.00	1.00	2.00	0.00
#NULL!	#NULL!	#NULL!	#NULL!	#NULL!	#NULL!	#NULL!	#NULL!
4.00	2.00	1.00	0.00	0.00	0.00	1.00	3.00
4.00	0.00	1.00	0.00	0.00	1.00	1.00	1.00
4.00	0.00	2.00	0.00	0.00	2.00	3.00	1.00
1.00	1.00	0.00	1.00	1.00	3.00	0.00	3.00
2.00	1.00	1.00	1.00	1.00	2.00	2.00	1.00
3.00	0.00	2.00	0.00	0.00	2.00	3.00	#NULL!
#NULL!	#NULL!	#NULL!	#NULL!	#NULL!	#NULL!	#NULL!	#NULL!
2.00	1.00	0.00	0.00	1.00	1.00	1.00	1.00
#NULL!	#NULL!	#NULL!	#NULL!	#NULL!	#NULL!	#NULL!	#NULL!
3.00	0.00	0.00	0.00	1.00	0.00	0.00	1.00
3.00	1.00	2.00	1.00	3.00	3.00	1.00	1.00
#NULL!	#NULL!	#NULL!	#NULL!	#NULL!	#NULL!	#NULL!	#NULL!
3.00	0.00	2.00	0.00	0.00	4.00	3.00	3.00
#NULL!	#NULL!	#NULL!	#NULL!	#NULL!	#NULL!	#NULL!	#NULL!
0.00	0.00	1.00	1.00	2.00	2.00	3.00	2.00
3.00	1.00	1.00	3.00	1.00	3.00	1.00	1.00
2.00	0.00	0.00	3.00	2.00	2.00	0.00	0.00
4.00	1.00	1.00	3.00	1.00	1.00	3.00	0.00
3.00	1.00	1.00	0.00	0.00	1.00	1.00	0.00
#NULL!	#NULL!	#NULL!	#NULL!	#NULL!	#NULL!	#NULL!	#NULL!
#NULL!	#NULL!	#NULL!	#NULL!	#NULL!	#NULL!	#NULL!	#NULL!
4.00	0.00	2.00	0.00	0.00	1.00	3.00	0.00
3.00	0.00	1.00	1.00	0.00	1.00	2.00	1.00
4.00	2.00	1.00	3.00	0.00	3.00	0.00	0.00
2.00	2.00	1.00	0.00	1.00	2.00	1.00	3.00
3.00	2.00	2.00	2.00	3.00	3.00	2.00	1.00
4.00	1.00	3.00	0.00	0.00	3.00	1.00	3.00
#NULL!	#NULL!	#NULL!	#NULL!	#NULL!	#NULL!	#NULL!	#NULL!
3.00	1.00	3.00	1.00	1.00	1.00	1.00	3.00
1.00	1.00	3.00	3.00	1.00	3.00	4.00	4.00
#NULL!	#NULL!	#NULL!	#NULL!	#NULL!	#NULL!	#NULL!	#NULL!
4.00	3.00	3.00	3.00	2.00	0.00	0.00	1.00
4.00	2.00	1.00	2.00	1.00	1.00	0.00	1.00
4.00	2.00	2.00	3.00	3.00	1.00	1.00	2.00
3.00	1.00	1.00	1.00	1.00	1.00	1.00	0.00
3.00	1.00	2.00	0.00	0.00	2.00	2.00	0.00
3.00	0.00	0.00	0.00	0.00	1.00	0.00	0.00
4.00	3.00	1.00	2.00	3.00	2.00	1.00	2.00
4.00	1.00	1.00	1.00	0.00	1.00	1.00	3.00
999.00	999.00	999.00	1.00	0.00	0.00	2.00	0.00
4.00	1.00	3.00	1.00	1.00	1.00	2.00	1.00
2.00	0.00	1.00	1.00	1.00	4.00	1.00	1.00
#NULL!	#NULL!	#NULL!	#NULL!	#NULL!	#NULL!	#NULL!	#NULL!
3.00	0.00	0.00	0.00	0.00	1.00	0.00	1.00
3.00	1.00	1.00	1.00	0.00	2.00	2.00	1.00
4.00	1.00	2.00	1.00	1.00	1.00	1.00	2.00
4.00	1.00	1.00	1.00	1.00	1.00	1.00	1.00

3.00	1.00	0.00	0.00	1.00	2.00	4.00	0.00
4.00	0.00	0.00	0.00	0.00	2.00	1.00	0.00
4.00	0.00	0.00	0.00	0.00	0.00	0.00	0.00
3.00	1.00	1.00	0.00	0.00	2.00	0.00	0.00
#NULL!	#NULL!	#NULL!	#NULL!	#NULL!	#NULL!	#NULL!	#NULL!
4.00	0.00	0.00	0.00	0.00	0.00	1.00	0.00
4.00	1.00	1.00	1.00	1.00	2.00	2.00	3.00
4.00	0.00	0.00	0.00	0.00	0.00	0.00	0.00
0.00	1.00	0.00	2.00	0.00	4.00	3.00	4.00
3.00	1.00	1.00	2.00	3.00	1.00	1.00	3.00
2.00	1.00	2.00	1.00	1.00	3.00	4.00	2.00
4.00	1.00	0.00	0.00	0.00	0.00	2.00	0.00
4.00	0.00	0.00	1.00	1.00	1.00	0.00	3.00
3.00	1.00	3.00	999.00	3.00	3.00	1.00	1.00
1.00	1.00	0.00	0.00	0.00	0.00	4.00	4.00
#NULL!	#NULL!	#NULL!	#NULL!	#NULL!	#NULL!	#NULL!	#NULL!
3.00	1.00	1.00	2.00	1.00	2.00	1.00	1.00
4.00	1.00	2.00	1.00	1.00	2.00	0.00	2.00
4.00	1.00	1.00	1.00	0.00	2.00	3.00	3.00
#NULL!	#NULL!	#NULL!	#NULL!	#NULL!	#NULL!	#NULL!	#NULL!
4.00	0.00	0.00	0.00	2.00	4.00	2.00	4.00
#NULL!	#NULL!	#NULL!	#NULL!	#NULL!	#NULL!	#NULL!	#NULL!
3.00	1.00	1.00	1.00	0.00	3.00	3.00	2.00
3.00	1.00	1.00	0.00	0.00	1.00	2.00	0.00
4.00	0.00	0.00	4.00	0.00	2.00	4.00	0.00
4.00	0.00	1.00	1.00	1.00	1.00	2.00	1.00
4.00	1.00	2.00	1.00	1.00	1.00	1.00	1.00
3.00	0.00	0.00	0.00	0.00	1.00	3.00	0.00
4.00	1.00	1.00	1.00	1.00	1.00	2.00	2.00
4.00	0.00	4.00	1.00	1.00	0.00	2.00	3.00
4.00	2.00	0.00	1.00	1.00	1.00	1.00	0.00
#NULL!	#NULL!	#NULL!	#NULL!	#NULL!	#NULL!	#NULL!	#NULL!
2.00	2.00	1.00	3.00	2.00	3.00	1.00	4.00
#NULL!	#NULL!	#NULL!	#NULL!	#NULL!	#NULL!	#NULL!	#NULL!
2.00	1.00	2.00	1.00	4.00	2.00	1.00	4.00
3.00	1.00	2.00	1.00	1.00	2.00	1.00	1.00
#NULL!	#NULL!	#NULL!	#NULL!	#NULL!	#NULL!	#NULL!	#NULL!
4.00	1.00	0.00	3.00	2.00	1.00	1.00	1.00
3.00	3.00	3.00	3.00	3.00	4.00	0.00	4.00
4.00	0.00	1.00	0.00	0.00	2.00	1.00	1.00
#NULL!	#NULL!	#NULL!	#NULL!	#NULL!	#NULL!	#NULL!	#NULL!
3.00	1.00	2.00	1.00	2.00	1.00	1.00	1.00
0.00	1.00	0.00	0.00	1.00	1.00	1.00	4.00
4.00	0.00	3.00	0.00	0.00	3.00	0.00	3.00
3.00	0.00	2.00	1.00	1.00	1.00	1.00	1.00
#NULL!	#NULL!	#NULL!	#NULL!	#NULL!	#NULL!	#NULL!	#NULL!
4.00	0.00	0.00	0.00	0.00	1.00	2.00	0.00
3.00	0.00	1.00	0.00	0.00	3.00	3.00	1.00
4.00	0.00	1.00	0.00	0.00	0.00	0.00	0.00
#NULL!	#NULL!	#NULL!	#NULL!	#NULL!	#NULL!	#NULL!	#NULL!
4.00	1.00	1.00	1.00	1.00	1.00	1.00	1.00
3.00	1.00	1.00	1.00	2.00	1.00	1.00	3.00
2.00	1.00	0.00	0.00	1.00	2.00	4.00	1.00
2.00	1.00	0.00	0.00	3.00	2.00	4.00	3.00
#NULL!	#NULL!	#NULL!	#NULL!	#NULL!	#NULL!	#NULL!	#NULL!
3.00	1.00	2.00	1.00	1.00	1.00	1.00	1.00

3.00	1.00	1.00	1.00	1.00	3.00	3.00	1.00
3.00	1.00	1.00	1.00	0.00	1.00	2.00	0.00
3.00	2.00	3.00	1.00	2.00	3.00	2.00	3.00
3.00	2.00	2.00	3.00	2.00	3.00	2.00	1.00
3.00	2.00	2.00	1.00	1.00	2.00	3.00	1.00
3.00	1.00	2.00	1.00	0.00	1.00	3.00	1.00
4.00	0.00	1.00	1.00	0.00	1.00	1.00	0.00
#NULL!	#NULL!	#NULL!	#NULL!	#NULL!	#NULL!	#NULL!	#NULL!
4.00	1.00	1.00	1.00	1.00	1.00	1.00	1.00
2.00	1.00	1.00	1.00	2.00	1.00	3.00	1.00
4.00	0.00	0.00	0.00	0.00	0.00	0.00	0.00
#NULL!	#NULL!	#NULL!	#NULL!	#NULL!	#NULL!	#NULL!	#NULL!
#NULL!	#NULL!	#NULL!	#NULL!	#NULL!	#NULL!	#NULL!	#NULL!
3.00	1.00	2.00	2.00	1.00	1.00	0.00	2.00
2.00	2.00	2.00	2.00	2.00	3.00	2.00	1.00
3.00	2.00	1.00	0.00	0.00	0.00	1.00	2.00
999.00	1.00	1.00	1.00	2.00	1.00	1.00	2.00
2.00	1.00	2.00	1.00	1.00	2.00	2.00	2.00
4.00	1.00	2.00	2.00	1.00	4.00	4.00	0.00
1.00	0.00	1.00	0.00	0.00	1.00	0.00	1.00
4.00	0.00	0.00	1.00	0.00	1.00	2.00	0.00
3.00	1.00	1.00	1.00	1.00	1.00	1.00	2.00
4.00	1.00	0.00	3.00	0.00	2.00	1.00	0.00
2.00	3.00	0.00	0.00	3.00	3.00	4.00	1.00
3.00	1.00	1.00	2.00	1.00	2.00	3.00	3.00
2.00	2.00	1.00	3.00	3.00	3.00	4.00	4.00
2.00	3.00	2.00	2.00	1.00	4.00	2.00	3.00
2.00	1.00	1.00	1.00	0.00	3.00	3.00	2.00
4.00	1.00	1.00	0.00	0.00	1.00	0.00	1.00
4.00	1.00	1.00	1.00	1.00	2.00	3.00	1.00
2.00	2.00	1.00	2.00	2.00	2.00	4.00	0.00
4.00	0.00	3.00	3.00	0.00	4.00	4.00	4.00
1.00	1.00	1.00	0.00	1.00	2.00	1.00	1.00
3.00	2.00	0.00	2.00	0.00	2.00	2.00	0.00
1.00	0.00	0.00	0.00	0.00	0.00	1.00	0.00
#NULL!	#NULL!	#NULL!	#NULL!	#NULL!	#NULL!	#NULL!	#NULL!
0.00	1.00	2.00	0.00	0.00	2.00	4.00	4.00
0.00	0.00	0.00	0.00	0.00	1.00	0.00	0.00
2.00	1.00	1.00	0.00	1.00	2.00	0.00	0.00
3.00	1.00	1.00	0.00	0.00	1.00	0.00	1.00
3.00	0.00	1.00	3.00	1.00	2.00	0.00	1.00
1.00	3.00	0.00	3.00	1.00	3.00	3.00	4.00
3.00	2.00	2.00	3.00	1.00	1.00	3.00	2.00
4.00	0.00	1.00	0.00	1.00	2.00	2.00	3.00
3.00	1.00	1.00	0.00	0.00	0.00	2.00	0.00
#NULL!	#NULL!	#NULL!	#NULL!	#NULL!	#NULL!	#NULL!	#NULL!
1.00	1.00	1.00	2.00	1.00	2.00	1.00	2.00
3.00	3.00	1.00	2.00	1.00	3.00	3.00	3.00
3.00	1.00	1.00	1.00	0.00	1.00	3.00	1.00
4.00	1.00	0.00	0.00	0.00	1.00	1.00	1.00
4.00	1.00	2.00	2.00	1.00	0.00	1.00	1.00
4.00	0.00	1.00	1.00	1.00	1.00	0.00	0.00
2.00	1.00	1.00	1.00	1.00	3.00	2.00	3.00
#NULL!	#NULL!	#NULL!	#NULL!	#NULL!	#NULL!	#NULL!	#NULL!
4.00	2.00	0.00	3.00	3.00	2.00	1.00	1.00
3.00	1.00	1.00	1.00	1.00	1.00	1.00	2.00

4.00	0.00	2.00	0.00	0.00	1.00	1.00	1.00
3.00	1.00	2.00	1.00	1.00	3.00	1.00	1.00
4.00	2.00	0.00	0.00	0.00	3.00	0.00	3.00
4.00	0.00	0.00	0.00	0.00	1.00	2.00	1.00
#NULL!	#NULL!	#NULL!	#NULL!	#NULL!	#NULL!	#NULL!	#NULL!
#NULL!	#NULL!	#NULL!	#NULL!	#NULL!	#NULL!	#NULL!	#NULL!
1.00	0.00	0.00	0.00	0.00	0.00	1.00	0.00
4.00	0.00	1.00	0.00	2.00	1.00	2.00	1.00
4.00	0.00	0.00	0.00	0.00	0.00	2.00	0.00
2.00	0.00	0.00	0.00	0.00	0.00	0.00	0.00
2.00	0.00	0.00	0.00	0.00	2.00	3.00	3.00
4.00	0.00	1.00	1.00	1.00	3.00	4.00	3.00
3.00	0.00	0.00	3.00	0.00	2.00	0.00	2.00
3.00	1.00	2.00	1.00	3.00	4.00	1.00	3.00
3.00	0.00	0.00	0.00	0.00	1.00	0.00	1.00
3.00	2.00	1.00	1.00	1.00	2.00	2.00	2.00
2.00	4.00	4.00	4.00	4.00	4.00	3.00	3.00
3.00	1.00	1.00	1.00	1.00	1.00	3.00	1.00

Tas_18_P3_ε	Tas_19_P3_ε	Tas_20_P3_ε	P_Activity_P3	Height_P3	Weight_P3
3.00	2.00	0.00	#NULL!	#NULL!	#NULL!
4.00	4.00	0.00	55.00	163.00	81.00
3.00	3.00	2.00	#NULL!	#NULL!	#NULL!
3.00	3.00	0.00	3.00	178.00	84.00
#NULL!	#NULL!	#NULL!	#NULL!	#NULL!	#NULL!
2.00	0.00	0.00	10.00	181.00	85.00
3.00	2.00	2.00	8.00	162.00	63.00
3.00	3.00	1.00	#NULL!	#NULL!	#NULL!
0.00	0.00	1.00	8.00	175.00	80.00
4.00	4.00	3.00	#NULL!	#NULL!	#NULL!
4.00	4.00	1.00	7.00	159.00	74.80
3.00	3.00	1.00	6.50	153.00	61.00
4.00	4.00	0.00	3.00	155.00	68.00
3.00	3.00	3.00	10.00	184.00	92.00
3.00	2.00	1.00	#NULL!	#NULL!	#NULL!
#NULL!	#NULL!	#NULL!	#NULL!	#NULL!	#NULL!
3.00	3.00	2.00	4.00	152.00	64.00
0.00	2.00	0.00	5.00	157.00	62.10
2.00	2.00	4.00	#NULL!	#NULL!	#NULL!
3.00	1.00	4.00	0.00	162.00	62.00
3.00	2.00	2.00	6.00	172.00	86.60
4.00	4.00	0.00	#NULL!	#NULL!	#NULL!
2.00	2.00	2.00	#NULL!	#NULL!	#NULL!
2.00	2.00	2.00	#NULL!	#NULL!	#NULL!
1.00	4.00	0.00	1.00	163.00	61.40
3.00	4.00	1.00	10.00	158.00	61.50
4.00	4.00	4.00	#NULL!	#NULL!	#NULL!
3.00	3.00	4.00	#NULL!	#NULL!	#NULL!
3.00	1.00	1.00	0.00	180.00	92.00
0.00	2.00	1.00	10.00	147.00	58.00
2.00	2.00	1.00	#NULL!	#NULL!	#NULL!
1.00	3.00	1.00	5.00	182.00	73.00
3.00	3.00	3.00	2.00	158.00	56.00
3.00	1.00	4.00	2.00	173.00	85.20
#NULL!	#NULL!	#NULL!	1.00	125.00	81.00
#NULL!	#NULL!	#NULL!	#NULL!	#NULL!	#NULL!
#NULL!	#NULL!	#NULL!	#NULL!	#NULL!	#NULL!
#NULL!	#NULL!	#NULL!	#NULL!	#NULL!	#NULL!
3.00	3.00	1.00	4.00	158.00	68.10
3.00	3.00	1.00	1.00	165.00	67.00
4.00	2.00	2.00	#NULL!	#NULL!	#NULL!
3.00	3.00	0.00	3.00	150.00	57.00
3.00	2.00	0.00	#NULL!	#NULL!	#NULL!
#NULL!	#NULL!	#NULL!	#NULL!	#NULL!	#NULL!
3.00	3.00	1.00	#NULL!	#NULL!	#NULL!
0.00	1.00	2.00	#NULL!	#NULL!	#NULL!
4.00	4.00	1.00	#NULL!	#NULL!	#NULL!
#NULL!	#NULL!	#NULL!	#NULL!	#NULL!	#NULL!
4.00	3.00	0.00	2.00	179.00	79.00
4.00	4.00	0.00	7.00	160.00	62.00
3.00	4.00	0.00	#NULL!	#NULL!	#NULL!
#NULL!	#NULL!	#NULL!	#NULL!	#NULL!	#NULL!
4.00	4.00	0.00	2.00	157.00	56.10
3.00	4.00	0.00	2.00	172.00	82.00
#NULL!	#NULL!	#NULL!	#NULL!	#NULL!	#NULL!

3.00	2.00	3.00	5.00	161.00	64.00
3.00	4.00	0.00	5.00	163.00	70.40
3.00	3.00	0.00	3.00	169.00	158.00
4.00	0.00	0.00	10.00	178.00	73.00
0.00	0.00	0.00	#NULL!	#NULL!	#NULL!
#NULL!	#NULL!	#NULL!	#NULL!	#NULL!	#NULL!
2.00	2.00	4.00	5.00	176.00	69.00
4.00	3.00	1.00	5.00	184.00	90.00
3.00	2.00	1.00	2.50	156.00	98.00
3.00	2.00	3.00	8.00	173.00	62.00
2.00	2.00	4.00	5.00	172.00	74.00
0.00	0.00	2.00	4.00	149.00	58.00
4.00	4.00	2.00	2.00	158.00	71.00
2.00	2.00	1.00	#NULL!	#NULL!	#NULL!
2.00	2.00	1.00	4.00	165.00	64.00
4.00	4.00	0.00	#NULL!	#NULL!	#NULL!
3.00	3.00	4.00	10.00	180.00	70.10
3.00	3.00	2.00	6.00	167.00	66.50
2.00	2.00	1.00	8.00	168.00	68.90
3.00	3.00	1.00	0.00	163.00	73.00
4.00	0.00	0.00	5.00	183.00	92.00
3.00	3.00	0.00	3.00	173.00	89.00
#NULL!	#NULL!	#NULL!	#NULL!	#NULL!	#NULL!
4.00	0.00	4.00	0.00	170.00	76.00
4.00	4.00	0.00	6.00	167.00	94.60
3.00	3.00	1.00	5.00	170.00	79.40
3.00	2.00	1.00	#NULL!	#NULL!	#NULL!
3.00	4.00	0.00	3.00	171.00	77.00
3.00	2.00	3.00	3.00	168.00	73.00
3.00	3.00	3.00	0.00	166.00	76.00
3.00	3.00	0.00	2.00	180.00	95.00
4.00	4.00	1.00	5.00	183.00	80.00
4.00	4.00	0.00	10.00	156.00	55.00
3.00	3.00	3.00	#NULL!	#NULL!	#NULL!
3.00	3.00	4.00	#NULL!	#NULL!	#NULL!
4.00	4.00	1.00	3.00	167.00	81.00
3.00	2.00	1.00	2.50	172.00	85.00
3.00	3.00	1.00	6.00	168.00	63.40
#NULL!	#NULL!	#NULL!	3.00	178.00	88.00
3.00	3.00	3.00	6.00	170.00	69.00
0.00	3.00	1.00	2.00	171.00	81.00
3.00	1.00	3.00	#NULL!	#NULL!	#NULL!
3.00	3.00	3.00	4.00	167.00	78.00
4.00	4.00	1.00	4.00	165.00	93.00
2.00	3.00	2.00	0.00	164.00	87.30
4.00	2.00	1.00	6.00	169.00	88.50
1.00	3.00	1.00	7.00	163.00	63.90
4.00	1.00	1.00	4.50	168.00	93.00
2.00	3.00	2.00	5.00	159.00	47.48
3.00	3.00	3.00	7.00	172.00	80.00
#NULL!	#NULL!	#NULL!	#NULL!	#NULL!	#NULL!
2.00	3.00	2.00	#NULL!	#NULL!	#NULL!
2.00	1.00	3.00	#NULL!	#NULL!	#NULL!
4.00	4.00	0.00	10.00	165.00	68.20
#NULL!	#NULL!	#NULL!	1.00	165.00	67.00
4.00	2.00	1.00	3.00	161.00	74.00

3.00	4.00	1.00	1.00	159.00	71.90
3.00	3.00	2.00	22.00	175.00	73.00
4.00	1.00	0.00	#NULL!	#NULL!	#NULL!
#NULL!	#NULL!	#NULL!	#NULL!	#NULL!	#NULL!
3.00	3.00	1.00	7.00	172.00	67.00
4.00	#NULL!	3.00	#NULL!	#NULL!	#NULL!
1.00	3.00	3.00	3.00	173.00	82.60
4.00	4.00	1.00	4.00	153.00	55.00
#NULL!	#NULL!	#NULL!	#NULL!	#NULL!	#NULL!
4.00	3.00	0.00	0.00	162.00	70.00
3.00	4.00	0.00	#NULL!	#NULL!	#NULL!
3.00	2.00	0.00	7.00	152.00	49.80
4.00	3.00	1.00	5.00	157.00	81.00
3.00	3.00	2.00	0.00	173.00	93.00
3.00	3.00	4.00	4.00	188.00	82.00
#NULL!	#NULL!	#NULL!	#NULL!	#NULL!	#NULL!
3.00	2.00	0.00	5.00	172.00	82.00
#NULL!	#NULL!	#NULL!	#NULL!	#NULL!	#NULL!
4.00	4.00	2.00	#NULL!	#NULL!	#NULL!
3.00	3.00	2.00	6.00	162.00	69.70
#NULL!	#NULL!	#NULL!	0.00	168.00	67.30
4.00	1.00	1.00	5.00	174.00	67.00
#NULL!	#NULL!	#NULL!	#NULL!	#NULL!	#NULL!
1.00	2.00	3.00	2.00	172.00	87.00
3.00	3.00	3.00	10.00	164.00	66.50
3.00	3.00	1.00	3.00	174.00	65.30
3.00	4.00	3.00	#NULL!	#NULL!	#NULL!
3.00	3.00	1.00	#NULL!	#NULL!	#NULL!
#NULL!	#NULL!	#NULL!	#NULL!	#NULL!	#NULL!
#NULL!	#NULL!	#NULL!	#NULL!	#NULL!	#NULL!
3.00	3.00	0.00	2.00	172.00	77.00
4.00	4.00	3.00	10.00	164.00	81.00
4.00	4.00	4.00	3.00	163.00	80.00
3.00	2.00	1.00	#NULL!	#NULL!	#NULL!
2.00	2.00	2.00	7.00	172.00	84.00
4.00	4.00	0.00	7.00	162.00	66.00
#NULL!	#NULL!	#NULL!	#NULL!	#NULL!	#NULL!
3.00	3.00	3.00	1.00	168.00	61.00
2.00	3.00	4.00	#NULL!	#NULL!	#NULL!
#NULL!	#NULL!	#NULL!	#NULL!	#NULL!	#NULL!
4.00	4.00	2.00	#NULL!	#NULL!	#NULL!
3.00	3.00	0.00	3.00	170.00	61.20
2.00	2.00	0.00	7.00	178.00	59.90
3.00	2.00	2.00	#NULL!	#NULL!	#NULL!
3.00	4.00	0.00	8.00	160.00	57.00
3.00	3.00	0.00	5.00	150.00	51.00
3.00	3.00	0.00	5.00	175.00	73.50
3.00	3.00	0.00	8.00	150.00	51.00
4.00	2.00	1.00	#NULL!	#NULL!	#NULL!
4.00	1.00	1.00	2.00	160.00	84.30
3.00	2.00	2.00	3.00	180.00	84.50
#NULL!	#NULL!	#NULL!	#NULL!	#NULL!	#NULL!
4.00	3.00	0.00	0.00	164.00	67.00
3.00	3.00	1.00	10.00	184.00	90.80
4.00	3.00	4.00	5.00	168.00	75.40
4.00	3.00	0.00	3.00	158.00	54.50

3.00	3.00	0.00	#NULL!	#NULL!	#NULL!
2.00	4.00	0.00	3.00	162.00	72.30
0.00	4.00	0.00	0.00	184.00	110.00
0.00	0.00	2.00	0.00	155.00	86.30
#NULL!	#NULL!	#NULL!	#NULL!	#NULL!	#NULL!
4.00	4.00	3.00	4.00	155.00	66.80
1.00	1.00	3.00	#NULL!	#NULL!	#NULL!
4.00	4.00	0.00	4.50	168.00	86.00
0.00	0.00	0.00	0.00	165.00	65.00
3.00	3.00	1.00	10.00	168.00	71.90
2.00	3.00	0.00	5.00	155.00	81.70
3.00	2.00	0.00	1.00	186.00	110.00
2.00	1.00	0.00	0.00	157.00	59.50
3.00	3.00	2.00	#NULL!	#NULL!	#NULL!
4.00	0.00	0.00	#NULL!	#NULL!	#NULL!
#NULL!	#NULL!	#NULL!	#NULL!	#NULL!	#NULL!
3.00	2.00	2.00	#NULL!	#NULL!	#NULL!
3.00	3.00	0.00	7.00	170.00	78.00
4.00	3.00	1.00	#NULL!	#NULL!	#NULL!
#NULL!	#NULL!	#NULL!	#NULL!	#NULL!	#NULL!
4.00	4.00	2.00	6.00	149.00	58.00
#NULL!	#NULL!	#NULL!	#NULL!	#NULL!	#NULL!
4.00	2.00	3.00	5.00	191.00	107.00
4.00	3.00	2.00	#NULL!	#NULL!	#NULL!
2.00	3.00	0.00	#NULL!	#NULL!	#NULL!
4.00	4.00	1.00	#NULL!	#NULL!	#NULL!
3.00	4.00	2.00	#NULL!	#NULL!	#NULL!
4.00	4.00	0.00	10.00	164.00	85.00
3.00	4.00	0.00	3.00	162.00	69.00
4.00	3.00	2.00	4.00	188.00	74.00
4.00	3.00	1.00	0.00	172.00	97.00
#NULL!	#NULL!	#NULL!	#NULL!	#NULL!	#NULL!
3.00	3.00	3.00	3.00	172.00	81.00
#NULL!	#NULL!	#NULL!	#NULL!	#NULL!	#NULL!
4.00	2.00	0.00	6.00	171.00	70.00
3.00	3.00	0.00	7.00	158.00	59.00
#NULL!	#NULL!	#NULL!	#NULL!	#NULL!	#NULL!
3.00	3.00	1.00	4.00	175.00	83.00
4.00	1.00	3.00	8.00	170.00	69.00
2.00	2.00	0.00	2.00	158.00	70.00
#NULL!	#NULL!	#NULL!	#NULL!	#NULL!	#NULL!
3.00	2.00	1.00	33.00	158.00	54.00
4.00	1.00	4.00	#NULL!	#NULL!	#NULL!
4.00	4.00	0.00	5.00	156.50	61.20
3.00	4.00	1.00	1.00	162.00	68.00
#NULL!	#NULL!	#NULL!	#NULL!	#NULL!	#NULL!
4.00	3.00	1.00	#NULL!	#NULL!	#NULL!
3.00	3.00	1.00	7.00	161.00	77.00
4.00	4.00	0.00	#NULL!	#NULL!	#NULL!
#NULL!	#NULL!	#NULL!	0.00	165.00	74.00
4.00	3.00	1.00	#NULL!	#NULL!	#NULL!
999.00	3.00	2.00	#NULL!	#NULL!	#NULL!
2.00	2.00	2.00	#NULL!	#NULL!	#NULL!
3.00	3.00	0.00	3.00	179.00	71.00
#NULL!	#NULL!	#NULL!	#NULL!	#NULL!	#NULL!
4.00	1.00	3.00	#NULL!	#NULL!	#NULL!

3.00	3.00	3.00	5.00	161.00	75.30
3.00	3.00	3.00	#NULL!	#NULL!	#NULL!
3.00	3.00	1.00	2.00	174.00	86.20
3.00	3.00	1.00	#NULL!	#NULL!	#NULL!
3.00	3.00	1.00	2.00	178.00	78.00
3.00	2.00	1.00	3.00	158.00	59.00
3.00	0.00	0.00	4.00	165.00	69.00
#NULL!	#NULL!	#NULL!	#NULL!	#NULL!	#NULL!
3.00	1.00	0.00	1.00	161.00	84.30
4.00	2.00	2.00	#NULL!	#NULL!	#NULL!
3.00	3.00	1.00	6.00	163.00	70.00
#NULL!	#NULL!	#NULL!	#NULL!	#NULL!	#NULL!
#NULL!	#NULL!	#NULL!	#NULL!	#NULL!	#NULL!
3.00	2.00	1.00	#NULL!	#NULL!	#NULL!
3.00	2.00	2.00	0.00	180.00	88.70
3.00	3.00	2.00	0.00	176.00	120.00
2.00	4.00	1.00	6.00	158.00	55.00
2.00	1.00	2.00	2.00	190.00	92.00
4.00	4.00	1.00	#NULL!	#NULL!	#NULL!
4.00	4.00	1.00	#NULL!	#NULL!	#NULL!
4.00	3.00	1.00	0.00	161.00	62.00
3.00	3.00	1.00	10.00	160.00	63.00
3.00	1.00	1.00	0.00	173.00	81.00
3.00	999.00	1.00	#NULL!	#NULL!	#NULL!
3.00	3.00	1.00	2.50	159.00	87.30
0.00	2.00	2.00	6.00	159.00	64.00
3.00	2.00	1.00	5.50	170.00	60.00
2.00	1.00	2.00	2.00	158.00	110.00
3.00	4.00	0.00	4.50	170.00	50.00
4.00	1.00	0.00	0.00	157.00	62.00
2.00	3.00	1.00	2.00	158.00	65.00
4.00	3.00	3.00	#NULL!	#NULL!	#NULL!
3.00	3.00	2.00	4.00	160.00	65.00
1.00	2.00	4.00	#NULL!	#NULL!	#NULL!
2.00	3.00	0.00	2.00	181.00	88.00
#NULL!	#NULL!	#NULL!	#NULL!	#NULL!	#NULL!
3.00	2.00	4.00	#NULL!	#NULL!	#NULL!
4.00	4.00	0.00	6.00	165.00	68.00
3.00	3.00	1.00	2.00	161.00	61.70
3.00	3.00	0.00	2.00	162.00	66.00
4.00	2.00	0.00	#NULL!	#NULL!	#NULL!
3.00	1.00	1.00	0.00	165.00	83.00
2.00	2.00	1.00	#NULL!	#NULL!	#NULL!
3.00	3.00	1.00	0.00	162.00	80.00
4.00	4.00	3.00	8.00	180.00	82.00
#NULL!	#NULL!	#NULL!	#NULL!	#NULL!	#NULL!
2.00	1.00	1.00	#NULL!	#NULL!	#NULL!
4.00	2.00	1.00	0.00	163.00	68.30
3.00	3.00	1.00	0.00	156.00	58.00
4.00	4.00	0.00	3.00	159.00	57.00
3.00	4.00	0.00	2.00	163.00	69.00
4.00	3.00	1.00	3.00	164.00	59.50
3.00	2.00	1.00	#NULL!	#NULL!	#NULL!
#NULL!	#NULL!	#NULL!	0.00	160.00	58.00
1.00	3.00	1.00	22.00	172.00	69.00
3.00	3.00	1.00	2.00	159.00	2.00

3.00	4.00	0.00	2.00	163.00	58.30
3.00	4.00	2.00	3.00	180.00	100.00
3.00	3.00	0.00	5.00	174.00	76.00
4.00	4.00	0.00	#NULL!	#NULL!	#NULL!
#NULL!	#NULL!	#NULL!	#NULL!	#NULL!	#NULL!
#NULL!	#NULL!	#NULL!	#NULL!	#NULL!	#NULL!
0.00	0.00	0.00	0.00	174.00	110.00
3.00	2.00	1.00	#NULL!	#NULL!	#NULL!
4.00	3.00	0.00	#NULL!	#NULL!	#NULL!
3.00	3.00	3.00	#NULL!	#NULL!	#NULL!
1.00	1.00	3.00	4.00	180.00	80.00
4.00	1.00	3.00	3.00	168.00	71.40
3.00	3.00	0.00	2.00	184.00	86.00
4.00	4.00	3.00	#NULL!	#NULL!	#NULL!
3.00	3.00	0.00	5.00	155.00	85.00
2.00	3.00	2.00	3.00	158.00	60.00
4.00	3.00	4.00	#NULL!	#NULL!	#NULL!
3.00	2.00	1.00	#NULL!	#NULL!	#NULL!

Chronic_Dis_P3	extroversion_P3	agreeableness_P3	consciencious_P3
	3.71	2.25	2.71
0	4.00	4.25	2.71
	2.43	3.25	4.29
0	3.29	3.88	4.00
	2.57	4.00	4.86
0	3.25	4.25	4.43
0	3.29	4.38	3.86
	3.57	3.38	4.43
hypertension, colesterol	3.14	4.25	3.29
	3.43	4.38	4.14
hypertension, blood lipids	3.14	4.25	4.14
hypertension	3.57	3.50	3.14
0	2.86	4.63	3.14
colesterol	3.86	3.63	4.14
	2.57	3.75	3.43
	3.29	3.75	4.29
0	3.29	4.00	4.57
Hypertension	2.00	3.75	4.00
	3.67	3.00	2.67
Osteoporosis, blood pressure, cholesterol	2.29	4.25	3.29
0	4.29	3.88	3.86
	3.71	3.00	4.14
	2.57	3.75	4.57
	3.29	3.88	3.43
לפני כשנתיים תקף וירוס את המערכת הוסטיבולרית ועדיין	4.00	3.63	2.86
cholestrol	4.57	4.38	2.86
	4.57	3.00	4.86
	2.86	4.13	3.14
hypertension, BPH	3.38	3.50	3.00
0	3.57	3.75	4.14
	3.43	4.75	3.86
0	2.86	4.75	3.29
0	2.43	3.00	4.00
בקע סרעפתי	3.43	3.25	4.29
polio	3.29	4.14	2.86
	3.14	4.00	3.17
	2.00	4.13	3.86
	3.57	3.13	2.86
0	2.38	4.00	3.86
0	3.57	4.88	2.86
	3.86	3.25	3.67
0	3.71	4.00	3.57
	3.71	4.13	3.86
	3.14	4.13	3.71
	4.14	4.25	2.57
	3.71	3.38	3.57
	3.57	4.88	4.29
	2.43	4.50	4.86
Coeliac disease	3.00	4.38	4.57
Melanoma	3.57	2.88	4.00
	3.43	4.38	5.00
	3.86	4.38	2.86
0	4.29	4.75	4.00
3 catheterizations	3.86	4.13	4.29
	#NULL!	#NULL!	#NULL!

hypertension, Joint ossification, Osteoporosis, Th	2.29	3.63	3.00
0	3.57	4.38	3.86
טרשת נפוצה.	3.00	4.38	4.14
diabetes	3.57	3.88	4.71
	3.33	3.63	4.57
	4.00	3.88	4.71
0	2.43	3.75	4.57
0	3.57	4.38	3.71
Diabetes	3.00	4.13	2.86
0	3.00	3.00	3.71
0	3.86	4.25	3.43
0	3.57	4.00	4.57
0	3.29	4.38	4.43
	3.14	3.75	3.86
0	2.57	4.00	3.43
	3.00	3.75	2.86
0	2.57	4.75	5.00
0	2.43	4.00	5.00
Asthma	3.57	4.13	2.86
cholesterol	2.43	4.75	3.71
נאמר לו שיתכן ויש לו טרשת נפוצה	3.71	4.50	3.57
0	3.29	4.25	3.00
	3.86	3.63	4.86
Diabetes, cholesterol, hypertension	2.43	4.00	3.14
999	2.00	3.50	3.57
Cholesterol	3.57	3.88	4.29
	3.14	4.00	3.57
0	3.43	4.71	4.50
high blood pressure	2.71	4.13	4.29
0	2.57	4.88	4.71
0	4.00	4.63	3.86
Chronic intestinal disease	2.71	4.88	3.71
0	4.14	4.50	2.86
	3.14	4.00	3.29
	3.71	3.88	4.86
0	3.86	4.13	4.29
hypertension	3.00	3.75	3.43
0	4.00	3.13	3.71
Depression	2.14	4.13	3.57
0	2.43	3.50	4.14
0	3.43	3.00	3.00
	3.14	4.25	4.00
0	2.14	4.00	4.86
Aulcos, Hypertension, Astma	3.86	3.50	3.57
Diabetes,Under activity of the thyroid gland	3.00	3.25	3.71
	3.00	3.63	3.57
Breast cance 10 years ago	2.86	3.25	4.71
0	3.00	4.38	2.71
0	3.71	3.38	3.43
0	3.00	3.75	2.86
	4.00	3.00	4.00
	3.71	5.00	5.00
	3.14	4.63	4.57
0	4.00	3.63	3.14
0	#NULL!	#NULL!	#NULL!
Cholersterol)	2.57	3.38	2.71

0	3.71	2.86	4.57
Cholesterol	2.86	3.50	4.29
	3.14	4.00	3.50
	2.00	3.63	3.43
0	3.29	2.75	3.86
	2.50	4.00	3.83
0	2.86	3.50	3.57
0	4.13	4.25	2.86
	#NULL!	#NULL!	#NULL!
0	2.43	3.13	4.71
	3.14	4.25	4.29
0	3.86	3.88	3.86
0	2.43	4.25	4.57
Diabetes, hypertension, cholesterol	3.88	2.75	3.57
Thyroid Cancer	3.14	4.50	4.00
	2.29	3.75	3.33
0	4.43	4.13	2.57
	3.71	4.50	4.14
	2.86	4.25	3.71
0	3.75	3.50	3.00
0	3.14	3.88	4.14
0	3.14	2.88	3.71
	2.29	4.00	3.57
Asthma	2.29	2.88	2.14
0	2.71	4.88	4.29
0	3.00	2.38	3.43
	4.29	4.00	4.33
	3.71	4.00	3.86
	2.86	2.75	4.71
	3.57	2.38	4.29
0	3.57	3.25	3.29
0	4.43	4.88	4.86
hypertantion, Diabetes,Liver problems,Osteoarthri	2.57	4.25	3.00
	2.43	3.50	3.86
0	2.86	4.13	3.71
0	2.43	4.63	4.57
	2.71	3.63	4.43
	3.00	3.63	4.86
	2.33	4.13	4.43
	3.14	3.25	3.71
	3.14	4.75	2.86
0	2.86	3.63	3.57
0	2.29	3.63	3.57
	3.71	3.00	3.71
0	3.57	4.13	4.71
minor diabetes	2.43	4.25	4.29
0	3.57	3.63	3.14
0	2.63	3.75	3.00
	3.43	3.50	3.43
0	3.71	4.88	4.43
Hypertension	3.29	4.63	3.67
	2.67	3.14	3.29
2006-2009 breast cancer	3.43	4.13	3.29
0	3.14	4.38	4.29
אלרגיה. נחנק במקומות שמעוררים זאת. לא מטופל תרופת	3.00	3.88	3.86
Under activity of the thyroid gland	4.00	3.50	2.86

	3.29	4.00	3.14
0	4.29	3.00	3.71
Diabetes, Hypertension, Cholesterol, Triglycerides	3.86	4.63	4.43
hypertension, Hiatal hernia, reflux	3.00	3.88	3.00
	3.86	3.13	4.00
Cholesterol	3.57	2.88	4.14
	2.71	3.75	4.43
Thyoid gland	4.71	3.88	3.57
0	1.86	3.13	4.86
0	3.86	4.00	4.33
0	2.14	4.63	3.71
Leukemia	2.29	4.75	4.14
0	3.86	4.50	3.57
	4.43	4.25	3.43
	2.57	3.63	2.29
	1.14	3.63	1.86
	3.29	3.50	3.57
0	2.71	3.63	5.00
	2.57	4.63	5.00
	4.43	3.25	4.00
0	3.71	4.63	4.43
	4.29	4.00	4.29
0	3.43	4.25	5.00
	3.57	4.13	3.43
	3.14	4.38	4.14
	4.00	4.63	4.00
	3.71	4.38	3.00
hypertention, high colesterole,Hyperglycemia	3.43	4.25	3.71
0	3.57	3.75	4.00
Colitis, Hypertension, Cholesterol	3.00	3.63	4.29
0	3.71	3.63	4.57
	3.71	3.25	3.43
eye problems	2.14	2.88	3.71
	4.14	5.00	4.86
0	2.57	4.13	3.00
0	2.71	3.88	3.00
	3.14	4.50	3.43
0	3.29	4.14	4.00
0	1.25	3.38	4.14
שחיקה במפרק הירך	2.43	4.25	2.57
	#NULL!	#NULL!	#NULL!
Osteoporosis	3.00	4.00	3.29
	1.86	3.88	3.00
Sinusitis	2.71	4.38	3.86
Cholesterol	3.71	2.88	3.43
	4.00	3.00	5.00
	4.43	5.00	3.57
0	3.50	4.43	4.43
	3.71	4.13	4.43
Hypertension	2.86	3.88	3.57
	3.25	3.50	4.14
	3.71	3.75	4.00
	3.29	4.13	4.00
hypertention, Diabetes mellitus	3.29	3.88	3.14
	4.00	4.00	4.71
	3.43	3.25	4.29

Hypertension, Hypothyroidism,Liver dysfunction	3.14	3.88	3.86
	3.86	3.88	3.43
0	2.29	3.88	3.43
	3.43	3.75	4.29
diabetes, hyperlipidemia	2.57	3.25	3.86
Osteoporosis, Bloos pressure, cholestrol	2.86	3.38	5.00
בעיה בשסתום המיטרלי	3.86	4.63	4.86
	3.14	4.63	5.00
0	3.14	3.75	3.14
	2.86	3.38	3.29
0	4.14	3.13	4.57
	3.14	3.25	4.57
	#NULL!	#NULL!	#NULL!
	2.14	4.25	4.14
0	2.86	3.25	3.43
hypertension, Prostate dysfunction, sleeping probl	2.71	3.38	4.67
0	3.43	4.13	3.71
Gout	2.71	3.75	4.14
	3.71	3.88	4.43
	3.29	4.25	4.29
0	4.86	4.75	3.00
0	3.71	4.50	4.86
colesterol. osteoporosis	3.86	3.25	3.00
	3.57	3.63	4.17
Hypothyroidism	2.86	4.38	3.43
Cholesterol	1.71	3.88	4.29
Cholesterol	2.57	3.13	4.29
Cholesterol, hypertension	3.57	4.38	4.43
בעיה בפרקי ירכיים	2.71	4.13	4.14
colesterol, hypertension, Joint problem	3.86	3.63	2.43
colesterol	2.14	3.25	4.00
	3.71	2.25	3.86
colesterol	4.00	3.75	4.29
	2.20	5.00	4.80
0	3.14	3.50	4.57
	#NULL!	#NULL!	#NULL!
	2.43	4.00	4.14
0	4.43	3.63	3.86
0	3.43	4.50	3.86
0	3.43	4.13	3.14
	3.14	4.13	4.57
0	1.88	4.38	2.71
	3.57	3.75	4.29
Osteoporosis	3.29	3.88	3.71
hypertention	3.57	4.00	4.29
	#NULL!	#NULL!	#NULL!
	2.71	3.75	4.00
0	2.57	4.38	3.71
0	2.43	4.38	3.57
hypertention,Thyroid Problem	3.43	3.63	4.71
hypertention	3.88	4.38	2.43
Osteoporosis,Cuvier colitis	4.00	3.63	2.86
	2.57	3.63	3.14
0	3.29	3.88	2.86
schizophrenia	2.00	3.63	2.86
0	2.57	4.88	4.86

0	3.86	3.13	5.00
Diabetes, Hypertention	3.57	4.38	3.83
Cholesterol	3.43	4.13	4.71
	3.57	3.88	4.43
	#NULL!	#NULL!	#NULL!
	4.14	4.00	3.43
Cardiac catheterization	4.14	3.38	4.33
	3.43	4.00	2.71
	3.57	3.75	3.29
	3.57	3.88	4.57
0	2.86	3.38	4.71
hypertention	3.71	4.13	4.86
0	3.71	4.25	4.86
	3.67	3.71	3.33
	#NULL!	#NULL!	#NULL!
	#NULL!	#NULL!	#NULL!
	3.00	2.71	2.71
	4.14	4.00	3.00

neuroticism_P3	openess_P3	ds_soc_P3	ds_neg_P3	typed_P3	Positivity_P3	Tas_identify_P3
4.50	1.78	20.00	10.00	1.00	3.75	0.00
4.88	4.44	1.00	17.00	0.00	4.25	2.43
2.75	4.00	13.00	8.00	0.00	3.88	1.00
3.00	4.22	8.00	6.00	0.00	4.13	1.10
1.63	2.78	10.00	7.00	0.00	4.63	#NULL!
1.50	3.67	3.00	0.00	0.00	5.00	0.00
1.50	3.56	2.00	7.00	0.00	3.75	0.00
1.75	3.67	7.00	5.00	0.00	4.13	0.29
2.38	4.33	6.00	10.00	0.00	3.88	0.86
1.00	4.00	4.00	1.00	0.00	4.88	1.65
2.63	3.50	11.00	4.00	0.00	3.63	0.43
2.63	3.67	8.00	5.00	0.00	3.25	1.00
2.00	4.00	4.00	5.00	0.00	4.50	0.43
2.13	3.78	0.00	4.00	0.00	4.00	1.65
2.25	3.67	16.00	13.00	1.00	3.88	1.00
2.50	3.22	3.00	4.00	0.00	3.75	#NULL!
2.25	4.33	9.00	3.00	0.00	4.00	1.14
3.38	2.33	20.00	0.00	0.00	3.00	0.00
3.00	4.00	15.00	16.00	1.00	3.00	2.00
2.00	3.78	25.00	6.00	0.00	4.13	1.57
1.63	3.22	2.00	3.00	0.00	4.25	0.29
1.63	4.56	3.00	0.00	0.00	3.29	0.29
1.50	3.67	6.00	0.00	0.00	3.88	0.14
2.63	3.56	12.00	9.00	0.00	3.63	1.57
2.63	3.56	8.00	12.00	0.00	3.63	1.29
3.13	4.00	1.00	4.00	0.00	3.38	0.86
3.38	4.11	0.00	20.00	0.00	3.63	1.29
1.63	4.56	11.00	6.00	0.00	4.00	0.57
3.00	4.11	7.00	9.00	0.00	4.00	0.43
2.75	3.89	6.00	9.00	0.00	3.75	0.00
2.25	3.56	9.00	8.00	0.00	3.50	0.86
2.13	3.44	5.00	2.00	0.00	3.63	0.14
3.50	3.25	14.00	15.00	1.00	2.63	0.71
1.88	2.11	13.00	7.00	0.00	3.88	1.00
2.75	4.33	7.00	6.00	0.00	3.63	#NULL!
1.88	4.33	9.00	2.00	0.00	4.25	#NULL!
3.13	1.56	21.00	19.00	1.00	2.75	#NULL!
2.25	4.00	9.00	6.00	0.00	3.38	#NULL!
2.50	3.33	9.00	3.00	0.00	3.25	0.29
1.38	4.67	7.00	9.00	0.00	3.88	0.71
2.25	4.29	0.00	4.00	0.00	3.29	2.00
2.25	4.22	4.00	6.00	0.00	3.88	0.71
3.75	3.22	4.00	12.00	0.00	7.13	1.43
2.50	2.78	0.00	10.00	0.00	3.75	#NULL!
3.13	4.89	0.00	6.00	0.00	4.00	1.85
1.63	3.11	1.00	3.00	0.00	4.25	0.29
2.00	4.22	3.00	2.00	0.00	4.50	0.00
2.38	3.67	14.00	14.00	1.00	4.00	#NULL!
3.25	4.11	14.00	16.00	1.00	4.75	0.14
1.50	4.44	3.00	4.00	0.00	4.75	0.00
2.00	4.44	6.00	5.00	0.00	4.13	0.71
2.00	3.11	1.00	10.00	0.00	3.38	#NULL!
2.50	3.89	0.00	7.00	0.00	3.38	0.86
2.63	4.78	7.00	11.00	0.00	3.88	1.14
#NULL!	#NULL!	12.00	13.00	1.00	#NULL!	#NULL!

2.88	3.56	17.00	4.00	0.00	3.88	0.43
2.38	4.00	7.00	4.00	0.00	4.00	0.43
1.63	4.22	0.00	3.00	0.00	4.00	0.00
3.50	3.44	10.00	14.00	1.00	3.88	0.00
3.13	4.56	5.00	7.00	0.00	4.38	0.71
2.63	3.78	2.00	9.00	0.00	3.88	#NULL!
1.13	3.89	24.00	1.00	0.00	3.75	0.00
2.13	4.00	0.00	6.00	0.00	3.75	0.14
2.00	4.44	9.00	17.00	0.00	3.50	2.14
4.00	3.11	16.00	14.00	1.00	3.00	1.71
2.13	3.22	0.00	4.00	0.00	4.13	0.14
4.00	3.22	6.00	16.00	0.00	3.88	0.43
4.00	2.33	1.00	8.00	0.00	3.25	0.14
2.13	3.22	17.00	7.00	0.00	3.50	1.29
3.25	3.44	22.00	16.00	1.00	3.63	1.14
2.88	3.44	10.00	4.00	0.00	3.38	0.71
1.50	3.67	15.00	3.00	0.00	4.25	0.43
1.13	4.33	13.00	2.00	0.00	3.88	1.00
3.13	3.78	6.00	16.00	0.00	4.00	1.00
1.88	2.78	12.00	7.00	0.00	3.88	0.71
2.13	2.89	10.00	2.00	0.00	4.50	0.00
1.63	4.22	3.00	5.00	0.00	3.75	0.57
2.88	4.11	13.00	13.00	1.00	4.00	#NULL!
1.75	3.75	28.00	8.00	0.00	2.88	0.43
3.50	2.56	21.00	10.00	1.00	3.13	0.57
2.25	4.11	6.00	5.00	0.00	4.25	1.29
2.75	3.67	13.00	7.00	0.00	3.88	0.86
1.38	4.75	5.00	1.00	0.00	4.38	0.57
3.00	3.78	15.00	10.00	1.00	3.63	1.00
1.63	4.56	20.00	3.00	0.00	4.38	1.14
1.00	3.78	0.00	1.00	0.00	4.63	0.00
2.63	3.78	20.00	8.00	0.00	3.38	0.71
5.00	3.89	2.00	19.00	0.00	2.63	0.00
2.38	4.11	5.00	3.00	0.00	3.88	1.43
1.75	3.89	0.00	2.00	0.00	4.88	0.14
2.13	4.67	3.00	3.00	0.00	4.50	1.30
2.63	3.89	9.00	13.00	0.00	3.13	1.00
3.38	3.56	7.00	16.00	0.00	3.13	2.30
4.75	2.44	25.00	25.00	1.00	1.50	#NULL!
2.25	2.67	13.00	0.00	0.00	3.63	1.00
3.25	4.00	5.00	9.00	0.00	3.63	1.29
1.38	3.11	6.00	5.00	0.00	3.63	1.00
2.25	4.00	26.00	6.00	0.00	4.38	0.43
3.38	4.00	2.00	8.00	0.00	#NULL!	1.00
2.50	3.11	14.00	8.00	0.00	3.25	1.86
3.63	3.78	20.00	16.00	1.00	3.50	0.71
2.75	3.78	17.00	12.00	1.00	3.38	1.43
2.25	3.56	11.00	12.00	1.00	3.50	1.29
2.75	3.67	0.00	1.00	0.00	4.13	1.30
2.88	2.89	9.00	10.00	0.00	3.75	1.14
1.75	3.89	9.00	0.00	0.00	3.63	#NULL!
1.00	4.11	4.00	1.00	0.00	4.13	0.00
2.88	3.56	3.00	8.00	0.00	3.38	1.29
2.88	4.44	12.00	11.00	1.00	3.75	1.14
#NULL!	#NULL!	#NULL!	#NULL!	#NULL!	#NULL!	#NULL!
2.50	3.11	16.00	10.00	1.00	3.13	0.57

2.75	4.11	4.00	20.00	0.00	4.57	0.71
1.38	3.67	9.00	3.00	0.00	3.63	1.14
3.38	3.33	14.00	15.00	1.00	3.63	0.14
2.25	3.33	21.00	9.00	0.00	3.38	#NULL!
2.50	3.33	4.00	2.00	0.00	3.63	1.00
2.00	3.44	11.00	3.00	0.00	3.75	0.43
2.88	3.44	12.00	7.00	0.00	4.00	1.14
2.13	4.44	1.00	5.00	0.00	4.75	0.14
#NULL!	#NULL!	7.00	3.00	0.00	#NULL!	#NULL!
2.50	4.00	19.00	17.00	1.00	3.63	1.29
1.63	4.44	7.00	4.00	0.00	4.00	0.00
3.88	3.78	9.00	10.00	0.00	3.75	0.00
2.00	3.11	8.00	4.00	0.00	4.00	0.71
2.75	3.89	0.00	3.00	0.00	3.50	1.00
2.38	4.38	8.00	3.00	0.00	4.00	0.00
3.50	3.67	18.00	18.00	1.00	3.50	#NULL!
2.00	4.11	0.00	7.00	0.00	4.13	0.43
2.00	3.56	0.00	5.00	0.00	3.75	#NULL!
2.00	4.11	12.00	4.00	0.00	4.88	0.14
3.50	3.78	10.00	12.00	1.00	3.50	1.71
2.13	4.11	10.00	12.00	1.00	3.50	#NULL!
2.75	3.56	14.00	10.00	1.00	3.50	0.14
4.00	4.11	20.00	16.00	1.00	2.88	#NULL!
4.25	2.89	7.00	5.00	0.00	4.00	1.57
2.75	3.78	20.00	2.00	0.00	4.43	1.57
3.88	3.78	21.00	18.00	1.00	2.75	1.14
1.71	4.67	4.00	9.00	0.00	4.63	1.43
3.00	4.11	6.00	6.00	0.00	3.88	0.57
1.13	3.89	11.00	0.00	0.00	4.50	#NULL!
3.75	3.78	13.00	23.00	1.00	1.75	#NULL!
3.13	3.56	8.00	14.00	0.00	2.71	#NULL!
2.25	3.22	2.00	4.00	0.00	5.00	0.57
2.50	3.50	7.00	13.00	0.00	3.88	1.83
2.25	3.22	16.00	11.00	1.00	3.50	1.00
2.38	2.89	10.00	15.00	1.00	3.50	1.71
2.00	4.11	18.00	1.00	0.00	4.38	0.00
2.63	3.00	4.00	5.00	0.00	4.50	#NULL!
2.88	3.44	11.00	12.00	1.00	3.75	0.71
3.00	3.22	20.00	8.00	0.00	3.13	1.29
2.50	3.33	10.00	11.00	1.00	3.88	#NULL!
2.50	3.44	3.00	8.00	0.00	4.00	2.57
4.13	3.78	15.00	22.00	1.00	2.88	1.71
3.38	4.11	13.00	18.00	1.00	3.00	1.86
3.38	3.11	6.00	8.00	0.00	3.50	1.14
2.25	4.56	4.00	4.00	0.00	4.00	0.86
3.13	3.11	7.00	7.00	0.00	3.50	0.00
4.38	4.33	9.00	22.00	0.00	2.13	2.05
2.38	3.78	10.00	7.00	0.00	3.38	0.86
2.75	3.44	8.00	10.00	0.00	3.63	0.71
1.25	4.56	0.00	6.00	0.00	4.88	1.29
1.25	3.67	4.00	3.00	0.00	4.50	0.29
3.25	3.00	10.00	8.00	0.00	3.00	#NULL!
2.38	4.11	1.00	7.00	0.00	3.88	0.43
2.00	3.89	10.00	4.00	0.00	4.00	0.57
3.13	4.22	7.00	14.00	0.00	3.38	1.43
4.25	4.78	9.00	13.00	0.00	3.13	1.57

3.13	4.00	7.00	14.00	0.00	3.38	1.65
2.63	4.67	0.00	9.00	0.00	4.38	0.00
1.50	4.44	0.00	2.00	0.00	4.75	0.71
3.88	4.75	18.00	19.00	1.00	3.25	0.57
4.13	3.78	7.00	23.00	0.00	2.25	#NULL!
2.00	4.89	7.00	4.00	0.00	3.75	0.14
2.50	4.00	7.00	5.00	0.00	3.50	1.29
2.38	4.67	1.00	12.00	0.00	3.63	0.00
2.75	2.44	16.00	13.00	1.00	3.00	1.43
3.38	2.78	3.00	17.00	0.00	3.50	2.43
1.88	2.11	20.00	7.00	0.00	3.88	2.14
2.75	2.78	15.00	10.00	1.00	2.25	0.00
4.13	3.67	6.00	9.00	0.00	4.38	1.14
4.00	4.11	7.00	12.00	0.00	3.63	2.00
2.75	2.89	19.00	18.00	1.00	3.38	1.71
1.63	2.78	27.00	8.00	0.00	3.75	#NULL!
2.63	3.67	14.00	15.00	1.00	3.25	1.43
3.25	4.44	9.00	15.00	0.00	3.88	0.57
2.63	3.89	13.00	8.00	0.00	2.88	1.86
1.75	5.00	0.00	4.00	0.00	3.75	#NULL!
1.75	4.78	2.00	5.00	0.00	4.00	0.29
1.38	4.11	5.00	11.00	0.00	4.00	#NULL!
1.13	4.44	0.00	1.00	0.00	4.00	0.43
2.38	3.56	7.00	8.00	0.00	4.00	0.57
1.38	3.56	1.00	6.00	0.00	4.38	0.57
2.13	4.78	5.00	5.00	0.00	4.38	0.71
2.38	2.89	7.00	8.00	0.00	3.50	2.14
2.13	3.00	0.00	2.00	0.00	4.00	0.00
3.00	3.44	10.00	7.00	0.00	3.63	1.43
2.25	4.22	6.00	16.00	0.00	3.63	1.43
2.63	4.22	3.00	13.00	0.00	4.00	1.14
3.75	4.33	16.00	24.00	1.00	3.75	#NULL!
2.38	3.22	22.00	22.00	1.00	2.38	2.43
1.38	4.44	2.00	3.00	0.00	4.75	#NULL!
3.25	4.11	11.00	16.00	1.00	2.88	1.57
3.75	4.44	12.00	9.00	0.00	3.63	1.14
2.88	4.22	12.00	3.00	0.00	3.63	#NULL!
3.00	3.33	15.00	18.00	1.00	3.00	2.00
3.25	3.00	28.00	18.00	1.00	2.50	2.29
3.00	4.11	8.00	11.00	0.00	3.00	0.57
#NULL!	#NULL!	8.00	8.00	0.00	#NULL!	#NULL!
2.38	3.11	5.00	3.00	0.00	3.63	1.29
2.75	2.33	23.00	14.00	1.00	2.88	0.29
1.25	4.11	13.00	5.00	0.00	4.86	1.05
2.25	4.67	6.00	7.00	0.00	3.13	#NULL!
3.00	#NULL!	11.00	16.00	1.00	3.38	#NULL!
2.00	4.89	2.00	3.00	0.00	4.38	0.00
3.38	2.38	7.00	2.00	0.00	3.13	0.29
1.38	4.22	1.00	5.00	0.00	3.75	0.86
3.50	4.33	9.00	20.00	0.00	3.38	#NULL!
2.88	3.67	3.00	13.00	0.00	4.13	0.57
3.00	3.78	4.00	15.00	0.00	3.75	1.14
2.13	3.33	6.00	10.00	0.00	4.13	1.14
2.86	3.78	8.00	12.00	0.00	2.14	1.71
2.13	3.67	4.00	2.00	0.00	4.50	#NULL!
1.75	3.56	6.00	7.00	0.00	3.13	1.00

3.38	3.50	15.00	16.00	1.00	3.50	1.14
3.00	3.78	1.00	10.00	0.00	3.75	1.43
2.63	3.33	17.00	8.00	0.00	3.00	0.86
3.00	3.89	9.00	11.00	0.00	3.71	2.57
3.38	2.56	16.00	19.00	1.00	3.25	1.29
3.13	4.78	16.00	9.00	0.00	3.75	0.57
1.00	3.78	4.00	2.00	0.00	4.63	0.57
1.88	3.89	8.00	2.00	0.00	3.88	#NULL!
2.63	4.33	5.00	9.00	0.00	3.50	1.29
3.25	3.33	7.00	9.00	0.00	3.50	1.86
3.63	3.89	0.00	12.00	0.00	3.63	0.43
2.50	2.89	16.00	3.00	0.00	3.63	#NULL!
#NULL!	#NULL!	13.00	5.00	0.00	#NULL!	#NULL!
4.13	4.00	20.00	22.00	1.00	3.00	1.57
2.75	3.00	13.00	13.00	1.00	3.63	1.57
2.29	4.11	14.00	11.00	1.00	4.13	1.00
3.88	4.89	6.00	23.00	0.00	3.50	1.29
1.63	3.89	12.00	6.00	0.00	3.63	0.71
3.38	3.78	10.00	12.00	1.00	3.25	1.50
1.00	3.56	2.00	3.00	0.00	3.88	0.33
3.00	3.33	0.00	5.00	0.00	4.75	0.71
1.50	4.22	2.00	3.00	0.00	4.50	1.29
3.13	3.89	6.00	3.00	0.00	3.50	1.45
2.25	3.50	4.00	11.00	0.00	3.14	2.71
2.75	4.00	8.00	8.00	0.00	3.38	1.71
3.13	2.78	28.00	21.00	1.00	1.75	1.86
2.00	3.89	21.00	4.00	0.00	3.75	1.29
2.50	3.11	3.00	4.00	0.00	4.88	0.43
3.38	3.44	22.00	7.00	0.00	3.50	0.00
3.00	3.56	3.00	5.00	0.00	3.75	1.50
4.00	2.67	24.00	15.00	1.00	3.00	1.86
3.29	4.00	1.00	16.00	0.00	3.75	1.57
4.00	4.11	10.00	20.00	1.00	3.75	1.70
2.60	2.25	0.00	3.00	0.00	4.00	0.57
2.00	4.44	6.00	5.00	0.00	4.25	0.00
#NULL!	#NULL!	5.00	13.00	0.00	#NULL!	#NULL!
1.38	3.44	16.00	4.00	0.00	3.75	0.43
1.75	4.67	1.00	3.00	0.00	4.13	0.43
2.63	3.89	6.00	9.00	0.00	3.88	0.71
2.13	4.00	13.00	4.00	0.00	3.75	0.57
1.63	4.67	9.00	3.00	0.00	4.13	1.00
2.13	3.00	23.00	11.00	1.00	3.13	1.29
3.00	3.00	1.00	7.00	0.00	3.00	1.57
1.88	3.67	6.00	4.00	0.00	4.00	#NULL!
2.63	3.89	10.00	11.00	1.00	3.88	0.57
#NULL!	#NULL!	10.00	7.00	0.00	#NULL!	#NULL!
2.63	2.89	13.00	8.00	0.00	3.38	1.43
1.75	4.11	7.00	3.00	0.00	3.75	1.57
2.75	3.67	17.00	8.00	0.00	4.00	0.71
3.13	4.67	9.00	10.00	0.00	3.75	0.57
3.00	4.78	18.00	7.00	0.00	3.88	1.29
4.75	4.56	12.00	22.00	1.00	3.38	1.43
2.50	3.44	13.00	7.00	0.00	3.38	1.29
3.63	3.00	10.00	11.00	1.00	3.38	#NULL!
4.13	3.78	18.00	17.00	1.00	1.88	3.00
1.75	3.44	15.00	2.00	0.00	4.25	0.71

2.50	4.78	3.00	3.00	0.00	4.00	0.43
2.25	4.33	2.00	9.00	0.00	3.88	1.14
1.88	4.22	4.00	3.00	0.00	3.88	0.00
2.13	3.56	2.00	4.00	0.00	3.88	0.14
#NULL!	#NULL!	15.00	9.00	0.00	3.88	#NULL!
2.13	3.89	2.00	6.00	0.00	3.88	#NULL!
1.75	4.00	0.00	4.00	0.00	5.00	0.00
4.50	3.56	6.00	22.00	0.00	2.75	1.71
4.75	4.56	3.00	24.00	0.00	2.50	1.14
1.25	2.56	14.00	14.00	1.00	3.75	0.00
3.25	2.78	13.00	12.00	1.00	3.88	0.29
2.57	3.78	4.00	0.00	0.00	4.00	1.00
1.00	4.33	4.00	3.00	0.00	4.13	0.71
2.29	4.44	7.00	7.00	0.00	4.00	2.00
#NULL!	#NULL!	#NULL!	#NULL!	#NULL!	#NULL!	0.14
#NULL!	#NULL!	#NULL!	#NULL!	#NULL!	#NULL!	1.00
3.00	2.78	21.00	28.00	1.00	3.63	3.50
3.13	3.67	1.00	8.00	0.00	3.88	1.65

Tas_describe_P3	Tas_external_P3	Tas_total_P3	NS_P3	HA_P3	RD_P3	PS_P3
1.80	1.63	1.10	55.00	42.00	58.00	65.00
1.25	0.13	1.21	73.68	76.00	84.00	66.32
1.00	1.50	1.20	49.00	70.00	51.00	64.00
0.43	0.40	2.13	41.00	62.00	66.00	67.00
#NULL!	#NULL!	#NULL!	36.00	45.00	45.26	65.00
0.00	1.86	0.68	46.00	34.00	79.00	85.00
1.40	1.88	1.10	56.00	51.00	68.00	66.00
0.80	1.50	0.90	50.00	42.00	64.00	95.00
0.80	2.13	1.35	62.00	51.00	76.00	69.00
1.00	2.00	2.00	57.00	48.00	65.00	67.00
0.60	0.75	0.60	52.00	51.00	70.53	58.00
1.00	1.38	1.15	64.00	55.00	69.00	61.05
0.60	0.75	0.60	55.00	51.00	80.00	67.00
0.86	1.20	2.63	56.00	34.00	56.00	75.00
1.40	1.75	1.40	49.00	55.00	54.00	67.00
#NULL!	#NULL!	#NULL!	46.00	48.00	75.00	66.00
1.00	1.38	1.20	59.00	44.00	66.00	68.00
2.20	2.00	1.35	48.00	69.00	60.00	41.00
1.00	2.67	1.94	50.00	54.00	57.00	60.00
3.40	2.38	2.35	50.00	54.00	56.00	70.00
0.80	1.50	0.90	56.00	31.00	64.00	66.00
0.20	0.25	0.25	55.00	35.79	55.00	68.00
1.60	1.50	1.05	46.00	43.00	60.00	73.00
1.80	2.25	1.90	66.00	49.00	56.00	61.00
0.80	1.25	1.15	67.00	55.00	67.00	61.00
1.20	0.75	0.90	63.00	56.00	79.00	49.00
0.80	1.13	1.10	50.00	56.00	79.00	95.00
0.60	2.13	1.20	79.00	55.00	74.00	71.00
1.40	1.63	1.15	60.00	41.00	61.00	73.00
1.60	1.63	1.05	51.00	47.00	59.00	62.00
0.80	1.75	1.20	60.00	56.00	72.63	56.00
1.20	1.63	1.00	57.00	49.00	67.00	60.00
1.40	1.38	1.15	61.00	61.00	59.00	51.00
1.80	2.75	1.90	35.00	43.00	50.00	72.00
#NULL!	#NULL!	#NULL!	49.00	47.00	81.00	68.00
#NULL!	#NULL!	#NULL!	58.00	44.00	62.00	73.00
#NULL!	#NULL!	#NULL!	43.00	87.00	75.00	38.00
#NULL!	#NULL!	#NULL!	55.00	52.63	70.53	73.33
1.40	1.13	0.90	46.00	54.00	71.00	68.00
1.40	0.88	0.95	60.00	46.00	69.00	55.00
1.80	1.88	1.90	53.00	50.00	73.33	63.00
1.00	1.00	0.90	59.00	57.00	70.00	62.00
0.20	0.88	0.90	61.00	53.00	80.00	62.00
#NULL!	#NULL!	#NULL!	54.00	50.00	82.00	63.00
2.00	1.20	2.13	69.00	46.00	77.00	67.00
0.40	1.75	0.90	50.00	35.00	71.58	81.00
0.20	0.25	0.15	55.00	51.00	71.00	64.00
#NULL!	#NULL!	#NULL!	50.00	59.00	65.26	69.00
0.40	0.88	0.50	48.00	31.00	75.00	94.00
1.00	0.75	0.55	51.00	30.00	49.47	69.00
1.20	0.63	0.80	48.00	37.00	67.00	74.00
#NULL!	#NULL!	#NULL!	70.00	57.89	73.00	51.00
0.80	0.25	0.60	55.00	55.00	80.00	60.00
0.40	0.38	0.65	55.00	53.00	70.00	77.00
#NULL!	#NULL!	#NULL!	55.00	58.00	69.00	63.00

2.00	2.13	1.50	46.00	63.00	66.00	62.00
2.40	1.00	1.15	62.00	46.00	65.26	83.00
0.80	1.00	0.60	59.00	53.00	69.00	60.00
1.40	1.00	0.75	55.00	59.00	74.00	83.00
0.60	1.25	0.90	55.00	54.00	65.00	73.00
#NULL!	#NULL!	#NULL!	55.00	54.00	71.00	72.00
1.80	2.25	1.35	38.00	54.00	36.00	65.00
0.20	1.13	0.55	56.00	57.00	65.00	69.00
1.60	1.50	1.75	54.00	57.00	61.00	53.00
1.80	2.38	2.00	54.00	72.00	54.00	58.00
0.40	2.50	1.15	67.00	31.00	83.00	69.00
1.20	1.88	1.20	47.00	73.00	72.63	63.16
0.60	0.50	0.40	58.00	66.00	69.00	51.00
2.00	2.00	1.75	53.00	57.00	56.00	61.00
1.60	1.38	1.35	54.00	71.00	53.00	57.00
0.60	0.63	0.65	62.00	61.00	77.00	53.00
1.20	1.88	1.20	60.00	46.00	60.00	75.00
1.00	1.25	1.10	49.00	47.00	55.00	67.00
1.00	2.00	1.44	60.00	57.00	78.00	62.00
1.60	1.63	1.30	54.00	50.53	64.21	61.11
0.00	2.00	0.80	60.00	51.00	74.00	57.00
2.60	1.25	1.35	49.00	51.00	63.00	64.00
#NULL!	#NULL!	#NULL!	59.00	57.00	76.00	83.00
1.60	2.75	1.65	58.00	55.00	50.00	65.26
1.60	0.75	0.90	50.00	72.00	64.00	62.00
0.80	1.75	1.35	55.00	43.00	78.00	58.00
1.60	1.75	1.40	56.00	54.00	56.00	69.00
1.20	0.63	0.75	56.00	39.00	72.00	70.00
1.80	2.25	1.70	55.00	57.00	53.00	71.00
1.20	1.50	1.30	52.00	57.00	64.00	75.00
0.20	1.13	0.50	62.00	31.00	73.00	67.00
0.60	0.50	0.60	50.00	50.00	71.00	60.00
1.60	0.25	0.50	55.00	77.00	88.00	79.00
1.00	1.75	1.45	65.00	48.00	70.00	69.00
0.80	1.63	0.90	47.00	43.00	64.00	74.00
1.00	1.20	1.63	60.00	52.00	73.00	60.00
1.00	1.63	1.25	49.00	59.00	65.00	74.00
2.00	2.40	2.50	54.00	56.00	63.00	77.00
#NULL!	#NULL!	#NULL!	38.00	82.00	43.00	45.00
1.80	2.00	1.60	52.00	58.00	63.00	62.00
1.00	1.75	1.40	66.00	54.00	72.00	69.00
1.60	2.38	1.70	63.16	46.67	54.74	76.25
1.60	1.50	1.15	50.00	52.00	44.00	91.00
1.40	0.50	0.90	58.00	58.00	71.00	71.00
1.60	1.50	1.65	52.00	58.00	57.89	71.00
0.20	1.38	0.85	60.00	52.00	51.00	70.00
1.60	1.63	1.55	47.00	69.00	63.00	71.00
1.40	1.63	1.45	65.00	58.00	70.00	52.00
0.29	1.00	2.38	59.00	38.95	74.00	74.00
2.00	2.13	1.75	51.00	50.00	61.00	51.00
#NULL!	#NULL!	#NULL!	58.00	44.00	49.00	70.00
0.80	1.50	0.80	51.00	28.00	74.00	79.00
1.00	2.88	1.85	56.00	56.00	83.00	78.95
1.60	0.88	1.15	63.00	42.00	64.00	61.00
#NULL!	#NULL!	#NULL!	#NULL!	#NULL!	#NULL!	#NULL!
0.80	1.13	0.85	55.00	61.00	67.00	51.00

1.20	1.50	1.15	54.00	49.00	67.78	72.00
1.20	1.38	1.25	51.00	45.00	56.00	60.00
0.00	1.50	0.65	58.00	66.00	72.00	61.00
#NULL!	#NULL!	#NULL!	42.00	60.00	49.00	58.00
1.00	1.13	1.05	64.00	44.00	67.00	60.00
0.80	0.86	0.68	50.00	53.00	63.16	60.00
3.00	2.00	1.95	57.00	51.00	58.00	75.00
0.00	0.75	0.35	67.00	26.00	74.00	65.00
#NULL!	#NULL!	#NULL!	54.00	53.68	63.00	73.00
2.00	0.75	1.25	56.00	63.00	64.00	62.00
0.60	0.50	0.35	62.00	43.00	73.00	71.00
1.00	1.13	0.70	59.00	54.00	66.00	63.00
1.20	1.50	1.15	52.00	46.00	61.00	74.00
1.00	1.50	1.20	56.00	43.00	69.00	79.00
0.50	1.63	0.83	55.00	54.00	64.00	66.00
#NULL!	#NULL!	#NULL!	54.00	68.00	56.00	69.00
1.00	1.13	0.85	73.00	31.00	78.00	55.00
#NULL!	#NULL!	#NULL!	63.00	46.00	72.00	59.00
0.20	0.38	0.25	50.00	40.00	55.00	63.16
1.20	1.75	1.60	66.00	58.00	78.00	70.00
#NULL!	#NULL!	#NULL!	60.00	45.00	59.00	75.00
1.40	1.75	1.10	57.00	54.00	54.00	55.00
#NULL!	#NULL!	#NULL!	39.00	66.00	58.00	45.00
0.80	2.50	1.75	67.00	33.00	54.00	81.00
1.60	1.50	1.55	47.00	60.00	63.00	75.00
0.80	1.00	1.00	62.00	63.00	44.00	66.00
0.60	1.75	1.35	68.00	48.00	84.00	58.00
0.80	1.25	0.90	58.00	50.00	76.00	72.00
#NULL!	#NULL!	#NULL!	62.00	52.00	53.00	76.00
#NULL!	#NULL!	#NULL!	50.00	72.00	65.00	51.00
#NULL!	#NULL!	#NULL!	58.00	46.32	73.68	63.00
0.80	1.13	0.85	47.00	34.00	78.00	80.00
1.20	1.38	1.47	60.00	66.00	66.00	57.00
2.20	1.63	1.55	48.00	67.00	65.00	58.00
1.80	2.00	1.85	56.00	61.00	71.00	57.00
1.40	0.88	0.70	50.00	48.42	55.00	79.00
#NULL!	#NULL!	#NULL!	44.00	46.00	67.00	77.00
2.20	1.50	1.40	51.00	67.00	65.00	74.00
2.40	2.43	2.00	44.00	64.00	51.58	62.00
#NULL!	#NULL!	#NULL!	43.33	44.62	52.73	70.00
2.00	0.75	1.70	58.00	50.00	73.00	60.00
1.20	0.75	1.20	52.00	67.00	63.00	66.00
1.60	1.00	1.45	68.00	63.00	63.00	68.00
0.80	1.38	1.15	52.00	54.00	75.00	65.00
1.00	0.88	0.90	56.00	47.00	61.00	67.00
0.00	0.50	0.20	45.00	63.00	69.00	64.00
2.14	2.40	1.75	51.00	76.00	74.00	55.00
1.40	0.63	0.90	44.00	42.00	49.00	62.00
1.00	1.00	0.88	57.00	54.00	76.00	69.00
1.40	1.00	1.20	71.00	37.89	67.00	65.00
0.60	2.00	1.05	53.68	39.00	75.00	60.00
#NULL!	#NULL!	#NULL!	50.00	61.05	64.00	51.00
1.00	0.75	0.70	57.00	38.00	76.00	64.00
1.00	1.25	0.95	52.00	43.00	72.00	77.00
1.40	1.00	1.25	49.00	54.00	67.00	72.00
0.80	0.63	1.00	55.00	57.00	76.00	43.00

1.29	1.20	2.25	54.00	61.00	73.00	69.00
0.00	1.13	0.45	61.00	35.00	83.00	82.00
0.00	0.50	0.45	66.00	28.00	76.00	94.00
1.20	1.88	1.25	65.00	66.32	71.00	60.00
#NULL!	#NULL!	#NULL!	56.00	62.00	79.00	76.00
0.00	0.63	0.30	57.00	46.00	59.00	57.00
1.60	2.13	1.70	57.00	48.00	66.00	73.00
0.00	0.00	0.00	64.00	38.00	84.00	73.68
1.20	2.88	1.95	40.00	62.00	45.00	77.00
1.80	1.25	1.80	64.00	46.00	72.00	72.00
1.80	1.88	1.95	45.00	59.00	74.00	65.00
0.40	1.25	0.60	52.00	58.00	78.00	67.00
1.80	1.25	1.35	68.00	54.00	81.00	65.00
1.40	1.50	1.63	69.00	60.00	73.00	77.00
1.60	1.88	1.75	50.00	65.56	56.67	52.00
#NULL!	#NULL!	#NULL!	26.00	67.00	48.00	24.00
1.00	1.63	1.40	55.00	59.00	63.16	56.00
1.00	0.75	0.75	49.00	53.00	67.00	80.00
2.20	1.25	1.70	53.00	41.00	58.00	71.00
#NULL!	#NULL!	#NULL!	61.00	38.00	65.00	70.00
0.80	1.13	0.75	47.00	40.00	72.00	83.00
#NULL!	#NULL!	#NULL!	56.00	45.00	63.00	71.00
1.00	1.75	1.10	52.00	35.00	70.00	91.00
0.80	1.25	0.90	53.00	50.00	74.00	75.00
0.00	1.50	0.80	46.32	40.00	65.00	66.00
0.60	1.00	0.80	60.00	49.00	74.00	69.00
1.60	0.88	1.50	63.00	45.00	73.00	51.00
0.00	1.38	0.55	57.00	46.00	69.00	51.00
1.80	0.75	1.25	53.00	65.00	74.00	63.00
1.60	1.25	1.40	62.00	37.00	65.00	74.00
1.20	1.00	1.10	54.00	41.00	64.00	84.00
#NULL!	#NULL!	#NULL!	56.00	65.00	56.84	70.00
2.20	1.50	2.00	56.00	54.00	46.00	61.00
#NULL!	#NULL!	#NULL!	54.00	40.00	79.00	77.00
2.60	1.50	1.80	63.00	56.00	64.00	53.68
1.20	1.00	1.10	71.00	50.00	62.00	58.00
#NULL!	#NULL!	#NULL!	60.00	58.00	56.00	69.00
1.40	1.38	1.60	73.00	54.00	68.00	68.00
2.80	1.63	2.15	59.00	62.11	53.00	72.00
0.80	1.13	0.85	61.00	59.00	67.00	55.00
#NULL!	#NULL!	#NULL!	54.00	56.00	63.00	66.00
1.20	1.38	1.30	59.00	59.00	69.00	67.00
1.80	2.38	1.50	46.00	61.00	58.00	44.00
0.00	1.80	1.50	48.00	42.00	67.00	84.00
#NULL!	#NULL!	#NULL!	54.00	54.00	71.00	69.00
#NULL!	#NULL!	#NULL!	57.00	67.00	70.00	72.00
0.00	1.38	0.55	68.00	37.00	72.00	76.00
0.80	1.38	0.85	61.00	61.00	68.00	50.00
1.00	0.50	0.75	58.00	41.00	66.00	67.00
#NULL!	#NULL!	#NULL!	60.00	56.00	56.00	65.00
0.80	0.88	0.75	42.00	48.00	74.00	65.00
1.80	1.29	1.37	55.00	56.00	69.47	72.63
0.80	2.13	1.45	53.00	51.00	67.00	71.00
0.80	1.75	1.50	47.37	64.21	61.05	58.00
#NULL!	#NULL!	#NULL!	53.00	45.00	74.00	74.74
1.00	1.50	1.20	58.95	54.00	57.00	65.00

1.40	2.00	1.55	57.00	59.00	67.00	71.00
0.80	1.50	1.30	71.00	50.00	86.00	60.00
2.40	1.38	1.45	59.00	58.00	53.00	61.00
1.80	1.38	1.90	56.00	54.00	73.00	74.00
1.60	1.63	1.50	54.00	60.00	67.00	63.00
1.40	1.38	1.10	47.00	55.00	63.00	83.00
0.20	1.00	0.65	56.00	41.00	69.00	73.00
#NULL!	#NULL!	#NULL!	53.00	44.00	51.00	79.00
1.00	1.13	1.15	61.00	53.00	74.00	52.00
1.40	1.88	1.75	63.00	63.00	57.00	68.42
0.40	0.88	0.60	57.00	42.00	70.00	68.00
#NULL!	#NULL!	#NULL!	46.00	51.00	49.00	66.00
#NULL!	#NULL!	#NULL!	52.00	49.47	63.00	80.00
1.40	1.13	1.35	48.00	87.00	67.00	55.79
1.60	2.00	1.75	55.00	59.00	66.00	65.00
1.40	0.75	1.00	50.00	61.05	51.00	66.00
1.80	1.14	1.37	49.00	71.00	73.00	73.00
1.80	2.25	1.60	60.00	46.00	59.00	75.00
0.75	1.63	1.39	53.00	48.00	72.22	76.00
0.40	0.63	0.47	59.00	30.00	74.00	61.00
0.00	1.13	0.70	61.00	51.00	85.00	64.00
1.20	1.00	1.15	60.00	26.00	72.00	82.00
0.71	0.60	2.63	53.00	62.00	73.00	51.00
1.80	2.00	2.22	65.00	53.68	69.47	71.58
1.80	1.50	1.65	54.00	43.00	64.00	66.00
2.60	3.00	2.50	56.00	71.00	46.00	55.00
2.80	1.88	1.90	49.00	61.00	53.00	68.00
1.20	2.25	1.35	44.00	63.00	66.00	72.00
0.60	0.25	0.25	52.00	63.00	56.00	61.00
1.00	1.20	2.13	59.00	46.00	66.00	70.00
2.00	1.88	1.90	47.00	87.00	76.00	63.00
1.60	1.50	1.55	66.00	42.00	67.00	67.00
0.71	1.60	2.63	58.00	55.00	64.00	80.00
1.60	2.25	1.50	48.00	46.00	73.00	69.00
0.80	1.25	0.70	59.00	36.00	77.00	87.00
#NULL!	#NULL!	#NULL!	64.21	58.82	72.63	52.63
2.20	2.75	1.80	52.63	44.00	53.00	65.00
0.00	1.13	0.60	61.00	39.00	75.79	72.00
0.80	1.13	0.90	50.00	55.00	70.00	70.00
0.80	0.63	0.65	64.21	44.00	67.00	74.00
1.20	1.13	1.10	51.00	45.26	70.00	80.00
2.60	2.50	2.10	46.00	55.00	45.00	66.00
1.80	1.88	1.75	54.12	51.11	64.44	70.00
#NULL!	#NULL!	#NULL!	61.00	36.00	60.00	63.00
1.00	1.00	0.85	49.00	54.00	74.00	63.00
#NULL!	#NULL!	#NULL!	52.00	62.00	60.00	62.00
1.80	2.25	1.85	50.00	60.00	60.00	60.00
1.80	1.50	1.60	55.00	43.00	55.00	73.00
1.00	1.00	0.90	46.00	57.00	72.00	62.00
1.00	0.63	0.70	51.00	50.00	69.00	77.00
1.20	0.38	0.90	67.00	40.00	69.00	61.00
0.60	0.50	0.85	67.00	70.00	77.00	58.00
1.60	1.88	1.60	60.00	53.00	56.00	58.00
#NULL!	#NULL!	#NULL!	49.00	63.00	71.00	47.00
2.00	1.50	2.15	60.00	80.00	65.00	52.00
1.60	1.38	1.20	51.00	52.63	62.00	59.00

0.80	0.63	0.60	60.00	36.00	86.00	88.00
1.20	1.38	1.25	49.00	64.00	79.00	71.00
1.20	0.63	0.55	55.00	34.74	60.00	80.00
0.40	0.38	0.30	52.00	43.00	72.00	76.00
#NULL!	#NULL!	#NULL!	52.00	50.00	68.00	77.00
#NULL!	#NULL!	#NULL!	64.00	42.00	66.00	62.00
0.00	1.50	0.60	48.00	38.00	79.00	75.79
1.00	1.38	1.40	65.00	64.00	74.00	43.00
0.00	0.75	0.70	59.00	77.00	78.00	55.00
0.80	1.38	0.75	58.00	51.00	52.00	62.00
0.80	2.88	1.45	43.00	61.00	71.00	69.00
1.00	1.75	1.30	50.00	50.00	68.00	71.00
0.60	0.75	0.70	61.00	29.00	70.00	81.00
1.80	1.25	1.65	56.00	46.32	65.26	77.89
0.20	0.63	0.35	44.21	45.00	71.00	79.00
1.40	1.50	1.30	37.89	51.00	63.00	66.00
3.57	3.80	3.25	70.53	56.25	54.67	82.11
0.86	1.40	2.50	69.00	40.00	84.00	75.00

SD_P3	CO_P3	ST_P3	avoidant_P3	birthdate	family_status	education
81.00	77.00	23.00	3.67	26-פבר-1949	2	6
57.00	83.00	61.00	1.67	05-מרץ-1947	2	4
74.00	77.00	40.00	3.00	20-ספט-1949	2	6
71.00	76.00	45.00	4.17	#NULL!	2	2
60.00	86.00	25.00	2.33	14-פבר-1949	2	5
98.00	91.00	27.00	4.83	06-דצמ-1962	2	#NULL!
75.00	80.00	33.00	3.00	21-דצמ-1963	2	5
82.11	80.00	42.00	2.33	08-יונ-2034	2	4
84.00	84.00	36.00	1.67	26-יונ-1953	2	5
83.00	86.00	38.40	4.67	#NULL!	4	-1
83.00	80.00	36.57	2.00	01-פבר-1944	2	4
79.00	76.00	43.00	2.00	25-אפר-1943	2	4
69.00	86.00	57.00	2.00	04-פבר-1960	2	5
77.00	77.00	39.00	5.00	#NULL!	2	6
65.00	65.00	44.00	4.00	27-ינו-2030	3	5
81.00	80.00	35.00	2.17	22-יול-1961	2	6
80.00	78.00	54.00	2.17	24-ינו-1942	3	6
92.00	85.00	18.00	3.00	09-ספט-1955	2	2
59.00	64.00	40.00	3.67	31-יול-2025	2	6
72.00	82.00	37.00	3.33	04-יונ-2040	3	2
82.00	76.00	26.00	2.67	13-פבר-1961	2	5
85.00	79.00	43.00	2.33	16-אוג-1944	2	6
89.00	71.58	23.00	2.50	14-פבר-1953	2	5
63.00	66.00	43.00	3.83	05-אפר-1966	2	5
67.00	70.00	19.00	2.83	06-נוב-1948	2	6
85.00	75.00	38.00	1.17	11-אוג-1947	2	5
77.00	67.00	36.00	2.67	07-ספט-1950	2	3
69.00	84.00	54.00	2.67	14-נוב-1961	2	5
74.00	73.00	37.00	3.83	15-יונ-1944	2	#NULL!
76.00	81.00	48.00	3.33	03-אוג-1951	2	6
77.00	78.00	23.00	2.33	22-ספט-1944	2	2
87.00	73.00	23.00	2.50	09-ספט-1955	4	5
53.00	72.00	32.00	3.00	07-אוק-1957	1	3
82.00	89.00	25.00	3.83	25-דצמ-1949	3	5
77.00	78.00	59.00	5.33	#NULL!	#NULL!	#NULL!
87.00	77.00	58.00	5.00	#NULL!	#NULL!	#NULL!
69.00	85.00	35.00	3.50	#NULL!	#NULL!	#NULL!
73.00	81.11	38.00	4.33	#NULL!	#NULL!	#NULL!
87.00	84.00	42.00	4.67	24-אפר-1944	2	#NULL!
68.00	83.00	51.00	2.17	09-אוג-1950	2	4
77.00	60.00	35.00	4.00	13-יונ-2017	2	4
67.00	74.00	45.00	2.17	25-אפר-2032	4	5
72.00	76.00	36.00	4.17	12-פבר-1948	2	4
82.00	79.00	40.00	3.83	26-דצמ-1943	2	4
66.00	83.00	60.80	4.33	#NULL!	2	5
87.00	77.00	36.00	1.50	05-ינו-1966	2	6
80.00	80.00	47.00	1.17	15-ינו-1948	2	5
68.00	77.00	42.00	1.50	22-יונ-1953	1	4
81.00	70.00	40.00	1.17	24-נוב-1954	2	5
90.00	74.00	23.00	3.00	16-נוב-1952	2	3
90.00	88.00	31.00	1.83	09-ינו-1944	2	6
69.00	82.00	23.00	2.50	02-נוב-1946	2	4
79.00	83.00	41.00	1.17	17-יול-1946	2	2
82.00	84.00	67.00	2.17	09-ספט-1955	4	4
71.00	80.00	44.00	#NULL!	31-אוק-1950	1	2

77.00	77.00	36.00	3.50	23-פבר-1943	2	2
87.00	84.00	39.00	2.00	05-מרץ-1949	-1	6
77.00	77.89	35.00	2.17	20-אוק-1963	2	5
67.00	81.00	54.00	2.00	29-יונ-1944	2	2
82.00	78.00	28.00	2.33	05-נוב-1944	4	5
73.68	81.00	57.00	1.67	24-מאי-1945	3	5
81.00	63.00	20.00	4.83	22-ספט-1943	2	3
78.00	79.00	32.00	2.17	03-יול-1947	3	5
59.00	89.00	51.00	3.17	13-ספט-1966	3	3
69.00	55.79	33.00	4.67	31-דצמ-2034	4	3
73.00	69.00	50.00	1.33	24-אפר-2036	2	5
74.00	74.74	38.00	1.00	07-ינו-2037	2	4
78.00	88.00	39.00	2.33	15-ספט-2035	2	6
72.63	71.00	37.00	3.33	09-יול-2035	2	4
59.00	81.00	48.00	3.50	14-ספט-1950	2	4
88.00	92.00	38.00	1.17	25-מאי-1966	2	3
86.00	81.00	45.00	3.00	04-נוב-1952	4	4
81.00	76.00	33.00	2.33	28-אפר-1955	2	4
54.00	77.00	42.00	3.17	10-פבר-1956	2	6
73.00	84.21	47.00	2.50	16-דצמ-1959	2	5
88.00	85.00	29.00	2.00	24-אוק-1957	2	2
80.00	84.00	44.00	2.50	29-מאי-1947	2	3
82.00	85.00	57.00	1.83	06-יול-1965	2	4
55.00	66.00	37.00	5.33	01-נוב-1956	2	5
73.00	74.00	32.00	2.00	08-ינו-1952	2	6
71.00	68.00	22.00	4.50	14-פבר-1951	2	3
63.00	72.00	28.00	2.50	26-יונ-1951	2	6
82.00	84.00	65.00	1.67	08-מרץ-1960	3	3
72.00	74.00	36.00	3.67	21-מאי-2034	2	4
78.00	84.00	59.00	2.83	06-ינו-1961	2	5
89.00	91.00	47.00	1.83	26-פבר-1957	2	2
81.00	89.00	25.00	2.33	05-דצמ-1954	2	4
65.00	86.00	24.00	1.83	04-יונ-1959	1	5
72.00	75.00	50.13	2.33	25-אפר-1953	2	4
83.00	80.00	30.00	2.67	07-אוק-1951	2	7
84.00	82.00	45.00	5.67	#NULL!	2	5
68.00	71.00	30.00	2.50	10-יונ-1955	2	5
57.00	66.00	33.00	4.50	#NULL!	2	6
41.00	68.00	17.00	6.67	13-אוג-1963	2	6
81.00	76.00	34.00	4.33	04-פבר-1942	2	4
61.00	70.00	52.00	2.50	19-פבר-1945	3	4
75.79	72.63	35.43	2.67	09-נוב-1953	2	5
90.00	82.00	30.00	2.67	15-מאי-1953	2	6
80.00	77.00	45.00	2.33	20-פבר-1942	1	5
67.00	66.00	45.00	4.17	04-יול-1958	2	4
73.00	72.00	33.00	3.67	18-נוב-2037	2	5
79.00	72.00	37.00	5.67	21-ספט-1952	2	6
68.00	79.00	37.00	2.67	23-יונ-1960	2	5
83.00	79.00	47.00	5.00	#NULL!	2	4
74.00	74.00	34.00	4.00	02-יונ-1962	2	3
79.00	72.00	29.00	2.67	09-ספט-1955	2	4
81.00	90.00	38.40	2.67	04-יול-1962	3	2
79.00	77.00	31.00	1.33	06-יול-1950	2	3
73.00	78.00	54.00	2.50	24-מאי-1963	2	3
#NULL!	#NULL!	#NULL!	#NULL!	#NULL!	#NULL!	#NULL!
68.00	76.00	43.73	3.67	04-אוק-1948	3	5

79.00	71.00	65.00	2.00	22-יונ-1948	3	4
81.00	71.00	36.00	2.17	09-ספט-1955	2	5
66.00	78.00	24.00	2.50	09-נוב-1964	2	6
70.00	64.00	33.00	3.83	04-אוק-1950	2	7
71.00	57.00	44.00	2.83	21-יול-1949	3	5
75.00	80.00	39.47	1.83	06-יול-2025	2	5
72.00	74.00	41.00	3.67	30-מאי-1948	2	4
93.00	96.00	40.00	5.00	28-ינו-1965	2	#NULL!
82.00	87.00	31.00	#NULL!	25-דצמ-1961	2	3
61.00	82.00	32.00	2.83	21-יול-1965	2	4
84.00	82.00	45.00	1.50	06-יול-1954	2	5
72.00	82.00	43.00	2.17	06-אוג-1951	2	3
70.00	69.00	33.00	3.67	22-פבר-1943	2	5
72.00	70.00	28.00	4.83	13-אפר-1958	2	#NULL!
84.00	83.00	38.00	2.50	17-אוק-1955	2	5
54.00	63.00	45.00	3.50	23-אפר-2036	3	4
62.00	87.00	36.00	1.33	28-אוק-1950	3	5
75.00	83.00	55.00	1.17	17-פבר-1954	2	6
85.00	78.00	44.00	2.33	09-ספט-1955	2	5
70.00	65.26	44.00	3.83	22-אוק-1946	2	#NULL!
71.00	77.78	52.00	2.50	14-אוג-1953	1	5
63.00	81.00	23.00	3.17	04-יול-1963	2	3
52.00	83.00	37.00	4.67	23-אוק-1953	2	5
86.00	52.00	31.00	3.67	13-מאי-1966	2	5
80.00	88.00	34.00	4.17	28-מאי-1948	4	3
61.00	78.00	22.00	4.83	02-פבר-1966	2	5
92.00	90.00	61.00	5.00	12-אוג-1953	2	#NULL!
74.00	74.00	38.00	2.00	20-יול-1955	2	6
83.16	65.00	43.00	2.17	09-אפר-1945	3	5
55.00	67.00	30.00	2.67	07-אוג-1957	3	3
58.00	73.68	25.00	2.17	16-דצמ-1956	3	3
91.00	86.00	26.00	1.17	27-ינו-1967	2	5
62.00	73.00	39.00	2.00	03-יונ-1942	4	5
72.00	66.00	32.00	3.00	13-מרץ-1946	2	2
57.00	73.00	40.00	2.67	09-ספט-1955	2	3
85.00	87.00	52.00	2.83	09-ספט-1955	2	4
95.00	87.00	21.00	1.50	27-אוק-2034	2	5
70.00	79.00	59.00	2.33	08-ינו-1957	2	3
71.58	71.00	28.80	4.50	13-אוק-1947	2	7
72.86	70.00	36.80	3.17	23-אוק-1954	2	6
66.00	94.00	61.00	1.33	23-דצמ-1941	2	2
52.00	68.00	43.00	3.83	21-אוק-1960	2	5
47.00	80.00	42.00	4.00	25-מרץ-1965	2	3
76.00	75.00	49.00	3.67	02-מאי-1957	2	6
79.00	75.00	56.00	3.50	15-אוק-1945	3	6
87.00	88.00	28.00	1.17	31-אוג-1945	2	6
55.00	78.00	46.00	4.33	#NULL!	2	2
79.00	81.00	51.00	3.83	19-יול-2036	2	#NULL!
75.00	86.00	50.00	1.17	27-אוק-1957	2	5
71.00	78.00	56.00	2.17	10-פבר-1954	2	5
84.00	78.95	28.00	2.00	09-ספט-1955	3	5
67.00	66.00	38.00	3.67	07-דצמ-1943	2	5
85.00	83.00	36.00	1.67	17-מרץ-1959	2	5
77.00	73.00	39.00	2.33	19-יול-1955	2	3
70.00	85.00	28.00	3.67	27-אפר-1958	2	6
65.00	80.00	50.00	2.83	10-מרץ-1959	3	6

82.00	85.00	46.00	4.17	#NULL!	3	6
83.00	72.00	47.00	3.17	25-אפר-1962	2	4
87.37	92.00	57.00	2.17	02-יונ-1957	2	2
61.00	75.00	42.00	2.83	20-ספט-1943	3	5
65.00	68.00	48.00	3.00	29-דצמ-1957	2	4
85.00	79.00	47.00	2.00	14-יול-1942	2	2
80.00	80.00	39.00	3.33	06-פבר-1956	2	5
82.00	83.00	63.00	1.17	02-ינו-1964	2	4
77.00	58.00	20.00	6.00	30-אוג-1943	1	5
68.00	72.00	34.00	2.33	12-מאי-1946	3	5
54.00	83.00	45.00	1.67	05-אוג-2035	4	2
57.00	84.00	32.00	3.33	22-ינו-1964	3	4
93.00	88.00	48.00	2.67	19-אוג-1953	2	5
66.32	78.00	66.00	4.00	08-אוק-1961	2	3
50.00	67.37	46.93	4.83	16-אוק-1951	3	5
53.00	74.00	18.00	5.17	01-נוב-1945	3	5
67.00	76.00	37.00	3.67	14-דצמ-1943	2	2
70.00	78.00	39.00	1.83	05-אפר-1943	2	6
62.00	83.00	30.00	3.00	25-דצמ-1944	3	6
85.00	79.00	40.00	1.33	10-אוג-2035	4	6
79.00	90.00	42.00	3.00	25-יונ-2037	1	4
79.00	73.00	34.00	2.00	11-פבר-1945	2	6
81.00	78.00	30.00	2.50	22-מאי-1963	2	6
77.00	76.00	53.00	2.50	17-אוק-1960	3	3
80.00	80.00	36.00	1.33	04-אוק-1943	2	5
89.00	89.00	70.40	1.50	02-דצמ-1944	2	3
67.00	74.00	59.00	2.50	07-אוג-1958	2	4
80.00	86.00	43.00	3.17	21-יול-1952	2	4
76.00	81.00	54.00	4.00	03-אוק-1943	4	3
71.00	85.00	46.00	2.83	24-מאי-1959	2	5
77.00	71.00	61.00	2.50	15-ינו-1966	1	5
75.00	66.00	53.33	4.67	07-אוק-2032	2	6
62.00	60.00	36.00	3.00	05-ספט-2033	2	6
78.00	87.37	47.00	2.50	17-פבר-1951	2	6
55.00	74.00	32.00	4.17	14-מאי-1951	3	4
72.00	77.00	64.00	3.33	07-ספט-2037	4	4
71.00	69.00	27.00	4.67	25-דצמ-1946	2	5
60.00	78.00	46.00	3.83	25-פבר-1967	3	5
51.00	62.00	30.93	3.83	09-ספט-1955	2	#NULL!
61.00	89.00	62.00	3.20	26-פבר-1942	3	6
76.00	75.00	43.00	#NULL!	07-דצמ-2034	2	5
75.00	73.00	34.00	3.67	27-מרץ-1953	2	5
64.00	77.89	27.00	4.17	07-פבר-1944	4	2
86.00	95.00	56.00	4.00	#NULL!	2	5
84.00	81.00	43.00	3.50	13-מרץ-1953	2	4
75.00	71.00	42.00	2.50	03-מאי-1957	2	3
82.00	83.00	52.00	2.00	11-נוב-1952	3	6
82.00	78.00	27.00	2.83	22-יול-2037	2	4
76.00	77.00	49.00	3.00	13-יונ-1967	2	5
68.00	79.00	66.00	2.67	25-מאי-1958	3	6
85.00	80.00	29.00	4.67	29-מרץ-1953	3	#NULL!
69.00	73.00	50.00	3.67	19-נוב-1949	-1	6
80.00	68.00	29.00	3.00	09-ספט-1955	2	5
69.00	75.79	28.31	2.17	10-מאי-1961	3	4
82.00	80.00	44.00	2.83	14-ספט-1955	3	5
63.00	59.00	38.00	1.83	25-פבר-1962	2	5

69.00	64.00	39.00	2.33	01-נוב-1942	2	4
61.00	73.00	53.00	2.00	30-יונ-1955	2	5
62.00	72.00	25.00	3.00	27-אוק-1962	2	3
67.00	65.00	50.00	3.17	13-אוג-1941	2	4
68.00	69.00	36.00	3.00	24-פבר-1961	3	3
80.00	75.00	34.00	4.83	19-ספט-1944	3	6
80.00	87.00	54.00	3.17	09-אוג-1946	2	6
76.00	72.00	27.00	3.00	06-ינו-2035	2	7
79.00	80.00	38.00	2.00	28-מרץ-1956	2	5
62.00	73.00	51.00	3.50	16-מאי-1957	3	3
85.00	83.00	37.00	3.17	06-פבר-1956	2	5
74.00	66.00	29.00	3.00	05-מרץ-2036	2	4
76.00	69.00	61.00	#NULL!	08-ינו-1953	3	5
54.00	74.00	29.00	3.33	13-מאי-1958	3	5
68.00	72.00	51.00	3.17	11-ינו-1965	2	5
74.00	70.53	37.00	3.50	20-פבר-1948	3	5
71.00	85.00	61.00	3.33	01-ינו-1956	2	2
75.00	72.00	30.00	2.83	21-אוק-1958	2	3
61.00	68.00	49.00	1.67	03-ספט-2027	2	2
89.00	90.00	52.00	1.67	24-פבר-1960	2	5
63.00	78.00	53.00	1.00	05-מרץ-1953	3	5
90.00	90.00	72.00	1.67	12-ינו-1957	3	4
68.00	80.00	45.00	5.50	#NULL!	2	5
59.00	75.71	58.67	#NULL!	30-אוג-1956	3	1
65.00	82.00	65.00	2.83	28-ספט-1953	2	5
66.00	72.00	31.00	5.00	16-ינו-1946	2	5
78.00	75.00	34.00	4.67	30-מאי-1959	2	7
86.00	89.00	23.00	2.17	06-אוג-1962	2	5
72.00	73.00	28.00	5.33	15-נוב-1949	2	5
83.00	84.00	64.00	4.80	#NULL!	2	3
60.00	64.00	32.00	3.00	04-ספט-1964	2	2
75.00	66.32	50.00	3.50	29-מרץ-1942	3	5
71.00	76.00	33.00	4.67	#NULL!	2	5
68.00	79.00	42.00	1.50	09-ספט-1955	3	4
68.00	80.00	62.00	2.33	27-יונ-1950	2	4
85.26	66.00	50.13	#NULL!	25-אוג-1949	2	5
71.00	71.00	22.00	5.33	30-נוב-2036	2	5
83.00	86.32	61.00	1.83	01-מאי-1946	2	5
75.00	73.00	31.00	2.33	18-מאי-1942	3	5
80.00	84.00	58.00	2.67	08-יול-1947	2	4
82.00	87.00	44.80	4.17	09-ספט-1955	2	4
69.00	80.00	38.00	3.83	13-מרץ-1954	2	#NULL!
80.00	85.26	30.93	2.50	03-מרץ-2035	2	2
81.00	87.00	53.00	4.33	06-ינו-1944	4	5
78.00	81.00	32.00	2.17	29-מאי-1956	2	6
74.00	69.00	49.00	#NULL!	06-ינו-2026	4	4
73.00	76.00	36.00	3.83	12-מרץ-2032	2	4
78.00	83.00	34.00	2.50	23-דצמ-1946	2	6
68.00	86.00	48.00	2.67	31-אוג-1958	2	5
84.00	87.00	58.00	1.83	15-אוק-1959	2	7
71.00	81.00	52.00	4.83	26-יונ-1951	3	#NULL!
60.00	83.00	58.00	3.00	09-ספט-1955	4	6
70.00	68.00	35.20	4.40	06-ינו-2036	2	5
62.00	69.00	53.00	3.67	17-פבר-1951	3	3
46.00	70.00	49.00	4.00	12-נוב-1952	2	#NULL!
84.00	88.00	31.00	1.83	02-מאי-1947	2	5

84.00	69.00	41.00	2.83	22-נוב-1950	2	4
69.00	75.00	46.00	2.17	28-יול-1953	2	7
84.00	74.00	31.00	2.50	09-ספט-1955	2	5
80.00	78.00	60.00	1.67	21-ספט-1945	3	3
80.00	80.00	41.00	3.17	19-דצמ-1962	2	5
72.00	74.00	43.00	2.17	22-אוק-1959	2	2
86.32	80.00	40.00	2.00	27-אוג-1953	2	5
53.00	66.00	40.00	2.50	29-אוג-1946	3	6
46.00	63.00	34.00	2.50	19-יול-1964	3	4
79.00	68.00	35.00	3.17	#NULL!	#NULL!	#NULL!
77.00	72.00	21.00	1.83	#NULL!	#NULL!	#NULL!
79.00	76.00	32.00	2.33	#NULL!	#NULL!	#NULL!
94.00	94.00	47.00	3.00	#NULL!	#NULL!	#NULL!
73.00	84.00	56.00	3.50	#NULL!	#NULL!	#NULL!
72.00	72.00	42.00	#NULL!	#NULL!	#NULL!	#NULL!
69.00	67.00	46.00	#NULL!	#NULL!	#NULL!	#NULL!
55.00	54.67	64.00	#NULL!	#NULL!	#NULL!	#NULL!
69.00	81.00	51.00	3.83	#NULL!	#NULL!	#NULL!

religion	children	birth_country	occupation	pas	sac
1	3	israel	Pharmacy	3	2
1	3	germany	Art	0	5
1	2	israel	Language Teacher	1	4
2	3	egypt	Pensioner	9	5
1	3	israel	Bookeeper	0	6
1	3	israel	Agriculturist	5	3
1	3	israel	999	1	3
1	3	romania	Computer Technician	1	-1
1	3	israel	Teacher	4	-1
1	4	israel	secretary	8	6
1	3	israel	Assist Dentist	3	3
1	3	israel	Archivist	4	-1
1	2	israel	Teacher	1	3
1	2	israel	Economist	2	3
1	2	israel	pensioner	2	3
1	3	israel	Teacher	1	2
1	3	israel	Social worker	2	3
1	3	israel	999	2	2
1	2	germany	Public Health	2	4
1	3	israel	archivist	0	2
1	5	israel	Self Employed	3	4
1	3	israel	Manager	3	5
1	3	israel	Financial exacutiva	3	2
1	2	israel	water agriculturist	1	4
1	3	israel	Psycologist	0	3
1	4	israel	Teacher	6	6
1	2	israel	business Advicer	5	5
1	2	israel	Manager	0	4
1	3	israel	Engineer	0	2
1	3	israel	Secretary	1	4
1	3	999	pensioner	3	3
1	3	israel	pensioner	4	2
1	1	israel	account managing	1	5
1	2	israel	account managing	0	6
#NULL!	#NULL!			#NULL!	#NULL!
#NULL!	#NULL!			#NULL!	#NULL!
#NULL!	#NULL!			#NULL!	#NULL!
#NULL!	#NULL!			#NULL!	#NULL!
1	3	israel	biology teacher	5	2
1	2	israel	teacher	0	3
1	2	germany	agriculture & indust	2	6
2	3	israel	pensioner	2	6
1	4	israel	pensioner	4	4
1	4	uruguay	pedicure	0	6
1	2	israel	events producer	0	1
3	5	israel	private business	7	2
1	4	brazil	quality assurance ma	5	2
1	-1	israel	Macanic	2	5
1	2	israel	security manager	6	3
1	4	israel	account manager	4	2
1	3	israel	education counsler	1	3
1	3	israel	nurse	0	4
1	3	israel	head secretery	1	6
1	5	israel	pensioner	1	6
1	0	israel	typist	0	6

2	5	israel	sewer	2	3
1	4	france	teacher	2	1
1	1	argentina	acquisitions manager	1	3
1	4	people's republic of	999	3	-1
1	2	israel	pensioner	0	6
1	4	israel	Secretery	9	5
1	4	switzerland	accountant	3	6
1	4	china	head of security	2	2
1	2	israel	Writing	0	2
1	5	israel	Sport teacher	9	6
1	4	israel	Agriculture	9	2
1	4	israel	Bookkipping	3	2
1	4	israel	Pension	4	3
1	4	israel	Agricalture	1	3
1	3	israel	accounting	4	3
1	3	israel	Artist	1	3
1	4	israel	self-employed	0	2
1	4	israel	Graphic designer	4	3
1	2	russia	telemarketing	6	4
1	3	israel	office manage	1	3
1	2	israel	bus driver	4	2
1	2	israel	information systems	3	4
1	3	israel	computer technician	0	2
1	2	israel	999	0	4
1	2	israel	teacher - pensioner	3	3
1	2	israel	sales	3	3
1	2	israel	teacher & counsultan	1	4
1	1	israel	spirtual teacher	1	6
1	4	germany	architect engineer	1	4
1	2	israel	visa coordinator	1	3
1	4	israel	agricultural	4	4
1	3	israel	maintaining heating	4	4
1	0	israel	dance teacher	9	2
1	2	israel	pedagogical secerta	2	4
1	3	argentina	family doctor	1	2
1	3	israel	occupational therapy	2	5
1	2	israel	marketing manager	0	4
1	2	israel	teacher	2	3
1	2	israel	software engineer	2	3
2	2	morocco	senior account manag	5	5
1	3	israel	parking lot manager	1	3
1	3	uruguay	Business manager	2	3
1	4	israel	Science administrati	3	3
1	1	israel	Social worker	2	5
1	4	israel	Bookkeeper	0	6
1	3	israel	Software Engineer	4	6
1	3	israel	Bookkeeper	2	5
1	3	israel	assigning of strange	3	3
1	3	israel	housewife	2	2
1	3	israel	self-employed	5	2
1	4	israel	teacher	5	6
1	4	israel	self-employed	1	2
1	1	iraq	Machine technician	5	2
1	2	israel	Furniture Painting	6	4
#NULL!	#NULL!			#NULL!	#NULL!
1	5	israel	Nanny	2	2

1	2	france	shoes designer	0	6
1	3	israel	economist	6	5
1	3	romania	culinary center mana	1	4
1	0	israel	engineer	0	4
1	2	israel	999	4	4
1	2	germany	administration	3	5
1	3	argentina	Industry and Managem	2	3
1	1	israel	cosmetician	2	2
1	1	israel	optician	1	2
1	2	israel	Department manager	1	5
1	4	israel	Engineer	1	5
1	3	israel	Natural medicine	6	3
2	4	israel	Store manager	3	3
1	3	israel	Machine engineer	1	3
2	2	israel	Electronic engineer	6	5
1	3	israel	bookkeeper	0	6
1	3	israel	human resources	5	2
2	3	israel	dietician	3	2
1	3	israel	pensioner	4	3
1	3	israel	librarian	4	3
1	3	israel	nurse	1	5
1	1	israel	sales and marketing	0	4
1	3	israel	teacher	3	2
1	3	israel	lawyer	4	2
1	3	israel	flowers interweaving	7	3
1	2	israel	quality manager	0	4
2	4	israel	investigator	7	4
1	3	romania	nurse	0	6
1	3	israel	pensioner	1	6
1	2	israel	assistant	1	1
1	3	israel	architect	4	1
1	3	israel	industry and managem	0	4
1	3	argentina	social worker	0	2
1	3	denmark	systems analysis	2	3
1	4	israel	999	5	5
-1	4	israel	teacher	5	5
1	3	israel	999	6	3
1	2	israel	export secretary	2	2
1	2	israel	pediatrician	3	-1
1	3	israel	manager	1	2
1	4	israel	acoount manager	0	4
1	4	israel	999	0	3
1	5	israel	movement instructor	1	2
1	3	israel	lawyer, account mana	5	6
2	3	israel	education	7	3
1	3	israel	biology teacher	2	3
1	1	israel	graphic artist	0	6
1	2	france	agricultural	6	5
1	3	poland	Teacher	0	4
2	4	israel	teacher	2	3
2	2	israel	engineer	5	4
1	2	israel	architect	3	3
1	2	israel	language aditing	1	1
1	2	israel	manager	7	4
1	2	israel	human resources	3	2
1	2	israel	social worker, speci	0	4

1	2	israel	Teacher	0	2
1	2	israel	Interior Designer	3	3
1	2	england	Lufthansa manager	1	5
1	3	israel	management and Educa	1	6
1	3	israel	insurance agent	1	4
1	2	999	999	4	3
1	3	romania	Industry and Managem	1	6
1	2	israel	Holistic medicine	4	4
1	0	poland	lawyer	1	4
1	3	romania	banking	9	3
1	5	israel	nurse	4	3
1	2	israel	computers	0	3
2	2	israel	kindergarten teacher	1	6
3	4	israel	coordinator	0	4
1	3	israel	lawyer	0	3
1	4	poland	electronics engineer	2	6
1	2	israel	secretary	4	6
1	3	israel	manager	9	2
1	3	israel	manager	0	5
1	3	israel	teacher guide	4	5
-1	0	israel	education	8	3
1	2	israel	archivist	0	6
1	3	israel	financial manager	2	2
1	3	israel	software	3	3
1	2	israel	pensioner	3	2
3	3	israel	consultant, advisor	5	3
1	3	israel	reflexology	2	3
1	2	latvia	budget	2	3
1	4	israel	kindergarten teacher	1	6
1	2	israel	lawyer	0	3
1	0	israel	software engineer	0	5
1	2	israel	veterinarian	1	5
1	2	israel	agronomist	1	6
2	3	israel	theacher	3	2
1	3	israel	art metalworker	6	2
-1	3	poland	medical secretery	4	3
1	5	romania	economist	5	5
1	0	israel	Bussiness managment	4	1
1	3	israel	Basketball coach	3	5
1	3	israel	teacher, educational	1	6
2	4	israel	education	4	3
1	3	israel	pharmacist	2	2
1	0	israel	secretary	1	6
2	5	israel	teacher, secretary	7	2
1	1	poland	practical engineer,	1	-1
2	2	israel	manager assistant	1	3
1	3	israel	management	1	3
1	3	turkey	technician	4	5
1	3	israel	lawyer	4	3
1	1	israel	Organizational consu	0	6
1	2	chile	secretary	1	5
1	2	israel	Teacher	2	3
1	3	israel	practical engineer,	4	3
2	1	israel	self-employed	0	4
1	3	poland	social worker	1	6
1	3	israel	secretary	1	4

2	4	israel	nurse	5	4
2	2	turkey	secretary	4	4
1	3	russia	electronic practical	1	3
2	3	yugoslavia	Building practical e	2	2
1	2	israel	salaried employee	2	0
1	1	poland	pensioner	2	6
1	5	poland	Teacher	2	2
1	3	israel	Engineer Advisor	3	3
1	2	romania	teaching	2	6
2	3	israel	bookkeeper	3	4
1	3	israel	paralegal	5	4
1	3	israel	pensioner	7	4
1	4	israel	999	5	2
1	1	ussr	journalist	0	4
3	6	israel	computers	1	2
1	3	israel	civilian engineer	0	2
1	3	iran	communication	0	3
1	3	israel	assistance manager	1	3
1	4	morocco	policeman	5	6
1	2	israel	self-employed	1	4
1	3	israel	teaching	0	3
2	2	israel	teacher	9	3
2	3	usa	occupational therapi	1	2
1	3	azerbaijan	999	0	6
2	6	israel	housekeeper	1	2
1	4	romania	housekeeper	4	2
1	2	israel	computers	1	5
2	3	israel	teacher	0	2
1	3	israel	physiotherapist	4	6
1	3	burma	preschool teacher	3	5
1	3	israel	999	1	2
1	3	israel	pensioner	4	3
1	2	germany	social worker	3	6
1	2	israel	quality engineer	3	4
2	5	israel	insurance agent cons	0	5
3	4	israel	education	3	4
1	5	israel	chemist	7	-1
1	2	israel	communication, books	6	2
1	1	israel	information manager	1	2
1	4	israel	guidance	2	2
1	3	israel	teacher	5	2
-1	3	israel	projects manager	1	-1
1	3	holland	Medical Secretary	4	5
1	3	canada	nurse	0	6
1	3	hungary	software technical s	1	2
2	2	usa	retired	0	6
1	3	israel	nurse	4	3
1	3	poland	manager	0	4
-1	3	israel	social worker	1	5
2	5	argentina	education, research	1	2
1	2	romania	consultant	3	-1
2	2	usa	teacher, discussion	1	6
1	3	israel	pensioner	3	6
1	3	israel	teacher	2	6
2	1	israel	999	3	6
1	3	cyprus	999	1	5

1	3	romania	accounting	2	1
1	3	israel	dentist	3	2
2	3	israel	economic consultant	4	5
3	2	israel	real estate marketin	0	6
1	2	israel	designer	2	5
1	3	israel	manager	1	4
1	3	argentina	sales manager	4	2
1	5	hungary	machines engineer	2	2
1	1	israel	architect	1	5
#NULL!	#NULL!			#NULL!	#NULL!
#NULL!	#NULL!			#NULL!	#NULL!
#NULL!	#NULL!			#NULL!	#NULL!
#NULL!	#NULL!			#NULL!	#NULL!
#NULL!	#NULL!			#NULL!	#NULL!
#NULL!	#NULL!			#NULL!	#NULL!
#NULL!	#NULL!			#NULL!	#NULL!
#NULL!	#NULL!			#NULL!	#NULL!
#NULL!	#NULL!			#NULL!	#NULL!

waist	hip	weight	height	ds_soc1	ds_neg1	typed1	NS1
94	99	78	178	18.00	10.00	1.00	61.00
96	104	73	163	6.00	20.00	0.00	65.00
97	122	87	165	13.00	13.00	1.00	51.00
95	95	80	178	9.00	6.00	0.00	48.00
103	108	95	177	6.00	2.00	0.00	39.00
95	109	88	186	1.00	1.00	0.00	52.00
80	96	56	163	9.00	3.00	0.00	50.00
100	104	70	169	5.00	4.00	0.00	56.00
92	113	80	177	7.00	6.00	0.00	63.75
85	99	58	148	4.00	0.00	0.00	43.00
91	115	74	158	6.00	2.00	0.00	51.00
39	112	78	150	8.00	8.00	0.00	66.00
91	104	68	155	6.00	8.00	0.00	60.00
102	104	90	184	0.00	9.00	0.00	52.00
104	102	85	181	16.00	5.00	0.00	52.00
72	86	57	160	10.00	8.00	0.00	51.00
80	100	56	159	8.00	6.00	0.00	54.00
87	110	64	158	17.00	6.00	0.00	44.00
108	111	84	178	10.00	12.00	1.00	56.84
86	105	61	164	25.00	9.00	0.00	50.00
92	106	83	175	3.00	4.00	0.00	52.00
86	109	80	169	5.00	7.00	0.00	52.00
94	100	70	172	11.00	4.00	0.00	49.00
91	94	80	180	7.00	7.00	0.00	62.00
75	97	64	165	13.00	11.00	1.00	68.00
85	95	62	159	5.00	5.00	0.00	61.00
110	125	83	168	0.00	5.00	0.00	52.00
100	103	92	187	14.00	7.00	0.00	76.00
106	108	90	180	6.00	10.00	0.00	54.00
79	105	54	151	9.00	6.00	0.00	52.00
999	999	79	165	4.00	4.00	0.00	53.00
85	93	73	182	10.00	2.00	0.00	60.00
81	99	56	155	14.00	16.00	1.00	62.00
96	117	82	173	19.00	6.00	0.00	41.00
#NULL!	#NULL!	#NULL!	#NULL!	#NULL!	#NULL!	#NULL!	54.00
#NULL!	#NULL!	#NULL!	#NULL!	#NULL!	#NULL!	#NULL!	53.00
#NULL!	#NULL!	#NULL!	#NULL!	#NULL!	#NULL!	#NULL!	44.00
#NULL!	#NULL!	#NULL!	#NULL!	#NULL!	#NULL!	#NULL!	63.00
91	101	66	159	10.00	4.00	0.00	47.00
87	115	70	167	9.00	6.00	0.00	58.00
110	105	90	179	6.00	7.00	0.00	57.00
85	106	58	155	3.00	3.00	0.00	61.00
93	106	78	170	8.00	11.00	0.00	66.00
95	112	83	163	4.00	9.00	0.00	55.00
78	91	66	167	1.00	14.00	0.00	74.00
999	999	87	178	5.00	3.00	0.00	55.00
75	98	60	160	7.00	9.00	0.00	52.00
98	99	71	170	10.00	4.00	0.00	43.00
87	97	77	181	11.00	13.00	1.00	51.00
71	94	56	160	8.00	0.00	0.00	60.00
92	102	62	159	3.00	4.00	0.00	47.00
107	113	78	160	6.00	11.00	0.00	65.00
87	102	63	151	2.00	10.00	0.00	62.00
94	110	80	172	8.00	11.00	0.00	55.00
89	109	78	165	10.00	11.00	1.00	51.00

87	108	66	162	21.00	6.00	0.00	47.00
94	111	71	163	4.00	6.00	0.00	57.00
58	62	140	170	7.00	8.00	0.00	55.00
106	106	76	178	17.00	15.00	1.00	55.00
79	100	65	160	12.00	10.00	1.00	60.00
88	120	70	155	5.00	11.00	0.00	56.00
98	96	70	178	19.00	3.00	0.00	43.00
100	103	82	184	8.00	7.00	0.00	55.00
90	127	87	158	7.00	8.00	0.00	54.00
85	90	62	170	11.00	13.00	1.00	51.00
94	98	74	172	1.00	5.00	0.00	67.00
86	100	61	153	11.00	12.00	1.00	44.00
90	112	69	160	5.00	7.00	0.00	56.00
104	105	86	181	11.00	4.00	0.00	47.00
80	104	61	168	17.00	18.00	1.00	56.00
86	102	56	147	15.00	9.00	0.00	66.00
90	99	75	184	11.00	3.00	0.00	63.00
77	103	60	167	12.00	3.00	0.00	49.00
89	96	66	171	7.00	14.00	0.00	51.00
94	105	74	163	11.00	9.00	0.00	47.00
95	102	85	182	11.00	2.00	0.00	56.00
99	108	93	174	6.00	9.00	0.00	52.00
80	91	54	158	9.00	8.00	0.00	63.00
97	103	79	170	18.00	5.00	0.00	59.00
100	118	86	170	18.00	16.00	1.00	47.00
98	97	76	172	4.00	7.00	0.00	58.00
120	120	114	195	13.00	11.00	1.00	55.00
99	115	90	172	5.00	0.00	0.00	55.00
999	999	80	168	16.00	13.00	1.00	53.00
94	107	73	166	7.00	3.00	0.00	50.00
62	60	90	181	1.00	2.00	0.00	57.00
90	104	82	183	17.00	10.00	1.00	49.00
65	87	49	160	8.00	13.00	0.00	57.00
90	110	69	165	8.00	5.00	0.00	63.00
95	97	76	174	1.00	12.00	0.00	55.00
88	105	75	167	6.00	4.00	0.00	56.00
108	103	90	170	2.00	12.00	0.00	43.00
75	100	62	169	8.00	15.00	0.00	49.00
89	101	75	180	21.00	28.00	1.00	34.00
96	94	70	170	14.00	1.00	0.00	54.00
102	96	80	172	4.00	8.00	0.00	68.00
98	98	80	175	10.00	7.00	0.00	63.00
94	105	72	168	21.00	7.00	0.00	52.00
106	132	89	164	3.00	8.00	0.00	58.00
104	130	95	166	17.00	16.00	1.00	54.00
102	111	91	174	13.00	12.00	1.00	61.00
79	102	66	164	12.00	11.00	1.00	49.00
97	120	88	168	12.00	8.00	0.00	65.00
73	86	45	159	3.00	0.00	0.00	55.00
95	97	78	174	9.00	10.00	0.00	53.00
88	105	68	153	7.00	0.00	0.00	61.00
80	103	65	168	2.00	0.00	0.00	48.00
81	94	65	175	2.00	11.00	0.00	54.00
80	96	70	165	11.00	5.00	0.00	66.00
#NULL!	#NULL!	#NULL!	#NULL!	#NULL!	#NULL!	#NULL!	#NULL!
92	110	73	166	17.00	7.00	0.00	54.00

94	113	70	160	1.00	12.00	0.00	63.00
92	99	79	180	8.00	8.00	0.00	48.00
110	116	97	180	7.00	5.00	0.00	51.00
87	96	67	168	21.00	9.00	0.00	42.00
80	98	56	174	7.00	8.00	0.00	60.00
86	104	65	155	11.00	4.00	0.00	48.00
98	103	80	176	7.00	5.00	0.00	54.74
73	96	51	154	3.00	5.00	0.00	69.00
91	102	75	182	7.00	12.00	0.00	62.00
77	101	60	161	21.00	12.00	1.00	52.00
115	115	110	185	13.00	3.00	0.00	62.00
77	94	48	154	15.00	16.00	1.00	58.00
99	103	85	185	11.00	8.00	0.00	46.00
102	105	89	174	0.00	5.00	0.00	59.00
95	105	84	188	15.00	4.00	0.00	53.00
128	116	100	170	16.00	14.00	1.00	56.00
92	116	85	172	0.00	4.00	0.00	67.00
90	100	78	165	4.00	6.00	0.00	64.00
87	94	68	168	13.00	3.00	0.00	49.00
81	102	68	163	11.00	12.00	1.00	60.00
82	99	63	168	11.00	10.00	1.00	65.00
96	96	75	174	8.00	8.00	0.00	50.00
78	100	55	158	16.00	15.00	1.00	47.00
102	102	85	172	9.00	9.00	0.00	61.00
82	99	64	165	19.00	10.00	1.00	58.00
87	100	70	174	19.00	12.00	1.00	60.00
97	52	72	170	4.00	5.00	0.00	68.00
102	113	74	159	9.00	18.00	0.00	55.00
90	106	73	162	14.00	0.00	0.00	62.00
85	107	74	178	16.00	15.00	1.00	46.00
81	105	74	172	9.00	13.00	0.00	55.00
95	110	75	163	1.00	3.00	0.00	52.00
91	112	69	167	11.00	11.00	1.00	57.00
85	113	74	175	15.00	5.00	0.00	51.00
96	100	77	172	11.00	12.00	1.00	57.00
84	100	64	162	13.00	3.00	0.00	48.00
84	99	56	170	4.00	3.00	0.00	41.00
81	98	62	170	17.00	16.00	1.00	50.00
97	98	80	180	19.00	8.00	0.00	46.00
93	104	75	175	10.00	6.00	0.00	51.00
105	101	86	178	9.00	8.00	0.00	61.00
71	96	58	172	11.00	18.00	1.00	59.00
77	96	60	177	12.00	21.00	1.00	67.00
79	92	57	173	12.00	12.00	1.00	50.00
74	100	57	160	8.00	5.00	0.00	50.00
82	98	58	154	15.00	10.00	1.00	44.00
81	107	74	178	8.00	15.00	0.00	66.00
66	63	52	150	4.00	4.00	0.00	47.00
100	120	92	165	8.00	12.00	0.00	58.00
102	117	76	160	6.00	5.00	0.00	64.00
99	107	84	179	7.00	4.00	0.00	65.00
90	95	63	169	11.00	13.00	1.00	56.00
68	103	64	165	1.00	2.00	0.00	56.00
99	106	90	186	12.00	3.00	0.00	49.00
98	97	75	172	9.00	16.00	0.00	51.00
75	88	52	158	5.00	13.00	0.00	59.00

78	97	60	164	11.00	13.00	1.00	51.00
84	107	67	164	4.00	9.00	0.00	57.00
116	116	103	183	3.00	7.00	0.00	60.00
93	118	80	155	16.00	14.00	1.00	64.00
95	103	83	175	7.00	17.00	0.00	56.00
76	95	65	157	9.00	7.00	0.00	62.11
130	120	107	170	10.00	4.00	0.00	59.00
109	99	75	165	4.00	12.00	0.00	64.00
80	92	63	164	15.00	9.00	0.00	50.00
999	999	77	172	9.00	11.00	0.00	60.00
83	120	83	157	18.00	4.00	0.00	48.00
110	112	101	186	14.00	9.00	0.00	55.00
94	101	61	157	15.00	18.00	1.00	62.00
80	101	999	160	6.00	11.00	0.00	67.00
104	120	69	172	19.00	17.00	1.00	59.00
93	100	80	171	28.00	20.00	1.00	43.00
93	102	65	154	16.00	10.00	1.00	59.00
98	103	80	173	13.00	18.00	1.00	50.00
116	114	100	176	13.00	6.00	0.00	57.00
85	105	67	164	6.00	6.00	0.00	63.00
84	97	57	154	5.00	1.00	0.00	47.00
84	99	63	175	7.00	7.00	0.00	62.00
101	128	104	191	4.00	5.00	0.00	50.00
92	99	75	175	11.00	12.00	1.00	50.00
97	105	87	182	6.00	9.00	0.00	54.00
999	999	62	167	4.00	2.00	0.00	57.00
86	101	66	170	3.00	7.00	0.00	62.00
110	122	89	164	5.00	1.00	0.00	62.00
90	109	68	166	14.00	15.00	1.00	49.00
85	95	70	188	8.00	14.00	0.00	63.00
96	102	89	172	3.00	7.00	0.00	55.00
102	97	82	170	13.00	23.00	1.00	50.00
111	103	82	173	13.00	19.00	1.00	58.00
97	104	67	160	5.00	6.00	0.00	61.00
88	93	71	174	13.00	14.00	1.00	58.00
84	102	61	158	11.00	7.00	0.00	67.00
94	98	73	164	9.00	8.00	0.00	64.00
94	101	84	174	16.00	25.00	1.00	70.00
96	92	74	170	26.00	24.00	1.00	54.00
98	107	70	158	7.00	12.00	0.00	62.00
999	999	69	172	12.00	9.00	0.00	57.00
76	98	54	159	8.00	8.00	0.00	52.00
92	104	65	163	21.00	17.00	1.00	46.00
86	106	63	156	11.00	1.00	0.00	49.00
89	104	62	160	4.00	8.00	0.00	49.00
65	96	57	154	8.00	17.00	0.00	58.00
80	99	62	169	3.00	3.00	0.00	73.00
96	101	75	162	11.00	15.00	1.00	64.00
99	102	83	176	7.00	8.00	0.00	55.00
107	118	92	165	11.00	15.00	1.00	58.00
83	99	55	158	7.00	2.00	0.00	45.00
999	999	79	168	3.00	13.00	0.00	63.00
102	104	76	164	7.00	8.00	0.00	57.00
89	97	70	178	10.00	14.00	1.00	56.67
99	111	76	170	7.00	11.00	0.00	56.00
103	112	89	161	9.00	8.00	0.00	63.00

96	105	73	160	17.00	20.00	1.00	55.00
95	100	72	164	1.00	5.00	0.00	59.00
95	97	90	175	14.00	9.00	0.00	53.00
110	104	90	179	6.00	10.00	0.00	56.00
99	94	80	179	14.00	13.00	1.00	52.00
86	100	63	163	16.00	12.00	1.00	47.37
80	104	65	164	5.00	1.00	0.00	54.00
136	124	120	180	9.00	6.00	0.00	51.00
105	108	86	163	9.00	11.00	0.00	59.00
108	119	87	157	11.00	10.00	1.00	60.00
96	112	86	163	0.00	15.00	0.00	58.00
102	97	73	169	14.00	6.00	0.00	46.00
96	99	77	184	15.00	2.00	0.00	51.00
62	98	70	999	17.00	9.00	0.00	48.00
107	103	88	180	11.00	13.00	1.00	53.00
127	123	118	178	13.00	8.00	0.00	65.00
60	95	50	157	12.00	24.00	1.00	58.00
99	106	93	190	15.00	7.00	0.00	60.00
94	98	60	155	5.00	19.00	0.00	52.00
64	102	73	169	7.00	0.00	0.00	64.00
70	88	59	163	1.00	8.00	0.00	55.00
83	100	65	158	1.00	1.00	0.00	57.00
91	107	77	173	7.00	8.00	0.00	58.00
88	103	61	155	7.00	12.00	0.00	69.00
97	126	88	159	4.00	8.00	0.00	50.00
90	104	68	165	21.00	20.00	1.00	53.00
77	89	59	170	16.00	7.00	0.00	49.00
110	133	101	156	11.00	13.00	1.00	51.00
79	103	57	172	11.00	9.00	0.00	49.00
83	106	64	157	9.00	10.00	0.00	57.00
99	104	69	154	22.00	16.00	1.00	54.00
108	106	89	180	1.00	8.00	0.00	64.00
88	108	67	160	8.00	13.00	0.00	68.00
100	95	72	173	5.00	6.00	0.00	49.00
98	105	86	181	8.00	5.00	0.00	68.00
95	112	68	163	3.00	10.00	0.00	53.00
96	97	73	178	9.00	2.00	0.00	50.00
87	115	70	170	2.00	3.00	0.00	62.00
78	101	62	161	5.00	9.00	0.00	52.00
81	103	66	162	8.00	2.00	0.00	68.00
102	108	73	155	12.00	4.00	0.00	48.00
108	107	80	168	17.00	11.00	1.00	41.00
86	100	60	164	11.00	16.00	1.00	54.74
105	125	80	165	8.00	1.00	0.00	62.00
100	104	82	180	10.00	16.00	1.00	50.00
27	37	50	153	6.00	6.00	0.00	44.00
67	92	51	151	12.00	7.00	0.00	47.00
93	97	67	163	9.00	2.00	0.00	64.00
80	95	56	158	16.00	7.00	0.00	48.00
80	104	60	159	12.00	10.00	1.00	53.00
81	109	73	162	3.00	3.00	0.00	64.00
81	102	60	165	9.00	12.00	0.00	69.33
106	116	88	180	10.00	8.00	0.00	62.11
85	96	52	160	18.00	9.00	0.00	57.00
103	108	80	172	21.00	18.00	1.00	55.00
76	96	53	158	18.00	3.00	0.00	50.00

81	92	63	164	10.00	10.00	1.00	57.00
107	112	97	180	8.00	10.00	0.00	53.00
94	105	79	176	10.00	2.00	0.00	51.00
90	122	85	173	8.00	6.00	0.00	54.00
100	100	75	172	15.00	10.00	1.00	49.00
106	113	98	181	4.00	7.00	0.00	69.00
110	110	99	178	0.00	8.00	0.00	57.00
101	109	80	173	6.00	17.00	0.00	63.00
90	95	74	183	8.00	20.00	0.00	63.00
#NULL!	#NULL!	#NULL!	#NULL!	#NULL!	#NULL!	#NULL!	#NULL!
#NULL!	#NULL!	#NULL!	#NULL!	#NULL!	#NULL!	#NULL!	#NULL!
#NULL!	#NULL!	#NULL!	#NULL!	#NULL!	#NULL!	#NULL!	#NULL!
#NULL!	#NULL!	#NULL!	#NULL!	#NULL!	#NULL!	#NULL!	#NULL!
#NULL!	#NULL!	#NULL!	#NULL!	#NULL!	#NULL!	#NULL!	#NULL!
#NULL!	#NULL!	#NULL!	#NULL!	#NULL!	#NULL!	#NULL!	#NULL!
#NULL!	#NULL!	#NULL!	#NULL!	#NULL!	#NULL!	#NULL!	#NULL!
#NULL!	#NULL!	#NULL!	#NULL!	#NULL!	#NULL!	#NULL!	#NULL!
#NULL!	#NULL!	#NULL!	#NULL!	#NULL!	#NULL!	#NULL!	#NULL!

HA1	RD1	PS1	SD1	CO1	ST1	tas_total1
38.00	62.11	65.00	86.00	75.00	32.00	1.65
80.00	81.00	64.00	56.00	88.00	58.00	1.70
64.00	56.00	63.00	73.00	79.00	35.00	1.55
50.00	66.00	73.00	72.00	67.00	49.00	16.00
38.00	41.00	75.00	84.00	74.00	26.00	1.61
36.00	74.74	76.00	90.00	87.00	39.00	1.20
48.00	71.00	76.00	90.00	81.00	39.00	1.21
39.00	60.00	86.00	87.00	67.00	25.00	1.45
53.33	80.00	53.00	78.67	49.00	28.00	1.70
47.00	81.00	67.00	76.00	76.00	52.00	12.00
54.00	65.26	59.00	83.00	83.00	33.00	1.05
52.00	69.00	58.00	86.00	78.00	41.00	1.32
45.00	84.00	72.00	68.00	86.00	59.00	1.35
38.00	63.00	84.00	76.00	74.00	45.00	1.63
51.00	57.00	71.00	64.00	65.00	45.00	2.15
51.00	63.00	64.00	78.00	74.00	40.00	1.85
43.00	72.00	68.00	80.00	76.00	49.00	1.70
66.00	65.00	36.00	90.00	83.00	18.00	1.33
51.00	61.11	63.00	54.74	63.00	32.00	1.75
67.00	51.00	62.00	70.00	80.00	28.00	2.83
31.00	59.00	68.00	81.00	74.00	23.00	1.40
36.00	57.00	65.00	76.00	84.00	48.00	0.65
46.00	59.00	76.00	81.00	74.00	34.00	1.45
42.00	48.00	65.00	77.00	69.00	26.00	2.20
56.00	64.00	62.00	71.00	78.00	26.00	1.65
56.00	74.00	55.00	82.00	79.00	35.00	1.35
46.00	74.00	92.00	72.00	64.00	37.00	1.20
64.00	75.00	70.00	72.00	79.00	43.00	1.45
40.00	62.00	66.00	80.00	69.00	33.00	1.65
42.00	66.00	64.00	83.00	82.00	43.00	0.60
55.00	74.00	55.00	77.00	77.00	31.00	1.85
45.00	64.00	58.00	90.00	84.00	18.00	1.40
71.00	65.00	52.00	64.00	69.00	28.00	1.60
45.00	57.00	77.00	80.00	73.00	18.00	2.17
51.00	79.00	70.00	81.00	82.00	56.00	1.45
32.00	64.00	73.00	95.00	89.00	46.00	1.15
78.00	72.00	46.00	65.00	81.00	38.00	1.85
51.00	72.00	76.00	75.00	72.00	38.00	2.45
56.00	71.00	67.00	86.00	84.00	39.00	1.15
53.00	69.00	56.00	71.58	86.00	54.00	1.65
41.00	69.00	71.00	88.00	81.00	33.00	1.61
53.00	77.00	61.00	74.00	74.00	46.00	1.25
60.00	81.00	62.00	78.00	74.00	32.00	1.30
48.00	86.00	72.00	79.00	77.00	35.00	0.90
48.00	75.00	77.00	64.00	85.00	59.00	1.75
31.00	75.00	92.00	76.00	66.00	44.00	1.35
50.00	74.00	63.00	87.00	83.00	42.00	1.35
47.00	68.00	73.00	83.00	76.00	34.00	1.75
35.00	71.00	90.00	84.00	76.00	39.00	1.50
28.00	43.00	70.00	90.00	60.00	25.00	0.75
31.00	70.00	81.00	79.00	91.00	37.00	0.95
58.00	69.00	58.00	59.00	77.00	26.00	2.45
52.00	87.00	62.00	82.00	84.00	44.00	1.45
56.00	68.00	72.00	82.00	79.00	60.00	1.45
57.00	64.00	63.00	71.00	74.00	31.00	1.45

63.00	56.00	64.00	78.00	74.00	30.00	2.50
53.00	63.00	81.00	67.00	72.00	55.00	1.80
48.00	71.00	76.00	80.00	74.00	38.00	1.65
63.00	72.00	75.00	70.00	79.00	57.00	0.61
55.00	61.00	73.00	82.00	78.00	36.00	1.50
53.00	72.00	75.00	74.00	76.00	53.00	1.55
47.00	47.00	74.00	71.00	74.00	24.00	1.63
47.00	68.00	63.00	74.00	72.00	32.00	1.42
47.00	71.00	54.00	52.00	92.00	44.00	1.70
71.00	50.00	61.00	69.00	60.00	34.00	2.58
32.00	81.00	73.00	69.00	62.00	49.00	1.45
62.00	71.00	62.00	80.00	70.00	26.00	1.20
55.00	69.00	51.00	76.00	85.00	39.00	0.85
52.00	57.00	63.00	78.00	73.00	37.00	1.68
74.00	65.00	59.00	58.00	71.00	50.00	2.00
63.00	61.00	43.00	90.00	82.00	36.00	2.00
53.00	54.00	69.00	82.00	82.00	34.00	1.40
41.00	50.00	68.00	83.00	72.00	34.00	1.35
59.00	79.00	70.00	56.00	80.00	41.00	1.50
55.00	71.00	63.00	78.00	87.00	39.00	1.25
38.00	67.00	52.00	94.00	92.00	27.00	1.45
42.00	62.00	66.00	75.00	77.00	31.00	1.60
47.00	65.00	75.00	76.00	83.00	54.00	1.15
56.00	53.00	68.00	63.00	76.00	36.00	2.16
70.00	60.00	63.00	69.00	73.00	38.00	2.50
46.00	75.00	60.00	71.00	59.00	31.00	1.95
54.00	58.00	69.00	66.00	74.00	33.00	1.80
37.00	73.00	68.00	82.00	86.00	69.00	1.00
56.00	53.00	71.00	75.00	71.00	34.00	2.35
48.00	69.00	81.00	88.00	90.00	56.00	0.85
35.00	65.00	62.00	89.00	83.00	51.00	1.10
55.00	68.00	71.00	72.00	88.00	50.00	1.05
72.00	81.00	70.00	66.00	81.00	36.00	1.25
55.00	70.00	65.00	71.00	78.00	55.00	1.60
40.00	67.00	69.00	79.00	86.00	34.00	1.21
55.00	75.00	58.00	81.00	81.00	43.00	1.50
58.00	74.00	76.00	65.00	74.00	26.00	1.60
64.00	74.00	73.00	55.00	72.00	24.00	1.50
85.00	56.00	61.00	39.00	63.00	23.00	1.72
55.00	56.00	65.00	80.00	72.00	29.00	1.75
47.00	78.00	70.00	66.00	75.00	49.00	1.45
47.00	56.00	71.00	73.00	69.00	32.00	2.16
52.00	50.00	82.00	83.00	78.00	29.00	1.75
52.00	63.00	75.00	84.00	70.00	45.00	1.30
55.00	56.00	61.00	63.00	62.00	42.00	2.15
46.00	54.00	60.00	77.00	78.00	32.00	1.55
70.00	62.00	72.00	70.00	72.00	43.00	1.80
54.00	67.00	63.00	59.00	74.00	39.00	2.00
42.00	70.00	73.00	79.00	73.00	47.00	1.05
62.00	61.00	47.00	60.00	73.00	34.00	2.20
42.00	57.00	69.00	80.00	68.00	20.00	1.15
33.00	70.00	79.00	79.00	85.00	30.00	1.10
49.00	88.00	84.00	80.00	86.00	34.00	1.90
43.00	68.00	60.00	78.00	88.00	46.00	2.26
#NULL!	#NULL!	#NULL!	#NULL!	#NULL!	#NULL!	#NULL!
57.00	66.00	50.00	63.00	78.00	41.00	1.58

52.00	70.00	73.00	56.84	70.00	66.00	1.50
47.00	55.00	65.00	82.00	76.00	24.00	1.65
59.00	74.00	60.00	67.37	73.00	41.00	1.10
54.00	46.00	62.00	76.00	65.00	24.00	1.80
44.00	69.00	53.00	74.00	68.00	37.00	0.90
56.00	58.00	63.00	78.00	78.00	45.00	1.55
47.37	57.00	65.00	84.21	82.00	21.00	2.41
34.00	78.00	47.00	79.00	86.00	38.00	1.35
48.00	63.00	85.00	76.00	72.00	28.00	2.55
62.00	45.00	61.00	74.00	77.00	31.00	1.65
40.00	67.00	72.00	75.00	76.00	35.00	1.35
61.00	63.00	60.00	68.00	74.00	34.00	2.00
49.00	58.00	67.00	69.00	66.00	28.00	2.15
44.00	72.00	77.00	77.00	77.00	29.00	1.45
55.00	68.00	69.00	73.00	76.00	51.00	1.40
69.00	60.00	68.00	61.00	62.00	41.00	2.60
32.00	82.00	68.00	59.00	77.00	24.00	1.75
50.00	83.00	53.00	80.00	87.00	56.00	0.65
50.00	54.00	55.00	69.00	75.00	41.00	1.40
51.00	76.00	73.00	68.00	68.00	47.00	2.00
52.00	62.00	65.00	76.00	70.00	53.00	1.45
61.00	56.00	64.00	76.00	69.00	28.00	1.37
61.00	67.00	58.00	73.00	79.00	35.00	1.60
38.00	64.00	70.00	79.00	67.00	31.00	2.39
66.00	67.00	82.00	76.00	81.00	35.00	1.84
65.00	53.00	68.00	60.00	76.00	23.00	1.65
37.00	76.00	71.00	95.00	94.00	59.00	0.95
59.00	77.00	79.00	59.00	85.00	24.00	1.10
55.00	60.00	70.00	83.00	71.00	40.00	1.20
72.00	62.00	55.00	61.00	72.00	45.00	1.50
50.00	64.00	74.00	63.00	66.00	28.00	1.50
27.00	78.00	88.00	97.00	81.00	21.00	1.90
63.16	67.00	61.00	70.00	74.00	41.00	2.00
65.00	67.00	60.00	69.00	74.00	42.00	1.75
67.00	65.00	54.00	64.00	71.00	33.00	2.53
50.00	56.00	65.00	90.00	81.00	47.00	1.80
48.00	70.00	80.00	91.00	82.00	18.00	1.15
68.00	64.00	71.00	63.00	76.00	50.00	1.25
58.00	56.00	69.00	74.00	74.00	26.00	1.80
52.00	53.00	70.00	75.00	69.00	44.00	2.20
39.00	65.00	54.00	71.00	78.00	54.00	1.58
73.68	61.00	54.00	63.00	64.00	33.00	1.05
64.21	64.00	60.00	47.00	74.00	44.00	1.90
65.00	67.00	57.00	74.00	69.00	42.00	1.60
50.00	57.00	70.00	80.00	68.00	53.00	1.70
61.00	69.00	66.00	83.00	83.00	23.00	0.90
61.00	72.00	60.00	69.00	79.00	40.00	2.10
47.00	53.00	68.00	79.00	85.00	46.00	1.30
54.00	78.00	71.00	73.00	83.00	49.00	1.85
43.00	73.00	59.00	75.00	79.00	57.00	1.85
42.00	67.00	62.00	82.00	77.00	45.00	1.95
62.11	64.00	54.00	62.00	64.00	34.00	1.95
30.00	75.00	60.00	85.00	89.00	32.00	0.80
42.00	72.00	77.00	78.00	77.00	40.00	2.00
54.00	61.00	79.00	67.00	82.00	24.00	1.70
52.00	74.00	52.00	64.00	84.00	50.00	0.90

59.00	67.00	65.00	61.00	78.00	38.00	24.00
44.00	82.00	82.00	83.00	72.00	52.00	1.70
35.00	71.00	82.00	82.00	90.00	43.00	1.45
75.00	67.00	63.00	58.00	75.00	44.00	1.85
55.79	75.00	78.00	70.00	67.00	46.00	2.10
55.00	63.00	59.00	70.00	86.00	51.00	0.85
49.00	66.00	72.00	79.00	84.00	53.00	1.30
47.00	75.00	59.00	59.00	77.00	53.00	1.55
48.00	46.00	80.00	83.00	59.00	18.00	1.68
48.00	72.00	74.00	79.00	72.00	37.00	1.90
61.00	81.00	62.00	56.00	76.00	48.00	1.25
52.00	73.00	76.00	57.00	77.00	46.00	1.45
65.00	75.00	84.00	75.00	79.00	47.00	2.47
64.00	71.00	70.00	61.00	73.00	45.00	1.85
82.00	57.00	45.00	46.00	59.00	42.00	2.40
74.00	52.00	29.00	36.00	59.00	26.00	1.85
64.00	57.00	56.00	60.00	68.00	37.00	1.75
53.00	62.00	86.00	72.00	78.00	33.00	1.05
40.00	60.00	75.00	61.00	79.00	47.00	1.40
39.00	59.00	71.00	79.00	77.00	42.00	1.05
35.00	67.00	82.00	95.00	89.00	38.00	1.05
48.00	68.00	71.00	74.00	74.00	43.00	1.75
33.00	62.00	83.00	90.00	79.00	21.00	1.90
63.00	68.00	72.00	77.00	82.00	40.00	1.80
40.00	68.00	73.00	82.00	73.00	36.00	1.75
43.00	61.00	60.00	87.00	75.00	72.00	0.74
43.00	75.00	64.00	81.00	79.00	62.00	1.20
43.00	74.00	58.00	93.00	84.00	53.00	1.05
77.00	68.00	61.00	71.00	75.00	53.00	2.25
39.00	66.00	79.00	70.00	86.00	48.00	1.45
39.00	69.00	84.00	73.00	71.00	54.00	1.60
68.00	53.00	70.00	73.00	78.00	49.00	1.95
52.00	48.00	67.00	64.00	70.00	31.00	2.15
32.00	82.00	76.00	92.00	89.00	35.00	1.25
56.00	65.00	52.00	54.00	79.00	33.00	1.72
55.00	66.00	62.00	62.00	74.00	62.00	1.60
57.00	61.00	65.00	67.00	65.00	31.00	1.95
68.00	67.00	55.00	33.00	79.00	44.00	2.30
70.00	46.00	73.00	55.00	68.00	32.00	2.72
48.00	72.00	52.00	72.00	89.00	53.00	1.10
51.00	55.00	69.00	73.00	73.00	37.00	1.80
50.00	69.00	61.00	76.00	79.00	33.00	1.75
59.00	53.00	47.00	51.00	66.00	24.00	1.74
45.00	66.00	66.00	81.00	82.00	49.00	1.40
55.00	73.00	70.00	71.58	77.00	44.00	1.35
71.00	69.00	68.00	70.00	79.00	34.00	1.55
49.00	73.00	74.00	87.00	86.00	48.00	1.05
60.00	68.00	44.00	72.00	71.00	30.00	1.74
46.00	68.00	66.00	77.00	80.00	43.00	1.50
66.00	49.00	64.00	59.00	78.00	64.00	1.17
46.00	80.00	73.00	83.00	81.00	39.00	1.05
54.00	67.00	78.00	56.00	70.00	55.00	2.50
46.00	70.00	72.00	74.00	73.00	34.00	1.95
74.00	56.00	58.00	48.00	66.00	23.00	1.75
47.00	78.00	73.00	83.00	84.00	44.00	0.75
57.00	69.00	68.00	61.00	63.00	33.00	1.75

68.00	71.00	63.00	54.00	59.00	40.00	2.35
43.00	87.00	69.00	77.00	83.00	56.00	1.15
56.00	61.00	57.00	67.00	71.00	32.00	2.00
52.00	67.00	75.00	68.00	67.00	48.00	1.80
65.00	68.00	53.00	60.00	69.00	37.00	1.90
60.00	54.00	71.00	76.00	67.00	28.00	1.72
37.00	79.00	87.00	81.00	86.00	54.00	0.68
40.00	48.00	88.00	81.00	79.00	27.00	1.55
62.00	74.00	49.00	75.00	78.00	41.00	1.40
65.00	61.00	61.00	66.00	69.00	38.00	2.10
37.00	67.00	74.00	74.00	81.00	43.00	2.05
55.00	49.00	75.00	79.00	74.00	36.00	1.55
45.00	69.00	76.00	74.00	74.00	53.00	1.35
74.00	67.00	61.00	66.00	67.00	30.00	1.80
59.00	66.00	59.00	68.00	74.00	50.00	2.20
52.00	57.00	67.00	78.00	70.00	29.00	1.85
74.74	73.68	66.00	51.58	74.00	45.00	2.10
49.00	54.00	78.00	69.00	78.00	40.00	2.10
58.00	73.00	73.00	48.00	70.00	57.00	1.63
31.00	69.00	61.00	80.00	86.00	46.00	1.05
44.00	67.00	69.00	72.00	79.00	32.00	1.75
32.00	72.00	75.00	94.00	91.00	71.00	1.45
65.00	78.00	53.00	59.00	82.00	53.00	1.60
41.00	74.00	76.00	52.00	63.00	59.00	2.45
46.00	68.00	62.00	77.00	82.00	59.00	1.90
78.00	49.00	60.00	50.00	68.00	27.00	3.00
58.00	56.00	70.00	73.00	71.00	35.00	2.05
64.00	66.00	72.00	79.00	90.00	39.00	1.25
69.00	61.00	62.00	67.00	72.00	31.00	1.50
52.00	62.00	63.00	80.00	80.00	55.00	1.85
75.00	71.00	66.00	58.00	70.00	37.00	2.50
38.00	78.00	93.00	78.00	76.00	59.00	1.05
47.00	66.00	74.00	72.00	73.00	43.00	1.55
46.00	68.00	69.00	64.00	79.00	44.00	1.85
52.00	70.00	72.00	78.00	78.00	43.00	1.05
57.00	70.00	55.00	80.00	58.00	45.00	1.80
50.00	50.00	64.00	77.00	68.00	28.00	1.79
50.00	67.00	67.00	80.00	82.00	58.00	1.25
61.00	73.00	63.00	72.00	76.00	27.00	1.55
45.00	70.00	67.00	80.00	86.00	52.00	1.60
43.00	65.00	82.00	89.00	86.00	40.00	1.26
60.00	59.00	70.00	73.00	82.00	46.00	2.65
41.00	78.00	79.00	75.00	79.00	36.00	2.35
44.00	61.00	57.00	77.00	84.00	48.00	1.70
50.00	70.00	75.00	78.00	68.00	37.00	1.90
57.00	71.00	72.00	68.00	73.00	46.00	2.15
61.05	67.00	62.00	70.00	80.00	33.00	1.60
43.00	58.00	74.00	82.00	83.00	32.00	1.60
60.00	67.00	53.00	75.00	85.00	48.00	1.55
52.00	68.00	70.00	72.00	78.00	53.00	1.20
44.00	68.00	63.00	75.00	75.00	30.00	1.35
71.43	76.67	44.00	61.25	68.00	50.00	1.65
56.00	52.00	63.00	68.00	65.00	46.00	2.15
65.00	68.00	37.00	63.00	71.00	43.00	2.20
82.00	64.00	37.00	33.00	58.00	44.00	2.65
59.00	62.00	59.00	80.00	88.00	20.00	1.65

40.00	72.00	86.00	76.00	65.00	32.00	0.95
56.00	76.00	72.00	78.00	73.00	49.00	1.70
43.00	63.00	84.00	81.00	72.00	41.00	2.65
49.00	71.00	69.00	77.00	77.00	57.00	1.30
52.00	68.00	71.00	61.00	75.00	44.00	1.70
44.00	67.00	71.00	74.00	73.00	40.00	1.75
38.00	73.00	68.00	88.00	78.00	44.00	0.85
63.00	75.00	56.00	61.00	68.00	45.00	2.00
72.00	75.00	56.00	48.00	64.00	43.00	1.60
#NULL!	#NULL!	#NULL!	#NULL!	#NULL!	#NULL!	#NULL!
#NULL!	#NULL!	#NULL!	#NULL!	#NULL!	#NULL!	#NULL!
#NULL!	#NULL!	#NULL!	#NULL!	#NULL!	#NULL!	#NULL!
#NULL!	#NULL!	#NULL!	#NULL!	#NULL!	#NULL!	#NULL!
#NULL!	#NULL!	#NULL!	#NULL!	#NULL!	#NULL!	#NULL!
#NULL!	#NULL!	#NULL!	#NULL!	#NULL!	#NULL!	#NULL!
#NULL!	#NULL!	#NULL!	#NULL!	#NULL!	#NULL!	#NULL!
#NULL!	#NULL!	#NULL!	#NULL!	#NULL!	#NULL!	#NULL!
#NULL!	#NULL!	#NULL!	#NULL!	#NULL!	#NULL!	#NULL!

tas_identify1	tas_describe1	tas_external1	npanas1	ppanas1	well_being1	social_support1
0.33	1.80	2.75	21.00	39.00	27.00	49.00
1.17	2.00	2.00	32.00	41.00	25.00	66.00
0.67	1.60	2.13	25.00	37.00	21.00	65.00
4.00	5.00	7.00	19.00	41.00	32.00	61.00
0.33	1.80	3.00	19.00	38.00	27.00	53.00
0.00	0.40	2.75	16.00	40.00	34.00	70.00
0.00	1.20	2.43	23.00	40.00	24.00	63.00
0.50	1.40	2.38	25.00	42.00	27.00	67.00
1.00	1.40	2.50	19.00	42.00	29.00	50.00
1.00	1.00	10.00	13.00	29.00	35.00	53.00
0.17	0.80	2.00	21.00	37.00	12.00	52.00
0.33	1.00	2.38	21.00	38.00	23.00	61.00
0.83	1.60	1.75	21.00	47.00	27.00	70.00
1.00	1.40	2.43	23.00	43.00	30.00	#NULL!
1.83	2.00	2.63	15.00	31.00	26.00	45.00
1.33	1.80	2.38	21.00	37.00	30.00	64.00
0.83	1.80	2.38	23.00	42.00	29.00	64.00
0.17	1.75	2.29	13.00	25.00	23.00	50.00
0.80	1.00	3.00	29.00	34.00	23.00	62.00
2.50	2.75	3.29	20.00	27.00	25.00	49.00
0.00	1.40	2.63	19.00	44.00	29.00	63.00
0.17	0.60	1.13	23.00	44.00	12.00	54.00
0.50	1.60	2.25	20.00	39.00	29.00	51.00
1.17	2.00	3.13	19.00	38.00	17.00	56.00
1.33	1.80	1.88	28.00	44.00	28.00	56.00
0.67	1.40	1.88	21.00	40.00	27.00	57.00
0.00	1.00	2.38	20.00	37.00	11.00	70.00
0.50	0.80	2.63	21.00	39.00	25.00	58.00
0.33	2.00	2.50	19.00	38.00	29.00	64.00
0.00	0.60	1.13	18.00	42.00	35.00	65.00
0.83	1.80	2.75	17.00	38.00	34.00	63.00
0.00	1.20	2.38	14.00	34.00	32.00	64.00
1.00	1.20	2.25	22.00	32.00	20.00	54.00
1.17	2.40	3.33	18.00	34.00	29.00	59.00
0.67	1.20	2.38	#NULL!	#NULL!	#NULL!	#NULL!
0.00	2.40	1.38	#NULL!	#NULL!	#NULL!	#NULL!
1.00	2.80	2.00	#NULL!	#NULL!	#NULL!	#NULL!
2.50	1.80	2.75	#NULL!	#NULL!	#NULL!	#NULL!
0.50	1.40	1.63	16.00	38.00	32.00	67.00
1.00	1.60	2.25	22.00	41.00	17.00	48.00
0.83	1.50	2.57	14.00	34.00	25.00	28.00
0.50	1.60	1.75	17.00	35.00	25.00	54.00
1.17	0.80	1.88	22.00	37.00	25.00	59.00
0.00	0.60	1.88	21.00	40.00	23.00	52.00
2.33	0.80	2.00	25.00	44.00	24.00	#NULL!
0.67	0.60	2.50	18.00	47.00	29.00	65.00
0.83	1.00	2.00	16.00	44.00	30.00	69.00
1.00	1.40	2.63	18.00	37.00	27.00	63.00
1.00	1.60	2.00	25.00	42.00	35.00	70.00
0.17	1.00	1.00	19.00	42.00	35.00	59.00
0.50	0.80	1.50	19.00	45.00	30.00	57.00
1.33	2.60	3.13	25.00	35.00	23.00	67.00
1.33	1.20	1.75	24.00	38.00	15.00	70.00
1.67	1.00	1.63	30.00	41.00	19.00	46.00
0.83	1.60	1.88	26.00	40.00	15.00	64.00

1.25	2.80	3.13	16.00	36.00	28.00	64.00
1.00	2.60	1.75	20.00	46.00	35.00	69.00
1.17	1.40	2.25	24.00	37.00	22.00	60.00
0.00	0.50	1.29	23.00	41.00	21.00	52.00
1.00	1.40	2.00	24.00	42.00	29.00	46.00
1.17	1.20	2.00	30.00	44.00	24.00	60.00
0.00	2.00	3.00	18.00	39.00	24.00	41.00
0.50	1.00	2.38	18.00	35.00	27.00	60.00
1.67	1.20	1.88	24.00	37.00	22.00	64.00
1.83	3.00	3.00	29.00	37.00	28.00	43.00
0.33	0.80	2.75	17.00	40.00	27.00	56.00
0.67	0.60	2.13	23.00	34.00	34.00	60.00
0.00	0.60	1.75	18.00	35.00	26.00	59.00
0.17	2.40	2.71	13.00	35.00	26.00	50.00
2.00	1.60	2.25	31.00	32.00	27.00	53.00
1.50	2.40	2.25	21.00	46.00	33.00	70.00
0.00	1.40	2.63	22.00	36.00	16.00	45.00
0.33	1.40	2.25	17.00	40.00	29.00	47.00
0.83	1.40	2.13	36.00	38.00	19.00	60.00
0.50	0.80	2.25	19.00	34.00	32.00	70.00
0.67	1.80	2.00	18.00	38.00	35.00	70.00
0.50	2.00	2.38	18.00	37.00	13.00	52.00
0.17	0.40	2.50	20.00	45.00	30.00	70.00
0.67	3.00	3.14	19.00	33.00	6.00	59.00
2.67	2.40	2.50	19.00	31.00	19.00	56.00
1.67	1.60	2.50	18.00	38.00	25.00	56.00
1.33	2.00	2.13	24.00	37.00	27.00	58.00
0.17	0.80	1.75	14.00	42.00	26.00	54.00
1.00	2.40	3.50	28.00	41.00	28.00	51.00
0.00	0.60	1.75	15.00	42.00	28.00	62.00
0.00	1.20	2.00	14.00	38.00	31.00	62.00
1.00	0.60	1.50	27.00	36.00	23.00	63.00
0.83	1.00	1.88	34.00	36.00	14.00	60.00
0.67	1.60	2.38	19.00	38.00	23.00	70.00
0.00	1.00	2.25	16.00	48.00	35.00	68.00
1.00	1.20	2.13	25.00	41.00	28.00	#NULL!
1.00	1.40	2.25	23.00	36.00	25.00	63.00
1.00	1.20	2.13	22.00	38.00	23.00	#NULL!
0.83	1.60	3.00	36.00	25.00	17.00	47.00
1.00	1.60	2.63	16.00	30.00	28.00	33.00
1.00	1.40	2.00	24.00	42.00	21.00	63.00
1.33	1.80	3.29	14.00	34.00	21.00	43.00
0.50	2.80	2.13	17.00	42.00	35.00	67.00
1.00	1.20	1.63	21.00	42.00	28.00	51.00
2.83	1.40	2.13	26.00	30.00	21.00	41.00
0.50	1.40	2.63	26.00	39.00	17.00	41.00
1.17	1.60	2.63	32.00	38.00	9.00	61.00
1.00	2.40	2.63	21.00	33.00	21.00	40.00
0.17	0.60	2.13	16.00	40.00	24.00	#NULL!
1.33	2.00	3.13	21.00	36.00	17.00	51.00
0.00	0.80	2.38	15.00	39.00	20.00	43.00
0.00	0.40	2.50	14.00	36.00	29.00	61.00
0.83	1.00	3.38	27.00	41.00	8.00	65.00
2.17	1.00	2.88	18.00	46.00	26.00	57.00
#NULL!	#NULL!	#NULL!	#NULL!	#NULL!	#NULL!	#NULL!
0.67	1.25	2.50	23.00	31.00	22.00	55.00

1.17	1.80	1.75	29.00	43.00	21.00	49.00
0.50	1.40	2.88	19.00	32.00	28.00	60.00
0.33	0.40	2.25	24.00	35.00	18.00	40.00
0.33	2.60	2.63	22.00	32.00	24.00	56.00
0.67	0.60	1.25	25.00	36.00	23.00	60.00
1.00	1.60	2.00	19.00	34.00	26.00	64.00
1.00	2.80	3.50	22.00	42.00	28.00	58.00
0.50	1.00	2.13	19.00	41.00	28.00	69.00
1.83	2.00	3.50	23.00	37.00	27.00	48.00
0.50	2.80	1.88	29.00	37.00	28.00	60.00
0.83	1.60	1.63	22.00	41.00	30.00	69.00
0.67	2.40	2.75	20.00	29.00	23.00	55.00
1.33	2.00	2.88	23.00	38.00	24.00	47.00
0.33	1.40	2.38	18.00	40.00	24.00	60.00
0.50	1.40	2.25	22.00	42.00	29.00	66.00
2.83	2.00	2.88	29.00	42.00	25.00	50.00
1.00	2.20	2.25	24.00	46.00	22.00	50.00
0.00	0.40	1.38	13.00	38.00	34.00	67.00
0.67	1.40	2.13	16.00	37.00	29.00	64.00
1.00	2.20	2.50	28.00	45.00	28.00	59.00
0.83	1.60	1.88	30.00	47.00	24.00	41.00
0.00	1.00	2.63	21.00	33.00	16.00	51.00
0.67	1.20	2.75	25.00	32.00	19.00	55.00
1.00	2.50	3.43	21.00	40.00	34.00	46.00
1.17	2.40	2.29	25.00	43.00	28.00	58.00
0.50	2.40	2.13	32.00	38.00	17.00	61.00
0.00	0.60	2.00	18.00	44.00	29.00	70.00
0.67	0.60	1.88	34.00	40.00	14.00	44.00
0.83	1.00	1.63	17.00	35.00	30.00	58.00
1.67	1.40	1.50	29.00	35.00	27.00	67.00
0.50	1.40	2.38	22.00	34.00	26.00	52.00
0.83	1.20	3.38	16.00	40.00	31.00	66.00
1.50	2.00	2.63	21.00	40.00	24.00	42.00
1.17	1.80	2.25	23.00	35.00	27.00	58.00
2.50	2.25	2.75	24.00	35.00	29.00	57.00
1.17	2.20	2.25	22.00	43.00	33.00	62.00
0.17	0.60	2.38	17.00	38.00	29.00	70.00
1.00	1.20	1.63	25.00	39.00	21.00	54.00
0.50	2.20	2.63	24.00	36.00	20.00	49.00
0.83	2.60	3.25	18.00	40.00	28.00	56.00
1.83	2.20	1.14	20.00	37.00	29.00	56.00
0.50	0.80	1.63	23.00	30.00	14.00	33.00
1.33	2.20	2.00	34.00	32.00	12.00	43.00
1.00	1.40	2.25	29.00	38.00	28.00	56.00
1.83	1.80	1.75	19.00	37.00	31.00	46.00
0.00	0.60	1.88	19.00	35.00	27.00	64.00
1.50	2.80	2.25	24.00	36.00	23.00	#NULL!
0.67	1.60	1.75	14.00	35.00	22.00	35.00
1.50	2.00	2.13	29.00	42.00	28.00	45.00
1.17	1.60	2.63	22.00	41.00	30.00	62.00
1.17	2.00	2.63	17.00	38.00	26.00	54.00
1.33	1.60	2.75	22.00	26.00	23.00	49.00
0.17	0.40	1.63	19.00	42.00	29.00	70.00
1.00	1.80	3.00	18.00	39.00	20.00	47.00
1.33	2.20	1.63	29.00	36.00	22.00	55.00
0.67	0.40	1.50	33.00	40.00	11.00	55.00

8.00	7.00	9.00	26.00	38.00	17.00	40.00
1.83	0.80	2.38	21.00	45.00	33.00	68.00
1.17	0.60	2.13	19.00	40.00	29.00	64.00
1.33	2.40	2.00	35.00	37.00	8.00	35.00
1.33	1.60	3.00	26.00	40.00	22.00	50.00
0.67	0.60	1.25	22.00	39.00	17.00	58.00
0.33	1.60	2.00	20.00	40.00	23.00	60.00
0.83	1.40	2.25	23.00	41.00	24.00	60.00
0.00	1.60	3.43	17.00	32.00	28.00	13.00
1.67	1.40	2.25	21.00	35.00	22.00	49.00
0.33	0.60	2.25	18.00	34.00	31.00	66.00
0.00	1.20	2.88	17.00	26.00	7.00	18.00
3.00	2.80	1.57	34.00	38.00	26.00	49.00
2.00	1.20	2.25	23.00	36.00	14.00	55.00
2.33	2.20	2.63	28.00	34.00	13.00	32.00
3.00	0.80	1.50	24.00	18.00	6.00	25.00
1.17	1.40	2.38	31.00	34.00	20.00	45.00
0.17	0.80	2.00	30.00	44.00	24.00	45.00
0.83	1.60	1.88	18.00	35.00	13.00	36.00
0.00	0.80	2.00	19.00	43.00	27.00	67.00
0.00	0.40	2.38	15.00	45.00	33.00	62.00
1.17	1.80	2.25	24.00	39.00	26.00	60.00
0.50	1.80	3.13	15.00	38.00	25.00	53.00
1.17	2.80	1.88	28.00	40.00	26.00	48.00
1.17	1.60	2.38	22.00	37.00	31.00	52.00
0.33	0.40	1.43	20.00	45.00	35.00	70.00
0.83	1.00	1.63	22.00	40.00	29.00	67.00
0.17	1.20	1.86	16.00	34.00	25.00	62.00
2.00	2.60	2.38	26.00	25.00	22.00	37.00
1.00	1.80	1.63	29.00	41.00	26.00	46.00
1.50	1.00	2.13	18.00	43.00	19.00	46.00
1.00	1.80	2.75	36.00	41.00	30.00	51.00
1.50	2.20	2.75	23.00	33.00	20.00	36.00
0.00	0.60	2.75	20.00	43.00	28.00	70.00
1.17	1.00	2.57	18.00	34.00	18.00	45.00
1.33	1.40	2.00	26.00	41.00	17.00	49.00
0.67	1.80	3.00	25.00	39.00	24.00	41.00
2.33	2.60	1.88	42.00	33.00	18.00	50.00
2.40	3.25	2.63	41.00	30.00	11.00	44.00
0.67	1.40	1.38	23.00	38.00	19.00	41.00
1.33	1.20	2.75	17.00	32.00	19.00	47.00
0.83	1.40	2.75	16.00	39.00	27.00	54.00
0.33	1.80	3.00	38.00	26.00	9.00	10.00
0.67	2.00	1.75	16.00	41.00	34.00	#NULL!
1.00	1.40	1.63	28.00	42.00	15.00	54.00
0.50	1.80	2.38	32.00	35.00	27.00	41.00
0.17	0.40	2.25	16.00	43.00	31.00	54.00
0.33	1.75	2.88	19.00	33.00	19.00	46.00
1.00	1.40	2.00	19.00	39.00	27.00	67.00
0.50	1.50	1.71	28.00	38.00	17.00	51.00
0.67	0.60	1.75	21.00	45.00	24.00	61.00
2.50	2.80	2.25	29.00	40.00	19.00	47.00
1.00	1.80	3.00	22.00	39.00	20.00	43.00
1.50	0.80	2.75	24.00	26.00	7.00	35.00
0.17	0.60	1.38	23.00	40.00	28.00	64.00
1.33	1.40	2.38	16.00	38.00	19.00	47.00

1.83	2.20	2.75	33.00	35.00	17.00	47.00
0.17	0.60	2.25	19.00	40.00	27.00	65.00
1.00	2.40	2.50	26.00	36.00	13.00	44.00
1.33	1.40	2.50	18.00	33.00	16.00	52.00
1.17	1.60	2.75	26.00	33.00	20.00	45.00
1.17	1.75	2.29	23.00	39.00	12.00	44.00
0.50	0.00	1.25	17.00	43.00	30.00	61.00
0.50	1.80	2.38	20.00	37.00	29.00	61.00
1.17	1.20	1.75	23.00	35.00	18.00	47.00
1.17	2.20	2.88	27.00	34.00	10.00	45.00
2.00	1.60	2.38	19.00	43.00	27.00	43.00
0.83	1.80	2.13	21.00	36.00	31.00	61.00
0.67	1.60	1.88	19.00	44.00	25.00	42.00
1.00	1.60	2.63	29.00	35.00	22.00	48.00
1.50	2.00	2.88	31.00	31.00	28.00	56.00
1.17	1.60	2.50	23.00	41.00	29.00	49.00
2.50	1.40	2.38	38.00	40.00	19.00	51.00
1.50	2.60	2.38	19.00	41.00	27.00	56.00
0.80	1.00	2.75	19.00	35.00	21.00	55.00
0.67	1.20	1.43	16.00	36.00	28.00	51.00
1.00	1.80	2.38	16.00	44.00	26.00	70.00
0.67	2.20	1.63	13.00	42.00	29.00	60.00
1.17	1.20	2.38	28.00	36.00	26.00	#NULL!
2.83	1.40	2.75	24.00	41.00	22.00	43.00
1.83	2.00	2.00	24.00	36.00	21.00	43.00
2.83	1.75	3.86	30.00	25.00	9.00	30.00
1.33	2.00	2.75	20.00	41.00	24.00	42.00
0.33	1.00	2.25	23.00	38.00	35.00	70.00
0.67	1.60	2.13	18.00	30.00	19.00	69.00
1.00	1.40	2.88	27.00	39.00	22.00	#NULL!
2.50	2.60	2.50	32.00	32.00	19.00	36.00
0.00	1.60	1.63	26.00	47.00	25.00	55.00
1.00	1.60	2.00	29.00	44.00	28.00	#NULL!
0.33	2.60	2.75	20.00	35.00	29.00	62.00
0.33	0.60	2.00	18.00	41.00	21.00	39.00
1.00	1.00	2.88	23.00	41.00	25.00	54.00
0.67	2.00	2.86	17.00	31.00	25.00	52.00
0.50	1.20	1.88	15.00	43.00	27.00	52.00
0.83	1.80	1.88	26.00	38.00	22.00	60.00
1.17	1.60	2.13	22.00	41.00	29.00	69.00
0.33	1.20	2.29	17.00	52.00	29.00	65.00
2.33	3.00	2.88	26.00	35.00	27.00	37.00
1.67	2.80	2.75	29.00	45.00	21.00	49.00
1.00	1.60	2.38	16.00	34.00	25.00	45.00
1.67	1.40	2.25	29.00	37.00	22.00	47.00
1.33	2.60	2.63	17.00	40.00	26.00	58.00
1.00	1.40	2.25	23.00	35.00	26.00	33.00
1.17	1.60	2.13	15.00	37.00	26.00	70.00
1.17	1.40	2.00	21.00	37.00	21.00	59.00
1.17	1.00	1.50	22.00	43.00	21.00	53.00
0.83	1.60	1.63	16.00	43.00	23.00	55.00
2.00	1.20	1.75	27.00	39.00	23.00	50.00
1.33	1.80	3.00	23.00	32.00	29.00	54.00
3.00	2.20	1.75	29.00	29.00	19.00	61.00
2.50	2.80	2.75	35.00	25.00	6.00	31.00
0.83	2.00	2.13	17.00	33.00	20.00	50.00

0.00	1.60	1.38	20.00	40.00	24.00	44.00
1.00	1.60	2.38	19.00	43.00	27.00	67.00
3.00	2.40	2.75	23.00	43.00	25.00	44.00
0.50	1.20	2.13	15.00	41.00	24.00	60.00
1.17	1.80	2.13	30.00	45.00	23.00	58.00
1.00	1.60	2.50	18.00	43.00	19.00	49.00
0.17	0.40	1.75	16.00	42.00	32.00	62.00
1.83	2.00	2.25	35.00	35.00	14.00	43.00
1.67	1.20	1.88	38.00	35.00	13.00	52.00
#NULL!	#NULL!	#NULL!	#NULL!	#NULL!	#NULL!	#NULL!
#NULL!	#NULL!	#NULL!	#NULL!	#NULL!	#NULL!	#NULL!
#NULL!	#NULL!	#NULL!	#NULL!	#NULL!	#NULL!	#NULL!
#NULL!	#NULL!	#NULL!	#NULL!	#NULL!	#NULL!	#NULL!
#NULL!	#NULL!	#NULL!	#NULL!	#NULL!	#NULL!	#NULL!
#NULL!	#NULL!	#NULL!	#NULL!	#NULL!	#NULL!	#NULL!
#NULL!	#NULL!	#NULL!	#NULL!	#NULL!	#NULL!	#NULL!
#NULL!	#NULL!	#NULL!	#NULL!	#NULL!	#NULL!	#NULL!
#NULL!	#NULL!	#NULL!	#NULL!	#NULL!	#NULL!	#NULL!

subjective_health1	sexual_activity	ppanas_z	npanas_z	smoking_currently	years_smoking
4.00	10.00	0.12	-0.41	0.00	0.00
2.00	#NULL!	#NULL!	#NULL!	#NULL!	#NULL!
3.00	1.00	0.31	0.26	0.00	14.00
1.00	0.25	1.11	-0.74	0.00	0.00
5.00	0.00	-0.08	-0.74	0.00	0.00
5.00	4.00	0.31	-1.23	0.00	7.00
5.00	4.00	0.51	-0.07	1.00	9.00
4.00	#NULL!	0.91	0.26	0.00	17.00
4.00	#NULL!	#NULL!	#NULL!	#NULL!	#NULL!
1.00	0.00	-1.27	-1.73	0.00	0.00
4.00	4.00	-0.28	-0.41	0.00	0.00
5.00	#NULL!	#NULL!	#NULL!	#NULL!	#NULL!
4.00	#NULL!	#NULL!	#NULL!	#NULL!	#NULL!
4.00	#NULL!	#NULL!	#NULL!	#NULL!	#NULL!
3.00	4.00	-1.47	-1.40	0.00	18.00
4.00	#NULL!	#NULL!	#NULL!	#NULL!	#NULL!
4.00	#NULL!	#NULL!	#NULL!	#NULL!	#NULL!
4.00	10.00	-2.46	-1.73	0.00	0.00
5.00	1.00	-0.48	0.92	0.00	25.00
4.00	10.00	-1.87	-0.57	0.00	0.00
4.00	1.00	1.11	-0.74	0.00	0.00
4.00	#NULL!	#NULL!	#NULL!	#NULL!	#NULL!
4.00	#NULL!	#NULL!	#NULL!	#NULL!	#NULL!
4.00	1.00	0.31	-0.74	1.00	0.00
3.00	4.00	1.31	0.75	0.00	0.00
4.00	#NULL!	#NULL!	#NULL!	#NULL!	#NULL!
3.00	#NULL!	#NULL!	#NULL!	#NULL!	#NULL!
3.00	#NULL!	#NULL!	#NULL!	#NULL!	#NULL!
5.00	#NULL!	#NULL!	#NULL!	#NULL!	#NULL!
5.00	#NULL!	#NULL!	#NULL!	#NULL!	#NULL!
5.00	#NULL!	#NULL!	#NULL!	#NULL!	#NULL!
5.00	10.00	-0.68	-1.56	0.00	0.00
4.00	0.25	-0.48	-0.24	1.00	30.00
5.00	#NULL!	#NULL!	#NULL!	#NULL!	#NULL!
#NULL!	#NULL!	#NULL!	#NULL!	#NULL!	#NULL!
#NULL!	#NULL!	#NULL!	#NULL!	#NULL!	#NULL!
#NULL!	#NULL!	#NULL!	#NULL!	#NULL!	#NULL!
#NULL!	#NULL!	#NULL!	#NULL!	#NULL!	#NULL!
5.00	#NULL!	#NULL!	#NULL!	#NULL!	#NULL!
3.00	#NULL!	#NULL!	#NULL!	#NULL!	#NULL!
4.00	#NULL!	#NULL!	#NULL!	#NULL!	#NULL!
4.00	0.00	-0.68	-1.07	0.00	0.00
3.00	#NULL!	#NULL!	#NULL!	#NULL!	#NULL!
4.00	#NULL!	#NULL!	#NULL!	#NULL!	#NULL!
3.00	#NULL!	#NULL!	#NULL!	#NULL!	#NULL!
5.00	10.00	1.90	-0.90	0.00	0.00
4.00	10.00	0.91	-1.23	0.00	14.00
4.00	#NULL!	#NULL!	#NULL!	#NULL!	#NULL!
5.00	4.00	1.50	0.26	0.00	0.00
5.00	#NULL!	#NULL!	#NULL!	#NULL!	#NULL!
4.00	4.00	1.50	-0.74	0.00	0.00
3.00	#NULL!	#NULL!	#NULL!	#NULL!	#NULL!
3.00	#NULL!	#NULL!	#NULL!	#NULL!	#NULL!
4.00	#NULL!	#NULL!	#NULL!	#NULL!	#NULL!
2.00	0.00	0.51	0.42	0.00	1.50

	5.00	4.00	-0.48	-1.23	0.00	0.00
	5.00	#NULL!	#NULL!	#NULL!	#NULL!	#NULL!
	2.00	4.00	-0.08	0.09	0.00	0.00
	5.00	#NULL!	0.71	-0.07	0.00	0.00
	5.00	0.00	1.11	0.09	0.00	0.00
	3.00	#NULL!	#NULL!	#NULL!	#NULL!	#NULL!
	5.00	#NULL!	#NULL!	#NULL!	#NULL!	#NULL!
	4.00	10.00	-0.48	-0.90	0.00	0.00
	4.00	#NULL!	#NULL!	#NULL!	#NULL!	#NULL!
	3.00	0.00	0.12	0.92	0.00	5.00
	5.00	10.00	0.51	-1.07	0.00	0.00
	5.00	10.00	-0.68	-0.07	0.00	0.00
	4.00	#NULL!	#NULL!	#NULL!	#NULL!	#NULL!
	4.00	#NULL!	#NULL!	#NULL!	#NULL!	#NULL!
	4.00	4.00	-0.88	1.25	0.00	0.00
	5.00	4.00	1.70	-0.41	0.00	8.00
	4.00	10.00	-0.28	-0.24	0.00	0.00
	5.00	#NULL!	#NULL!	#NULL!	#NULL!	#NULL!
	2.00	1.00	0.12	2.08	1.00	10.00
	4.00	#NULL!	#NULL!	#NULL!	#NULL!	#NULL!
	4.00	10.00	-0.28	-0.90	0.00	13.00
	4.00	#NULL!	#NULL!	#NULL!	#NULL!	#NULL!
	4.00	#NULL!	#NULL!	#NULL!	#NULL!	#NULL!
	4.00	1.00	-1.27	-0.74	0.00	0.00
	3.00	4.00	-1.07	-0.74	0.00	32.00
	5.00	#NULL!	#NULL!	#NULL!	#NULL!	#NULL!
	3.00	1.00	0.12	0.09	0.00	0.00
	5.00	#NULL!	#NULL!	#NULL!	#NULL!	#NULL!
	4.00	#NULL!	#NULL!	#NULL!	#NULL!	#NULL!
	5.00	4.00	0.71	-1.40	0.00	17.00
	5.00	1.00	-0.08	-1.56	0.00	0.00
	5.00	1.00	-0.08	0.59	0.00	0.00
	3.00	#NULL!	#NULL!	#NULL!	#NULL!	#NULL!
	4.00	#NULL!	#NULL!	#NULL!	#NULL!	#NULL!
	5.00	10.00	1.70	-1.23	0.00	0.00
	5.00	#NULL!	#NULL!	#NULL!	#NULL!	#NULL!
	4.00	#NULL!	#NULL!	#NULL!	#NULL!	#NULL!
	4.00	#NULL!	#NULL!	#NULL!	#NULL!	#NULL!
	2.00	4.00	-1.87	2.08	0.00	0.00
	4.00	0.25	-1.27	-1.23	1.00	46.00
	3.00	#NULL!	#NULL!	#NULL!	#NULL!	#NULL!
	4.00	#NULL!	#NULL!	#NULL!	#NULL!	#NULL!
	5.00	#NULL!	#NULL!	#NULL!	#NULL!	#NULL!
	4.00	#NULL!	#NULL!	#NULL!	#NULL!	#NULL!
	3.00	0.00	-0.88	0.42	0.00	10.00
	4.00	0.00	0.71	0.42	0.00	25.00
	3.00	0.25	0.51	1.42	0.00	5.00
	5.00	#NULL!	#NULL!	#NULL!	#NULL!	#NULL!
	4.00	#NULL!	#NULL!	#NULL!	#NULL!	#NULL!
	5.00	#NULL!	#NULL!	#NULL!	#NULL!	#NULL!
	5.00	#NULL!	#NULL!	#NULL!	#NULL!	#NULL!
	5.00	#NULL!	#NULL!	#NULL!	#NULL!	#NULL!
	4.00	10.00	0.91	0.59	0.00	5.00
	4.00	#NULL!	#NULL!	#NULL!	#NULL!	#NULL!
#NULL!		#NULL!	#NULL!	#NULL!	#NULL!	#NULL!
	5.00	10.00	-1.07	-0.07	0.00	13.00

4.00	#NULL!	#NULL!	#NULL!	#NULL!	#NULL!
4.00	0.25	-1.27	-0.74	1.00	50.00
4.00	#NULL!	#NULL!	#NULL!	#NULL!	#NULL!
4.00	1.00	-1.27	-0.24	0.00	0.00
5.00	#NULL!	#NULL!	#NULL!	#NULL!	#NULL!
4.00	#NULL!	#NULL!	#NULL!	#NULL!	#NULL!
4.00	#NULL!	#NULL!	#NULL!	#NULL!	#NULL!
5.00	#NULL!	#NULL!	#NULL!	#NULL!	#NULL!
4.00	#NULL!	#NULL!	#NULL!	#NULL!	#NULL!
5.00	0.25	-0.28	0.92	0.00	0.00
4.00	#NULL!	#NULL!	#NULL!	#NULL!	#NULL!
4.00	#NULL!	#NULL!	#NULL!	#NULL!	#NULL!
5.00	4.00	0.51	-0.07	0.00	0.00
4.00	#NULL!	#NULL!	#NULL!	#NULL!	#NULL!
5.00	#NULL!	#NULL!	#NULL!	#NULL!	#NULL!
3.00	0.00	1.11	0.92	0.00	0.00
5.00	#NULL!	#NULL!	#NULL!	#NULL!	#NULL!
4.00	#NULL!	#NULL!	#NULL!	#NULL!	#NULL!
#NULL!	4.00	-0.08	-1.23	0.00	0.00
4.00	4.00	1.50	0.75	0.00	0.00
5.00	#NULL!	#NULL!	#NULL!	#NULL!	#NULL!
4.00	1.00	-0.68	-0.41	0.00	0.00
4.00	#NULL!	#NULL!	#NULL!	#NULL!	#NULL!
4.00	#NULL!	#NULL!	#NULL!	#NULL!	#NULL!
5.00	4.00	1.11	0.26	1.00	35.00
4.00	1.00	0.12	1.42	0.00	0.00
5.00	1.00	1.11	-0.90	0.00	10.00
4.00	0.00	0.91	1.75	0.00	0.00
5.00	0.00	-0.48	-1.07	0.00	0.00
4.00	#NULL!	#NULL!	#NULL!	#NULL!	#NULL!
5.00	#NULL!	#NULL!	#NULL!	#NULL!	#NULL!
5.00	1.00	0.71	-1.23	0.00	0.00
#NULL!	#NULL!	#NULL!	#NULL!	#NULL!	#NULL!
5.00	#NULL!	#NULL!	#NULL!	#NULL!	#NULL!
4.00	0.25	-0.28	0.09	0.00	0.00
4.00	0.25	0.91	-0.24	0.00	0.00
4.00	#NULL!	#NULL!	#NULL!	#NULL!	#NULL!
3.00	10.00	0.71	0.26	0.00	0.00
5.00	#NULL!	-0.28	0.09	0.00	0.00
4.00	#NULL!	#NULL!	#NULL!	#NULL!	#NULL!
4.00	#NULL!	#NULL!	#NULL!	#NULL!	#NULL!
5.00	4.00	-0.88	-0.07	0.00	0.00
4.00	10.00	-0.48	1.75	0.00	0.00
5.00	0.00	0.31	0.92	0.00	0.00
4.00	#NULL!	#NULL!	#NULL!	#NULL!	#NULL!
5.00	#NULL!	#NULL!	#NULL!	#NULL!	#NULL!
4.00	#NULL!	#NULL!	#NULL!	#NULL!	#NULL!
5.00	0.25	-0.68	-1.56	0.00	0.00
3.00	#NULL!	#NULL!	#NULL!	#NULL!	#NULL!
4.00	4.00	0.71	-0.24	0.00	0.00
5.00	#NULL!	#NULL!	#NULL!	#NULL!	#NULL!
4.00	4.00	-1.67	-0.24	0.00	0.00
5.00	#NULL!	#NULL!	#NULL!	#NULL!	#NULL!
5.00	#NULL!	#NULL!	#NULL!	#NULL!	#NULL!
3.00	#NULL!	#NULL!	#NULL!	#NULL!	#NULL!
3.00	#NULL!	#NULL!	#NULL!	#NULL!	#NULL!

3.00	10.00	0.51	0.42	0.00	0.00
4.00	4.00	1.50	-0.41	0.00	16.00
4.00	0.25	0.91	-0.74	1.00	32.00
#NULL!	0.00	0.51	1.91	0.00	45.00
3.00	#NULL!	#NULL!	#NULL!	#NULL!	#NULL!
4.00	4.00	-0.08	-0.24	0.00	0.00
4.00	#NULL!	#NULL!	#NULL!	#NULL!	#NULL!
5.00	#NULL!	#NULL!	#NULL!	#NULL!	#NULL!
4.00	#NULL!	#NULL!	#NULL!	#NULL!	#NULL!
4.00	#NULL!	#NULL!	#NULL!	#NULL!	#NULL!
4.00	#NULL!	#NULL!	#NULL!	#NULL!	#NULL!
3.00	4.00	-1.67	-1.07	0.00	0.00
3.00	0.00	0.71	1.75	0.00	0.00
4.00	#NULL!	#NULL!	#NULL!	#NULL!	#NULL!
3.00	#NULL!	#NULL!	#NULL!	#NULL!	#NULL!
2.00	0.00	-3.65	0.09	0.00	5.00
4.00	0.00	-0.28	1.25	0.00	0.00
5.00	#NULL!	#NULL!	#NULL!	#NULL!	#NULL!
4.00	#NULL!	#NULL!	#NULL!	#NULL!	#NULL!
4.00	#NULL!	#NULL!	#NULL!	#NULL!	#NULL!
5.00	#NULL!	#NULL!	#NULL!	#NULL!	#NULL!
4.00	#NULL!	#NULL!	#NULL!	#NULL!	#NULL!
4.00	#NULL!	#NULL!	#NULL!	#NULL!	#NULL!
4.00	4.00	0.71	0.75	0.00	0.00
5.00	#NULL!	#NULL!	#NULL!	#NULL!	#NULL!
5.00	#NULL!	#NULL!	#NULL!	#NULL!	#NULL!
4.00	#NULL!	#NULL!	#NULL!	#NULL!	#NULL!
4.00	#NULL!	#NULL!	#NULL!	#NULL!	#NULL!
3.00	0.00	-1.87	0.42	0.00	0.00
4.00	#NULL!	#NULL!	#NULL!	#NULL!	#NULL!
5.00	#NULL!	#NULL!	#NULL!	#NULL!	#NULL!
3.00	#NULL!	#NULL!	#NULL!	#NULL!	#NULL!
4.00	#NULL!	#NULL!	#NULL!	#NULL!	#NULL!
5.00	#NULL!	#NULL!	#NULL!	#NULL!	#NULL!
5.00	10.00	-0.68	-0.90	1.00	0.00
4.00	#NULL!	#NULL!	#NULL!	#NULL!	#NULL!
4.00	0.25	0.51	0.26	0.00	0.00
5.00	20.00	-0.68	3.07	0.00	21.00
4.00	0.25	-1.07	2.91	0.00	0.00
5.00	0.00	-0.08	-0.07	0.00	0.00
3.00	#NULL!	#NULL!	#NULL!	#NULL!	#NULL!
4.00	#NULL!	#NULL!	#NULL!	#NULL!	#NULL!
5.00	#NULL!	#NULL!	#NULL!	#NULL!	#NULL!
5.00	#NULL!	#NULL!	#NULL!	#NULL!	#NULL!
4.00	#NULL!	1.50	0.75	0.00	22.00
4.00	#NULL!	#NULL!	#NULL!	#NULL!	#NULL!
4.00	#NULL!	#NULL!	#NULL!	#NULL!	#NULL!
4.00	0.25	-0.28	-0.74	0.00	0.00
5.00	4.00	0.12	-0.74	1.00	0.00
4.00	0.00	-0.08	0.75	0.00	12.00
4.00	#NULL!	#NULL!	#NULL!	#NULL!	#NULL!
4.00	4.00	0.91	0.92	0.00	12.00
3.00	#NULL!	#NULL!	#NULL!	#NULL!	#NULL!
2.00	1.00	-2.07	0.09	1.00	0.00
5.00	#NULL!	#NULL!	#NULL!	#NULL!	#NULL!
4.00	#NULL!	#NULL!	#NULL!	#NULL!	#NULL!

4.00	#NULL!	#NULL!	#NULL!	#NULL!	#NULL!
4.00	1.00	0.51	-0.74	0.00	0.00
5.00	4.00	-0.28	0.42	0.00	0.00
3.00	#NULL!	#NULL!	#NULL!	#NULL!	#NULL!
5.00	30.00	-0.68	0.42	0.00	0.00
4.00	0.00	0.31	-0.07	0.00	40.00
5.00	#NULL!	#NULL!	#NULL!	#NULL!	#NULL!
4.00	4.00	-0.28	-0.57	0.00	0.00
3.00	#NULL!	#NULL!	#NULL!	#NULL!	#NULL!
3.00	1.00	-0.28	0.59	0.00	0.00
5.00	#NULL!	#NULL!	#NULL!	#NULL!	#NULL!
5.00	1.00	-0.08	-0.41	0.00	0.00
4.00	#NULL!	#NULL!	#NULL!	#NULL!	#NULL!
4.00	#NULL!	#NULL!	#NULL!	#NULL!	#NULL!
4.00	#NULL!	#NULL!	#NULL!	#NULL!	#NULL!
3.00	#NULL!	#NULL!	#NULL!	#NULL!	#NULL!
3.00	4.00	0.91	2.41	0.00	0.00
4.00	#NULL!	#NULL!	#NULL!	#NULL!	#NULL!
3.00	#NULL!	#NULL!	#NULL!	#NULL!	#NULL!
5.00	#NULL!	#NULL!	#NULL!	#NULL!	#NULL!
4.00	#NULL!	#NULL!	#NULL!	#NULL!	#NULL!
4.00	#NULL!	#NULL!	#NULL!	#NULL!	#NULL!
5.00	#NULL!	#NULL!	#NULL!	#NULL!	#NULL!
4.00	0.00	0.71	0.09	0.00	0.00
4.00	#NULL!	#NULL!	#NULL!	#NULL!	#NULL!
4.00	10.00	-2.27	1.09	0.00	2.00
5.00	0.25	0.91	-0.57	0.00	0.00
4.00	10.00	-0.08	-0.07	1.00	28.00
4.00	#NULL!	#NULL!	#NULL!	#NULL!	#NULL!
3.00	#NULL!	#NULL!	#NULL!	#NULL!	#NULL!
4.00	10.00	-0.48	1.42	0.00	0.00
4.00	#NULL!	#NULL!	#NULL!	#NULL!	#NULL!
3.00	#NULL!	#NULL!	#NULL!	#NULL!	#NULL!
4.00	#NULL!	#NULL!	#NULL!	#NULL!	#NULL!
5.00	0.25	0.51	-0.90	0.00	3.00
4.00	#NULL!	#NULL!	#NULL!	#NULL!	#NULL!
4.00	#NULL!	#NULL!	#NULL!	#NULL!	#NULL!
4.00	#NULL!	#NULL!	#NULL!	#NULL!	#NULL!
4.00	#NULL!	#NULL!	#NULL!	#NULL!	#NULL!
5.00	#NULL!	#NULL!	#NULL!	#NULL!	#NULL!
4.00	#NULL!	#NULL!	#NULL!	#NULL!	#NULL!
4.00	#NULL!	-0.28	0.42	0.00	0.00
5.00	#NULL!	#NULL!	#NULL!	#NULL!	#NULL!
2.00	0.00	-0.68	-1.23	1.00	17.00
5.00	10.00	0.31	0.92	0.00	0.00
4.00	#NULL!	#NULL!	#NULL!	#NULL!	#NULL!
3.00	#NULL!	#NULL!	#NULL!	#NULL!	#NULL!
4.00	#NULL!	#NULL!	#NULL!	#NULL!	#NULL!
4.00	#NULL!	#NULL!	#NULL!	#NULL!	#NULL!
4.00	#NULL!	#NULL!	#NULL!	#NULL!	#NULL!
4.00	#NULL!	0.91	-1.23	0.00	26.00
5.00	#NULL!	#NULL!	#NULL!	#NULL!	#NULL!
4.00	#NULL!	#NULL!	#NULL!	#NULL!	#NULL!
3.00	#NULL!	#NULL!	#NULL!	#NULL!	#NULL!
3.00	#NULL!	#NULL!	#NULL!	#NULL!	#NULL!
5.00	0.25	-1.07	-1.07	0.00	0.00

5.00	20.00	0.91	-0.57	0.00	1.00
4.00	#NULL!	#NULL!	#NULL!	#NULL!	#NULL!
4.00	#NULL!	#NULL!	#NULL!	#NULL!	#NULL!
4.00	#NULL!	#NULL!	#NULL!	#NULL!	#NULL!
5.00	0.25	2.10	1.09	0.00	24.00
4.00	#NULL!	#NULL!	#NULL!	#NULL!	#NULL!
4.00	10.00	0.71	-1.23	0.00	18.00
3.00	#NULL!	#NULL!	#NULL!	#NULL!	#NULL!
4.00	#NULL!	#NULL!	#NULL!	#NULL!	#NULL!
#NULL!	#NULL!	#NULL!	#NULL!	#NULL!	#NULL!
#NULL!	#NULL!	#NULL!	#NULL!	#NULL!	#NULL!
#NULL!	#NULL!	#NULL!	#NULL!	#NULL!	#NULL!
#NULL!	#NULL!	#NULL!	#NULL!	#NULL!	#NULL!
#NULL!	#NULL!	#NULL!	#NULL!	#NULL!	#NULL!
#NULL!	#NULL!	#NULL!	#NULL!	#NULL!	#NULL!
#NULL!	#NULL!	#NULL!	#NULL!	#NULL!	#NULL!
#NULL!	#NULL!	#NULL!	#NULL!	#NULL!	#NULL!
#NULL!	#NULL!	#NULL!	#NULL!	#NULL!	#NULL!

physical_disease	physical_disease1	illness	illness1	lo_hi	SD_Z	HA_Z
4.00	3.00	1.00	1.00	1.00	1.29	-1.40
#NULL!	#NULL!	#NULL!	#NULL!	#NULL!	#NULL!	#NULL!
6.00	5.00	1.00	1.00	1.00	0.16	0.84
1.00	1.00	1.00	0.00	1.00	0.07	-0.37
2.00	2.00	1.00	1.00	0.00	1.11	-1.40
0.00	0.00	0.00	0.00	1.00	1.63	-1.58
1.00	1.00	1.00	0.00	1.00	1.63	-0.54
3.00	3.00	1.00	1.00	1.00	1.37	-1.32
#NULL!	#NULL!	#NULL!	#NULL!	#NULL!	#NULL!	#NULL!
2.00	2.00	1.00	1.00	1.00	0.42	-0.63
2.00	2.00	1.00	1.00	1.00	1.03	-0.02
#NULL!	#NULL!	#NULL!	#NULL!	#NULL!	#NULL!	#NULL!
#NULL!	#NULL!	#NULL!	#NULL!	#NULL!	#NULL!	#NULL!
#NULL!	#NULL!	#NULL!	#NULL!	#NULL!	#NULL!	#NULL!
4.00	3.00	1.00	1.00	1.00	-0.62	-0.28
#NULL!	#NULL!	#NULL!	#NULL!	#NULL!	#NULL!	#NULL!
#NULL!	#NULL!	#NULL!	#NULL!	#NULL!	#NULL!	#NULL!
3.00	3.00	1.00	1.00	0.00	1.63	1.01
1.00	1.00	1.00	0.00	0.00	-1.66	-0.28
1.00	1.00	1.00	0.00	0.00	-0.10	1.10
1.00	1.00	1.00	0.00	1.00	0.85	-2.01
#NULL!	#NULL!	#NULL!	#NULL!	#NULL!	#NULL!	#NULL!
#NULL!	#NULL!	#NULL!	#NULL!	#NULL!	#NULL!	#NULL!
3.00	3.00	1.00	1.00	1.00	0.51	-1.06
0.00	0.00	0.00	0.00	0.00	-0.01	0.15
#NULL!	#NULL!	#NULL!	#NULL!	#NULL!	#NULL!	#NULL!
#NULL!	#NULL!	#NULL!	#NULL!	#NULL!	#NULL!	#NULL!
#NULL!	#NULL!	#NULL!	#NULL!	#NULL!	#NULL!	#NULL!
#NULL!	#NULL!	#NULL!	#NULL!	#NULL!	#NULL!	#NULL!
#NULL!	#NULL!	#NULL!	#NULL!	#NULL!	#NULL!	#NULL!
#NULL!	#NULL!	#NULL!	#NULL!	#NULL!	#NULL!	#NULL!
1.00	1.00	1.00	0.00	1.00	1.63	-0.80
1.00	1.00	1.00	0.00	0.00	-0.62	1.45
#NULL!	#NULL!	#NULL!	#NULL!	#NULL!	#NULL!	#NULL!
#NULL!	#NULL!	#NULL!	#NULL!	#NULL!	#NULL!	#NULL!
#NULL!	#NULL!	#NULL!	#NULL!	#NULL!	#NULL!	#NULL!
#NULL!	#NULL!	#NULL!	#NULL!	#NULL!	#NULL!	#NULL!
#NULL!	#NULL!	#NULL!	#NULL!	#NULL!	#NULL!	#NULL!
#NULL!	#NULL!	#NULL!	#NULL!	#NULL!	#NULL!	#NULL!
#NULL!	#NULL!	#NULL!	#NULL!	#NULL!	#NULL!	#NULL!
#NULL!	#NULL!	#NULL!	#NULL!	#NULL!	#NULL!	#NULL!
1.00	1.00	1.00	0.00	1.00	0.25	-0.11
#NULL!	#NULL!	#NULL!	#NULL!	#NULL!	#NULL!	#NULL!
#NULL!	#NULL!	#NULL!	#NULL!	#NULL!	#NULL!	#NULL!
#NULL!	#NULL!	#NULL!	#NULL!	#NULL!	#NULL!	#NULL!
0.00	0.00	0.00	0.00	1.00	0.42	-2.01
2.00	2.00	1.00	1.00	1.00	1.37	-0.37
#NULL!	#NULL!	#NULL!	#NULL!	#NULL!	#NULL!	#NULL!
2.00	1.00	1.00	1.00	1.00	1.11	-1.66
#NULL!	#NULL!	#NULL!	#NULL!	#NULL!	#NULL!	#NULL!
1.00	1.00	1.00	0.00	1.00	0.68	-2.01
#NULL!	#NULL!	#NULL!	#NULL!	#NULL!	#NULL!	#NULL!
#NULL!	#NULL!	#NULL!	#NULL!	#NULL!	#NULL!	#NULL!
#NULL!	#NULL!	#NULL!	#NULL!	#NULL!	#NULL!	#NULL!
3.00	3.00	1.00	1.00	0.00	-0.01	0.24

3.00	3.00	1.00	1.00	0.00	0.59	0.75
#NULL!	#NULL!	#NULL!	#NULL!	#NULL!	#NULL!	#NULL!
1.00	1.00	1.00	0.00	0.00	0.77	-0.54
4.00	3.00	1.00	1.00	0.00	-0.10	0.75
3.00	3.00	1.00	1.00	0.00	0.94	0.06
#NULL!	#NULL!	#NULL!	#NULL!	#NULL!	#NULL!	#NULL!
#NULL!	#NULL!	#NULL!	#NULL!	#NULL!	#NULL!	#NULL!
1.00	1.00	1.00	0.00	1.00	0.25	-0.63
#NULL!	#NULL!	#NULL!	#NULL!	#NULL!	#NULL!	#NULL!
2.00	2.00	1.00	1.00	1.00	-0.19	1.45
1.00	1.00	1.00	0.00	0.00	-0.19	-1.92
3.00	3.00	1.00	1.00	1.00	0.77	0.67
#NULL!	#NULL!	#NULL!	#NULL!	#NULL!	#NULL!	#NULL!
#NULL!	#NULL!	#NULL!	#NULL!	#NULL!	#NULL!	#NULL!
1.00	1.00	1.00	0.00	0.00	-1.14	1.70
0.00	0.00	0.00	0.00	0.00	1.63	0.75
0.00	0.00	0.00	0.00	1.00	0.94	-0.11
#NULL!	#NULL!	#NULL!	#NULL!	#NULL!	#NULL!	#NULL!
2.00	2.00	1.00	1.00	0.00	-1.31	0.41
#NULL!	#NULL!	#NULL!	#NULL!	#NULL!	#NULL!	#NULL!
1.00	1.00	1.00	0.00	0.00	1.98	-1.40
#NULL!	#NULL!	#NULL!	#NULL!	#NULL!	#NULL!	#NULL!
#NULL!	#NULL!	#NULL!	#NULL!	#NULL!	#NULL!	#NULL!
0.00	0.00	0.00	0.00	0.00	-0.71	0.15
1.00	1.00	1.00	0.00	0.00	-0.19	1.36
#NULL!	#NULL!	#NULL!	#NULL!	#NULL!	#NULL!	#NULL!
4.00	4.00	1.00	1.00	1.00	-0.45	-0.02
#NULL!	#NULL!	#NULL!	#NULL!	#NULL!	#NULL!	#NULL!
#NULL!	#NULL!	#NULL!	#NULL!	#NULL!	#NULL!	#NULL!
1.00	1.00	1.00	0.00	1.00	1.46	-0.54
1.00	1.00	1.00	0.00	1.00	1.55	-1.66
0.00	0.00	0.00	0.00	0.00	0.07	0.06
#NULL!	#NULL!	#NULL!	#NULL!	#NULL!	#NULL!	#NULL!
#NULL!	#NULL!	#NULL!	#NULL!	#NULL!	#NULL!	#NULL!
1.00	1.00	1.00	0.00	1.00	0.68	-1.23
#NULL!	#NULL!	#NULL!	#NULL!	#NULL!	#NULL!	#NULL!
#NULL!	#NULL!	#NULL!	#NULL!	#NULL!	#NULL!	#NULL!
#NULL!	#NULL!	#NULL!	#NULL!	#NULL!	#NULL!	#NULL!
1.00	1.00	1.00	0.00	0.00	-2.79	2.65
2.00	1.00	1.00	1.00	0.00	0.77	0.06
#NULL!	#NULL!	#NULL!	#NULL!	#NULL!	#NULL!	#NULL!
#NULL!	#NULL!	#NULL!	#NULL!	#NULL!	#NULL!	#NULL!
#NULL!	#NULL!	#NULL!	#NULL!	#NULL!	#NULL!	#NULL!
#NULL!	#NULL!	#NULL!	#NULL!	#NULL!	#NULL!	#NULL!
4.00	3.00	1.00	1.00	0.00	-0.71	0.06
2.00	2.00	1.00	1.00	1.00	0.51	-0.71
2.00	2.00	1.00	1.00	0.00	-0.10	1.36
#NULL!	#NULL!	#NULL!	#NULL!	#NULL!	#NULL!	#NULL!
#NULL!	#NULL!	#NULL!	#NULL!	#NULL!	#NULL!	#NULL!
#NULL!	#NULL!	#NULL!	#NULL!	#NULL!	#NULL!	#NULL!
#NULL!	#NULL!	#NULL!	#NULL!	#NULL!	#NULL!	#NULL!
#NULL!	#NULL!	#NULL!	#NULL!	#NULL!	#NULL!	#NULL!
0.00	0.00	0.00	0.00	1.00	0.77	-0.45
#NULL!	#NULL!	#NULL!	#NULL!	#NULL!	#NULL!	#NULL!
#NULL!	#NULL!	#NULL!	#NULL!	#NULL!	#NULL!	#NULL!
2.00	1.00	1.00	1.00	0.00	-0.71	0.24

#NULL!	#NULL!	#NULL!	#NULL!	#NULL!	#NULL!	#NULL!
1.00	1.00	1.00	0.00	1.00	0.94	-0.63
#NULL!	#NULL!	#NULL!	#NULL!	#NULL!	#NULL!	#NULL!
1.00	1.00	1.00	0.00	0.00	0.42	-0.02
#NULL!	#NULL!	#NULL!	#NULL!	#NULL!	#NULL!	#NULL!
#NULL!	#NULL!	#NULL!	#NULL!	#NULL!	#NULL!	#NULL!
#NULL!	#NULL!	#NULL!	#NULL!	#NULL!	#NULL!	#NULL!
#NULL!	#NULL!	#NULL!	#NULL!	#NULL!	#NULL!	#NULL!
#NULL!	#NULL!	#NULL!	#NULL!	#NULL!	#NULL!	#NULL!
0.00	0.00	0.00	0.00	0.00	0.25	0.67
#NULL!	#NULL!	#NULL!	#NULL!	#NULL!	#NULL!	#NULL!
#NULL!	#NULL!	#NULL!	#NULL!	#NULL!	#NULL!	#NULL!
1.00	1.00	1.00	0.00	1.00	-0.19	-0.45
#NULL!	#NULL!	#NULL!	#NULL!	#NULL!	#NULL!	#NULL!
#NULL!	#NULL!	#NULL!	#NULL!	#NULL!	#NULL!	#NULL!
4.00	3.00	1.00	1.00	1.00	-0.88	1.27
#NULL!	#NULL!	#NULL!	#NULL!	#NULL!	#NULL!	#NULL!
#NULL!	#NULL!	#NULL!	#NULL!	#NULL!	#NULL!	#NULL!
2.00	2.00	1.00	1.00	1.00	-0.19	-0.37
0.00	0.00	0.00	0.00	1.00	-0.27	-0.28
#NULL!	#NULL!	#NULL!	#NULL!	#NULL!	#NULL!	#NULL!
1.00	1.00	1.00	0.00	0.00	0.42	0.58
#NULL!	#NULL!	#NULL!	#NULL!	#NULL!	#NULL!	#NULL!
#NULL!	#NULL!	#NULL!	#NULL!	#NULL!	#NULL!	#NULL!
1.00	0.00	0.00	0.00	1.00	0.42	1.01
0.00	0.00	0.00	0.00	0.00	-0.97	0.93
0.00	0.00	0.00	0.00	0.00	2.07	-1.49
1.00	1.00	1.00	0.00	1.00	-1.05	0.41
2.00	1.00	1.00	1.00	0.00	1.03	0.06
#NULL!	#NULL!	#NULL!	#NULL!	#NULL!	#NULL!	#NULL!
#NULL!	#NULL!	#NULL!	#NULL!	#NULL!	#NULL!	#NULL!
1.00	1.00	1.00	0.00	1.00	2.24	-2.35
#NULL!	#NULL!	#NULL!	#NULL!	#NULL!	#NULL!	#NULL!
#NULL!	#NULL!	#NULL!	#NULL!	#NULL!	#NULL!	#NULL!
1.00	1.00	1.00	0.00	1.00	-0.62	1.10
2.00	2.00	1.00	1.00	1.00	1.63	-0.37
#NULL!	#NULL!	#NULL!	#NULL!	#NULL!	#NULL!	#NULL!
1.00	1.00	1.00	0.00	0.00	-0.71	1.19
1.00	1.00	1.00	0.00	0.00	0.25	0.32
#NULL!	#NULL!	#NULL!	#NULL!	#NULL!	#NULL!	#NULL!
#NULL!	#NULL!	#NULL!	#NULL!	#NULL!	#NULL!	#NULL!
1.00	1.00	1.00	0.00	0.00	-0.71	1.36
0.00	0.00	0.00	0.00	0.00	-2.09	0.58
0.00	0.00	0.00	0.00	0.00	0.25	0.93
#NULL!	#NULL!	#NULL!	#NULL!	#NULL!	#NULL!	#NULL!
#NULL!	#NULL!	#NULL!	#NULL!	#NULL!	#NULL!	#NULL!
#NULL!	#NULL!	#NULL!	#NULL!	#NULL!	#NULL!	#NULL!
1.00	1.00	1.00	0.00	0.00	0.68	-0.63
#NULL!	#NULL!	#NULL!	#NULL!	#NULL!	#NULL!	#NULL!
2.00	1.00	1.00	1.00	1.00	0.33	-0.97
#NULL!	#NULL!	#NULL!	#NULL!	#NULL!	#NULL!	#NULL!
1.00	1.00	1.00	0.00	0.00	-0.79	0.41
#NULL!	#NULL!	#NULL!	#NULL!	#NULL!	#NULL!	#NULL!
#NULL!	#NULL!	#NULL!	#NULL!	#NULL!	#NULL!	#NULL!
#NULL!	#NULL!	#NULL!	#NULL!	#NULL!	#NULL!	#NULL!
#NULL!	#NULL!	#NULL!	#NULL!	#NULL!	#NULL!	#NULL!

0.00	0.00	0.00	0.00	0.00	-0.88	0.41
1.00	1.00	1.00	0.00	0.00	1.03	-0.88
2.00	1.00	1.00	1.00	0.00	0.94	-1.66
3.00	3.00	1.00	1.00	1.00	-1.14	1.79
#NULL!	#NULL!	#NULL!	#NULL!	#NULL!	#NULL!	#NULL!
2.00	2.00	1.00	1.00	1.00	-0.10	0.06
#NULL!	#NULL!	#NULL!	#NULL!	#NULL!	#NULL!	#NULL!
#NULL!	#NULL!	#NULL!	#NULL!	#NULL!	#NULL!	#NULL!
#NULL!	#NULL!	#NULL!	#NULL!	#NULL!	#NULL!	#NULL!
#NULL!	#NULL!	#NULL!	#NULL!	#NULL!	#NULL!	#NULL!
#NULL!	#NULL!	#NULL!	#NULL!	#NULL!	#NULL!	#NULL!
1.00	1.00	1.00	0.00	0.00	-1.23	-0.19
0.00	0.00	0.00	0.00	0.00	0.33	0.93
#NULL!	#NULL!	#NULL!	#NULL!	#NULL!	#NULL!	#NULL!
#NULL!	#NULL!	#NULL!	#NULL!	#NULL!	#NULL!	#NULL!
4.00	4.00	1.00	1.00	0.00	-3.05	1.70
2.00	2.00	1.00	1.00	1.00	-0.97	0.84
#NULL!	#NULL!	#NULL!	#NULL!	#NULL!	#NULL!	#NULL!
#NULL!	#NULL!	#NULL!	#NULL!	#NULL!	#NULL!	#NULL!
#NULL!	#NULL!	#NULL!	#NULL!	#NULL!	#NULL!	#NULL!
#NULL!	#NULL!	#NULL!	#NULL!	#NULL!	#NULL!	#NULL!
#NULL!	#NULL!	#NULL!	#NULL!	#NULL!	#NULL!	#NULL!
#NULL!	#NULL!	#NULL!	#NULL!	#NULL!	#NULL!	#NULL!
2.00	2.00	1.00	1.00	1.00	0.51	0.75
#NULL!	#NULL!	#NULL!	#NULL!	#NULL!	#NULL!	#NULL!
#NULL!	#NULL!	#NULL!	#NULL!	#NULL!	#NULL!	#NULL!
#NULL!	#NULL!	#NULL!	#NULL!	#NULL!	#NULL!	#NULL!
#NULL!	#NULL!	#NULL!	#NULL!	#NULL!	#NULL!	#NULL!
1.00	1.00	1.00	0.00	0.00	-0.01	1.96
#NULL!	#NULL!	#NULL!	#NULL!	#NULL!	#NULL!	#NULL!
#NULL!	#NULL!	#NULL!	#NULL!	#NULL!	#NULL!	#NULL!
#NULL!	#NULL!	#NULL!	#NULL!	#NULL!	#NULL!	#NULL!
#NULL!	#NULL!	#NULL!	#NULL!	#NULL!	#NULL!	#NULL!
#NULL!	#NULL!	#NULL!	#NULL!	#NULL!	#NULL!	#NULL!
1.00	1.00	1.00	0.00	0.00	-1.49	0.15
#NULL!	#NULL!	#NULL!	#NULL!	#NULL!	#NULL!	#NULL!
2.00	2.00	1.00	1.00	1.00	-0.36	0.24
0.00	0.00	0.00	0.00	0.00	-3.31	1.19
2.00	2.00	1.00	1.00	0.00	-1.40	1.36
1.00	1.00	1.00	0.00	0.00	0.07	-0.54
#NULL!	#NULL!	#NULL!	#NULL!	#NULL!	#NULL!	#NULL!
#NULL!	#NULL!	#NULL!	#NULL!	#NULL!	#NULL!	#NULL!
#NULL!	#NULL!	#NULL!	#NULL!	#NULL!	#NULL!	#NULL!
#NULL!	#NULL!	#NULL!	#NULL!	#NULL!	#NULL!	#NULL!
1.00	1.00	1.00	0.00	0.00	-0.27	0.06
#NULL!	#NULL!	#NULL!	#NULL!	#NULL!	#NULL!	#NULL!
#NULL!	#NULL!	#NULL!	#NULL!	#NULL!	#NULL!	#NULL!
2.00	2.00	1.00	1.00	1.00	0.07	0.50
1.00	1.00	1.00	0.00	1.00	0.51	-0.71
1.00	1.00	1.00	0.00	0.00	-1.05	1.01
#NULL!	#NULL!	#NULL!	#NULL!	#NULL!	#NULL!	#NULL!
1.00	1.00	1.00	0.00	1.00	-1.31	-0.02
#NULL!	#NULL!	#NULL!	#NULL!	#NULL!	#NULL!	#NULL!
4.00	3.00	1.00	1.00	0.00	-2.01	1.70
#NULL!	#NULL!	#NULL!	#NULL!	#NULL!	#NULL!	#NULL!
#NULL!	#NULL!	#NULL!	#NULL!	#NULL!	#NULL!	#NULL!

#NULL!	#NULL!	#NULL!	#NULL!	#NULL!	#NULL!	#NULL!
2.00	2.00	1.00	1.00	1.00	0.51	-0.97
0.00	0.00	0.00	0.00	1.00	-0.36	0.15
#NULL!	#NULL!	#NULL!	#NULL!	#NULL!	#NULL!	#NULL!
1.00	1.00	1.00	0.00	0.00	-0.97	0.93
2.00	2.00	1.00	1.00	0.00	0.42	0.50
#NULL!	#NULL!	#NULL!	#NULL!	#NULL!	#NULL!	#NULL!
1.00	1.00	1.00	0.00	1.00	0.85	-1.23
#NULL!	#NULL!	#NULL!	#NULL!	#NULL!	#NULL!	#NULL!
3.00	2.00	1.00	1.00	0.00	-0.45	0.93
#NULL!	#NULL!	#NULL!	#NULL!	#NULL!	#NULL!	#NULL!
1.00	1.00	1.00	0.00	1.00	0.68	0.06
#NULL!	#NULL!	#NULL!	#NULL!	#NULL!	#NULL!	#NULL!
#NULL!	#NULL!	#NULL!	#NULL!	#NULL!	#NULL!	#NULL!
#NULL!	#NULL!	#NULL!	#NULL!	#NULL!	#NULL!	#NULL!
#NULL!	#NULL!	#NULL!	#NULL!	#NULL!	#NULL!	#NULL!
2.00	1.00	1.00	1.00	0.00	-1.92	1.45
#NULL!	#NULL!	#NULL!	#NULL!	#NULL!	#NULL!	#NULL!
#NULL!	#NULL!	#NULL!	#NULL!	#NULL!	#NULL!	#NULL!
#NULL!	#NULL!	#NULL!	#NULL!	#NULL!	#NULL!	#NULL!
#NULL!	#NULL!	#NULL!	#NULL!	#NULL!	#NULL!	#NULL!
#NULL!	#NULL!	#NULL!	#NULL!	#NULL!	#NULL!	#NULL!
#NULL!	#NULL!	#NULL!	#NULL!	#NULL!	#NULL!	#NULL!
2.00	2.00	1.00	1.00	0.00	-1.66	-1.14
#NULL!	#NULL!	#NULL!	#NULL!	#NULL!	#NULL!	#NULL!
0.00	0.00	0.00	0.00	0.00	-1.83	2.05
0.00	0.00	0.00	0.00	0.00	0.16	0.32
1.00	1.00	1.00	0.00	1.00	0.68	0.84
#NULL!	#NULL!	#NULL!	#NULL!	#NULL!	#NULL!	#NULL!
#NULL!	#NULL!	#NULL!	#NULL!	#NULL!	#NULL!	#NULL!
1.00	1.00	1.00	0.00	0.00	-1.14	1.79
#NULL!	#NULL!	#NULL!	#NULL!	#NULL!	#NULL!	#NULL!
#NULL!	#NULL!	#NULL!	#NULL!	#NULL!	#NULL!	#NULL!
#NULL!	#NULL!	#NULL!	#NULL!	#NULL!	#NULL!	#NULL!
0.00	0.00	0.00	0.00	1.00	0.59	-0.19
#NULL!	#NULL!	#NULL!	#NULL!	#NULL!	#NULL!	#NULL!
#NULL!	#NULL!	#NULL!	#NULL!	#NULL!	#NULL!	#NULL!
#NULL!	#NULL!	#NULL!	#NULL!	#NULL!	#NULL!	#NULL!
#NULL!	#NULL!	#NULL!	#NULL!	#NULL!	#NULL!	#NULL!
#NULL!	#NULL!	#NULL!	#NULL!	#NULL!	#NULL!	#NULL!
#NULL!	#NULL!	#NULL!	#NULL!	#NULL!	#NULL!	#NULL!
1.00	1.00	1.00	0.00	0.00	0.16	0.50
#NULL!	#NULL!	#NULL!	#NULL!	#NULL!	#NULL!	#NULL!
2.00	2.00	1.00	1.00	1.00	0.51	-0.88
2.00	2.00	1.00	1.00	1.00	0.59	-0.37
#NULL!	#NULL!	#NULL!	#NULL!	#NULL!	#NULL!	#NULL!
#NULL!	#NULL!	#NULL!	#NULL!	#NULL!	#NULL!	#NULL!
#NULL!	#NULL!	#NULL!	#NULL!	#NULL!	#NULL!	#NULL!
#NULL!	#NULL!	#NULL!	#NULL!	#NULL!	#NULL!	#NULL!
#NULL!	#NULL!	#NULL!	#NULL!	#NULL!	#NULL!	#NULL!
1.00	1.00	1.00	0.00	0.00	0.33	-0.88
#NULL!	#NULL!	#NULL!	#NULL!	#NULL!	#NULL!	#NULL!
#NULL!	#NULL!	#NULL!	#NULL!	#NULL!	#NULL!	#NULL!
#NULL!	#NULL!	#NULL!	#NULL!	#NULL!	#NULL!	#NULL!
#NULL!	#NULL!	#NULL!	#NULL!	#NULL!	#NULL!	#NULL!
1.00	1.00	1.00	0.00	0.00	0.77	0.41

1.00	1.00	1.00	0.00	0.00	0.42	-1.23
#NULL!	#NULL!	#NULL!	#NULL!	#NULL!	#NULL!	#NULL!
#NULL!	#NULL!	#NULL!	#NULL!	#NULL!	#NULL!	#NULL!
#NULL!	#NULL!	#NULL!	#NULL!	#NULL!	#NULL!	#NULL!
1.00	1.00	1.00	0.00	1.00	-0.88	-0.19
#NULL!	#NULL!	#NULL!	#NULL!	#NULL!	#NULL!	#NULL!
4.00	4.00	1.00	1.00	1.00	1.46	-1.40
#NULL!	#NULL!	#NULL!	#NULL!	#NULL!	#NULL!	#NULL!
#NULL!	#NULL!	#NULL!	#NULL!	#NULL!	#NULL!	#NULL!
#NULL!	#NULL!	#NULL!	#NULL!	#NULL!	#NULL!	#NULL!
#NULL!	#NULL!	#NULL!	#NULL!	#NULL!	#NULL!	#NULL!
#NULL!	#NULL!	#NULL!	#NULL!	#NULL!	#NULL!	#NULL!
#NULL!	#NULL!	#NULL!	#NULL!	#NULL!	#NULL!	#NULL!
#NULL!	#NULL!	#NULL!	#NULL!	#NULL!	#NULL!	#NULL!
#NULL!	#NULL!	#NULL!	#NULL!	#NULL!	#NULL!	#NULL!
#NULL!	#NULL!	#NULL!	#NULL!	#NULL!	#NULL!	#NULL!
#NULL!	#NULL!	#NULL!	#NULL!	#NULL!	#NULL!	#NULL!
#NULL!	#NULL!	#NULL!	#NULL!	#NULL!	#NULL!	#NULL!

COOP_Z	bmi_z	whr_z	pas_z	sex_z	tas_z	ZNS	ZHA
0.01	-0.41	0.25	0.31	1.23	-0.29	0.93847	-1.39423
#NULL!	#NULL!	#NULL!	#NULL!	#NULL!	#NULL!	#NULL!	#NULL!
0.49	1.32	-0.69	-0.57	-0.55	-0.29	-0.48590	0.84215
-0.94	-0.26	0.55	2.93	-0.70	-0.70	-0.91322	-0.36205
-0.11	0.97	0.27	-1.01	-0.75	0.74	-2.19515	-1.39423
1.44	-0.21	-0.22	1.18	0.04	-1.22	-0.34347	-1.56626
0.73	-1.24	-0.46	-0.57	0.04	-1.01	-0.62834	-0.53408
-0.94	-0.43	0.32	-0.57	#NULL!	-0.70	0.22628	-1.30821
#NULL!	#NULL!	#NULL!	#NULL!	#NULL!	#NULL!	#NULL!	#NULL!
0.13	0.03	-0.30	2.49	-0.75	-1.11	-1.62540	-0.62010
0.97	0.78	-0.71	0.31	0.04	-1.32	-0.48590	-0.01800
#NULL!	#NULL!	#NULL!	#NULL!	#NULL!	#NULL!	#NULL!	#NULL!
#NULL!	#NULL!	#NULL!	#NULL!	#NULL!	#NULL!	#NULL!	#NULL!
#NULL!	#NULL!	#NULL!	#NULL!	#NULL!	#NULL!	#NULL!	#NULL!
-1.18	-0.09	0.67	-0.13	0.04	0.74	-0.34347	-0.27604
#NULL!	#NULL!	#NULL!	#NULL!	#NULL!	#NULL!	#NULL!	#NULL!
#NULL!	#NULL!	#NULL!	#NULL!	#NULL!	#NULL!	#NULL!	#NULL!
0.97	-0.17	-0.71	-0.13	1.23	0.12	-1.48296	1.01418
-1.41	0.04	0.39	-0.13	-0.55	-0.29	-0.05859	-0.27604
0.61	-0.86	-0.54	-1.01	1.23	2.70	-0.62834	1.10019
-0.11	0.18	-0.25	0.31	-0.55	-0.80	-0.34347	-1.99633
#NULL!	#NULL!	#NULL!	#NULL!	#NULL!	#NULL!	#NULL!	#NULL!
#NULL!	#NULL!	#NULL!	#NULL!	#NULL!	#NULL!	#NULL!	#NULL!
-0.70	-0.39	0.36	-0.57	-0.55	0.95	1.08091	-1.05017
0.37	-0.67	-0.82	-1.01	0.04	-0.19	1.93553	0.15403
#NULL!	#NULL!	#NULL!	#NULL!	#NULL!	#NULL!	#NULL!	#NULL!
#NULL!	#NULL!	#NULL!	#NULL!	#NULL!	#NULL!	#NULL!	#NULL!
#NULL!	#NULL!	#NULL!	#NULL!	#NULL!	#NULL!	#NULL!	#NULL!
#NULL!	#NULL!	#NULL!	#NULL!	#NULL!	#NULL!	#NULL!	#NULL!
#NULL!	#NULL!	#NULL!	#NULL!	#NULL!	#NULL!	#NULL!	#NULL!
#NULL!	#NULL!	#NULL!	#NULL!	#NULL!	#NULL!	#NULL!	#NULL!
1.08	-1.01	0.03	0.74	1.23	-0.60	0.79603	-0.79213
-0.70	-0.72	-0.55	-0.57	-0.70	-0.19	1.08091	1.44425
#NULL!	#NULL!	#NULL!	#NULL!	#NULL!	#NULL!	#NULL!	#NULL!
#NULL!	#NULL!	#NULL!	#NULL!	#NULL!	#NULL!	#NULL!	#NULL!
#NULL!	#NULL!	#NULL!	#NULL!	#NULL!	#NULL!	#NULL!	#NULL!
#NULL!	#NULL!	#NULL!	#NULL!	#NULL!	#NULL!	#NULL!	#NULL!
#NULL!	#NULL!	#NULL!	#NULL!	#NULL!	#NULL!	#NULL!	#NULL!
#NULL!	#NULL!	#NULL!	#NULL!	#NULL!	#NULL!	#NULL!	#NULL!
#NULL!	#NULL!	#NULL!	#NULL!	#NULL!	#NULL!	#NULL!	#NULL!
#NULL!	#NULL!	#NULL!	#NULL!	#NULL!	#NULL!	#NULL!	#NULL!
-0.11	-0.52	-0.65	-0.13	-0.75	-0.91	0.93847	-0.10401
#NULL!	#NULL!	#NULL!	#NULL!	#NULL!	#NULL!	#NULL!	#NULL!
#NULL!	#NULL!	#NULL!	#NULL!	#NULL!	#NULL!	#NULL!	#NULL!
#NULL!	#NULL!	#NULL!	#NULL!	#NULL!	#NULL!	#NULL!	#NULL!
-1.06	0.26	#NULL!	2.06	1.23	-0.80	0.08385	-1.99633
0.97	-0.68	-0.87	1.18	1.23	-0.70	-0.34347	-0.36205
#NULL!	#NULL!	#NULL!	#NULL!	#NULL!	#NULL!	#NULL!	#NULL!
0.13	-0.67	-0.07	1.62	0.04	-0.70	-0.48590	-1.65227
#NULL!	#NULL!	#NULL!	#NULL!	#NULL!	#NULL!	#NULL!	#NULL!
1.92	-0.43	-0.04	-0.57	0.04	-1.53	-1.05565	-1.99633
#NULL!	#NULL!	#NULL!	#NULL!	#NULL!	#NULL!	#NULL!	#NULL!
#NULL!	#NULL!	#NULL!	#NULL!	#NULL!	#NULL!	#NULL!	#NULL!
#NULL!	#NULL!	#NULL!	#NULL!	#NULL!	#NULL!	#NULL!	#NULL!
-0.11	0.54	-0.56	-1.01	-0.75	-0.39	-0.48590	0.24005

-0.11	-0.28	-0.62	-0.13	0.04	0.95	-1.05565	0.75614
#NULL!	#NULL!	#NULL!	#NULL!	#NULL!	#NULL!	#NULL!	#NULL!
-0.11	5.21	0.16	-0.57	0.04	-0.29	0.08385	-0.53408
0.49	-0.56	0.55	0.31	#NULL!	-1.01	0.08385	0.75614
0.37	-0.22	-0.72	-1.01	-0.75	-0.39	0.79603	0.06802
#NULL!	#NULL!	#NULL!	#NULL!	#NULL!	#NULL!	#NULL!	#NULL!
#NULL!	#NULL!	#NULL!	#NULL!	#NULL!	#NULL!	#NULL!	#NULL!
-0.34	-0.50	0.38	-0.13	1.23	-0.19	0.08385	-0.62010
#NULL!	#NULL!	#NULL!	#NULL!	#NULL!	#NULL!	#NULL!	#NULL!
-1.77	-1.15	0.22	2.93	-0.75	1.98	-0.48590	1.44425
-1.53	-0.31	0.31	2.93	1.23	-0.70	1.79309	-1.91032
-0.58	-0.07	-0.29	0.31	1.23	-0.91	-1.48296	0.67012
#NULL!	#NULL!	#NULL!	#NULL!	#NULL!	#NULL!	#NULL!	#NULL!
#NULL!	#NULL!	#NULL!	#NULL!	#NULL!	#NULL!	#NULL!	#NULL!
-0.46	-1.12	-0.84	0.74	0.04	0.64	0.22628	1.70230
0.85	-0.06	-0.40	-0.57	0.04	0.74	1.65066	0.75614
0.85	-0.99	0.00	-1.01	1.23	-0.80	1.22334	-0.10401
#NULL!	#NULL!	#NULL!	#NULL!	#NULL!	#NULL!	#NULL!	#NULL!
0.61	-0.89	0.11	1.62	-0.55	-0.29	-0.48590	0.41208
#NULL!	#NULL!	#NULL!	#NULL!	#NULL!	#NULL!	#NULL!	#NULL!
2.04	-0.16	0.14	0.74	1.23	-0.49	0.22628	-1.39423
#NULL!	#NULL!	#NULL!	#NULL!	#NULL!	#NULL!	#NULL!	#NULL!
#NULL!	#NULL!	#NULL!	#NULL!	#NULL!	#NULL!	#NULL!	#NULL!
0.13	0.23	0.20	-1.01	-0.55	1.05	0.65360	0.15403
-0.22	0.81	-0.37	0.31	0.04	1.67	-1.05565	1.35824
#NULL!	#NULL!	#NULL!	#NULL!	#NULL!	#NULL!	#NULL!	#NULL!
-0.11	0.86	0.55	-0.57	-0.55	0.02	0.08385	-0.01800
#NULL!	#NULL!	#NULL!	#NULL!	#NULL!	#NULL!	#NULL!	#NULL!
#NULL!	#NULL!	#NULL!	#NULL!	#NULL!	#NULL!	#NULL!	#NULL!
1.80	0.04	-0.18	-0.57	0.04	-1.63	-0.62834	-0.53408
0.97	0.27	0.75	0.74	-0.55	-1.11	0.36872	-1.65227
1.56	-0.44	-0.26	0.74	-0.55	-1.22	-0.77078	0.06802
#NULL!	#NULL!	#NULL!	#NULL!	#NULL!	#NULL!	#NULL!	#NULL!
#NULL!	#NULL!	#NULL!	#NULL!	#NULL!	#NULL!	#NULL!	#NULL!
1.32	-0.29	0.43	-0.57	1.23	-0.91	0.08385	-1.22220
#NULL!	#NULL!	#NULL!	#NULL!	#NULL!	#NULL!	#NULL!	#NULL!
#NULL!	#NULL!	#NULL!	#NULL!	#NULL!	#NULL!	#NULL!	#NULL!
#NULL!	#NULL!	#NULL!	#NULL!	#NULL!	#NULL!	#NULL!	#NULL!
-1.41	-0.79	-0.17	-0.13	0.04	0.64	-2.90734	2.64846
-0.34	-0.50	0.68	1.18	-0.70	-0.08	-0.05859	0.06802
#NULL!	#NULL!	#NULL!	#NULL!	#NULL!	#NULL!	#NULL!	#NULL!
#NULL!	#NULL!	#NULL!	#NULL!	#NULL!	#NULL!	#NULL!	#NULL!
#NULL!	#NULL!	#NULL!	#NULL!	#NULL!	#NULL!	#NULL!	#NULL!
#NULL!	#NULL!	#NULL!	#NULL!	#NULL!	#NULL!	#NULL!	#NULL!
-1.53	1.92	-0.66	-1.01	-0.75	0.95	-0.05859	0.06802
0.37	0.88	0.06	0.74	-0.75	-0.29	0.93847	-0.70611
-0.34	-0.43	-0.81	-0.13	-0.70	0.12	-0.77078	1.35824
#NULL!	#NULL!	#NULL!	#NULL!	#NULL!	#NULL!	#NULL!	#NULL!
#NULL!	#NULL!	#NULL!	#NULL!	#NULL!	#NULL!	#NULL!	#NULL!
#NULL!	#NULL!	#NULL!	#NULL!	#NULL!	#NULL!	#NULL!	#NULL!
#NULL!	#NULL!	#NULL!	#NULL!	#NULL!	#NULL!	#NULL!	#NULL!
#NULL!	#NULL!	#NULL!	#NULL!	#NULL!	#NULL!	#NULL!	#NULL!
1.32	-1.21	-0.28	1.18	1.23	0.23	-0.05859	-0.44807
#NULL!	#NULL!	#NULL!	#NULL!	#NULL!	#NULL!	#NULL!	#NULL!
#NULL!	#NULL!	#NULL!	#NULL!	#NULL!	#NULL!	#NULL!	#NULL!
0.37	0.04	-0.44	-0.13	1.23	-0.49	-0.05859	0.24005

#NULL!	#NULL!	#NULL!	#NULL!	#NULL!	#NULL!	#NULL!	#NULL!
0.13	-0.46	0.12	1.62	-0.70	-0.19	-0.91322	-0.62010
#NULL!	#NULL!	#NULL!	#NULL!	#NULL!	#NULL!	#NULL!	#NULL!
-1.18	-0.61	-0.01	-1.01	-0.55	0.23	-1.76784	-0.01800
#NULL!	#NULL!	#NULL!	#NULL!	#NULL!	#NULL!	#NULL!	#NULL!
#NULL!	#NULL!	#NULL!	#NULL!	#NULL!	#NULL!	#NULL!	#NULL!
#NULL!	#NULL!	#NULL!	#NULL!	#NULL!	#NULL!	#NULL!	#NULL!
#NULL!	#NULL!	#NULL!	#NULL!	#NULL!	#NULL!	#NULL!	#NULL!
#NULL!	#NULL!	#NULL!	#NULL!	#NULL!	#NULL!	#NULL!	#NULL!
0.25	-0.75	-0.88	-0.57	-0.70	-0.08	-0.34347	0.67012
#NULL!	#NULL!	#NULL!	#NULL!	#NULL!	#NULL!	#NULL!	#NULL!
#NULL!	#NULL!	#NULL!	#NULL!	#NULL!	#NULL!	#NULL!	#NULL!
-1.06	-0.36	0.32	0.31	0.04	0.95	-1.19809	-0.44807
#NULL!	#NULL!	#NULL!	#NULL!	#NULL!	#NULL!	#NULL!	#NULL!
#NULL!	#NULL!	#NULL!	#NULL!	#NULL!	#NULL!	#NULL!	#NULL!
-1.53	1.95	1.18	-1.01	-0.75	1.78	0.22628	1.27222
#NULL!	#NULL!	#NULL!	#NULL!	#NULL!	#NULL!	#NULL!	#NULL!
#NULL!	#NULL!	#NULL!	#NULL!	#NULL!	#NULL!	#NULL!	#NULL!
0.01	-0.57	0.10	0.74	0.04	-0.60	-0.77078	-0.36205
-0.82	-0.18	-0.69	0.74	0.04	0.43	0.79603	-0.27604
#NULL!	#NULL!	#NULL!	#NULL!	#NULL!	#NULL!	#NULL!	#NULL!
-0.70	-0.37	0.55	-1.01	-0.55	-1.01	-0.62834	0.58411
#NULL!	#NULL!	#NULL!	#NULL!	#NULL!	#NULL!	#NULL!	#NULL!
#NULL!	#NULL!	#NULL!	#NULL!	#NULL!	#NULL!	#NULL!	#NULL!
0.73	-0.67	-0.49	2.06	0.04	0.54	0.51116	1.01418
0.13	-0.76	-0.23	-1.01	-0.55	0.02	0.79603	0.92816
2.27	-0.34	5.78	2.06	-0.55	-1.63	1.93553	-1.48024
1.20	0.69	-0.04	-1.01	-0.75	-1.11	0.08385	0.41208
-0.46	0.35	-0.36	-0.57	-0.75	-0.91	1.08091	0.06802
#NULL!	#NULL!	#NULL!	#NULL!	#NULL!	#NULL!	#NULL!	#NULL!
#NULL!	#NULL!	#NULL!	#NULL!	#NULL!	#NULL!	#NULL!	#NULL!
0.73	0.44	-0.27	-1.01	-0.55	0.23	-0.34347	-2.34039
#NULL!	#NULL!	#NULL!	#NULL!	#NULL!	#NULL!	#NULL!	#NULL!
#NULL!	#NULL!	#NULL!	#NULL!	#NULL!	#NULL!	#NULL!	#NULL!
-0.46	-0.07	0.31	1.18	-0.70	1.57	0.36872	1.10019
0.73	-0.46	-0.42	1.18	-0.70	0.23	-0.91322	-0.36205
#NULL!	#NULL!	#NULL!	#NULL!	#NULL!	#NULL!	#NULL!	#NULL!
0.13	-1.15	-0.50	-0.13	1.23	-0.91	-0.62834	1.18621
-0.11	-0.39	0.49	0.31	#NULL!	0.23	-1.19809	0.32606
#NULL!	#NULL!	#NULL!	#NULL!	#NULL!	#NULL!	#NULL!	#NULL!
#NULL!	#NULL!	#NULL!	#NULL!	#NULL!	#NULL!	#NULL!	#NULL!
-1.29	-1.59	-1.02	-1.01	0.04	-1.22	0.65360	1.35824
-0.11	-1.70	-0.64	-0.57	1.23	0.43	1.79309	0.58411
-0.70	-1.72	-0.30	1.18	-0.75	-0.29	-0.62834	0.92816
#NULL!	#NULL!	#NULL!	#NULL!	#NULL!	#NULL!	#NULL!	#NULL!
#NULL!	#NULL!	#NULL!	#NULL!	#NULL!	#NULL!	#NULL!	#NULL!
#NULL!	#NULL!	#NULL!	#NULL!	#NULL!	#NULL!	#NULL!	#NULL!
1.20	-0.76	0.79	1.62	-0.70	-0.91	-1.05565	-0.62010
#NULL!	#NULL!	#NULL!	#NULL!	#NULL!	#NULL!	#NULL!	#NULL!
0.49	0.79	-0.22	-0.13	0.04	0.23	1.36578	-0.96416
#NULL!	#NULL!	#NULL!	#NULL!	#NULL!	#NULL!	#NULL!	#NULL!
-1.29	-1.01	0.23	0.31	0.04	0.33	0.22628	0.41208
#NULL!	#NULL!	#NULL!	#NULL!	#NULL!	#NULL!	#NULL!	#NULL!
#NULL!	#NULL!	#NULL!	#NULL!	#NULL!	#NULL!	#NULL!	#NULL!
#NULL!	#NULL!	#NULL!	#NULL!	#NULL!	#NULL!	#NULL!	#NULL!
#NULL!	#NULL!	#NULL!	#NULL!	#NULL!	#NULL!	#NULL!	#NULL!

0.37	-0.95	-0.63	-1.01	1.23	0.12	-0.48590	0.41208
-0.34	-0.34	-0.75	0.31	0.04	-0.08	0.36872	-0.87814
1.80	1.04	0.55	-0.57	-0.70	-0.60	0.79603	-1.65227
0.01	1.64	-0.73	-0.57	-0.75	0.33	1.36578	1.78831
#NULL!	#NULL!	#NULL!	#NULL!	#NULL!	#NULL!	#NULL!	#NULL!
1.32	0.01	-0.66	0.74	0.04	-1.73	0.65360	0.06802
#NULL!	#NULL!	#NULL!	#NULL!	#NULL!	#NULL!	#NULL!	#NULL!
#NULL!	#NULL!	#NULL!	#NULL!	#NULL!	#NULL!	#NULL!	#NULL!
#NULL!	#NULL!	#NULL!	#NULL!	#NULL!	#NULL!	#NULL!	#NULL!
#NULL!	#NULL!	#NULL!	#NULL!	#NULL!	#NULL!	#NULL!	#NULL!
#NULL!	#NULL!	#NULL!	#NULL!	#NULL!	#NULL!	#NULL!	#NULL!
0.25	0.67	0.44	-1.01	0.04	-0.70	0.08385	-0.19003
0.49	-0.38	0.13	-0.57	-0.75	1.98	1.08091	0.92816
#NULL!	#NULL!	#NULL!	#NULL!	#NULL!	#NULL!	#NULL!	#NULL!
#NULL!	#NULL!	#NULL!	#NULL!	#NULL!	#NULL!	#NULL!	#NULL!
-1.89	0.24	0.13	-0.13	-0.75	0.43	-1.62540	1.70230
-0.82	0.25	0.02	0.74	-0.75	0.12	0.65360	0.84215
#NULL!	#NULL!	#NULL!	#NULL!	#NULL!	#NULL!	#NULL!	#NULL!
#NULL!	#NULL!	#NULL!	#NULL!	#NULL!	#NULL!	#NULL!	#NULL!
#NULL!	#NULL!	#NULL!	#NULL!	#NULL!	#NULL!	#NULL!	#NULL!
#NULL!	#NULL!	#NULL!	#NULL!	#NULL!	#NULL!	#NULL!	#NULL!
#NULL!	#NULL!	#NULL!	#NULL!	#NULL!	#NULL!	#NULL!	#NULL!
#NULL!	#NULL!	#NULL!	#NULL!	#NULL!	#NULL!	#NULL!	#NULL!
0.85	-0.44	0.12	0.31	0.04	0.12	-0.62834	0.75614
#NULL!	#NULL!	#NULL!	#NULL!	#NULL!	#NULL!	#NULL!	#NULL!
#NULL!	#NULL!	#NULL!	#NULL!	#NULL!	#NULL!	#NULL!	#NULL!
#NULL!	#NULL!	#NULL!	#NULL!	#NULL!	#NULL!	#NULL!	#NULL!
#NULL!	#NULL!	#NULL!	#NULL!	#NULL!	#NULL!	#NULL!	#NULL!
0.01	-0.39	-0.50	-0.57	-0.75	1.05	-0.77078	1.96034
#NULL!	#NULL!	#NULL!	#NULL!	#NULL!	#NULL!	#NULL!	#NULL!
#NULL!	#NULL!	#NULL!	#NULL!	#NULL!	#NULL!	#NULL!	#NULL!
#NULL!	#NULL!	#NULL!	#NULL!	#NULL!	#NULL!	#NULL!	#NULL!
#NULL!	#NULL!	#NULL!	#NULL!	#NULL!	#NULL!	#NULL!	#NULL!
#NULL!	#NULL!	#NULL!	#NULL!	#NULL!	#NULL!	#NULL!	#NULL!
0.49	-0.68	0.23	1.62	1.23	0.12	0.51116	0.15403
#NULL!	#NULL!	#NULL!	#NULL!	#NULL!	#NULL!	#NULL!	#NULL!
-1.18	0.19	0.31	1.18	-0.70	0.33	1.36578	0.24005
0.49	0.33	0.13	0.74	3.22	1.36	2.22041	1.18621
-0.82	-0.17	0.81	0.31	-0.70	2.19	-0.05859	1.35824
1.68	0.40	0.04	-0.57	-0.75	-1.11	1.08091	-0.53408
#NULL!	#NULL!	#NULL!	#NULL!	#NULL!	#NULL!	#NULL!	#NULL!
#NULL!	#NULL!	#NULL!	#NULL!	#NULL!	#NULL!	#NULL!	#NULL!
#NULL!	#NULL!	#NULL!	#NULL!	#NULL!	#NULL!	#NULL!	#NULL!
#NULL!	#NULL!	#NULL!	#NULL!	#NULL!	#NULL!	#NULL!	#NULL!
0.25	-0.50	-0.32	-0.57	#NULL!	-0.70	-0.77078	0.06802
#NULL!	#NULL!	#NULL!	#NULL!	#NULL!	#NULL!	#NULL!	#NULL!
#NULL!	#NULL!	#NULL!	#NULL!	#NULL!	#NULL!	#NULL!	#NULL!
-0.46	0.53	0.25	0.74	-0.70	-0.39	1.36578	0.49809
0.61	0.11	0.37	0.74	0.04	-0.39	0.08385	-0.70611
0.37	1.76	-0.01	-1.01	-0.75	-0.91	0.51116	1.01418
#NULL!	#NULL!	#NULL!	#NULL!	#NULL!	#NULL!	#NULL!	#NULL!
-0.58	0.39	#NULL!	-0.13	0.04	1.67	1.22334	-0.01800
#NULL!	#NULL!	#NULL!	#NULL!	#NULL!	#NULL!	#NULL!	#NULL!
-1.06	-1.00	0.08	-1.01	-0.55	0.12	-0.48590	1.70230
#NULL!	#NULL!	#NULL!	#NULL!	#NULL!	#NULL!	#NULL!	#NULL!
#NULL!	#NULL!	#NULL!	#NULL!	#NULL!	#NULL!	#NULL!	#NULL!

#NULL!	#NULL!	#NULL!	#NULL!	#NULL!	#NULL!	#NULL!	#NULL!
0.97	0.10	0.25	0.74	-0.55	-1.11	0.65360	-0.96416
-0.46	0.72	0.43	-0.57	0.04	0.54	-0.20103	0.15403
#NULL!	#NULL!	#NULL!	#NULL!	#NULL!	#NULL!	#NULL!	#NULL!
-0.70	-0.32	0.87	-0.13	5.20	0.23	-0.34347	0.92816
-0.94	-0.66	-0.29	-0.13	-0.75	-0.19	-1.34053	0.49809
#NULL!	#NULL!	#NULL!	#NULL!	#NULL!	#NULL!	#NULL!	#NULL!
0.49	2.52	1.14	0.31	0.04	-0.39	-0.48590	-1.22220
#NULL!	#NULL!	#NULL!	#NULL!	#NULL!	#NULL!	#NULL!	#NULL!
-0.70	2.11	-0.01	0.31	-0.55	0.64	0.79603	0.92816
#NULL!	#NULL!	#NULL!	#NULL!	#NULL!	#NULL!	#NULL!	#NULL!
-0.11	-0.18	0.86	2.06	-0.55	-0.19	-1.19809	0.06802
#NULL!	#NULL!	#NULL!	#NULL!	#NULL!	#NULL!	#NULL!	#NULL!
#NULL!	#NULL!	#NULL!	#NULL!	#NULL!	#NULL!	#NULL!	#NULL!
#NULL!	#NULL!	#NULL!	#NULL!	#NULL!	#NULL!	#NULL!	#NULL!
#NULL!	#NULL!	#NULL!	#NULL!	#NULL!	#NULL!	#NULL!	#NULL!
-0.11	-1.43	-1.67	-1.01	0.04	0.64	0.51116	1.44425
#NULL!	#NULL!	#NULL!	#NULL!	#NULL!	#NULL!	#NULL!	#NULL!
#NULL!	#NULL!	#NULL!	#NULL!	#NULL!	#NULL!	#NULL!	#NULL!
#NULL!	#NULL!	#NULL!	#NULL!	#NULL!	#NULL!	#NULL!	#NULL!
#NULL!	#NULL!	#NULL!	#NULL!	#NULL!	#NULL!	#NULL!	#NULL!
#NULL!	#NULL!	#NULL!	#NULL!	#NULL!	#NULL!	#NULL!	#NULL!
#NULL!	#NULL!	#NULL!	#NULL!	#NULL!	#NULL!	#NULL!	#NULL!
-1.41	-0.22	-0.33	-1.01	-0.75	1.36	2.07797	-1.13619
#NULL!	#NULL!	#NULL!	#NULL!	#NULL!	#NULL!	#NULL!	#NULL!
-0.82	-0.32	-0.26	0.74	1.23	3.01	-0.20103	2.04635
-0.46	-1.40	-0.26	-0.57	-0.70	0.54	-0.77078	0.32606
1.80	3.57	-0.49	-1.01	1.23	-0.91	-0.48590	0.84215
#NULL!	#NULL!	#NULL!	#NULL!	#NULL!	#NULL!	#NULL!	#NULL!
#NULL!	#NULL!	#NULL!	#NULL!	#NULL!	#NULL!	#NULL!	#NULL!
-0.58	0.65	0.26	-0.57	1.23	1.47	-0.05859	1.78831
#NULL!	#NULL!	#NULL!	#NULL!	#NULL!	#NULL!	#NULL!	#NULL!
#NULL!	#NULL!	#NULL!	#NULL!	#NULL!	#NULL!	#NULL!	#NULL!
#NULL!	#NULL!	#NULL!	#NULL!	#NULL!	#NULL!	#NULL!	#NULL!
0.37	-0.02	0.15	-1.01	-0.70	-1.53	1.93553	-0.19003
#NULL!	#NULL!	#NULL!	#NULL!	#NULL!	#NULL!	#NULL!	#NULL!
#NULL!	#NULL!	#NULL!	#NULL!	#NULL!	#NULL!	#NULL!	#NULL!
#NULL!	#NULL!	#NULL!	#NULL!	#NULL!	#NULL!	#NULL!	#NULL!
#NULL!	#NULL!	#NULL!	#NULL!	#NULL!	#NULL!	#NULL!	#NULL!
#NULL!	#NULL!	#NULL!	#NULL!	#NULL!	#NULL!	#NULL!	#NULL!
#NULL!	#NULL!	#NULL!	#NULL!	#NULL!	#NULL!	#NULL!	#NULL!
0.85	0.47	0.61	-0.57	#NULL!	1.88	-1.91028	0.49809
#NULL!	#NULL!	#NULL!	#NULL!	#NULL!	#NULL!	#NULL!	#NULL!
1.08	0.72	-0.42	-1.01	-0.75	-0.08	1.08091	-0.87814
-0.82	-0.24	0.32	-0.57	1.23	0.33	-0.62834	-0.36205
#NULL!	#NULL!	#NULL!	#NULL!	#NULL!	#NULL!	#NULL!	#NULL!
#NULL!	#NULL!	#NULL!	#NULL!	#NULL!	#NULL!	#NULL!	#NULL!
#NULL!	#NULL!	#NULL!	#NULL!	#NULL!	#NULL!	#NULL!	#NULL!
#NULL!	#NULL!	#NULL!	#NULL!	#NULL!	#NULL!	#NULL!	#NULL!
#NULL!	#NULL!	#NULL!	#NULL!	#NULL!	#NULL!	#NULL!	#NULL!
0.01	0.35	-1.00	0.31	#NULL!	-0.70	1.36578	-0.87814
#NULL!	#NULL!	#NULL!	#NULL!	#NULL!	#NULL!	#NULL!	#NULL!
#NULL!	#NULL!	#NULL!	#NULL!	#NULL!	#NULL!	#NULL!	#NULL!
#NULL!	#NULL!	#NULL!	#NULL!	#NULL!	#NULL!	#NULL!	#NULL!
#NULL!	#NULL!	#NULL!	#NULL!	#NULL!	#NULL!	#NULL!	#NULL!
1.56	-1.21	-0.71	-0.57	-0.70	-0.08	-0.62834	0.41208

-1.18	-0.69	-0.17	-0.13	3.22	-1.42	0.36872	-1.22220
#NULL!	#NULL!	#NULL!	#NULL!	#NULL!	#NULL!	#NULL!	#NULL!
#NULL!	#NULL!	#NULL!	#NULL!	#NULL!	#NULL!	#NULL!	#NULL!
#NULL!	#NULL!	#NULL!	#NULL!	#NULL!	#NULL!	#NULL!	#NULL!
0.01	-0.23	0.55	-0.13	-0.70	-0.19	-0.77078	-0.19003
#NULL!	#NULL!	#NULL!	#NULL!	#NULL!	#NULL!	#NULL!	#NULL!
0.37	1.16	0.55	0.74	1.23	-1.84	0.36872	-1.39423
#NULL!	#NULL!	#NULL!	#NULL!	#NULL!	#NULL!	#NULL!	#NULL!
#NULL!	#NULL!	#NULL!	#NULL!	#NULL!	#NULL!	#NULL!	#NULL!
#NULL!	#NULL!	#NULL!	#NULL!	#NULL!	#NULL!	#NULL!	#NULL!
#NULL!	#NULL!	#NULL!	#NULL!	#NULL!	#NULL!	#NULL!	#NULL!
#NULL!	#NULL!	#NULL!	#NULL!	#NULL!	#NULL!	#NULL!	#NULL!
#NULL!	#NULL!	#NULL!	#NULL!	#NULL!	#NULL!	#NULL!	#NULL!
#NULL!	#NULL!	#NULL!	#NULL!	#NULL!	#NULL!	#NULL!	#NULL!
#NULL!	#NULL!	#NULL!	#NULL!	#NULL!	#NULL!	#NULL!	#NULL!
#NULL!	#NULL!	#NULL!	#NULL!	#NULL!	#NULL!	#NULL!	#NULL!
#NULL!	#NULL!	#NULL!	#NULL!	#NULL!	#NULL!	#NULL!	#NULL!
#NULL!	#NULL!	#NULL!	#NULL!	#NULL!	#NULL!	#NULL!	#NULL!

ZRD	Zpersist	ZSD	ZCOOP	ZSELFT	age	NS_diff	HA_diff
-0.63362	-0.39361	1.27772	0.01092	-0.65296	63.00	-6.00	4.00
#NULL!	#NULL!	#NULL!	#NULL!	#NULL!	65.00	8.68	-4.00
-0.95441	-0.28616	0.15551	0.48485	-0.45665	63.00	-2.00	6.00
0.11489	0.78837	0.06918	-0.93692	1.01563	#NULL!	#NULL!	#NULL!
-2.55837	0.68092	1.10507	-0.10756	-1.24187	63.00	-3.00	7.00
0.64955	0.89583	1.62301	1.43269	0.23041	#NULL!	#NULL!	#NULL!
0.64955	0.78837	1.62301	0.72181	0.03411	49.00	6.00	3.00
-0.52669	1.97036	1.36404	-0.93692	-1.34002	78.00	-6.00	3.00
#NULL!	#NULL!	#NULL!	#NULL!	#NULL!	59.00	-1.75	-2.33
1.71885	0.14365	0.41448	0.12940	1.31009	#NULL!	#NULL!	#NULL!
-0.31283	-0.82343	1.01875	0.95877	-0.65296	68.00	1.00	-3.00
#NULL!	#NULL!	#NULL!	#NULL!	#NULL!	69.00	-2.00	3.00
#NULL!	#NULL!	#NULL!	#NULL!	#NULL!	52.00	-5.00	6.00
#NULL!	#NULL!	#NULL!	#NULL!	#NULL!	#NULL!	#NULL!	#NULL!
-0.84748	0.57347	-0.62141	-1.17388	0.62302	82.00	-3.00	4.00
#NULL!	#NULL!	#NULL!	#NULL!	#NULL!	52.00	-5.00	-3.00
#NULL!	#NULL!	#NULL!	#NULL!	#NULL!	70.00	5.00	1.00
0.00796	-3.07995	1.62301	0.95877	-2.02709	72.00	4.00	3.00
-1.06134	-0.28616	-1.65730	-1.41084	-1.14372	77.00	-6.84	3.00
-1.48907	-0.28616	-0.10347	0.60333	-1.04557	72.00	0.00	-13.00
-0.63362	0.25111	0.84610	-0.10756	-1.53633	51.00	4.00	0.00
#NULL!	#NULL!	#NULL!	#NULL!	#NULL!	68.00	3.00	-0.21
#NULL!	#NULL!	#NULL!	#NULL!	#NULL!	59.00	-3.00	-3.00
-1.80986	-0.28616	0.50080	-0.69996	-1.24187	46.00	4.00	7.00
-0.09897	-0.39361	-0.01714	0.36637	-1.24187	64.00	-1.00	-1.00
#NULL!	#NULL!	#NULL!	#NULL!	#NULL!	65.00	2.00	0.00
#NULL!	#NULL!	#NULL!	#NULL!	#NULL!	62.00	-2.00	10.00
#NULL!	#NULL!	#NULL!	#NULL!	#NULL!	51.00	3.00	-9.00
#NULL!	#NULL!	#NULL!	#NULL!	#NULL!	#NULL!	#NULL!	#NULL!
#NULL!	#NULL!	#NULL!	#NULL!	#NULL!	61.00	-1.00	5.00
#NULL!	#NULL!	#NULL!	#NULL!	#NULL!	68.00	7.00	1.00
-0.09897	-0.82343	1.62301	1.07725	-2.02709	74.00	-3.00	4.00
0.00796	-1.46815	-0.62141	-0.69996	-1.04557	55.00	-1.00	-10.00
#NULL!	#NULL!	#NULL!	#NULL!	#NULL!	63.00	-6.00	-2.00
#NULL!	#NULL!	#NULL!	#NULL!	#NULL!	#NULL!	#NULL!	#NULL!
#NULL!	#NULL!	#NULL!	#NULL!	#NULL!	#NULL!	#NULL!	#NULL!
#NULL!	#NULL!	#NULL!	#NULL!	#NULL!	#NULL!	#NULL!	#NULL!
#NULL!	#NULL!	#NULL!	#NULL!	#NULL!	#NULL!	#NULL!	#NULL!
#NULL!	#NULL!	#NULL!	#NULL!	#NULL!	#NULL!	#NULL!	#NULL!
#NULL!	#NULL!	#NULL!	#NULL!	#NULL!	62.00	2.00	-7.00
#NULL!	#NULL!	#NULL!	#NULL!	#NULL!	95.00	-4.00	9.00
1.29113	-0.60852	0.24183	-0.10756	0.52487	80.00	-2.00	4.00
#NULL!	#NULL!	#NULL!	#NULL!	#NULL!	64.00	-5.00	-7.00
#NULL!	#NULL!	#NULL!	#NULL!	#NULL!	69.00	-1.00	2.00
#NULL!	#NULL!	#NULL!	#NULL!	#NULL!	#NULL!	#NULL!	#NULL!
1.07727	2.50762	0.41448	-1.05540	0.52487	46.00	-5.00	4.00
0.97034	-0.50107	1.36404	0.95877	0.42672	64.00	3.00	1.00
#NULL!	#NULL!	#NULL!	#NULL!	#NULL!	59.00	7.00	12.00
0.64955	2.18526	1.10507	0.12940	-0.16219	58.00	-3.00	-4.00
#NULL!	#NULL!	#NULL!	#NULL!	#NULL!	60.00	-9.00	2.00
0.54262	1.43309	0.67345	1.90661	-0.26035	68.00	1.00	6.00
#NULL!	#NULL!	#NULL!	#NULL!	#NULL!	66.00	5.00	-0.11
#NULL!	#NULL!	#NULL!	#NULL!	#NULL!	66.00	-7.00	3.00
#NULL!	#NULL!	#NULL!	#NULL!	#NULL!	72.00	0.00	-3.00
-0.09897	-0.50107	-0.01714	-0.10756	-0.75111	62.00	4.00	1.00

-0.95441	-0.28616	0.58713	-0.10756	-1.24187	69.00	-1.00	0.00
#NULL!	#NULL!	#NULL!	#NULL!	#NULL!	63.00	5.00	-7.00
0.64955	0.89583	0.75977	-0.10756	0.03411	49.00	4.00	5.00
0.75648	1.21818	-0.10347	0.48485	1.80085	68.00	0.00	-4.00
-0.41976	0.68092	0.93242	0.36637	-0.35850	68.00	-5.00	-1.00
#NULL!	#NULL!	#NULL!	#NULL!	#NULL!	67.00	-1.00	1.00
#NULL!	#NULL!	#NULL!	#NULL!	#NULL!	69.00	-5.00	7.00
0.32876	-0.39361	0.24183	-0.34452	-0.75111	65.00	1.00	10.00
#NULL!	#NULL!	#NULL!	#NULL!	#NULL!	46.00	0.00	10.00
-1.59600	-0.39361	-0.18979	-1.76628	-0.45665	78.00	3.00	1.00
1.71885	0.68092	-0.18979	-1.52932	1.21194	76.00	0.00	-1.00
0.64955	-0.50107	0.75977	-0.58148	-1.24187	75.00	3.00	11.00
#NULL!	#NULL!	#NULL!	#NULL!	#NULL!	77.00	2.00	11.00
#NULL!	#NULL!	#NULL!	#NULL!	#NULL!	77.00	6.00	5.00
0.00796	-0.71597	-1.13935	-0.46300	1.11378	62.00	-2.00	-3.00
-0.41976	-2.54268	1.62301	0.84029	-0.45665	46.00	-4.00	-2.00
-1.16828	0.25111	0.93242	0.84029	-0.45665	60.00	-3.00	-7.00
#NULL!	#NULL!	#NULL!	#NULL!	#NULL!	57.00	0.00	6.00
1.50499	0.68092	-1.31200	0.60333	0.23041	56.00	9.00	-2.00
#NULL!	#NULL!	#NULL!	#NULL!	#NULL!	53.00	7.00	-4.47
0.22182	-1.79051	1.96831	2.02509	-1.14372	55.00	4.00	13.00
#NULL!	#NULL!	#NULL!	#NULL!	#NULL!	65.00	-3.00	9.00
#NULL!	#NULL!	#NULL!	#NULL!	#NULL!	47.00	-4.00	10.00
-1.27521	0.25111	-0.70773	0.12940	-0.16219	56.00	5.00	11.00
-0.52669	-0.17871	-0.18979	-0.22604	-0.06404	60.00	3.00	2.00
#NULL!	#NULL!	#NULL!	#NULL!	#NULL!	61.00	-3.00	-3.00
-0.74055	0.35856	-0.44876	-0.10756	-0.55480	61.00	1.00	0.00
#NULL!	#NULL!	#NULL!	#NULL!	#NULL!	52.00	1.00	2.00
#NULL!	#NULL!	#NULL!	#NULL!	#NULL!	78.00	2.00	1.00
0.43569	1.43309	1.45036	1.78813	1.70270	51.00	2.00	9.00
0.00796	-0.39361	1.53669	0.95877	1.21194	55.00	5.00	-4.00
0.32876	0.89583	0.06918	1.55117	1.21194	58.00	1.00	-5.00
#NULL!	#NULL!	#NULL!	#NULL!	#NULL!	53.00	-2.00	5.00
#NULL!	#NULL!	#NULL!	#NULL!	#NULL!	59.00	2.00	-7.00
0.22182	0.25111	0.67345	1.31421	-0.65296	61.00	-8.00	3.00
#NULL!	#NULL!	#NULL!	#NULL!	#NULL!	#NULL!	#NULL!	#NULL!
#NULL!	#NULL!	#NULL!	#NULL!	#NULL!	57.00	6.00	1.00
#NULL!	#NULL!	#NULL!	#NULL!	#NULL!	#NULL!	#NULL!	#NULL!
-0.95441	-0.28616	-2.77951	-1.41084	-1.53633	49.00	4.00	-3.00
-0.95441	-0.28616	0.75977	-0.34452	-0.94741	70.00	-2.00	3.00
#NULL!	#NULL!	#NULL!	#NULL!	#NULL!	67.00	-2.00	7.00
#NULL!	#NULL!	#NULL!	#NULL!	#NULL!	59.00	0.16	-0.33
#NULL!	#NULL!	#NULL!	#NULL!	#NULL!	59.00	-2.00	0.00
#NULL!	#NULL!	#NULL!	#NULL!	#NULL!	70.00	0.00	6.00
-0.95441	-0.39361	-0.70773	-1.52932	0.42672	54.00	-2.00	3.00
-1.16828	-0.50107	0.50080	0.36637	-0.94741	75.00	-1.00	6.00
-0.31283	0.68092	-0.10347	-0.34452	0.42672	60.00	-2.00	-1.00
#NULL!	#NULL!	#NULL!	#NULL!	#NULL!	52.00	0.00	4.00
#NULL!	#NULL!	#NULL!	#NULL!	#NULL!	#NULL!	#NULL!	#NULL!
#NULL!	#NULL!	#NULL!	#NULL!	#NULL!	50.00	-2.00	-12.00
#NULL!	#NULL!	#NULL!	#NULL!	#NULL!	73.00	-3.00	2.00
#NULL!	#NULL!	#NULL!	#NULL!	#NULL!	50.00	3.00	-5.00
2.46737	1.86290	0.75977	1.31421	-0.35850	62.00	2.00	7.00
#NULL!	#NULL!	#NULL!	#NULL!	#NULL!	49.00	-3.00	-1.00
#NULL!	#NULL!	#NULL!	#NULL!	#NULL!	#NULL!	#NULL!	#NULL!
0.11489	-1.57560	-0.70773	0.36637	0.23041	64.00	1.00	4.00

#NULL!	#NULL!	#NULL!	#NULL!	#NULL!	64.00	-9.00	-3.00
-1.06134	-0.17871	0.93242	0.12940	-1.53633	72.00	3.00	-2.00
#NULL!	#NULL!	#NULL!	#NULL!	#NULL!	48.00	7.00	7.00
-2.02372	-0.60852	0.41448	-1.17388	-1.53633	62.00	0.00	6.00
#NULL!	#NULL!	#NULL!	#NULL!	#NULL!	63.00	4.00	0.00
#NULL!	#NULL!	#NULL!	#NULL!	#NULL!	87.00	2.00	-3.00
#NULL!	#NULL!	#NULL!	#NULL!	#NULL!	64.00	2.26	3.63
#NULL!	#NULL!	#NULL!	#NULL!	#NULL!	#NULL!	#NULL!	#NULL!
#NULL!	#NULL!	#NULL!	#NULL!	#NULL!	51.00	-8.00	5.68
-2.13065	-0.50107	0.24183	0.24788	-0.65296	47.00	4.00	1.00
#NULL!	#NULL!	#NULL!	#NULL!	#NULL!	58.00	0.00	3.00
#NULL!	#NULL!	#NULL!	#NULL!	#NULL!	61.00	1.00	-7.00
-0.74055	0.14365	-0.18979	-1.05540	-0.94741	69.00	6.00	-3.00
#NULL!	#NULL!	#NULL!	#NULL!	#NULL!	#NULL!	#NULL!	#NULL!
#NULL!	#NULL!	#NULL!	#NULL!	#NULL!	57.00	2.00	-1.00
-0.52669	0.25111	-0.88038	-1.52932	0.13226	76.00	-2.00	-1.00
#NULL!	#NULL!	#NULL!	#NULL!	#NULL!	62.00	7.00	-3.00
#NULL!	#NULL!	#NULL!	#NULL!	#NULL!	58.00	-1.00	-4.00
-1.16828	-0.93088	-0.18979	0.01092	0.23041	73.00	1.00	-10.00
1.18420	0.57347	-0.27611	-0.81844	0.81933	#NULL!	#NULL!	#NULL!
#NULL!	#NULL!	#NULL!	#NULL!	#NULL!	59.00	-5.00	-7.00
-0.95441	-0.07125	0.41448	-0.69996	-0.94741	49.00	7.00	-7.00
#NULL!	#NULL!	#NULL!	#NULL!	#NULL!	59.00	-8.00	5.00
#NULL!	#NULL!	#NULL!	#NULL!	#NULL!	46.00	6.00	-5.00
0.22182	1.32564	0.41448	0.72181	-0.55480	64.00	-11.00	-6.00
-1.27521	0.35856	-0.96671	0.12940	-1.53633	46.00	2.00	-2.00
1.18420	0.25111	2.05463	2.26205	1.99715	#NULL!	#NULL!	#NULL!
1.29113	1.21818	-1.05303	1.19573	-1.43817	57.00	3.00	-9.00
-0.52669	0.25111	1.01875	-0.46300	0.03411	67.00	0.00	-3.00
#NULL!	#NULL!	#NULL!	#NULL!	#NULL!	55.00	4.00	0.00
#NULL!	#NULL!	#NULL!	#NULL!	#NULL!	56.00	3.00	-3.68
1.39806	1.97036	2.22728	0.72181	-1.73263	45.00	-5.00	7.00
#NULL!	#NULL!	#NULL!	#NULL!	#NULL!	70.00	3.00	2.84
#NULL!	#NULL!	#NULL!	#NULL!	#NULL!	66.00	-3.00	2.00
0.00796	-1.25324	-0.62141	-0.46300	-0.55480	73.00	-1.00	-6.00
-0.95441	-0.17871	1.62301	0.72181	0.72118	73.00	2.00	-1.58
#NULL!	#NULL!	#NULL!	#NULL!	#NULL!	78.00	3.00	-2.00
-0.09897	0.57347	-0.70773	0.12940	1.01563	55.00	1.00	-1.00
-0.95441	0.25111	0.24183	-0.10756	-1.24187	65.00	-2.00	6.00
#NULL!	#NULL!	#NULL!	#NULL!	#NULL!	58.00	-7.67	-7.38
#NULL!	#NULL!	#NULL!	#NULL!	#NULL!	71.00	-3.00	11.00
-0.41976	-1.25324	-0.70773	-1.29236	-0.55480	52.00	-7.00	-6.68
-0.09897	-0.50107	-2.08892	-0.10756	0.52487	47.00	1.00	-1.21
0.22182	-0.93088	0.24183	-0.69996	0.23041	55.00	2.00	-11.00
#NULL!	#NULL!	#NULL!	#NULL!	#NULL!	67.00	6.00	-3.00
#NULL!	#NULL!	#NULL!	#NULL!	#NULL!	67.00	1.00	2.00
#NULL!	#NULL!	#NULL!	#NULL!	#NULL!	#NULL!	#NULL!	#NULL!
-1.27521	0.14365	0.67345	1.19573	0.81933	#NULL!	#NULL!	#NULL!
#NULL!	#NULL!	#NULL!	#NULL!	#NULL!	55.00	-1.00	0.00
0.86341	-0.82343	0.32815	0.48485	1.80085	58.00	7.00	-5.11
#NULL!	#NULL!	#NULL!	#NULL!	#NULL!	74.00	-11.32	-3.00
-0.09897	-1.14579	-0.79406	-1.29236	-0.55480	69.00	-6.00	-1.05
#NULL!	#NULL!	#NULL!	#NULL!	#NULL!	53.00	1.00	8.00
#NULL!	#NULL!	#NULL!	#NULL!	#NULL!	57.00	3.00	1.00
#NULL!	#NULL!	#NULL!	#NULL!	#NULL!	54.00	-2.00	0.00
#NULL!	#NULL!	#NULL!	#NULL!	#NULL!	53.00	-4.00	5.00

0.22182	-0.07125	-0.88038	0.36637	-0.06404	#NULL!	#NULL!	#NULL!
1.82579	1.43309	1.01875	-0.34452	1.21194	50.00	4.00	-9.00
0.64955	1.54054	0.93242	1.78813	0.52487	55.00	6.00	-7.00
0.22182	-0.07125	-1.13935	0.01092	0.52487	69.00	1.00	-8.68
#NULL!	#NULL!	#NULL!	#NULL!	#NULL!	55.00	0.00	6.21
-0.20590	-0.93088	-0.10347	1.31421	1.11378	70.00	-5.11	-9.00
#NULL!	#NULL!	#NULL!	#NULL!	#NULL!	56.00	-2.00	-1.00
#NULL!	#NULL!	#NULL!	#NULL!	#NULL!	48.00	0.00	-9.00
#NULL!	#NULL!	#NULL!	#NULL!	#NULL!	69.00	-10.00	14.00
#NULL!	#NULL!	#NULL!	#NULL!	#NULL!	66.00	4.00	-2.00
#NULL!	#NULL!	#NULL!	#NULL!	#NULL!	77.00	-3.00	-2.00
0.86341	0.89583	-1.22568	0.24788	0.62302	48.00	-3.00	6.00
1.07727	1.64800	0.32815	0.48485	1.01563	59.00	6.00	-11.00
#NULL!	#NULL!	#NULL!	#NULL!	#NULL!	51.00	2.00	-4.00
#NULL!	#NULL!	#NULL!	#NULL!	#NULL!	61.00	-9.00	-16.44
-1.38214	-3.72467	-3.03848	-1.88477	-0.94741	67.00	-17.00	-7.00
-0.84748	-1.14579	-0.96671	-0.81844	-0.26035	69.00	-4.00	-5.00
#NULL!	#NULL!	#NULL!	#NULL!	#NULL!	69.00	-1.00	0.00
#NULL!	#NULL!	#NULL!	#NULL!	#NULL!	68.00	-4.00	1.00
#NULL!	#NULL!	#NULL!	#NULL!	#NULL!	77.00	-2.00	-1.00
#NULL!	#NULL!	#NULL!	#NULL!	#NULL!	75.00	0.00	5.00
#NULL!	#NULL!	#NULL!	#NULL!	#NULL!	67.00	-6.00	-3.00
#NULL!	#NULL!	#NULL!	#NULL!	#NULL!	49.00	2.00	2.00
0.32876	0.57347	0.50080	0.84029	-0.06404	52.00	3.00	-13.00
#NULL!	#NULL!	#NULL!	#NULL!	#NULL!	69.00	-7.68	0.00
#NULL!	#NULL!	#NULL!	#NULL!	#NULL!	68.00	3.00	6.00
#NULL!	#NULL!	#NULL!	#NULL!	#NULL!	54.00	1.00	2.00
#NULL!	#NULL!	#NULL!	#NULL!	#NULL!	60.00	-5.00	3.00
0.32876	-0.28616	-0.01714	0.01092	1.40824	69.00	4.00	-12.00
#NULL!	#NULL!	#NULL!	#NULL!	#NULL!	53.00	-1.00	-2.00
#NULL!	#NULL!	#NULL!	#NULL!	#NULL!	46.00	-1.00	2.00
#NULL!	#NULL!	#NULL!	#NULL!	#NULL!	80.00	6.00	-3.00
#NULL!	#NULL!	#NULL!	#NULL!	#NULL!	79.00	-2.00	2.00
#NULL!	#NULL!	#NULL!	#NULL!	#NULL!	61.00	-7.00	8.00
0.00796	-1.46815	-1.48465	0.48485	-0.75111	61.00	5.00	0.00
#NULL!	#NULL!	#NULL!	#NULL!	#NULL!	75.00	4.00	-5.00
-0.41976	0.03620	-0.36244	-1.17388	-0.75111	66.00	-4.00	1.00
0.22182	-0.93088	-3.29745	0.48485	0.52487	45.00	3.00	-14.00
-2.02372	0.78837	-1.39833	-0.81844	-0.65296	#NULL!	#NULL!	#NULL!
0.75648	-1.46815	0.06918	1.66965	1.40824	70.00	-1.00	11.00
#NULL!	#NULL!	#NULL!	#NULL!	#NULL!	78.00	-3.00	5.00
#NULL!	#NULL!	#NULL!	#NULL!	#NULL!	59.00	7.00	9.00
#NULL!	#NULL!	#NULL!	#NULL!	#NULL!	68.00	0.00	2.00
#NULL!	#NULL!	#NULL!	#NULL!	#NULL!	#NULL!	#NULL!	#NULL!
0.86341	0.46601	-0.27611	0.24788	0.42672	59.00	5.00	-1.00
#NULL!	#NULL!	#NULL!	#NULL!	#NULL!	55.00	-1.00	-4.00
#NULL!	#NULL!	#NULL!	#NULL!	#NULL!	60.00	-3.00	-2.00
0.32876	-2.22032	0.06918	-0.46300	-0.84926	75.00	-3.00	1.00
0.32876	0.03620	0.50080	0.60333	0.32857	45.00	3.00	-5.00
-1.70293	-0.07125	-1.05303	0.36637	2.38976	54.00	2.00	-10.00
#NULL!	#NULL!	#NULL!	#NULL!	#NULL!	#NULL!	#NULL!	#NULL!
0.22182	1.32564	-1.31200	-0.58148	1.50639	63.00	-8.00	2.00
#NULL!	#NULL!	#NULL!	#NULL!	#NULL!	73.00	-4.00	5.00
-0.95441	-0.71597	-2.00259	-1.05540	-1.53633	51.00	-9.30	-9.79
#NULL!	#NULL!	#NULL!	#NULL!	#NULL!	57.00	-3.00	-2.00
#NULL!	#NULL!	#NULL!	#NULL!	#NULL!	50.00	-4.05	-3.00

#NULL!	#NULL!	#NULL!	#NULL!	#NULL!	70.00	2.00	-9.00
2.36044	0.25111	0.50080	0.95877	1.70270	57.00	12.00	7.00
-0.41976	-0.93088	-0.36244	-0.46300	-0.65296	50.00	6.00	2.00
#NULL!	#NULL!	#NULL!	#NULL!	#NULL!	71.00	0.00	2.00
0.32876	-1.46815	-0.96671	-0.69996	-0.16219	51.00	2.00	-5.00
-1.16828	0.35856	0.41448	-0.93692	-1.04557	68.00	-0.37	-5.00
#NULL!	#NULL!	#NULL!	#NULL!	#NULL!	66.00	2.00	4.00
-1.80986	2.18526	0.84610	0.48485	-1.14372	77.00	2.00	4.00
#NULL!	#NULL!	#NULL!	#NULL!	#NULL!	56.00	2.00	-9.00
-0.41976	-0.60852	-0.44876	-0.69996	-0.06404	55.00	3.00	-2.00
#NULL!	#NULL!	#NULL!	#NULL!	#NULL!	56.00	-1.00	5.00
-1.70293	0.78837	0.67345	-0.10756	-0.26035	76.00	0.00	-4.00
#NULL!	#NULL!	#NULL!	#NULL!	#NULL!	59.00	1.00	4.47
#NULL!	#NULL!	#NULL!	#NULL!	#NULL!	54.00	0.00	13.00
#NULL!	#NULL!	#NULL!	#NULL!	#NULL!	47.00	2.00	0.00
#NULL!	#NULL!	#NULL!	#NULL!	#NULL!	64.00	-15.00	9.05
0.54262	0.35856	-1.91627	-0.10756	0.62302	56.00	-9.00	-3.74
#NULL!	#NULL!	#NULL!	#NULL!	#NULL!	54.00	0.00	-3.00
#NULL!	#NULL!	#NULL!	#NULL!	#NULL!	82.00	1.00	-10.00
#NULL!	#NULL!	#NULL!	#NULL!	#NULL!	52.00	-5.00	-1.00
#NULL!	#NULL!	#NULL!	#NULL!	#NULL!	59.00	6.00	7.00
#NULL!	#NULL!	#NULL!	#NULL!	#NULL!	55.00	3.00	-6.00
#NULL!	#NULL!	#NULL!	#NULL!	#NULL!	#NULL!	#NULL!	#NULL!
0.97034	1.00328	-1.65730	-1.41084	1.89900	56.00	-4.00	12.68
#NULL!	#NULL!	#NULL!	#NULL!	#NULL!	59.00	4.00	-3.00
-1.70293	-0.82343	-1.82995	-0.81844	-1.14372	66.00	3.00	-7.00
-0.95441	0.25111	0.15551	-0.46300	-0.45665	53.00	0.00	3.00
0.11489	0.46601	0.67345	1.78813	0.03411	50.00	-7.00	-1.00
#NULL!	#NULL!	#NULL!	#NULL!	#NULL!	63.00	3.00	-6.00
#NULL!	#NULL!	#NULL!	#NULL!	#NULL!	#NULL!	#NULL!	#NULL!
0.64955	-0.07125	-1.13935	-0.58148	-0.06404	48.00	-7.00	12.00
#NULL!	#NULL!	#NULL!	#NULL!	#NULL!	70.00	2.00	4.00
#NULL!	#NULL!	#NULL!	#NULL!	#NULL!	#NULL!	#NULL!	#NULL!
#NULL!	#NULL!	#NULL!	#NULL!	#NULL!	73.00	-1.00	0.00
0.54262	0.57347	0.58713	0.36637	0.23041	62.00	-9.00	-16.00
#NULL!	#NULL!	#NULL!	#NULL!	#NULL!	63.00	11.21	1.82
#NULL!	#NULL!	#NULL!	#NULL!	#NULL!	76.00	2.63	-6.00
#NULL!	#NULL!	#NULL!	#NULL!	#NULL!	66.00	-1.00	-11.00
#NULL!	#NULL!	#NULL!	#NULL!	#NULL!	70.00	-2.00	-6.00
#NULL!	#NULL!	#NULL!	#NULL!	#NULL!	65.00	-3.79	-1.00
#NULL!	#NULL!	#NULL!	#NULL!	#NULL!	74.00	3.00	2.26
-0.63362	0.57347	0.15551	0.84029	0.42672	#NULL!	#NULL!	#NULL!
#NULL!	#NULL!	#NULL!	#NULL!	#NULL!	77.00	-0.62	10.11
-0.41976	-1.03833	0.50080	1.07725	0.72118	68.00	-1.00	-8.00
0.54262	1.00328	0.58713	-0.81844	-0.16219	56.00	-1.00	4.00
#NULL!	#NULL!	#NULL!	#NULL!	#NULL!	86.00	8.00	5.00
#NULL!	#NULL!	#NULL!	#NULL!	#NULL!	80.00	3.00	-1.05
#NULL!	#NULL!	#NULL!	#NULL!	#NULL!	66.00	-9.00	0.00
#NULL!	#NULL!	#NULL!	#NULL!	#NULL!	54.00	-2.00	-3.00
#NULL!	#NULL!	#NULL!	#NULL!	#NULL!	53.00	-2.00	-2.00
0.32876	-0.28616	0.32815	0.01092	-0.84926	#NULL!	#NULL!	#NULL!
#NULL!	#NULL!	#NULL!	#NULL!	#NULL!	72.00	-2.33	-1.43
#NULL!	#NULL!	#NULL!	#NULL!	#NULL!	76.00	-2.11	-3.00
#NULL!	#NULL!	#NULL!	#NULL!	#NULL!	61.00	-8.00	-2.00
#NULL!	#NULL!	#NULL!	#NULL!	#NULL!	#NULL!	#NULL!	#NULL!
-0.31283	-0.82343	0.75977	1.55117	-1.83078	65.00	1.00	-6.37

0.75648	2.18526	0.41448	-1.17388	-0.94741	62.00	3.00	-4.00
#NULL!	#NULL!	#NULL!	#NULL!	#NULL!	59.00	-4.00	8.00
#NULL!	#NULL!	#NULL!	#NULL!	#NULL!	72.00	4.00	-8.26
#NULL!	#NULL!	#NULL!	#NULL!	#NULL!	67.00	-2.00	-6.00
0.32876	0.57347	-0.88038	0.01092	0.52487	50.00	3.00	-2.00
#NULL!	#NULL!	#NULL!	#NULL!	#NULL!	53.00	-5.00	-2.00
0.86341	0.03620	1.45036	0.36637	0.52487	59.00	-9.00	0.00
#NULL!	#NULL!	#NULL!	#NULL!	#NULL!	66.00	2.00	1.00
#NULL!	#NULL!	#NULL!	#NULL!	#NULL!	48.00	-4.00	5.00
#NULL!	#NULL!	#NULL!	#NULL!	#NULL!	70.00	#NULL!	#NULL!
#NULL!	#NULL!	#NULL!	#NULL!	#NULL!	72.00	#NULL!	#NULL!
#NULL!	#NULL!	#NULL!	#NULL!	#NULL!	72.00	#NULL!	#NULL!
#NULL!	#NULL!	#NULL!	#NULL!	#NULL!	56.00	#NULL!	#NULL!
#NULL!	#NULL!	#NULL!	#NULL!	#NULL!	69.00	#NULL!	#NULL!
#NULL!	#NULL!	#NULL!	#NULL!	#NULL!	61.00	#NULL!	#NULL!
#NULL!	#NULL!	#NULL!	#NULL!	#NULL!	52.00	#NULL!	#NULL!
#NULL!	#NULL!	#NULL!	#NULL!	#NULL!	#NULL!	#NULL!	#NULL!
#NULL!	#NULL!	#NULL!	#NULL!	#NULL!	#NULL!	#NULL!	#NULL!

RD_diff	PS_diff	SD_diff	CO_diff	ST_diff	NS_abs	HA_abs	RD_abs
-4.11	0.00	-5.00	2.00	-9.00	6.00	4.00	4.11
3.00	2.32	1.00	-5.00	3.00	8.68	4.00	3.00
-5.00	1.00	1.00	-2.00	5.00	2.00	6.00	5.00
#NULL!	#NULL!	#NULL!	#NULL!	#NULL!	#NULL!	#NULL!	#NULL!
4.26	-10.00	-24.00	12.00	-1.00	3.00	7.00	4.26
#NULL!	#NULL!	#NULL!	#NULL!	#NULL!	#NULL!	#NULL!	#NULL!
-3.00	-10.00	-15.00	-1.00	-6.00	6.00	3.00	3.00
4.00	9.00	-4.89	13.00	17.00	6.00	3.00	4.00
-4.00	16.00	5.33	35.00	8.00	1.75	2.33	4.00
#NULL!	#NULL!	#NULL!	#NULL!	#NULL!	#NULL!	#NULL!	#NULL!
5.26	-1.00	0.00	-3.00	3.57	1.00	3.00	5.26
0.00	3.05	-7.00	-2.00	2.00	2.00	3.00	0.00
-4.00	-5.00	1.00	0.00	-2.00	5.00	6.00	4.00
#NULL!	#NULL!	#NULL!	#NULL!	#NULL!	#NULL!	#NULL!	#NULL!
-3.00	-4.00	1.00	0.00	-1.00	3.00	4.00	3.00
12.00	2.00	3.00	6.00	-5.00	5.00	3.00	12.00
-6.00	0.00	0.00	2.00	5.00	5.00	1.00	6.00
-5.00	5.00	2.00	2.00	0.00	4.00	3.00	5.00
-4.11	-3.00	4.26	1.00	8.00	6.84	3.00	4.11
5.00	8.00	2.00	2.00	9.00	0.00	13.00	5.00
5.00	-2.00	1.00	2.00	3.00	4.00	0.00	5.00
-2.00	3.00	9.00	-5.00	-5.00	3.00	0.21	2.00
1.00	-3.00	8.00	-2.42	-11.00	3.00	3.00	1.00
8.00	-4.00	-14.00	-3.00	17.00	4.00	7.00	8.00
3.00	-1.00	-4.00	-8.00	-7.00	1.00	1.00	3.00
5.00	-6.00	3.00	-4.00	3.00	2.00	0.00	5.00
5.00	3.00	5.00	3.00	-1.00	2.00	10.00	5.00
-1.00	1.00	-3.00	5.00	11.00	3.00	9.00	1.00
#NULL!	#NULL!	#NULL!	#NULL!	#NULL!	#NULL!	#NULL!	#NULL!
-7.00	-2.00	-7.00	-1.00	5.00	1.00	5.00	7.00
-1.37	1.00	0.00	1.00	-8.00	7.00	1.00	1.37
3.00	2.00	-3.00	-11.00	5.00	3.00	4.00	3.00
-6.00	-1.00	-11.00	3.00	4.00	1.00	10.00	6.00
-7.00	-5.00	2.00	16.00	7.00	6.00	2.00	7.00
#NULL!	#NULL!	#NULL!	#NULL!	#NULL!	#NULL!	#NULL!	#NULL!
#NULL!	#NULL!	#NULL!	#NULL!	#NULL!	#NULL!	#NULL!	#NULL!
#NULL!	#NULL!	#NULL!	#NULL!	#NULL!	#NULL!	#NULL!	#NULL!
#NULL!	#NULL!	#NULL!	#NULL!	#NULL!	#NULL!	#NULL!	#NULL!
#NULL!	#NULL!	#NULL!	#NULL!	#NULL!	#NULL!	#NULL!	#NULL!
0.00	-1.00	-3.58	-3.00	-3.00	2.00	7.00	0.00
4.33	-8.00	-11.00	-21.00	2.00	4.00	9.00	4.33
-7.00	1.00	-7.00	0.00	-1.00	2.00	4.00	7.00
-1.00	0.00	-6.00	2.00	4.00	5.00	7.00	1.00
-4.00	-9.00	3.00	2.00	5.00	1.00	2.00	4.00
#NULL!	#NULL!	#NULL!	#NULL!	#NULL!	#NULL!	#NULL!	#NULL!
-3.42	-11.00	11.00	11.00	-8.00	5.00	4.00	3.42
-3.00	1.00	-7.00	-3.00	5.00	3.00	1.00	3.00
-2.74	-4.00	-15.00	1.00	8.00	7.00	12.00	2.74
4.00	4.00	-3.00	-6.00	1.00	3.00	4.00	4.00
6.47	-1.00	0.00	14.00	-2.00	9.00	2.00	6.47
-3.00	-7.00	11.00	-3.00	-6.00	1.00	6.00	3.00
4.00	-7.00	10.00	5.00	-3.00	5.00	0.11	4.00
-7.00	-2.00	-3.00	-1.00	-3.00	7.00	3.00	7.00
2.00	5.00	0.00	5.00	7.00	0.00	3.00	2.00
5.00	0.00	0.00	6.00	13.00	4.00	1.00	5.00

10.00	-2.00	-1.00	3.00	6.00	1.00	0.00	10.00
2.26	2.00	20.00	12.00	-16.00	5.00	7.00	2.26
-2.00	-16.00	-3.00	3.89	-3.00	4.00	5.00	2.00
2.00	8.00	-3.00	2.00	-3.00	0.00	4.00	2.00
4.00	0.00	0.00	0.00	-8.00	5.00	1.00	4.00
-1.00	-3.00	-0.32	5.00	4.00	1.00	1.00	1.00
-11.00	-9.00	10.00	-11.00	-4.00	5.00	7.00	11.00
-3.00	6.00	4.00	7.00	0.00	1.00	10.00	3.00
-10.00	-1.00	7.00	-3.00	7.00	0.00	10.00	10.00
4.00	-3.00	0.00	-4.21	-1.00	3.00	1.00	4.00
2.00	-4.00	4.00	7.00	1.00	0.00	1.00	2.00
1.63	1.16	-6.00	4.74	12.00	3.00	11.00	1.63
0.00	0.00	2.00	3.00	0.00	2.00	11.00	0.00
-1.00	-2.00	-5.37	-2.00	0.00	6.00	5.00	1.00
-12.00	-2.00	1.00	10.00	-2.00	2.00	3.00	12.00
16.00	10.00	-2.00	10.00	2.00	4.00	2.00	16.00
6.00	6.00	4.00	-1.00	11.00	3.00	7.00	6.00
5.00	-1.00	-2.00	4.00	-1.00	0.00	6.00	5.00
-1.00	-8.00	-2.00	-3.00	1.00	9.00	2.00	1.00
-6.79	-1.89	-5.00	-2.79	8.00	7.00	4.47	6.79
7.00	5.00	-6.00	-7.00	2.00	4.00	13.00	7.00
1.00	-2.00	5.00	7.00	13.00	3.00	9.00	1.00
11.00	8.00	6.00	2.00	3.00	4.00	10.00	11.00
-7.00	-22.44	-6.00	-11.00	-1.00	5.00	11.00	7.00
4.00	-1.00	4.00	1.00	-6.00	3.00	2.00	4.00
3.00	-2.00	0.00	9.00	-9.00	3.00	3.00	3.00
-2.00	0.00	-3.00	-2.00	-5.00	1.00	0.00	2.00
-1.00	2.00	0.00	-2.00	-4.00	1.00	2.00	1.00
0.00	0.00	-3.00	3.00	2.00	2.00	1.00	0.00
-5.00	-6.00	-10.00	-6.00	3.00	2.00	9.00	5.00
8.00	5.00	0.00	8.00	-4.00	5.00	4.00	8.00
3.00	-11.00	9.00	1.00	-25.00	1.00	5.00	3.00
7.00	9.00	-1.00	5.00	-12.00	2.00	5.00	7.00
0.00	4.00	1.00	-3.00	-4.87	2.00	7.00	0.00
-3.00	5.00	4.00	-6.00	-4.00	8.00	3.00	3.00
#NULL!	#NULL!	#NULL!	#NULL!	#NULL!	#NULL!	#NULL!	#NULL!
-9.00	-2.00	3.00	-3.00	4.00	6.00	1.00	9.00
#NULL!	#NULL!	#NULL!	#NULL!	#NULL!	#NULL!	#NULL!	#NULL!
-13.00	-16.00	2.00	5.00	-6.00	4.00	3.00	13.00
7.00	-3.00	1.00	4.00	5.00	2.00	3.00	7.00
-6.00	-1.00	-5.00	-5.00	3.00	2.00	7.00	6.00
-1.26	5.25	2.79	3.63	3.43	0.16	0.33	1.26
-6.00	9.00	7.00	4.00	1.00	2.00	0.00	6.00
8.00	-4.00	-4.00	7.00	0.00	0.00	6.00	8.00
1.89	10.00	4.00	4.00	3.00	2.00	3.00	1.89
-3.00	10.00	-4.00	-6.00	1.00	1.00	6.00	3.00
1.00	-1.00	9.00	0.00	-6.00	2.00	1.00	1.00
3.00	-11.00	9.00	5.00	-2.00	0.00	4.00	3.00
#NULL!	#NULL!	#NULL!	#NULL!	#NULL!	#NULL!	#NULL!	#NULL!
0.00	4.00	14.00	1.00	0.00	2.00	12.00	0.00
-8.00	1.00	-1.00	4.00	9.00	3.00	2.00	8.00
4.00	0.00	2.00	5.00	8.40	3.00	5.00	4.00
-5.00	-5.05	-1.00	-9.00	-3.00	2.00	7.00	5.00
-4.00	1.00	-5.00	-10.00	8.00	3.00	1.00	4.00
#NULL!	#NULL!	#NULL!	#NULL!	#NULL!	#NULL!	#NULL!	#NULL!
1.00	1.00	5.00	-2.00	2.73	1.00	4.00	1.00

-2.22	-1.00	22.16	1.00	-1.00	9.00	3.00	2.22
1.00	-5.00	-1.00	-5.00	12.00	3.00	2.00	1.00
-2.00	1.00	-1.37	5.00	-17.00	7.00	7.00	2.00
3.00	-4.00	-6.00	-1.00	9.00	0.00	6.00	3.00
-2.00	7.00	-3.00	-11.00	7.00	4.00	0.00	2.00
5.16	-3.00	-3.00	2.00	-5.53	2.00	3.00	5.16
1.00	10.00	-12.21	-8.00	20.00	2.26	3.63	1.00
#NULL!	#NULL!	#NULL!	#NULL!	#NULL!	#NULL!	#NULL!	#NULL!
0.00	-12.00	6.00	15.00	3.00	8.00	5.68	0.00
19.00	1.00	-13.00	5.00	1.00	4.00	1.00	19.00
6.00	-1.00	9.00	6.00	10.00	0.00	3.00	6.00
3.00	3.00	4.00	8.00	9.00	1.00	7.00	3.00
3.00	7.00	1.00	3.00	5.00	6.00	3.00	3.00
#NULL!	#NULL!	#NULL!	#NULL!	#NULL!	#NULL!	#NULL!	#NULL!
-4.00	-3.00	11.00	7.00	-13.00	2.00	1.00	4.00
-4.00	1.00	-7.00	1.00	4.00	2.00	1.00	4.00
0.00	-10.00	6.00	6.00	6.00	7.00	3.00	0.00
-11.00	6.00	-5.00	-4.00	-1.00	1.00	4.00	11.00
1.00	8.16	16.00	3.00	3.00	1.00	10.00	1.00
#NULL!	#NULL!	#NULL!	#NULL!	#NULL!	#NULL!	#NULL!	#NULL!
-3.00	10.00	-5.00	7.78	-1.00	5.00	7.00	3.00
-2.00	-9.00	-13.00	12.00	-5.00	7.00	7.00	2.00
-9.00	-13.00	-21.00	4.00	2.00	8.00	5.00	9.00
-10.00	11.00	7.00	-15.00	0.00	6.00	5.00	10.00
-4.00	-7.00	4.00	7.00	-1.00	11.00	6.00	4.00
-9.00	-2.00	1.00	2.00	-1.00	2.00	2.00	9.00
#NULL!	#NULL!	#NULL!	#NULL!	#NULL!	#NULL!	#NULL!	#NULL!
-1.00	-7.00	15.00	-11.00	14.00	3.00	9.00	1.00
-7.00	6.00	0.16	-6.00	3.00	0.00	3.00	7.00
3.00	-4.00	-6.00	-5.00	-15.00	4.00	0.00	3.00
9.68	-11.00	-5.00	7.68	-3.00	3.00	3.68	9.68
0.00	-8.00	-6.00	5.00	5.00	5.00	7.00	0.00
-1.00	-4.00	-8.00	-1.00	-2.00	3.00	2.84	1.00
-2.00	-2.00	3.00	-8.00	-10.00	3.00	2.00	2.00
6.00	3.00	-7.00	2.00	7.00	1.00	6.00	6.00
-1.00	14.00	-5.00	6.00	5.00	2.00	1.58	1.00
-3.00	-3.00	4.00	5.00	3.00	3.00	2.00	3.00
1.00	3.00	7.00	3.00	9.00	1.00	1.00	1.00
-4.42	-7.00	-2.42	-3.00	2.80	2.00	6.00	4.42
-0.27	0.00	-2.14	1.00	-7.20	7.67	7.38	0.27
8.00	6.00	-5.00	16.00	7.00	3.00	11.00	8.00
2.00	12.00	-11.00	4.00	10.00	7.00	6.68	2.00
-1.00	8.00	0.00	6.00	-2.00	1.00	1.21	1.00
8.00	8.00	2.00	6.00	7.00	2.00	11.00	8.00
4.00	-3.00	-1.00	7.00	3.00	6.00	3.00	4.00
0.00	-2.00	4.00	5.00	5.00	1.00	2.00	0.00
#NULL!	#NULL!	#NULL!	#NULL!	#NULL!	#NULL!	#NULL!	#NULL!
#NULL!	#NULL!	#NULL!	#NULL!	#NULL!	#NULL!	#NULL!	#NULL!
-2.00	-2.00	2.00	3.00	1.00	1.00	0.00	2.00
-6.00	6.00	-4.00	-1.00	-1.00	7.00	5.11	6.00
8.00	-2.00	2.00	1.95	-17.00	11.32	3.00	8.00
0.00	-3.00	5.00	2.00	4.00	6.00	1.05	0.00
1.00	4.00	0.00	-6.00	4.00	1.00	8.00	1.00
0.00	0.00	-1.00	-4.00	-1.00	3.00	1.00	0.00
6.00	-7.00	3.00	3.00	4.00	2.00	0.00	6.00
2.00	-9.00	1.00	-4.00	0.00	4.00	5.00	2.00

#NULL!	#NULL!	#NULL!	#NULL!	#NULL!	#NULL!	#NULL!	#NULL!
1.00	0.00	0.00	0.00	-5.00	4.00	9.00	1.00
5.00	12.00	5.37	2.00	14.00	6.00	7.00	5.00
4.00	-3.00	3.00	0.00	-2.00	1.00	8.68	4.00
4.00	-2.00	-5.00	1.00	2.00	0.00	6.21	4.00
-4.00	-2.00	15.00	-7.00	-4.00	5.11	9.00	4.00
0.00	1.00	1.00	-4.00	-14.00	2.00	1.00	0.00
9.00	14.68	23.00	6.00	10.00	0.00	9.00	9.00
-1.00	-3.00	-6.00	-1.00	2.00	10.00	14.00	1.00
0.00	-2.00	-11.00	0.00	-3.00	4.00	2.00	0.00
-7.00	3.00	-2.00	7.00	-3.00	3.00	2.00	7.00
5.00	-9.00	0.00	7.00	-14.00	3.00	6.00	5.00
6.00	-19.00	18.00	9.00	1.00	6.00	11.00	6.00
2.00	7.00	5.32	5.00	21.00	2.00	4.00	2.00
-0.33	7.00	4.00	8.37	4.93	9.00	16.44	0.33
-4.00	-5.00	17.00	15.00	-8.00	17.00	7.00	4.00
6.16	0.00	7.00	8.00	0.00	4.00	5.00	6.16
5.00	-6.00	-2.00	0.00	6.00	1.00	0.00	5.00
-2.00	-4.00	1.00	4.00	-17.00	4.00	1.00	2.00
6.00	-1.00	6.00	2.00	-2.00	2.00	1.00	6.00
5.00	1.00	-16.00	1.00	4.00	0.00	5.00	5.00
-5.00	0.00	5.00	-1.00	-9.00	6.00	3.00	5.00
8.00	8.00	-9.00	-1.00	9.00	2.00	2.00	8.00
6.00	3.00	0.00	-6.00	13.00	3.00	13.00	6.00
-3.00	-7.00	-2.00	7.00	0.00	7.68	0.00	3.00
13.00	9.00	2.00	14.00	-1.60	3.00	6.00	13.00
-2.00	-13.00	-14.00	-5.00	-3.00	1.00	2.00	2.00
-5.00	-7.00	-13.00	2.00	-10.00	5.00	3.00	5.00
6.00	2.00	5.00	6.00	1.00	4.00	12.00	6.00
-1.00	-5.00	1.00	-1.00	-2.00	1.00	2.00	1.00
-5.00	0.00	4.00	0.00	7.00	1.00	2.00	5.00
3.84	0.00	2.00	-12.00	4.33	6.00	3.00	3.84
-2.00	-6.00	-2.00	-10.00	5.00	2.00	2.00	2.00
-3.00	1.00	-14.00	-1.63	12.00	7.00	8.00	3.00
-1.00	1.68	1.00	-5.00	-1.00	5.00	0.00	1.00
-4.00	-4.00	10.00	3.00	2.00	4.00	5.00	4.00
-5.00	4.00	4.00	4.00	-4.00	4.00	1.00	5.00
1.00	13.00	27.00	-1.00	2.00	3.00	14.00	1.00
#NULL!	#NULL!	#NULL!	#NULL!	#NULL!	#NULL!	#NULL!	#NULL!
-5.00	3.00	-11.00	0.00	9.00	1.00	11.00	5.00
8.00	-3.00	3.00	2.00	6.00	3.00	5.00	8.00
0.00	6.00	-1.00	-6.00	1.00	7.00	9.00	0.00
5.00	-3.00	13.00	11.89	3.00	0.00	2.00	5.00
#NULL!	#NULL!	#NULL!	#NULL!	#NULL!	#NULL!	#NULL!	#NULL!
-2.00	-1.00	12.42	4.00	-1.00	5.00	1.00	2.00
1.00	4.00	5.00	-8.00	8.00	1.00	4.00	1.00
-5.00	0.74	-3.00	0.32	3.00	3.00	2.00	5.00
0.00	6.00	10.00	7.00	-3.00	3.00	1.00	0.00
-2.00	1.00	-1.00	-3.00	6.00	3.00	5.00	2.00
7.00	1.00	9.00	1.00	2.00	2.00	10.00	7.00
#NULL!	#NULL!	#NULL!	#NULL!	#NULL!	#NULL!	#NULL!	#NULL!
2.47	-5.37	13.00	3.00	-5.00	8.00	2.00	2.47
-3.00	-1.00	6.00	-5.00	-5.00	4.00	5.00	3.00
5.05	0.00	21.00	9.79	5.31	9.30	9.79	5.05
-4.00	1.74	-1.00	-4.00	0.00	3.00	2.00	4.00
-12.00	-3.00	2.00	-4.00	5.00	4.05	3.00	12.00

-4.00	8.00	15.00	5.00	-1.00	2.00	9.00	4.00
-1.00	-9.00	-16.00	-10.00	-3.00	12.00	7.00	1.00
-8.00	4.00	-5.00	1.00	-7.00	6.00	2.00	8.00
6.00	-1.00	-1.00	-2.00	2.00	0.00	2.00	6.00
-1.00	10.00	8.00	0.00	-1.00	2.00	5.00	1.00
9.00	12.00	4.00	8.00	6.00	0.37	5.00	9.00
-10.00	-14.00	-1.00	1.00	0.00	2.00	4.00	10.00
3.00	-9.00	-5.00	-7.00	0.00	2.00	4.00	3.00
0.00	3.00	4.00	2.00	-3.00	2.00	9.00	0.00
-4.00	7.42	-4.00	4.00	13.00	3.00	2.00	4.00
3.00	-6.00	11.00	2.00	-6.00	1.00	5.00	3.00
0.00	-9.00	-5.00	-8.00	-7.00	0.00	4.00	0.00
-6.00	4.00	2.00	-5.00	8.00	1.00	4.47	6.00
0.00	-5.21	-12.00	7.00	-1.00	0.00	13.00	0.00
0.00	6.00	0.00	-2.00	1.00	2.00	0.00	0.00
-6.00	-1.00	-4.00	0.53	8.00	15.00	9.05	6.00
-0.68	7.00	19.42	11.00	16.00	9.00	3.74	0.68
5.00	-3.00	6.00	-6.00	-10.00	0.00	3.00	5.00
-0.78	3.00	13.00	-2.00	-8.00	1.00	10.00	0.78
5.00	0.00	9.00	4.00	6.00	5.00	1.00	5.00
18.00	-5.00	-9.00	-1.00	21.00	6.00	7.00	18.00
0.00	7.00	-4.00	-1.00	1.00	3.00	6.00	0.00
#NULL!	#NULL!	#NULL!	#NULL!	#NULL!	#NULL!	#NULL!	#NULL!
-4.53	-4.42	7.00	12.71	-0.33	4.00	12.68	4.53
-4.00	4.00	-12.00	0.00	6.00	4.00	3.00	4.00
-3.00	-5.00	16.00	4.00	4.00	3.00	7.00	3.00
-3.00	-2.00	5.00	4.00	-1.00	0.00	3.00	3.00
0.00	0.00	7.00	-1.00	-16.00	7.00	1.00	0.00
-5.00	-1.00	5.00	1.00	-3.00	3.00	6.00	5.00
#NULL!	#NULL!	#NULL!	#NULL!	#NULL!	#NULL!	#NULL!	#NULL!
5.00	-3.00	2.00	-6.00	-5.00	7.00	12.00	5.00
-11.00	-26.00	-3.00	-9.68	-9.00	2.00	4.00	11.00
#NULL!	#NULL!	#NULL!	#NULL!	#NULL!	#NULL!	#NULL!	#NULL!
5.00	0.00	4.00	0.00	-2.00	1.00	0.00	5.00
7.00	15.00	-10.00	2.00	19.00	9.00	16.00	7.00
2.63	-2.37	5.26	8.00	5.13	11.21	1.82	2.63
3.00	1.00	-6.00	3.00	-6.00	2.63	6.00	3.00
8.79	5.00	3.00	4.32	3.00	1.00	11.00	8.79
-3.00	7.00	3.00	-3.00	4.00	2.00	6.00	3.00
-3.00	7.00	0.00	-2.00	6.00	3.79	1.00	3.00
5.00	-2.00	-7.00	1.00	4.80	3.00	2.26	5.00
#NULL!	#NULL!	#NULL!	#NULL!	#NULL!	#NULL!	#NULL!	#NULL!
-13.56	-9.00	5.00	6.26	-5.07	0.62	10.11	13.56
-1.00	6.00	4.00	3.00	5.00	1.00	8.00	1.00
4.00	-12.00	0.00	13.00	-5.00	1.00	4.00	4.00
-11.00	-10.00	6.00	-4.00	3.00	8.00	5.00	11.00
-7.00	-2.00	3.00	-4.00	3.00	3.00	1.05	7.00
-3.00	-1.00	-4.00	0.00	2.00	9.00	0.00	3.00
5.00	9.00	-7.00	1.00	0.00	2.00	3.00	5.00
1.00	7.00	12.00	9.00	5.00	2.00	2.00	1.00
#NULL!	#NULL!	#NULL!	#NULL!	#NULL!	#NULL!	#NULL!	#NULL!
0.33	14.00	-1.25	15.00	8.00	2.33	1.43	0.33
4.00	-5.00	2.00	3.00	-10.80	2.11	3.00	4.00
3.00	10.00	-1.00	-2.00	10.00	8.00	2.00	3.00
#NULL!	#NULL!	#NULL!	#NULL!	#NULL!	#NULL!	#NULL!	#NULL!
0.00	0.00	4.00	0.00	11.00	1.00	6.37	0.00

14.00	2.00	8.00	4.00	9.00	3.00	4.00	14.00
3.00	-1.00	-9.00	2.00	-3.00	4.00	8.00	3.00
-3.00	-4.00	3.00	2.00	-10.00	4.00	8.26	3.00
1.00	7.00	3.00	1.00	3.00	2.00	6.00	1.00
0.00	6.00	19.00	5.00	-3.00	3.00	2.00	0.00
-1.00	-9.00	-2.00	1.00	3.00	5.00	2.00	1.00
6.00	7.79	-1.68	2.00	-4.00	9.00	0.00	6.00
-1.00	-13.00	-8.00	-2.00	-5.00	2.00	1.00	1.00
3.00	-1.00	-2.00	-1.00	-9.00	4.00	5.00	3.00
#NULL!	#NULL!	#NULL!	#NULL!	#NULL!	#NULL!	#NULL!	#NULL!
#NULL!	#NULL!	#NULL!	#NULL!	#NULL!	#NULL!	#NULL!	#NULL!
#NULL!	#NULL!	#NULL!	#NULL!	#NULL!	#NULL!	#NULL!	#NULL!
#NULL!	#NULL!	#NULL!	#NULL!	#NULL!	#NULL!	#NULL!	#NULL!
#NULL!	#NULL!	#NULL!	#NULL!	#NULL!	#NULL!	#NULL!	#NULL!
#NULL!	#NULL!	#NULL!	#NULL!	#NULL!	#NULL!	#NULL!	#NULL!
#NULL!	#NULL!	#NULL!	#NULL!	#NULL!	#NULL!	#NULL!	#NULL!
#NULL!	#NULL!	#NULL!	#NULL!	#NULL!	#NULL!	#NULL!	#NULL!
#NULL!	#NULL!	#NULL!	#NULL!	#NULL!	#NULL!	#NULL!	#NULL!

PS_abs	SD_abs	CO_abs	ST_abs	tas4_P3_a	tas5_P3_a	tas10_P3_a	tas18_P3_a
0.00	5.00	2.00	9.00	2.00	3.00	2.00	1.00
2.32	1.00	5.00	3.00	1.00	0.00	0.00	0.00
1.00	1.00	2.00	5.00	1.00	3.00	1.00	1.00
#NULL!	#NULL!	#NULL!	#NULL!	#NULL!	#NULL!	#NULL!	#NULL!
10.00	24.00	12.00	1.00	#NULL!	#NULL!	#NULL!	#NULL!
#NULL!	#NULL!	#NULL!	#NULL!	#NULL!	#NULL!	#NULL!	#NULL!
10.00	15.00	1.00	6.00	3.00	2.00	2.00	1.00
9.00	4.89	13.00	17.00	1.00	1.00	1.00	1.00
16.00	5.33	35.00	8.00	1.00	1.00	0.00	4.00
#NULL!	#NULL!	#NULL!	#NULL!	#NULL!	#NULL!	#NULL!	#NULL!
1.00	0.00	3.00	3.57	1.00	1.00	0.00	0.00
3.05	7.00	2.00	2.00	1.00	2.00	1.00	1.00
5.00	1.00	0.00	2.00	1.00	1.00	1.00	0.00
#NULL!	#NULL!	#NULL!	#NULL!	#NULL!	#NULL!	#NULL!	#NULL!
4.00	1.00	0.00	1.00	1.00	1.00	1.00	1.00
2.00	3.00	6.00	5.00	#NULL!	#NULL!	#NULL!	#NULL!
0.00	0.00	2.00	5.00	1.00	1.00	1.00	1.00
5.00	2.00	2.00	0.00	4.00	4.00	2.00	4.00
3.00	4.26	1.00	8.00	0.00	0.00	#NULL!	2.00
8.00	2.00	2.00	9.00	4.00	4.00	1.00	1.00
2.00	1.00	2.00	3.00	1.00	0.00	1.00	1.00
3.00	9.00	5.00	5.00	0.00	0.00	0.00	0.00
3.00	8.00	2.42	11.00	3.00	1.00	2.00	2.00
4.00	14.00	3.00	17.00	2.00	2.00	2.00	2.00
1.00	4.00	8.00	7.00	1.00	3.00	0.00	3.00
6.00	3.00	4.00	3.00	0.00	1.00	0.00	1.00
3.00	5.00	3.00	1.00	0.00	0.00	1.00	0.00
1.00	3.00	5.00	11.00	0.00	1.00	0.00	1.00
#NULL!	#NULL!	#NULL!	#NULL!	#NULL!	#NULL!	#NULL!	#NULL!
2.00	7.00	1.00	5.00	1.00	0.00	4.00	4.00
1.00	0.00	1.00	8.00	2.00	4.00	1.00	2.00
2.00	3.00	11.00	5.00	1.00	2.00	1.00	3.00
1.00	11.00	3.00	4.00	1.00	1.00	1.00	1.00
5.00	2.00	16.00	7.00	1.00	2.00	3.00	1.00
#NULL!	#NULL!	#NULL!	#NULL!	#NULL!	#NULL!	#NULL!	#NULL!
#NULL!	#NULL!	#NULL!	#NULL!	#NULL!	#NULL!	#NULL!	#NULL!
#NULL!	#NULL!	#NULL!	#NULL!	#NULL!	#NULL!	#NULL!	#NULL!
#NULL!	#NULL!	#NULL!	#NULL!	#NULL!	#NULL!	#NULL!	#NULL!
#NULL!	#NULL!	#NULL!	#NULL!	#NULL!	#NULL!	#NULL!	#NULL!
1.00	3.58	3.00	3.00	2.00	1.00	0.00	1.00
8.00	11.00	21.00	2.00	2.00	2.00	0.00	0.00
1.00	7.00	0.00	1.00	1.00	0.00	1.00	1.00
0.00	6.00	2.00	4.00	0.00	1.00	0.00	1.00
9.00	3.00	2.00	5.00	#NULL!	#NULL!	#NULL!	#NULL!
#NULL!	#NULL!	#NULL!	#NULL!	#NULL!	#NULL!	#NULL!	#NULL!
11.00	11.00	11.00	8.00	0.00	4.00	1.00	4.00
1.00	7.00	3.00	5.00	1.00	1.00	0.00	0.00
4.00	15.00	1.00	8.00	#NULL!	#NULL!	#NULL!	#NULL!
4.00	3.00	6.00	1.00	1.00	0.00	0.00	0.00
1.00	0.00	14.00	2.00	0.00	1.00	0.00	0.00
7.00	11.00	3.00	6.00	2.00	1.00	1.00	1.00
7.00	10.00	5.00	3.00	#NULL!	#NULL!	#NULL!	#NULL!
2.00	3.00	1.00	3.00	0.00	1.00	0.00	0.00
5.00	0.00	5.00	7.00	0.00	1.00	0.00	1.00
0.00	0.00	6.00	13.00	#NULL!	#NULL!	#NULL!	#NULL!

2.00	1.00	3.00	6.00	2.00	1.00	2.00	1.00
2.00	20.00	12.00	16.00	4.00	4.00	0.00	1.00
16.00	3.00	3.89	3.00	4.00	2.00	1.00	1.00
8.00	3.00	2.00	3.00	4.00	4.00	0.00	0.00
0.00	0.00	0.00	8.00	1.00	1.00	0.00	4.00
3.00	0.32	5.00	4.00	#NULL!	#NULL!	#NULL!	#NULL!
9.00	10.00	11.00	4.00	0.00	0.00	1.00	2.00
6.00	4.00	7.00	0.00	0.00	1.00	1.00	0.00
1.00	7.00	3.00	7.00	1.00	1.00	1.00	1.00
3.00	0.00	4.21	1.00	3.00	3.00	2.00	1.00
4.00	4.00	7.00	1.00	0.00	2.00	1.00	2.00
1.16	6.00	4.74	12.00	1.00	4.00	1.00	4.00
0.00	2.00	3.00	0.00	3.00	0.00	0.00	0.00
2.00	5.37	2.00	0.00	2.00	2.00	2.00	2.00
2.00	1.00	10.00	2.00	2.00	2.00	2.00	2.00
10.00	2.00	10.00	2.00	1.00	1.00	0.00	0.00
6.00	4.00	1.00	11.00	1.00	2.00	1.00	1.00
1.00	2.00	4.00	1.00	1.00	1.00	1.00	1.00
8.00	2.00	3.00	1.00	1.00	2.00	2.00	2.00
1.89	5.00	2.79	8.00	2.00	3.00	1.00	1.00
5.00	6.00	7.00	2.00	0.00	4.00	0.00	0.00
2.00	5.00	7.00	13.00	3.00	1.00	1.00	1.00
8.00	6.00	2.00	3.00	#NULL!	#NULL!	#NULL!	#NULL!
22.44	6.00	11.00	1.00	#NULL!	#NULL!	#NULL!	#NULL!
1.00	4.00	1.00	6.00	4.00	4.00	0.00	0.00
2.00	0.00	9.00	9.00	0.00	2.00	2.00	1.00
0.00	3.00	2.00	5.00	1.00	1.00	2.00	1.00
2.00	0.00	2.00	4.00	4.00	2.00	0.00	1.00
0.00	3.00	3.00	2.00	3.00	3.00	2.00	1.00
6.00	10.00	6.00	3.00	1.00	2.00	1.00	1.00
5.00	0.00	8.00	4.00	0.00	1.00	0.00	1.00
11.00	9.00	1.00	25.00	1.00	0.00	0.00	0.00
9.00	1.00	5.00	12.00	4.00	1.00	1.00	0.00
4.00	1.00	3.00	4.87	1.00	3.00	1.00	1.00
5.00	4.00	6.00	4.00	0.00	2.00	0.00	1.00
#NULL!	#NULL!	#NULL!	#NULL!	#NULL!	#NULL!	#NULL!	#NULL!
2.00	3.00	3.00	4.00	1.00	1.00	3.00	1.00
#NULL!	#NULL!	#NULL!	#NULL!	#NULL!	#NULL!	#NULL!	#NULL!
16.00	2.00	5.00	6.00	#NULL!	#NULL!	#NULL!	#NULL!
3.00	1.00	4.00	5.00	2.00	1.00	1.00	1.00
1.00	5.00	5.00	3.00	1.00	2.00	0.00	4.00
5.25	2.79	3.63	3.43	2.00	3.00	2.00	1.00
9.00	7.00	4.00	1.00	1.00	1.00	1.00	1.00
4.00	4.00	7.00	0.00	3.00	0.00	0.00	0.00
10.00	4.00	4.00	3.00	2.00	1.00	1.00	2.00
10.00	4.00	6.00	1.00	0.00	1.00	4.00	0.00
1.00	9.00	0.00	6.00	1.00	1.00	2.00	3.00
11.00	9.00	5.00	2.00	2.00	3.00	1.00	0.00
#NULL!	#NULL!	#NULL!	#NULL!	#NULL!	#NULL!	#NULL!	#NULL!
4.00	14.00	1.00	0.00	1.00	3.00	2.00	1.00
1.00	1.00	4.00	9.00	#NULL!	#NULL!	#NULL!	#NULL!
0.00	2.00	5.00	8.40	1.00	1.00	1.00	2.00
5.05	1.00	9.00	3.00	1.00	2.00	4.00	2.00
1.00	5.00	10.00	8.00	1.00	3.00	0.00	0.00
#NULL!	#NULL!	#NULL!	#NULL!	#NULL!	#NULL!	#NULL!	#NULL!
1.00	5.00	2.00	2.73	1.00	2.00	2.00	0.00

1.00	22.16	1.00	1.00	3.00	2.00	1.00	1.00
5.00	1.00	5.00	12.00	1.00	1.00	1.00	1.00
1.00	1.37	5.00	17.00	0.00	1.00	2.00	0.00
4.00	6.00	1.00	9.00	#NULL!	#NULL!	#NULL!	#NULL!
7.00	3.00	11.00	7.00	1.00	2.00	1.00	1.00
3.00	3.00	2.00	5.53	0.00	0.00	1.00	0.00
10.00	12.21	8.00	20.00	3.00	1.00	1.00	3.00
#NULL!	#NULL!	#NULL!	#NULL!	#NULL!	#NULL!	#NULL!	#NULL!
12.00	6.00	15.00	3.00	#NULL!	#NULL!	#NULL!	#NULL!
1.00	13.00	5.00	1.00	3.00	0.00	0.00	0.00
1.00	9.00	6.00	10.00	1.00	1.00	0.00	1.00
3.00	4.00	8.00	9.00	1.00	1.00	0.00	1.00
7.00	1.00	3.00	5.00	1.00	3.00	3.00	0.00
#NULL!	#NULL!	#NULL!	#NULL!	#NULL!	#NULL!	#NULL!	#NULL!
3.00	11.00	7.00	13.00	0.00	1.00	1.00	1.00
1.00	7.00	1.00	4.00	#NULL!	#NULL!	#NULL!	#NULL!
10.00	6.00	6.00	6.00	1.00	2.00	1.00	0.00
6.00	5.00	4.00	1.00	#NULL!	#NULL!	#NULL!	#NULL!
8.16	16.00	3.00	3.00	0.00	0.00	1.00	0.00
#NULL!	#NULL!	#NULL!	#NULL!	#NULL!	#NULL!	#NULL!	#NULL!
10.00	5.00	7.78	1.00	#NULL!	#NULL!	#NULL!	#NULL!
9.00	13.00	12.00	5.00	2.00	2.00	1.00	0.00
13.00	21.00	4.00	2.00	#NULL!	#NULL!	#NULL!	#NULL!
11.00	7.00	15.00	0.00	1.00	3.00	4.00	3.00
7.00	4.00	7.00	1.00	2.00	1.00	1.00	1.00
2.00	1.00	2.00	1.00	2.00	1.00	2.00	1.00
#NULL!	#NULL!	#NULL!	#NULL!	#NULL!	#NULL!	#NULL!	#NULL!
7.00	15.00	11.00	14.00	1.00	1.00	1.00	1.00
6.00	0.16	6.00	3.00	#NULL!	#NULL!	#NULL!	#NULL!
4.00	6.00	5.00	15.00	#NULL!	#NULL!	#NULL!	#NULL!
11.00	5.00	7.68	3.00	#NULL!	#NULL!	#NULL!	#NULL!
8.00	6.00	5.00	5.00	1.00	1.00	1.00	0.00
4.00	8.00	1.00	2.00	2.00	2.00	0.00	0.00
2.00	3.00	8.00	10.00	3.00	2.00	2.00	1.00
3.00	7.00	2.00	7.00	2.00	1.00	1.00	2.00
14.00	5.00	6.00	5.00	0.00	1.00	0.00	0.00
3.00	4.00	5.00	3.00	#NULL!	#NULL!	#NULL!	#NULL!
3.00	7.00	3.00	9.00	2.00	1.00	1.00	1.00
7.00	2.42	3.00	2.80	3.00	0.00	3.00	2.00
0.00	2.14	1.00	7.20	#NULL!	#NULL!	#NULL!	#NULL!
6.00	5.00	16.00	7.00	2.00	0.00	0.00	0.00
12.00	11.00	4.00	10.00	0.00	1.00	0.00	1.00
8.00	0.00	6.00	2.00	1.00	1.00	0.00	2.00
8.00	2.00	6.00	7.00	1.00	1.00	1.00	1.00
3.00	1.00	7.00	3.00	1.00	1.00	1.00	1.00
2.00	4.00	5.00	5.00	0.00	0.00	1.00	1.00
#NULL!	#NULL!	#NULL!	#NULL!	#NULL!	#NULL!	#NULL!	#NULL!
#NULL!	#NULL!	#NULL!	#NULL!	#NULL!	#NULL!	#NULL!	#NULL!
2.00	2.00	3.00	1.00	1.00	0.00	#NULL!	0.00
6.00	4.00	1.00	1.00	0.00	0.00	0.00	0.00
2.00	2.00	1.95	17.00	1.00	3.00	2.00	1.00
3.00	5.00	2.00	4.00	#NULL!	#NULL!	#NULL!	#NULL!
4.00	0.00	6.00	4.00	4.00	2.00	1.00	0.00
0.00	1.00	4.00	1.00	1.00	1.00	1.00	1.00
7.00	3.00	3.00	4.00	1.00	1.00	0.00	0.00
9.00	1.00	4.00	0.00	0.00	1.00	0.00	0.00

#NULL!	#NULL!	#NULL!	#NULL!	#NULL!	#NULL!	#NULL!	#NULL!
0.00	0.00	0.00	5.00	0.00	1.00	0.00	2.00
12.00	5.37	2.00	14.00	0.00	0.00	0.00	4.00
3.00	3.00	0.00	2.00	3.00	1.00	1.00	4.00
2.00	5.00	1.00	2.00	#NULL!	#NULL!	#NULL!	#NULL!
2.00	15.00	7.00	4.00	0.00	0.00	0.00	0.00
1.00	1.00	4.00	14.00	0.00	3.00	0.00	3.00
14.68	23.00	6.00	10.00	0.00	0.00	0.00	0.00
3.00	6.00	1.00	2.00	1.00	3.00	4.00	4.00
2.00	11.00	0.00	3.00	1.00	1.00	1.00	1.00
3.00	2.00	7.00	3.00	1.00	1.00	2.00	2.00
9.00	0.00	7.00	14.00	1.00	2.00	0.00	1.00
19.00	18.00	9.00	1.00	3.00	0.00	0.00	2.00
7.00	5.32	5.00	21.00	1.00	1.00	1.00	1.00
7.00	4.00	8.37	4.93	1.00	1.00	3.00	0.00
5.00	17.00	15.00	8.00	#NULL!	#NULL!	#NULL!	#NULL!
0.00	7.00	8.00	0.00	1.00	2.00	1.00	1.00
6.00	2.00	0.00	6.00	0.00	1.00	0.00	1.00
4.00	1.00	4.00	17.00	3.00	0.00	0.00	0.00
1.00	6.00	2.00	2.00	#NULL!	#NULL!	#NULL!	#NULL!
1.00	16.00	1.00	4.00	0.00	1.00	0.00	0.00
0.00	5.00	1.00	9.00	#NULL!	#NULL!	#NULL!	#NULL!
8.00	9.00	1.00	9.00	1.00	1.00	1.00	0.00
3.00	0.00	6.00	13.00	1.00	1.00	1.00	0.00
7.00	2.00	7.00	0.00	0.00	1.00	0.00	2.00
9.00	2.00	14.00	1.60	0.00	2.00	0.00	0.00
13.00	14.00	5.00	3.00	1.00	1.00	0.00	1.00
7.00	13.00	2.00	10.00	0.00	3.00	1.00	0.00
2.00	5.00	6.00	1.00	3.00	1.00	0.00	1.00
5.00	1.00	1.00	2.00	1.00	2.00	0.00	0.00
0.00	4.00	0.00	7.00	4.00	1.00	0.00	0.00
0.00	2.00	12.00	4.33	#NULL!	#NULL!	#NULL!	#NULL!
6.00	2.00	10.00	5.00	3.00	0.00	2.00	1.00
1.00	14.00	1.63	12.00	#NULL!	#NULL!	#NULL!	#NULL!
1.68	1.00	5.00	1.00	4.00	2.00	2.00	0.00
4.00	10.00	3.00	2.00	1.00	1.00	1.00	1.00
4.00	4.00	4.00	4.00	#NULL!	#NULL!	#NULL!	#NULL!
13.00	27.00	1.00	2.00	2.00	3.00	0.00	1.00
#NULL!	#NULL!	#NULL!	#NULL!	#NULL!	#NULL!	#NULL!	#NULL!
3.00	11.00	0.00	9.00	1.00	1.00	0.00	2.00
3.00	3.00	2.00	6.00	#NULL!	#NULL!	#NULL!	#NULL!
6.00	1.00	6.00	1.00	1.00	2.00	1.00	1.00
3.00	13.00	11.89	3.00	2.00	4.00	4.00	0.00
#NULL!	#NULL!	#NULL!	#NULL!	#NULL!	#NULL!	#NULL!	#NULL!
1.00	12.42	4.00	1.00	#NULL!	#NULL!	#NULL!	#NULL!
4.00	5.00	8.00	8.00	#NULL!	#NULL!	#NULL!	#NULL!
0.74	3.00	0.32	3.00	0.00	3.00	0.00	1.00
6.00	10.00	7.00	3.00	1.00	1.00	1.00	1.00
1.00	1.00	3.00	6.00	3.00	3.00	0.00	0.00
1.00	9.00	1.00	2.00	#NULL!	#NULL!	#NULL!	#NULL!
#NULL!	#NULL!	#NULL!	#NULL!	#NULL!	#NULL!	#NULL!	#NULL!
5.37	13.00	3.00	5.00	3.00	2.00	1.00	#NULL!
1.00	6.00	5.00	5.00	1.00	2.00	2.00	2.00
0.00	21.00	9.79	5.31	0.00	3.00	2.00	1.00
1.74	1.00	4.00	0.00	#NULL!	#NULL!	#NULL!	#NULL!
3.00	2.00	4.00	5.00	0.00	2.00	1.00	0.00

8.00	15.00	5.00	1.00	3.00	3.00	1.00	1.00
9.00	16.00	10.00	3.00	1.00	1.00	1.00	1.00
4.00	5.00	1.00	7.00	2.00	1.00	1.00	1.00
1.00	1.00	2.00	2.00	2.00	1.00	1.00	1.00
10.00	8.00	0.00	1.00	1.00	2.00	1.00	1.00
12.00	4.00	8.00	6.00	2.00	2.00	1.00	1.00
14.00	1.00	1.00	0.00	0.00	1.00	0.00	1.00
9.00	5.00	7.00	0.00	#NULL!	#NULL!	#NULL!	#NULL!
3.00	4.00	2.00	3.00	1.00	2.00	0.00	1.00
7.42	4.00	4.00	13.00	2.00	2.00	2.00	0.00
6.00	11.00	2.00	6.00	1.00	3.00	0.00	1.00
9.00	5.00	8.00	7.00	#NULL!	#NULL!	#NULL!	#NULL!
4.00	2.00	5.00	8.00	#NULL!	#NULL!	#NULL!	#NULL!
5.21	12.00	7.00	1.00	1.00	2.00	1.00	1.00
6.00	0.00	2.00	1.00	2.00	2.00	2.00	1.00
1.00	4.00	0.53	8.00	1.00	0.00	1.00	1.00
7.00	19.42	11.00	16.00	3.00	2.00	#NULL!	2.00
3.00	6.00	6.00	10.00	2.00	3.00	2.00	2.00
3.00	13.00	2.00	8.00	0.00	0.00	0.00	0.00
0.00	9.00	4.00	6.00	0.00	0.00	3.00	0.00
5.00	9.00	1.00	21.00	0.00	2.00	0.00	0.00
7.00	4.00	1.00	1.00	1.00	1.00	1.00	1.00
#NULL!	#NULL!	#NULL!	#NULL!	#NULL!	#NULL!	#NULL!	#NULL!
4.42	7.00	12.71	0.33	1.00	1.00	2.00	1.00
4.00	12.00	0.00	6.00	2.00	2.00	1.00	1.00
5.00	16.00	4.00	4.00	4.00	4.00	2.00	4.00
2.00	5.00	4.00	1.00	3.00	2.00	2.00	1.00
0.00	7.00	1.00	16.00	2.00	3.00	2.00	2.00
1.00	5.00	1.00	3.00	0.00	0.00	0.00	1.00
#NULL!	#NULL!	#NULL!	#NULL!	#NULL!	#NULL!	#NULL!	#NULL!
3.00	2.00	6.00	5.00	3.00	1.00	2.00	2.00
26.00	3.00	9.68	9.00	1.00	0.00	0.00	0.00
#NULL!	#NULL!	#NULL!	#NULL!	#NULL!	#NULL!	#NULL!	#NULL!
0.00	4.00	0.00	2.00	3.00	2.00	1.00	3.00
15.00	10.00	2.00	19.00	4.00	3.00	3.00	2.00
2.37	5.26	8.00	5.13	#NULL!	#NULL!	#NULL!	#NULL!
1.00	6.00	3.00	6.00	3.00	3.00	4.00	1.00
5.00	3.00	4.32	3.00	0.00	4.00	4.00	0.00
7.00	3.00	3.00	4.00	1.00	1.00	2.00	1.00
7.00	0.00	2.00	6.00	0.00	1.00	1.00	1.00
2.00	7.00	1.00	4.80	4.00	3.00	1.00	0.00
#NULL!	#NULL!	#NULL!	#NULL!	#NULL!	#NULL!	#NULL!	#NULL!
9.00	5.00	6.26	5.07	2.00	2.00	1.00	2.00
6.00	4.00	3.00	5.00	#NULL!	#NULL!	#NULL!	#NULL!
12.00	0.00	13.00	5.00	1.00	1.00	1.00	0.00
10.00	6.00	4.00	3.00	#NULL!	#NULL!	#NULL!	#NULL!
2.00	3.00	4.00	3.00	3.00	3.00	3.00	2.00
1.00	4.00	0.00	2.00	2.00	1.00	1.00	0.00
9.00	7.00	1.00	0.00	1.00	0.00	1.00	1.00
7.00	12.00	9.00	5.00	1.00	2.00	0.00	0.00
#NULL!	#NULL!	#NULL!	#NULL!	#NULL!	#NULL!	#NULL!	#NULL!
14.00	1.25	15.00	8.00	1.00	1.00	0.00	0.00
5.00	2.00	3.00	10.80	2.00	3.00	2.00	1.00
10.00	1.00	2.00	10.00	#NULL!	#NULL!	#NULL!	#NULL!
#NULL!	#NULL!	#NULL!	#NULL!	#NULL!	#NULL!	#NULL!	#NULL!
0.00	4.00	0.00	11.00	2.00	3.00	1.00	1.00

2.00	8.00	4.00	9.00	1.00	2.00	0.00	1.00
1.00	9.00	2.00	3.00	1.00	2.00	1.00	1.00
4.00	3.00	2.00	10.00	1.00	0.00	0.00	1.00
7.00	3.00	1.00	3.00	1.00	0.00	0.00	0.00
6.00	19.00	5.00	3.00	#NULL!	#NULL!	#NULL!	#NULL!
9.00	2.00	1.00	3.00	#NULL!	#NULL!	#NULL!	#NULL!
7.79	1.68	2.00	4.00	0.00	0.00	3.00	4.00
13.00	8.00	2.00	5.00	2.00	2.00	0.00	1.00
1.00	2.00	1.00	9.00	0.00	0.00	0.00	0.00
#NULL!	#NULL!	#NULL!	#NULL!	4.00	4.00	2.00	1.00
#NULL!	#NULL!	#NULL!	#NULL!	1.00	4.00	2.00	3.00
#NULL!	#NULL!	#NULL!	#NULL!	0.00	1.00	0.00	0.00
#NULL!	#NULL!	#NULL!	#NULL!	1.00	1.00	1.00	1.00
#NULL!	#NULL!	#NULL!	#NULL!	2.00	0.00	1.00	0.00
#NULL!	#NULL!	#NULL!	#NULL!	0.00	0.00	1.00	1.00
#NULL!	#NULL!	#NULL!	#NULL!	2.00	1.00	1.00	2.00
#NULL!	#NULL!	#NULL!	#NULL!	#NULL!	#NULL!	#NULL!	#NULL!
#NULL!	#NULL!	#NULL!	#NULL!	#NULL!	#NULL!	#NULL!	#NULL!

tas19_P3_a	age_p3	age_group	d1_product	d2_product	d1_oness	d2_oness	d11_oness
2.00	63.00	1.00	180.00	200.00	1.00	1.00	1.00
0.00	65.00	1.00	120.00	17.00	0.00	0.00	0.00
1.00	63.00	1.00	169.00	104.00	1.00	0.00	1.00
#NULL!	#NULL!	#NULL!	54.00	48.00	0.00	0.00	0.00
#NULL!	63.00	1.00	12.00	70.00	0.00	0.00	0.00
#NULL!	50.00	1.00	1.00	0.00	0.00	0.00	0.00
2.00	49.00	1.00	27.00	14.00	0.00	0.00	0.00
1.00	78.00	1.00	20.00	35.00	0.00	0.00	0.00
4.00	59.00	1.00	42.00	60.00	0.00	0.00	0.00
#NULL!	#NULL!	#NULL!	0.00	4.00	0.00	0.00	0.00
0.00	68.00	1.00	12.00	44.00	0.00	0.00	0.00
1.00	69.00	1.00	64.00	40.00	0.00	0.00	0.00
0.00	52.00	1.00	48.00	20.00	0.00	0.00	0.00
#NULL!	#NULL!	#NULL!	0.00	0.00	0.00	0.00	0.00
2.00	82.00	1.00	80.00	208.00	0.00	1.00	0.00
#NULL!	52.00	1.00	80.00	12.00	0.00	0.00	0.00
1.00	70.00	1.00	48.00	27.00	0.00	0.00	0.00
2.00	72.00	1.00	102.00	0.00	0.00	0.00	0.00
2.00	87.00	1.00	120.00	240.00	0.00	1.00	0.00
3.00	72.00	1.00	225.00	150.00	1.00	1.00	1.00
2.00	51.00	1.00	12.00	6.00	0.00	0.00	0.00
0.00	68.00	1.00	35.00	0.00	0.00	0.00	0.00
2.00	59.00	1.00	44.00	0.00	0.00	0.00	0.00
2.00	46.00	1.00	49.00	108.00	0.00	0.00	0.00
0.00	64.00	1.00	143.00	96.00	1.00	0.00	1.00
0.00	65.00	1.00	25.00	4.00	0.00	0.00	0.00
0.00	62.00	1.00	0.00	0.00	0.00	0.00	0.00
1.00	51.00	1.00	98.00	66.00	0.00	0.00	0.00
#NULL!	68.00	1.00	60.00	63.00	0.00	0.00	0.00
2.00	61.00	1.00	54.00	54.00	0.00	0.00	0.00
2.00	68.00	1.00	16.00	72.00	0.00	0.00	0.00
1.00	74.00	1.00	20.00	10.00	0.00	0.00	0.00
1.00	55.00	1.00	224.00	210.00	1.00	1.00	1.00
3.00	63.00	1.00	114.00	91.00	0.00	0.00	0.00
#NULL!	#NULL!	#NULL!	#NULL!	42.00	0.00	0.00	0.00
#NULL!	#NULL!	#NULL!	#NULL!	18.00	0.00	0.00	0.00
#NULL!	#NULL!	#NULL!	#NULL!	399.00	0.00	1.00	0.00
#NULL!	#NULL!	#NULL!	#NULL!	54.00	0.00	0.00	0.00
#NULL!	68.00	1.00	40.00	27.00	0.00	0.00	0.00
1.00	62.00	1.00	54.00	63.00	0.00	0.00	0.00
2.00	95.00	1.00	42.00	0.00	0.00	0.00	0.00
1.00	80.00	1.00	9.00	24.00	0.00	0.00	0.00
2.00	64.00	1.00	88.00	48.00	0.00	0.00	0.00
#NULL!	69.00	1.00	36.00	0.00	0.00	0.00	0.00
#NULL!	#NULL!	#NULL!	14.00	0.00	0.00	0.00	0.00
3.00	46.00	1.00	15.00	3.00	0.00	0.00	0.00
0.00	64.00	1.00	63.00	6.00	0.00	0.00	0.00
#NULL!	59.00	1.00	40.00	196.00	0.00	1.00	0.00
1.00	58.00	1.00	143.00	224.00	1.00	1.00	1.00
0.00	60.00	1.00	0.00	12.00	0.00	0.00	0.00
0.00	68.00	1.00	12.00	30.00	0.00	0.00	0.00
#NULL!	66.00	1.00	66.00	10.00	0.00	0.00	0.00
0.00	66.00	1.00	20.00	0.00	0.00	0.00	0.00
0.00	72.00	1.00	88.00	77.00	0.00	0.00	0.00
#NULL!	62.00	1.00	110.00	156.00	0.00	1.00	0.00

2.00	69.00	1.00	126.00	68.00	0.00	0.00	0.00
0.00	63.00	1.00	24.00	28.00	0.00	0.00	0.00
1.00	49.00	1.00	56.00	0.00	0.00	0.00	0.00
4.00	68.00	1.00	255.00	140.00	1.00	1.00	1.00
4.00	68.00	1.00	120.00	35.00	0.00	0.00	0.00
#NULL!	67.00	1.00	55.00	18.00	0.00	0.00	0.00
2.00	69.00	1.00	57.00	24.00	0.00	0.00	0.00
1.00	65.00	1.00	56.00	0.00	0.00	0.00	0.00
2.00	46.00	1.00	56.00	153.00	0.00	1.00	0.00
2.00	78.00	1.00	143.00	224.00	1.00	1.00	1.00
2.00	76.00	1.00	5.00	0.00	0.00	0.00	0.00
4.00	75.00	1.00	132.00	96.00	1.00	0.00	0.00
0.00	77.00	1.00	35.00	8.00	0.00	0.00	0.00
2.00	77.00	1.00	44.00	119.00	0.00	0.00	0.00
2.00	62.00	1.00	306.00	352.00	1.00	1.00	1.00
0.00	46.00	1.00	135.00	40.00	1.00	0.00	1.00
1.00	60.00	1.00	33.00	45.00	0.00	0.00	0.00
1.00	57.00	1.00	36.00	26.00	0.00	0.00	0.00
2.00	56.00	1.00	98.00	96.00	0.00	0.00	0.00
1.00	53.00	1.00	99.00	84.00	0.00	0.00	0.00
4.00	55.00	1.00	22.00	20.00	0.00	0.00	0.00
1.00	65.00	1.00	54.00	15.00	0.00	0.00	0.00
#NULL!	47.00	1.00	72.00	169.00	0.00	1.00	0.00
#NULL!	56.00	1.00	90.00	224.00	0.00	1.00	0.00
0.00	60.00	1.00	288.00	210.00	1.00	1.00	1.00
1.00	61.00	1.00	28.00	30.00	0.00	0.00	0.00
2.00	61.00	1.00	143.00	91.00	1.00	0.00	1.00
0.00	52.00	1.00	0.00	5.00	0.00	0.00	0.00
2.00	78.00	1.00	208.00	150.00	1.00	1.00	1.00
1.00	51.00	1.00	21.00	60.00	0.00	0.00	0.00
1.00	55.00	1.00	2.00	0.00	0.00	0.00	0.00
0.00	58.00	1.00	170.00	160.00	1.00	1.00	1.00
0.00	53.00	1.00	104.00	38.00	0.00	0.00	0.00
1.00	59.00	1.00	40.00	15.00	0.00	0.00	0.00
1.00	61.00	1.00	12.00	0.00	0.00	0.00	0.00
#NULL!	#NULL!	#NULL!	24.00	9.00	0.00	0.00	0.00
2.00	57.00	1.00	24.00	117.00	0.00	0.00	0.00
#NULL!	#NULL!	#NULL!	120.00	112.00	0.00	0.00	0.00
#NULL!	49.00	1.00	588.00	625.00	1.00	1.00	1.00
1.00	70.00	1.00	14.00	0.00	0.00	0.00	0.00
1.00	67.00	1.00	32.00	45.00	0.00	0.00	0.00
3.00	59.00	1.00	70.00	30.00	0.00	0.00	0.00
1.00	59.00	1.00	147.00	156.00	1.00	1.00	1.00
0.00	70.00	1.00	24.00	16.00	0.00	0.00	0.00
1.00	54.00	1.00	272.00	112.00	1.00	0.00	1.00
2.00	75.00	1.00	156.00	320.00	1.00	1.00	1.00
1.00	60.00	1.00	132.00	204.00	1.00	1.00	0.00
3.00	52.00	1.00	96.00	132.00	0.00	1.00	0.00
#NULL!	#NULL!	#NULL!	0.00	0.00	0.00	0.00	0.00
1.00	50.00	1.00	90.00	90.00	0.00	0.00	0.00
#NULL!	73.00	1.00	0.00	0.00	0.00	0.00	0.00
1.00	50.00	1.00	0.00	4.00	0.00	0.00	0.00
3.00	62.00	1.00	22.00	24.00	0.00	0.00	0.00
0.00	49.00	1.00	55.00	132.00	0.00	1.00	0.00
#NULL!	61.00	1.00	#NULL!	#NULL!	0.00	0.00	0.00
2.00	64.00	1.00	119.00	160.00	0.00	1.00	0.00

0.00	64.00	1.00	12.00	80.00	0.00	0.00	0.00
1.00	72.00	1.00	64.00	27.00	0.00	0.00	0.00
3.00	48.00	1.00	35.00	210.00	0.00	1.00	0.00
#NULL!	62.00	1.00	189.00	189.00	1.00	1.00	1.00
1.00	63.00	1.00	56.00	8.00	0.00	0.00	0.00
#NULL!	87.00	1.00	44.00	33.00	0.00	0.00	0.00
1.00	64.00	1.00	35.00	84.00	0.00	0.00	0.00
#NULL!	47.00	1.00	15.00	5.00	0.00	0.00	0.00
#NULL!	51.00	1.00	84.00	21.00	0.00	0.00	0.00
1.00	47.00	1.00	252.00	323.00	1.00	1.00	1.00
0.00	58.00	1.00	39.00	28.00	0.00	0.00	0.00
2.00	61.00	1.00	240.00	90.00	1.00	0.00	1.00
1.00	69.00	1.00	88.00	32.00	0.00	0.00	0.00
#NULL!	54.00	1.00	0.00	0.00	0.00	0.00	0.00
1.00	57.00	1.00	60.00	24.00	0.00	0.00	0.00
#NULL!	76.00	1.00	224.00	324.00	1.00	1.00	1.00
1.00	62.00	1.00	0.00	0.00	0.00	0.00	0.00
#NULL!	58.00	1.00	24.00	0.00	0.00	0.00	0.00
0.00	73.00	1.00	39.00	48.00	0.00	0.00	0.00
#NULL!	66.00	1.00	132.00	120.00	1.00	0.00	0.00
#NULL!	59.00	1.00	110.00	120.00	0.00	0.00	0.00
3.00	49.00	1.00	64.00	140.00	0.00	1.00	0.00
#NULL!	59.00	1.00	240.00	320.00	1.00	1.00	1.00
2.00	46.00	1.00	81.00	35.00	0.00	0.00	0.00
1.00	64.00	1.00	190.00	40.00	1.00	0.00	1.00
1.00	46.00	1.00	228.00	378.00	1.00	1.00	1.00
#NULL!	59.00	1.00	20.00	36.00	0.00	0.00	0.00
1.00	57.00	1.00	162.00	36.00	1.00	0.00	1.00
#NULL!	67.00	1.00	0.00	0.00	0.00	0.00	0.00
#NULL!	55.00	1.00	240.00	299.00	1.00	1.00	1.00
#NULL!	56.00	1.00	117.00	112.00	0.00	0.00	0.00
0.00	45.00	1.00	3.00	8.00	0.00	0.00	0.00
0.00	70.00	1.00	121.00	91.00	0.00	0.00	0.00
2.00	66.00	1.00	75.00	176.00	0.00	1.00	0.00
2.00	73.00	1.00	132.00	150.00	1.00	1.00	0.00
0.00	73.00	1.00	39.00	18.00	0.00	0.00	0.00
#NULL!	78.00	1.00	12.00	20.00	0.00	0.00	0.00
1.00	55.00	1.00	272.00	132.00	1.00	1.00	1.00
1.00	65.00	1.00	152.00	160.00	1.00	1.00	1.00
#NULL!	58.00	1.00	60.00	110.00	0.00	0.00	0.00
0.00	71.00	1.00	72.00	24.00	0.00	0.00	0.00
1.00	52.00	1.00	198.00	330.00	1.00	1.00	1.00
2.00	47.00	1.00	252.00	234.00	1.00	1.00	1.00
2.00	55.00	1.00	144.00	48.00	1.00	0.00	1.00
0.00	67.00	1.00	40.00	16.00	0.00	0.00	0.00
1.00	67.00	1.00	150.00	49.00	1.00	0.00	1.00
#NULL!	#NULL!	#NULL!	120.00	198.00	0.00	1.00	0.00
#NULL!	76.00	1.00	16.00	70.00	0.00	0.00	0.00
2.00	55.00	1.00	96.00	80.00	0.00	0.00	0.00
3.00	58.00	1.00	30.00	0.00	0.00	0.00	0.00
2.00	74.00	1.00	28.00	12.00	0.00	0.00	0.00
#NULL!	69.00	1.00	143.00	80.00	1.00	0.00	1.00
1.00	53.00	1.00	2.00	7.00	0.00	0.00	0.00
1.00	57.00	1.00	36.00	40.00	0.00	0.00	0.00
1.00	54.00	1.00	144.00	98.00	1.00	0.00	1.00
1.00	53.00	1.00	65.00	117.00	0.00	0.00	0.00

#NULL!	#NULL!	#NULL!	143.00	98.00	1.00	0.00	1.00
0.00	50.00	1.00	36.00	0.00	0.00	0.00	0.00
0.00	55.00	1.00	21.00	0.00	0.00	0.00	0.00
4.00	69.00	1.00	224.00	342.00	1.00	1.00	1.00
#NULL!	55.00	1.00	119.00	161.00	0.00	1.00	0.00
0.00	70.00	1.00	63.00	28.00	0.00	0.00	0.00
3.00	56.00	1.00	40.00	35.00	0.00	0.00	0.00
0.00	48.00	1.00	48.00	12.00	0.00	0.00	0.00
4.00	69.00	1.00	135.00	208.00	1.00	1.00	1.00
1.00	66.00	1.00	99.00	51.00	0.00	0.00	0.00
1.00	77.00	1.00	72.00	140.00	0.00	1.00	0.00
2.00	48.00	1.00	126.00	150.00	0.00	1.00	0.00
3.00	59.00	1.00	270.00	54.00	1.00	0.00	1.00
1.00	51.00	1.00	66.00	84.00	0.00	0.00	0.00
4.00	61.00	1.00	323.00	342.00	1.00	1.00	1.00
#NULL!	67.00	1.00	560.00	216.00	1.00	1.00	1.00
2.00	69.00	1.00	160.00	210.00	1.00	1.00	1.00
1.00	69.00	1.00	234.00	135.00	1.00	1.00	1.00
1.00	68.00	1.00	78.00	104.00	0.00	0.00	0.00
#NULL!	77.00	1.00	36.00	0.00	0.00	0.00	0.00
0.00	75.00	1.00	5.00	10.00	0.00	0.00	0.00
#NULL!	67.00	1.00	49.00	55.00	0.00	0.00	0.00
2.00	49.00	1.00	20.00	0.00	0.00	0.00	0.00
1.00	52.00	1.00	132.00	56.00	1.00	0.00	0.00
1.00	69.00	1.00	54.00	6.00	0.00	0.00	0.00
0.00	68.00	1.00	8.00	25.00	0.00	0.00	0.00
0.00	54.00	1.00	21.00	56.00	0.00	0.00	0.00
0.00	60.00	1.00	5.00	0.00	0.00	0.00	0.00
0.00	69.00	1.00	210.00	70.00	1.00	0.00	1.00
1.00	53.00	1.00	112.00	96.00	0.00	0.00	0.00
1.00	46.00	1.00	21.00	39.00	0.00	0.00	0.00
#NULL!	80.00	1.00	299.00	384.00	1.00	1.00	1.00
1.00	79.00	1.00	247.00	484.00	1.00	1.00	1.00
#NULL!	61.00	1.00	30.00	6.00	0.00	0.00	0.00
2.00	61.00	1.00	182.00	176.00	1.00	1.00	1.00
1.00	75.00	1.00	77.00	108.00	0.00	0.00	0.00
#NULL!	66.00	1.00	72.00	36.00	0.00	0.00	0.00
1.00	45.00	1.00	400.00	270.00	1.00	1.00	1.00
#NULL!	57.00	1.00	624.00	504.00	1.00	1.00	1.00
2.00	70.00	1.00	84.00	88.00	0.00	0.00	0.00
#NULL!	78.00	1.00	108.00	64.00	0.00	0.00	0.00
2.00	59.00	1.00	64.00	15.00	0.00	0.00	0.00
3.00	68.00	1.00	357.00	322.00	1.00	1.00	1.00
#NULL!	#NULL!	#NULL!	11.00	65.00	0.00	0.00	0.00
#NULL!	59.00	1.00	32.00	42.00	0.00	0.00	0.00
#NULL!	55.00	1.00	136.00	176.00	1.00	1.00	1.00
1.00	60.00	1.00	9.00	6.00	0.00	0.00	0.00
1.00	75.00	1.00	165.00	14.00	1.00	0.00	1.00
0.00	45.00	1.00	56.00	5.00	0.00	0.00	0.00
#NULL!	54.00	1.00	165.00	180.00	1.00	1.00	1.00
#NULL!	59.00	1.00	14.00	39.00	0.00	0.00	0.00
1.00	63.00	1.00	39.00	60.00	0.00	0.00	0.00
2.00	73.00	1.00	56.00	60.00	0.00	0.00	0.00
1.00	51.00	1.00	140.00	96.00	1.00	0.00	1.00
#NULL!	57.00	1.00	77.00	8.00	0.00	0.00	0.00
3.00	50.00	1.00	72.00	42.00	0.00	0.00	0.00

1.00	70.00	1.00	340.00	240.00	1.00	1.00	1.00
1.00	57.00	1.00	5.00	10.00	0.00	0.00	0.00
1.00	50.00	1.00	126.00	136.00	0.00	1.00	0.00
1.00	71.00	1.00	60.00	99.00	0.00	0.00	0.00
1.00	51.00	1.00	182.00	304.00	1.00	1.00	1.00
2.00	68.00	1.00	192.00	144.00	1.00	1.00	1.00
4.00	66.00	1.00	5.00	8.00	0.00	0.00	0.00
#NULL!	77.00	1.00	54.00	16.00	0.00	0.00	0.00
3.00	56.00	1.00	99.00	45.00	0.00	0.00	0.00
2.00	55.00	1.00	110.00	63.00	0.00	0.00	0.00
1.00	56.00	1.00	0.00	0.00	0.00	0.00	0.00
#NULL!	76.00	1.00	84.00	48.00	0.00	0.00	0.00
#NULL!	59.00	1.00	30.00	65.00	0.00	0.00	0.00
2.00	54.00	1.00	153.00	440.00	1.00	1.00	1.00
2.00	47.00	1.00	143.00	169.00	1.00	1.00	1.00
1.00	64.00	1.00	104.00	154.00	0.00	1.00	0.00
0.00	56.00	1.00	288.00	138.00	1.00	1.00	1.00
3.00	54.00	1.00	105.00	72.00	0.00	0.00	0.00
0.00	85.00	1.00	95.00	120.00	0.00	0.00	0.00
0.00	52.00	1.00	0.00	6.00	0.00	0.00	0.00
1.00	59.00	1.00	8.00	0.00	0.00	0.00	0.00
1.00	55.00	1.00	1.00	6.00	0.00	0.00	0.00
#NULL!	#NULL!	#NULL!	56.00	18.00	0.00	0.00	0.00
#NULL!	56.00	1.00	84.00	44.00	0.00	0.00	0.00
1.00	59.00	1.00	32.00	64.00	0.00	0.00	0.00
2.00	66.00	1.00	420.00	588.00	1.00	1.00	1.00
2.00	53.00	1.00	112.00	84.00	0.00	0.00	0.00
3.00	50.00	1.00	143.00	12.00	1.00	0.00	1.00
0.00	63.00	1.00	99.00	154.00	0.00	1.00	0.00
#NULL!	#NULL!	#NULL!	90.00	15.00	0.00	0.00	0.00
1.00	48.00	1.00	352.00	360.00	1.00	1.00	1.00
1.00	70.00	1.00	8.00	16.00	0.00	0.00	0.00
#NULL!	#NULL!	#NULL!	104.00	200.00	0.00	1.00	0.00
2.00	73.00	1.00	30.00	0.00	0.00	0.00	0.00
1.00	62.00	1.00	40.00	30.00	0.00	0.00	0.00
#NULL!	63.00	1.00	30.00	65.00	0.00	0.00	0.00
2.00	76.00	1.00	18.00	64.00	0.00	0.00	0.00
0.00	66.00	1.00	6.00	3.00	0.00	0.00	0.00
1.00	70.00	1.00	45.00	54.00	0.00	0.00	0.00
1.00	65.00	1.00	16.00	52.00	0.00	0.00	0.00
2.00	74.00	1.00	48.00	27.00	0.00	0.00	0.00
#NULL!	58.00	1.00	187.00	253.00	1.00	1.00	1.00
2.00	77.00	1.00	176.00	7.00	1.00	0.00	1.00
#NULL!	68.00	1.00	8.00	24.00	0.00	0.00	0.00
0.00	56.00	1.00	160.00	110.00	1.00	0.00	1.00
#NULL!	86.00	1.00	36.00	70.00	0.00	0.00	0.00
3.00	80.00	1.00	84.00	104.00	0.00	0.00	0.00
2.00	66.00	1.00	18.00	21.00	0.00	0.00	0.00
1.00	54.00	1.00	112.00	136.00	0.00	1.00	0.00
0.00	53.00	1.00	120.00	90.00	0.00	0.00	0.00
#NULL!	61.00	1.00	9.00	126.00	0.00	0.00	0.00
1.00	72.00	1.00	108.00	264.00	0.00	1.00	0.00
2.00	76.00	1.00	80.00	91.00	0.00	0.00	0.00
#NULL!	61.00	1.00	162.00	110.00	1.00	0.00	1.00
#NULL!	60.00	1.00	378.00	306.00	1.00	1.00	1.00
1.00	65.00	1.00	54.00	30.00	0.00	0.00	0.00

0.00	62.00	1.00	100.00	9.00	0.00	0.00	0.00
0.00	59.00	1.00	80.00	18.00	0.00	0.00	0.00
1.00	72.00	1.00	20.00	12.00	0.00	0.00	0.00
0.00	67.00	1.00	48.00	8.00	0.00	0.00	0.00
#NULL!	50.00	1.00	150.00	135.00	1.00	1.00	1.00
#NULL!	53.00	1.00	28.00	12.00	0.00	0.00	0.00
4.00	59.00	1.00	0.00	0.00	0.00	0.00	0.00
2.00	66.00	1.00	102.00	132.00	0.00	1.00	0.00
1.00	48.00	1.00	160.00	72.00	1.00	0.00	1.00
1.00	70.00	1.00	#NULL!	196.00	0.00	1.00	0.00
3.00	72.00	1.00	#NULL!	156.00	0.00	1.00	0.00
3.00	72.00	1.00	#NULL!	0.00	0.00	0.00	0.00
1.00	56.00	1.00	#NULL!	12.00	0.00	0.00	0.00
0.00	69.00	1.00	#NULL!	49.00	0.00	0.00	0.00
1.00	61.00	1.00	#NULL!	#NULL!	0.00	0.00	0.00
1.00	52.00	1.00	#NULL!	#NULL!	0.00	0.00	0.00
#NULL!	#NULL!	#NULL!	#NULL!	588.00	0.00	1.00	0.00
#NULL!	#NULL!	#NULL!	#NULL!	8.00	0.00	0.00	0.00

d22_oness	d120_oness	d2120_oness	d121_oness	d2121_oness	d126_oness	d2126_oness	d132_oness
1.00	1.00	1.00	1.00	1.00	1.00	1.00	1.00
0.00	1.00	0.00	0.00	0.00	0.00	0.00	0.00
0.00	1.00	0.00	1.00	0.00	1.00	0.00	1.00
0.00	0.00	0.00	0.00	0.00	0.00	0.00	0.00
0.00	0.00	0.00	0.00	0.00	0.00	0.00	0.00
0.00	0.00	0.00	0.00	0.00	0.00	0.00	0.00
0.00	0.00	0.00	0.00	0.00	0.00	0.00	0.00
0.00	0.00	0.00	0.00	0.00	0.00	0.00	0.00
0.00	0.00	0.00	0.00	0.00	0.00	0.00	0.00
0.00	0.00	0.00	0.00	0.00	0.00	0.00	0.00
0.00	0.00	0.00	0.00	0.00	0.00	0.00	0.00
0.00	0.00	0.00	0.00	0.00	0.00	0.00	0.00
0.00	0.00	0.00	0.00	0.00	0.00	0.00	0.00
0.00	0.00	0.00	0.00	0.00	0.00	0.00	0.00
1.00	0.00	1.00	0.00	1.00	0.00	1.00	0.00
0.00	0.00	0.00	0.00	0.00	0.00	0.00	0.00
0.00	0.00	0.00	0.00	0.00	0.00	0.00	0.00
0.00	0.00	0.00	0.00	0.00	0.00	0.00	0.00
1.00	1.00	1.00	0.00	1.00	0.00	1.00	0.00
1.00	1.00	1.00	1.00	1.00	1.00	1.00	1.00
0.00	0.00	0.00	0.00	0.00	0.00	0.00	0.00
0.00	0.00	0.00	0.00	0.00	0.00	0.00	0.00
0.00	0.00	0.00	0.00	0.00	0.00	0.00	0.00
0.00	0.00	0.00	0.00	0.00	0.00	0.00	0.00
0.00	1.00	0.00	1.00	0.00	1.00	0.00	1.00
0.00	0.00	0.00	0.00	0.00	0.00	0.00	0.00
0.00	0.00	0.00	0.00	0.00	0.00	0.00	0.00
0.00	0.00	0.00	0.00	0.00	0.00	0.00	0.00
0.00	0.00	0.00	0.00	0.00	0.00	0.00	0.00
0.00	0.00	0.00	0.00	0.00	0.00	0.00	0.00
0.00	0.00	0.00	0.00	0.00	0.00	0.00	0.00
0.00	0.00	0.00	0.00	0.00	0.00	0.00	0.00
1.00	1.00	1.00	1.00	1.00	1.00	1.00	1.00
0.00	0.00	0.00	0.00	0.00	0.00	0.00	0.00
0.00	0.00	0.00	0.00	0.00	0.00	0.00	0.00
0.00	0.00	0.00	0.00	0.00	0.00	0.00	0.00
1.00	0.00	1.00	0.00	1.00	0.00	1.00	0.00
0.00	0.00	0.00	0.00	0.00	0.00	0.00	0.00
0.00	0.00	0.00	0.00	0.00	0.00	0.00	0.00
0.00	0.00	0.00	0.00	0.00	0.00	0.00	0.00
0.00	0.00	0.00	0.00	0.00	0.00	0.00	0.00
0.00	0.00	0.00	0.00	0.00	0.00	0.00	0.00
0.00	0.00	0.00	0.00	0.00	0.00	0.00	0.00
0.00	0.00	0.00	0.00	0.00	0.00	0.00	0.00
0.00	0.00	0.00	0.00	0.00	0.00	0.00	0.00
0.00	0.00	0.00	0.00	0.00	0.00	0.00	0.00
0.00	0.00	0.00	0.00	0.00	0.00	0.00	0.00
1.00	0.00	1.00	0.00	1.00	0.00	1.00	0.00
1.00	1.00	1.00	1.00	1.00	1.00	1.00	1.00
0.00	0.00	0.00	0.00	0.00	0.00	0.00	0.00
0.00	0.00	0.00	0.00	0.00	0.00	0.00	0.00
0.00	0.00	0.00	0.00	0.00	0.00	0.00	0.00
0.00	0.00	0.00	0.00	0.00	0.00	0.00	0.00
0.00	0.00	0.00	0.00	0.00	0.00	0.00	0.00
1.00	0.00	1.00	0.00	1.00	0.00	1.00	0.00

0.00	1.00	0.00	1.00	0.00	1.00	0.00	0.00
0.00	0.00	0.00	0.00	0.00	0.00	0.00	0.00
0.00	0.00	0.00	0.00	0.00	0.00	0.00	0.00
1.00	1.00	1.00	1.00	1.00	1.00	1.00	1.00
0.00	1.00	0.00	0.00	0.00	0.00	0.00	0.00
0.00	0.00	0.00	0.00	0.00	0.00	0.00	0.00
0.00	0.00	0.00	0.00	0.00	0.00	0.00	0.00
0.00	0.00	0.00	0.00	0.00	0.00	0.00	0.00
1.00	0.00	1.00	0.00	1.00	0.00	1.00	0.00
1.00	1.00	1.00	1.00	1.00	1.00	1.00	1.00
0.00	0.00	0.00	0.00	0.00	0.00	0.00	0.00
0.00	1.00	0.00	1.00	0.00	1.00	0.00	1.00
0.00	0.00	0.00	0.00	0.00	0.00	0.00	0.00
0.00	0.00	0.00	0.00	0.00	0.00	0.00	0.00
1.00	1.00	1.00	1.00	1.00	1.00	1.00	1.00
0.00	1.00	0.00	1.00	0.00	1.00	0.00	1.00
0.00	0.00	0.00	0.00	0.00	0.00	0.00	0.00
0.00	0.00	0.00	0.00	0.00	0.00	0.00	0.00
0.00	0.00	0.00	0.00	0.00	0.00	0.00	0.00
0.00	0.00	0.00	0.00	0.00	0.00	0.00	0.00
0.00	0.00	0.00	0.00	0.00	0.00	0.00	0.00
0.00	0.00	0.00	0.00	0.00	0.00	0.00	0.00
1.00	0.00	1.00	0.00	1.00	0.00	1.00	0.00
1.00	0.00	1.00	0.00	1.00	0.00	1.00	0.00
1.00	1.00	1.00	1.00	1.00	1.00	1.00	1.00
0.00	0.00	0.00	0.00	0.00	0.00	0.00	0.00
0.00	1.00	0.00	1.00	0.00	1.00	0.00	1.00
0.00	0.00	0.00	0.00	0.00	0.00	0.00	0.00
1.00	1.00	1.00	1.00	1.00	1.00	1.00	1.00
0.00	0.00	0.00	0.00	0.00	0.00	0.00	0.00
0.00	0.00	0.00	0.00	0.00	0.00	0.00	0.00
1.00	1.00	1.00	1.00	1.00	1.00	1.00	1.00
0.00	0.00	0.00	0.00	0.00	0.00	0.00	0.00
0.00	0.00	0.00	0.00	0.00	0.00	0.00	0.00
0.00	0.00	0.00	0.00	0.00	0.00	0.00	0.00
0.00	0.00	0.00	0.00	0.00	0.00	0.00	0.00
0.00	0.00	0.00	0.00	0.00	0.00	0.00	0.00
0.00	1.00	0.00	0.00	0.00	0.00	0.00	0.00
1.00	1.00	1.00	1.00	1.00	1.00	1.00	1.00
0.00	0.00	0.00	0.00	0.00	0.00	0.00	0.00
0.00	0.00	0.00	0.00	0.00	0.00	0.00	0.00
0.00	0.00	0.00	0.00	0.00	0.00	0.00	0.00
1.00	1.00	1.00	1.00	1.00	1.00	1.00	1.00
0.00	0.00	0.00	0.00	0.00	0.00	0.00	0.00
0.00	1.00	0.00	1.00	0.00	1.00	0.00	1.00
1.00	1.00	1.00	1.00	1.00	1.00	1.00	1.00
1.00	1.00	1.00	1.00	1.00	1.00	1.00	1.00
0.00	0.00	1.00	0.00	1.00	0.00	1.00	0.00
0.00	0.00	0.00	0.00	0.00	0.00	0.00	0.00
0.00	0.00	0.00	0.00	0.00	0.00	0.00	0.00
0.00	0.00	0.00	0.00	0.00	0.00	0.00	0.00
0.00	0.00	0.00	0.00	0.00	0.00	0.00	0.00
0.00	0.00	0.00	0.00	0.00	0.00	0.00	0.00
0.00	0.00	1.00	0.00	1.00	0.00	1.00	0.00
0.00	0.00	0.00	0.00	0.00	0.00	0.00	0.00
1.00	0.00	1.00	0.00	1.00	0.00	1.00	0.00

0.00	0.00	0.00	0.00	0.00	0.00	0.00	0.00
0.00	0.00	0.00	0.00	0.00	0.00	0.00	0.00
1.00	0.00	1.00	0.00	1.00	0.00	1.00	0.00
1.00	1.00	1.00	1.00	1.00	1.00	1.00	1.00
0.00	0.00	0.00	0.00	0.00	0.00	0.00	0.00
0.00	0.00	0.00	0.00	0.00	0.00	0.00	0.00
0.00	0.00	0.00	0.00	0.00	0.00	0.00	0.00
0.00	0.00	0.00	0.00	0.00	0.00	0.00	0.00
0.00	0.00	0.00	0.00	0.00	0.00	0.00	0.00
1.00	1.00	1.00	1.00	1.00	1.00	1.00	1.00
0.00	0.00	0.00	0.00	0.00	0.00	0.00	0.00
0.00	1.00	0.00	1.00	0.00	1.00	0.00	1.00
0.00	0.00	0.00	0.00	0.00	0.00	0.00	0.00
0.00	0.00	0.00	0.00	0.00	0.00	0.00	0.00
0.00	0.00	0.00	0.00	0.00	0.00	0.00	0.00
1.00	1.00	1.00	1.00	1.00	1.00	1.00	1.00
0.00	0.00	0.00	0.00	0.00	0.00	0.00	0.00
0.00	0.00	0.00	0.00	0.00	0.00	0.00	0.00
0.00	0.00	0.00	0.00	0.00	0.00	0.00	0.00
0.00	1.00	1.00	1.00	0.00	1.00	0.00	1.00
0.00	0.00	1.00	0.00	0.00	0.00	0.00	0.00
1.00	0.00	1.00	0.00	1.00	0.00	1.00	0.00
1.00	1.00	1.00	1.00	1.00	1.00	1.00	1.00
0.00	0.00	0.00	0.00	0.00	0.00	0.00	0.00
0.00	1.00	0.00	1.00	0.00	1.00	0.00	1.00
1.00	1.00	1.00	1.00	1.00	1.00	1.00	1.00
0.00	0.00	0.00	0.00	0.00	0.00	0.00	0.00
0.00	1.00	0.00	1.00	0.00	1.00	0.00	1.00
0.00	0.00	0.00	0.00	0.00	0.00	0.00	0.00
1.00	1.00	1.00	1.00	1.00	1.00	1.00	1.00
0.00	0.00	0.00	0.00	0.00	0.00	0.00	0.00
0.00	0.00	0.00	0.00	0.00	0.00	0.00	0.00
0.00	1.00	0.00	1.00	0.00	0.00	0.00	0.00
1.00	0.00	1.00	0.00	1.00	0.00	1.00	0.00
1.00	1.00	1.00	1.00	1.00	1.00	1.00	1.00
0.00	0.00	0.00	0.00	0.00	0.00	0.00	0.00
0.00	0.00	0.00	0.00	0.00	0.00	0.00	0.00
0.00	1.00	1.00	1.00	1.00	1.00	1.00	1.00
1.00	1.00	1.00	1.00	1.00	1.00	1.00	1.00
0.00	0.00	0.00	0.00	0.00	0.00	0.00	0.00
0.00	0.00	0.00	0.00	0.00	0.00	0.00	0.00
1.00	1.00	1.00	1.00	1.00	1.00	1.00	1.00
1.00	1.00	1.00	1.00	1.00	1.00	1.00	1.00
0.00	1.00	0.00	1.00	0.00	1.00	0.00	1.00
0.00	0.00	0.00	0.00	0.00	0.00	0.00	0.00
0.00	1.00	0.00	1.00	0.00	1.00	0.00	1.00
1.00	1.00	1.00	0.00	1.00	0.00	1.00	0.00
0.00	0.00	0.00	0.00	0.00	0.00	0.00	0.00
0.00	0.00	0.00	0.00	0.00	0.00	0.00	0.00
0.00	0.00	0.00	0.00	0.00	0.00	0.00	0.00
0.00	0.00	0.00	0.00	0.00	0.00	0.00	0.00
0.00	1.00	0.00	1.00	0.00	1.00	0.00	1.00
0.00	0.00	0.00	0.00	0.00	0.00	0.00	0.00
0.00	0.00	0.00	0.00	0.00	0.00	0.00	0.00
0.00	1.00	0.00	1.00	0.00	1.00	0.00	1.00
0.00	0.00	0.00	0.00	0.00	0.00	0.00	0.00

0.00	1.00	0.00	1.00	0.00	1.00	0.00	1.00
0.00	0.00	0.00	0.00	0.00	0.00	0.00	0.00
0.00	0.00	0.00	0.00	0.00	0.00	0.00	0.00
1.00	1.00	1.00	1.00	1.00	1.00	1.00	1.00
1.00	0.00	1.00	0.00	1.00	0.00	1.00	0.00
0.00	0.00	0.00	0.00	0.00	0.00	0.00	0.00
0.00	0.00	0.00	0.00	0.00	0.00	0.00	0.00
0.00	0.00	0.00	0.00	0.00	0.00	0.00	0.00
1.00	1.00	1.00	1.00	1.00	1.00	1.00	1.00
0.00	0.00	0.00	0.00	0.00	0.00	0.00	0.00
1.00	0.00	1.00	0.00	1.00	0.00	1.00	0.00
1.00	1.00	1.00	1.00	1.00	1.00	1.00	0.00
0.00	1.00	0.00	1.00	0.00	1.00	0.00	1.00
0.00	0.00	0.00	0.00	0.00	0.00	0.00	0.00
1.00	1.00	1.00	1.00	1.00	1.00	1.00	1.00
1.00	1.00	1.00	1.00	1.00	1.00	1.00	1.00
1.00	1.00	1.00	1.00	1.00	1.00	1.00	1.00
1.00	1.00	1.00	1.00	1.00	1.00	1.00	1.00
0.00	0.00	0.00	0.00	0.00	0.00	0.00	0.00
0.00	0.00	0.00	0.00	0.00	0.00	0.00	0.00
0.00	0.00	0.00	0.00	0.00	0.00	0.00	0.00
0.00	0.00	0.00	0.00	0.00	0.00	0.00	0.00
0.00	0.00	0.00	0.00	0.00	0.00	0.00	0.00
0.00	1.00	0.00	1.00	0.00	1.00	0.00	1.00
0.00	0.00	0.00	0.00	0.00	0.00	0.00	0.00
0.00	0.00	0.00	0.00	0.00	0.00	0.00	0.00
0.00	0.00	0.00	0.00	0.00	0.00	0.00	0.00
0.00	0.00	0.00	0.00	0.00	0.00	0.00	0.00
0.00	1.00	0.00	1.00	0.00	1.00	0.00	1.00
0.00	0.00	0.00	0.00	0.00	0.00	0.00	0.00
0.00	0.00	0.00	0.00	0.00	0.00	0.00	0.00
1.00	1.00	1.00	1.00	1.00	1.00	1.00	1.00
1.00	1.00	1.00	1.00	1.00	1.00	1.00	1.00
0.00	0.00	0.00	0.00	0.00	0.00	0.00	0.00
1.00	1.00	1.00	1.00	1.00	1.00	1.00	1.00
0.00	0.00	0.00	0.00	0.00	0.00	0.00	0.00
0.00	0.00	0.00	0.00	0.00	0.00	0.00	0.00
1.00	1.00	1.00	1.00	1.00	1.00	1.00	1.00
1.00	1.00	1.00	1.00	1.00	1.00	1.00	1.00
0.00	0.00	0.00	0.00	0.00	0.00	0.00	0.00
0.00	0.00	0.00	0.00	0.00	0.00	0.00	0.00
0.00	0.00	0.00	0.00	0.00	0.00	0.00	0.00
1.00	1.00	1.00	1.00	1.00	1.00	1.00	1.00
0.00	0.00	0.00	0.00	0.00	0.00	0.00	0.00
0.00	0.00	0.00	0.00	0.00	0.00	0.00	0.00
1.00	1.00	1.00	1.00	1.00	1.00	1.00	1.00
0.00	0.00	0.00	0.00	0.00	0.00	0.00	0.00
0.00	1.00	0.00	1.00	0.00	1.00	0.00	1.00
0.00	0.00	0.00	0.00	0.00	0.00	0.00	0.00
1.00	1.00	1.00	1.00	1.00	1.00	1.00	1.00
0.00	0.00	0.00	0.00	0.00	0.00	0.00	0.00
0.00	0.00	0.00	0.00	0.00	0.00	0.00	0.00
0.00	0.00	0.00	0.00	0.00	0.00	0.00	0.00
0.00	1.00	0.00	1.00	0.00	1.00	0.00	1.00
0.00	0.00	0.00	0.00	0.00	0.00	0.00	0.00
0.00	0.00	0.00	0.00	0.00	0.00	0.00	0.00

1.00	1.00	1.00	1.00	1.00	1.00	1.00	1.00
0.00	0.00	0.00	0.00	0.00	0.00	0.00	0.00
1.00	1.00	1.00	1.00	1.00	1.00	1.00	0.00
0.00	0.00	0.00	0.00	0.00	0.00	0.00	0.00
1.00	1.00	1.00	1.00	1.00	1.00	1.00	1.00
1.00	1.00	1.00	1.00	1.00	1.00	1.00	1.00
0.00	0.00	0.00	0.00	0.00	0.00	0.00	0.00
0.00	0.00	0.00	0.00	0.00	0.00	0.00	0.00
0.00	0.00	0.00	0.00	0.00	0.00	0.00	0.00
0.00	0.00	0.00	0.00	0.00	0.00	0.00	0.00
0.00	0.00	0.00	0.00	0.00	0.00	0.00	0.00
0.00	0.00	0.00	0.00	0.00	0.00	0.00	0.00
0.00	0.00	0.00	0.00	0.00	0.00	0.00	0.00
1.00	1.00	1.00	1.00	1.00	1.00	1.00	1.00
1.00	1.00	1.00	1.00	1.00	1.00	1.00	1.00
1.00	0.00	1.00	0.00	1.00	0.00	1.00	0.00
1.00	1.00	1.00	1.00	1.00	1.00	1.00	1.00
0.00	0.00	0.00	0.00	0.00	0.00	0.00	0.00
0.00	0.00	1.00	0.00	0.00	0.00	0.00	0.00
0.00	0.00	0.00	0.00	0.00	0.00	0.00	0.00
0.00	0.00	0.00	0.00	0.00	0.00	0.00	0.00
0.00	0.00	0.00	0.00	0.00	0.00	0.00	0.00
0.00	0.00	0.00	0.00	0.00	0.00	0.00	0.00
0.00	0.00	0.00	0.00	0.00	0.00	0.00	0.00
0.00	0.00	0.00	0.00	0.00	0.00	0.00	0.00
1.00	1.00	1.00	1.00	1.00	1.00	1.00	1.00
0.00	0.00	0.00	0.00	0.00	0.00	0.00	0.00
0.00	1.00	0.00	1.00	0.00	1.00	0.00	1.00
1.00	0.00	1.00	0.00	1.00	0.00	1.00	0.00
0.00	0.00	0.00	0.00	0.00	0.00	0.00	0.00
1.00	1.00	1.00	1.00	1.00	1.00	1.00	1.00
0.00	0.00	0.00	0.00	0.00	0.00	0.00	0.00
1.00	0.00	1.00	0.00	1.00	0.00	1.00	0.00
0.00	0.00	0.00	0.00	0.00	0.00	0.00	0.00
0.00	0.00	0.00	0.00	0.00	0.00	0.00	0.00
0.00	0.00	0.00	0.00	0.00	0.00	0.00	0.00
0.00	0.00	0.00	0.00	0.00	0.00	0.00	0.00
0.00	0.00	0.00	0.00	0.00	0.00	0.00	0.00
0.00	0.00	0.00	0.00	0.00	0.00	0.00	0.00
0.00	0.00	0.00	0.00	0.00	0.00	0.00	0.00
0.00	0.00	0.00	0.00	0.00	0.00	0.00	0.00
1.00	1.00	1.00	1.00	1.00	1.00	1.00	1.00
0.00	1.00	0.00	1.00	0.00	1.00	0.00	1.00
0.00	0.00	0.00	0.00	0.00	0.00	0.00	0.00
0.00	1.00	0.00	1.00	0.00	1.00	0.00	1.00
0.00	0.00	0.00	0.00	0.00	0.00	0.00	0.00
0.00	0.00	0.00	0.00	0.00	0.00	0.00	0.00
0.00	0.00	0.00	0.00	0.00	0.00	0.00	0.00
1.00	0.00	1.00	0.00	1.00	0.00	1.00	0.00
0.00	1.00	0.00	0.00	0.00	0.00	0.00	0.00
0.00	0.00	1.00	0.00	1.00	0.00	1.00	0.00
1.00	0.00	1.00	0.00	1.00	0.00	1.00	0.00
0.00	0.00	0.00	0.00	0.00	0.00	0.00	0.00
0.00	1.00	0.00	1.00	0.00	1.00	0.00	1.00
1.00	1.00	1.00	1.00	1.00	1.00	1.00	1.00
0.00	0.00	0.00	0.00	0.00	0.00	0.00	0.00

0.00	0.00	0.00	0.00	0.00	0.00	0.00	0.00
0.00	0.00	0.00	0.00	0.00	0.00	0.00	0.00
0.00	0.00	0.00	0.00	0.00	0.00	0.00	0.00
0.00	0.00	0.00	0.00	0.00	0.00	0.00	0.00
1.00	1.00	1.00	1.00	1.00	1.00	1.00	1.00
0.00	0.00	0.00	0.00	0.00	0.00	0.00	0.00
0.00	0.00	0.00	0.00	0.00	0.00	0.00	0.00
0.00	0.00	1.00	0.00	1.00	0.00	1.00	0.00
0.00	1.00	0.00	1.00	0.00	1.00	0.00	1.00
1.00	0.00	1.00	0.00	1.00	0.00	1.00	0.00
1.00	0.00	1.00	0.00	1.00	0.00	1.00	0.00
0.00	0.00	0.00	0.00	0.00	0.00	0.00	0.00
0.00	0.00	0.00	0.00	0.00	0.00	0.00	0.00
0.00	0.00	0.00	0.00	0.00	0.00	0.00	0.00
0.00	0.00	0.00	0.00	0.00	0.00	0.00	0.00
0.00	0.00	0.00	0.00	0.00	0.00	0.00	0.00
1.00	0.00	1.00	0.00	1.00	0.00	1.00	0.00
0.00	0.00	0.00	0.00	0.00	0.00	0.00	0.00

d2132_oness	d135_oness	d2135_oness	d136_oness	d2136_oness	d140_oness	d2140_oness
1.00	1.00	1.00	1.00	1.00	0.00	1.00
0.00	0.00	0.00	0.00	0.00	0.00	0.00
0.00	1.00	0.00	1.00	0.00	0.00	0.00
0.00	0.00	0.00	0.00	0.00	0.00	0.00
0.00	0.00	0.00	0.00	0.00	0.00	0.00
0.00	0.00	0.00	0.00	0.00	0.00	0.00
0.00	0.00	0.00	0.00	0.00	0.00	0.00
0.00	0.00	0.00	0.00	0.00	0.00	0.00
0.00	0.00	0.00	0.00	0.00	0.00	0.00
0.00	0.00	0.00	0.00	0.00	0.00	0.00
0.00	0.00	0.00	0.00	0.00	0.00	0.00
0.00	0.00	0.00	0.00	0.00	0.00	0.00
0.00	0.00	0.00	0.00	0.00	0.00	0.00
0.00	0.00	0.00	0.00	0.00	0.00	0.00
1.00	0.00	1.00	0.00	1.00	0.00	1.00
0.00	0.00	0.00	0.00	0.00	0.00	0.00
0.00	0.00	0.00	0.00	0.00	0.00	0.00
0.00	0.00	0.00	0.00	0.00	0.00	0.00
1.00	0.00	1.00	0.00	1.00	0.00	1.00
1.00	1.00	1.00	1.00	1.00	0.00	1.00
0.00	0.00	0.00	0.00	0.00	0.00	0.00
0.00	0.00	0.00	0.00	0.00	0.00	0.00
0.00	0.00	0.00	0.00	0.00	0.00	0.00
0.00	0.00	0.00	0.00	0.00	0.00	0.00
0.00	1.00	0.00	1.00	0.00	0.00	0.00
0.00	0.00	0.00	0.00	0.00	0.00	0.00
0.00	0.00	0.00	0.00	0.00	0.00	0.00
0.00	0.00	0.00	0.00	0.00	0.00	0.00
0.00	0.00	0.00	0.00	0.00	0.00	0.00
0.00	0.00	0.00	0.00	0.00	0.00	0.00
0.00	0.00	0.00	0.00	0.00	0.00	0.00
0.00	0.00	0.00	0.00	0.00	0.00	0.00
1.00	1.00	1.00	1.00	1.00	0.00	1.00
0.00	0.00	0.00	0.00	0.00	0.00	0.00
0.00	0.00	0.00	0.00	0.00	0.00	0.00
0.00	0.00	0.00	0.00	0.00	0.00	0.00
1.00	0.00	1.00	0.00	1.00	0.00	1.00
0.00	0.00	0.00	0.00	0.00	0.00	0.00
0.00	0.00	0.00	0.00	0.00	0.00	0.00
0.00	0.00	0.00	0.00	0.00	0.00	0.00
0.00	0.00	0.00	0.00	0.00	0.00	0.00
0.00	0.00	0.00	0.00	0.00	0.00	0.00
0.00	0.00	0.00	0.00	0.00	0.00	0.00
0.00	0.00	0.00	0.00	0.00	0.00	0.00
0.00	0.00	0.00	0.00	0.00	0.00	0.00
0.00	0.00	0.00	0.00	0.00	0.00	0.00
0.00	0.00	0.00	0.00	0.00	0.00	0.00
1.00	0.00	1.00	0.00	1.00	0.00	1.00
1.00	1.00	1.00	1.00	1.00	0.00	1.00
0.00	0.00	0.00	0.00	0.00	0.00	0.00
0.00	0.00	0.00	0.00	0.00	0.00	0.00
0.00	0.00	0.00	0.00	0.00	0.00	0.00
0.00	0.00	0.00	0.00	0.00	0.00	0.00
0.00	0.00	0.00	0.00	0.00	0.00	0.00
1.00	0.00	1.00	0.00	1.00	0.00	1.00

0.00	0.00	0.00	0.00	0.00	0.00	0.00
0.00	0.00	0.00	0.00	0.00	0.00	0.00
0.00	0.00	0.00	0.00	0.00	0.00	0.00
1.00	1.00	1.00	1.00	1.00	0.00	1.00
0.00	0.00	0.00	0.00	0.00	0.00	0.00
0.00	0.00	0.00	0.00	0.00	0.00	0.00
0.00	0.00	0.00	0.00	0.00	0.00	0.00
0.00	0.00	0.00	0.00	0.00	0.00	0.00
1.00	0.00	1.00	0.00	1.00	0.00	1.00
1.00	1.00	1.00	1.00	1.00	0.00	1.00
0.00	0.00	0.00	0.00	0.00	0.00	0.00
0.00	0.00	0.00	0.00	0.00	0.00	0.00
0.00	0.00	0.00	0.00	0.00	0.00	0.00
0.00	0.00	0.00	0.00	0.00	0.00	0.00
1.00	1.00	1.00	1.00	1.00	0.00	1.00
0.00	1.00	0.00	0.00	0.00	0.00	0.00
0.00	0.00	0.00	0.00	0.00	0.00	0.00
0.00	0.00	0.00	0.00	0.00	0.00	0.00
0.00	0.00	0.00	0.00	0.00	0.00	0.00
0.00	0.00	0.00	0.00	0.00	0.00	0.00
0.00	0.00	0.00	0.00	0.00	0.00	0.00
0.00	0.00	0.00	0.00	0.00	0.00	0.00
1.00	0.00	1.00	0.00	1.00	0.00	1.00
1.00	0.00	1.00	0.00	1.00	0.00	1.00
1.00	1.00	1.00	1.00	1.00	0.00	1.00
0.00	0.00	0.00	0.00	0.00	0.00	0.00
0.00	1.00	0.00	1.00	0.00	0.00	0.00
0.00	0.00	0.00	0.00	0.00	0.00	0.00
1.00	1.00	1.00	1.00	1.00	0.00	1.00
0.00	0.00	0.00	0.00	0.00	0.00	0.00
0.00	0.00	0.00	0.00	0.00	0.00	0.00
1.00	1.00	1.00	1.00	1.00	0.00	1.00
0.00	0.00	0.00	0.00	0.00	0.00	0.00
0.00	0.00	0.00	0.00	0.00	0.00	0.00
0.00	0.00	0.00	0.00	0.00	0.00	0.00
0.00	0.00	0.00	0.00	0.00	0.00	0.00
0.00	0.00	0.00	0.00	0.00	0.00	0.00
0.00	0.00	0.00	0.00	0.00	0.00	0.00
1.00	1.00	1.00	1.00	1.00	0.00	1.00
0.00	0.00	0.00	0.00	0.00	0.00	0.00
0.00	0.00	0.00	0.00	0.00	0.00	0.00
0.00	0.00	0.00	0.00	0.00	0.00	0.00
1.00	1.00	1.00	1.00	1.00	0.00	1.00
0.00	0.00	0.00	0.00	0.00	0.00	0.00
0.00	1.00	0.00	1.00	0.00	0.00	0.00
1.00	1.00	1.00	1.00	1.00	0.00	1.00
1.00	0.00	1.00	0.00	1.00	0.00	1.00
1.00	0.00	0.00	0.00	0.00	0.00	0.00
0.00	0.00	0.00	0.00	0.00	0.00	0.00
0.00	0.00	0.00	0.00	0.00	0.00	0.00
0.00	0.00	0.00	0.00	0.00	0.00	0.00
0.00	0.00	0.00	0.00	0.00	0.00	0.00
0.00	0.00	0.00	0.00	0.00	0.00	0.00
1.00	0.00	0.00	0.00	0.00	0.00	0.00
0.00	0.00	0.00	0.00	0.00	0.00	0.00
1.00	0.00	1.00	0.00	1.00	0.00	1.00

0.00	0.00	0.00	0.00	0.00	0.00	0.00
0.00	0.00	0.00	0.00	0.00	0.00	0.00
1.00	0.00	1.00	0.00	1.00	0.00	1.00
1.00	1.00	1.00	1.00	1.00	0.00	1.00
0.00	0.00	0.00	0.00	0.00	0.00	0.00
0.00	0.00	0.00	0.00	0.00	0.00	0.00
0.00	0.00	0.00	0.00	0.00	0.00	0.00
0.00	0.00	0.00	0.00	0.00	0.00	0.00
0.00	0.00	0.00	0.00	0.00	0.00	0.00
1.00	1.00	1.00	1.00	1.00	0.00	1.00
0.00	0.00	0.00	0.00	0.00	0.00	0.00
0.00	1.00	0.00	1.00	0.00	0.00	0.00
0.00	0.00	0.00	0.00	0.00	0.00	0.00
0.00	0.00	0.00	0.00	0.00	0.00	0.00
0.00	0.00	0.00	0.00	0.00	0.00	0.00
1.00	1.00	1.00	1.00	1.00	0.00	1.00
0.00	0.00	0.00	0.00	0.00	0.00	0.00
0.00	0.00	0.00	0.00	0.00	0.00	0.00
0.00	0.00	0.00	0.00	0.00	0.00	0.00
0.00	0.00	0.00	0.00	0.00	0.00	0.00
0.00	0.00	0.00	0.00	0.00	0.00	0.00
1.00	0.00	1.00	0.00	1.00	0.00	1.00
1.00	1.00	1.00	1.00	1.00	0.00	1.00
0.00	0.00	0.00	0.00	0.00	0.00	0.00
0.00	1.00	0.00	1.00	0.00	0.00	0.00
1.00	1.00	1.00	1.00	1.00	0.00	1.00
0.00	0.00	0.00	0.00	0.00	0.00	0.00
0.00	1.00	0.00	1.00	0.00	0.00	0.00
0.00	0.00	0.00	0.00	0.00	0.00	0.00
1.00	1.00	1.00	1.00	1.00	0.00	1.00
0.00	0.00	0.00	0.00	0.00	0.00	0.00
0.00	0.00	0.00	0.00	0.00	0.00	0.00
0.00	0.00	0.00	0.00	0.00	0.00	0.00
1.00	0.00	1.00	0.00	1.00	0.00	1.00
1.00	0.00	1.00	0.00	1.00	0.00	1.00
0.00	0.00	0.00	0.00	0.00	0.00	0.00
0.00	0.00	0.00	0.00	0.00	0.00	0.00
1.00	1.00	0.00	1.00	0.00	0.00	0.00
1.00	1.00	1.00	1.00	1.00	0.00	1.00
0.00	0.00	0.00	0.00	0.00	0.00	0.00
0.00	0.00	0.00	0.00	0.00	0.00	0.00
1.00	1.00	1.00	1.00	1.00	0.00	1.00
1.00	1.00	1.00	1.00	1.00	0.00	1.00
0.00	1.00	0.00	1.00	0.00	0.00	0.00
0.00	0.00	0.00	0.00	0.00	0.00	0.00
0.00	1.00	0.00	1.00	0.00	0.00	0.00
1.00	0.00	1.00	0.00	1.00	0.00	1.00
0.00	0.00	0.00	0.00	0.00	0.00	0.00
0.00	0.00	0.00	0.00	0.00	0.00	0.00
0.00	0.00	0.00	0.00	0.00	0.00	0.00
0.00	0.00	0.00	0.00	0.00	0.00	0.00
0.00	1.00	0.00	1.00	0.00	0.00	0.00
0.00	0.00	0.00	0.00	0.00	0.00	0.00
0.00	0.00	0.00	0.00	0.00	0.00	0.00
0.00	1.00	0.00	1.00	0.00	0.00	0.00
0.00	0.00	0.00	0.00	0.00	0.00	0.00

0.00	1.00	0.00	1.00	0.00	0.00	0.00
0.00	0.00	0.00	0.00	0.00	0.00	0.00
0.00	0.00	0.00	0.00	0.00	0.00	0.00
1.00	1.00	1.00	1.00	1.00	0.00	1.00
1.00	0.00	1.00	0.00	1.00	0.00	1.00
0.00	0.00	0.00	0.00	0.00	0.00	0.00
0.00	0.00	0.00	0.00	0.00	0.00	0.00
0.00	0.00	0.00	0.00	0.00	0.00	0.00
1.00	1.00	1.00	0.00	1.00	0.00	1.00
0.00	0.00	0.00	0.00	0.00	0.00	0.00
1.00	0.00	1.00	0.00	1.00	0.00	1.00
1.00	0.00	1.00	0.00	1.00	0.00	1.00
0.00	1.00	0.00	1.00	0.00	0.00	0.00
0.00	0.00	0.00	0.00	0.00	0.00	0.00
1.00	1.00	1.00	1.00	1.00	0.00	1.00
1.00	1.00	1.00	1.00	1.00	0.00	1.00
1.00	1.00	1.00	1.00	1.00	0.00	1.00
1.00	1.00	1.00	1.00	0.00	0.00	0.00
0.00	0.00	0.00	0.00	0.00	0.00	0.00
0.00	0.00	0.00	0.00	0.00	0.00	0.00
0.00	0.00	0.00	0.00	0.00	0.00	0.00
0.00	0.00	0.00	0.00	0.00	0.00	0.00
0.00	0.00	0.00	0.00	0.00	0.00	0.00
0.00	0.00	0.00	0.00	0.00	0.00	0.00
0.00	0.00	0.00	0.00	0.00	0.00	0.00
0.00	0.00	0.00	0.00	0.00	0.00	0.00
0.00	0.00	0.00	0.00	0.00	0.00	0.00
0.00	0.00	0.00	0.00	0.00	0.00	0.00
0.00	1.00	0.00	1.00	0.00	0.00	0.00
0.00	0.00	0.00	0.00	0.00	0.00	0.00
0.00	0.00	0.00	0.00	0.00	0.00	0.00
1.00	1.00	1.00	1.00	1.00	0.00	1.00
1.00	1.00	1.00	1.00	1.00	0.00	1.00
0.00	0.00	0.00	0.00	0.00	0.00	0.00
1.00	1.00	1.00	1.00	1.00	0.00	1.00
0.00	0.00	0.00	0.00	0.00	0.00	0.00
0.00	0.00	0.00	0.00	0.00	0.00	0.00
1.00	1.00	1.00	1.00	1.00	0.00	1.00
1.00	1.00	1.00	1.00	1.00	0.00	1.00
0.00	0.00	0.00	0.00	0.00	0.00	0.00
0.00	0.00	0.00	0.00	0.00	0.00	0.00
0.00	0.00	0.00	0.00	0.00	0.00	0.00
1.00	1.00	1.00	1.00	1.00	0.00	1.00
0.00	0.00	0.00	0.00	0.00	0.00	0.00
0.00	0.00	0.00	0.00	0.00	0.00	0.00
1.00	1.00	1.00	1.00	1.00	0.00	1.00
0.00	0.00	0.00	0.00	0.00	0.00	0.00
0.00	1.00	0.00	1.00	0.00	0.00	0.00
0.00	0.00	0.00	0.00	0.00	0.00	0.00
1.00	1.00	1.00	1.00	1.00	0.00	1.00
0.00	0.00	0.00	0.00	0.00	0.00	0.00
0.00	0.00	0.00	0.00	0.00	0.00	0.00
0.00	0.00	0.00	0.00	0.00	0.00	0.00
0.00	1.00	0.00	1.00	0.00	0.00	0.00
0.00	0.00	0.00	0.00	0.00	0.00	0.00
0.00	0.00	0.00	0.00	0.00	0.00	0.00

1.00	1.00	1.00	1.00	1.00	0.00	1.00
0.00	0.00	0.00	0.00	0.00	0.00	0.00
1.00	0.00	1.00	0.00	1.00	0.00	0.00
0.00	0.00	0.00	0.00	0.00	0.00	0.00
1.00	1.00	1.00	1.00	1.00	0.00	1.00
1.00	1.00	1.00	1.00	1.00	0.00	1.00
0.00	0.00	0.00	0.00	0.00	0.00	0.00
0.00	0.00	0.00	0.00	0.00	0.00	0.00
0.00	0.00	0.00	0.00	0.00	0.00	0.00
0.00	0.00	0.00	0.00	0.00	0.00	0.00
0.00	0.00	0.00	0.00	0.00	0.00	0.00
0.00	0.00	0.00	0.00	0.00	0.00	0.00
0.00	0.00	0.00	0.00	0.00	0.00	0.00
1.00	1.00	1.00	1.00	1.00	0.00	1.00
1.00	1.00	1.00	1.00	1.00	0.00	1.00
1.00	0.00	1.00	0.00	1.00	0.00	1.00
1.00	1.00	1.00	1.00	1.00	0.00	0.00
0.00	0.00	0.00	0.00	0.00	0.00	0.00
0.00	0.00	0.00	0.00	0.00	0.00	0.00
0.00	0.00	0.00	0.00	0.00	0.00	0.00
0.00	0.00	0.00	0.00	0.00	0.00	0.00
0.00	0.00	0.00	0.00	0.00	0.00	0.00
0.00	0.00	0.00	0.00	0.00	0.00	0.00
0.00	0.00	0.00	0.00	0.00	0.00	0.00
0.00	0.00	0.00	0.00	0.00	0.00	0.00
1.00	1.00	1.00	1.00	1.00	0.00	1.00
0.00	0.00	0.00	0.00	0.00	0.00	0.00
0.00	1.00	0.00	1.00	0.00	0.00	0.00
1.00	0.00	1.00	0.00	1.00	0.00	1.00
0.00	0.00	0.00	0.00	0.00	0.00	0.00
1.00	1.00	1.00	1.00	1.00	0.00	1.00
0.00	0.00	0.00	0.00	0.00	0.00	0.00
1.00	0.00	1.00	0.00	1.00	0.00	1.00
0.00	0.00	0.00	0.00	0.00	0.00	0.00
0.00	0.00	0.00	0.00	0.00	0.00	0.00
0.00	0.00	0.00	0.00	0.00	0.00	0.00
0.00	0.00	0.00	0.00	0.00	0.00	0.00
0.00	0.00	0.00	0.00	0.00	0.00	0.00
0.00	0.00	0.00	0.00	0.00	0.00	0.00
0.00	0.00	0.00	0.00	0.00	0.00	0.00
0.00	0.00	0.00	0.00	0.00	0.00	0.00
1.00	1.00	1.00	1.00	1.00	0.00	1.00
0.00	1.00	0.00	1.00	0.00	0.00	0.00
0.00	0.00	0.00	0.00	0.00	0.00	0.00
0.00	1.00	0.00	1.00	0.00	0.00	0.00
0.00	0.00	0.00	0.00	0.00	0.00	0.00
0.00	0.00	0.00	0.00	0.00	0.00	0.00
0.00	0.00	0.00	0.00	0.00	0.00	0.00
1.00	0.00	1.00	0.00	1.00	0.00	0.00
0.00	0.00	0.00	0.00	0.00	0.00	0.00
0.00	0.00	0.00	0.00	0.00	0.00	0.00
1.00	0.00	1.00	0.00	1.00	0.00	1.00
0.00	0.00	0.00	0.00	0.00	0.00	0.00
0.00	1.00	0.00	1.00	0.00	0.00	0.00
1.00	1.00	1.00	1.00	1.00	0.00	1.00
0.00	0.00	0.00	0.00	0.00	0.00	0.00

0.00	0.00	0.00	0.00	0.00	0.00	0.00
0.00	0.00	0.00	0.00	0.00	0.00	0.00
0.00	0.00	0.00	0.00	0.00	0.00	0.00
0.00	0.00	0.00	0.00	0.00	0.00	0.00
1.00	1.00	1.00	1.00	0.00	0.00	0.00
0.00	0.00	0.00	0.00	0.00	0.00	0.00
0.00	0.00	0.00	0.00	0.00	0.00	0.00
1.00	0.00	0.00	0.00	0.00	0.00	0.00
0.00	1.00	0.00	1.00	0.00	0.00	0.00
1.00	0.00	1.00	0.00	1.00	0.00	1.00
1.00	0.00	1.00	0.00	1.00	0.00	1.00
0.00	0.00	0.00	0.00	0.00	0.00	0.00
0.00	0.00	0.00	0.00	0.00	0.00	0.00
0.00	0.00	0.00	0.00	0.00	0.00	0.00
0.00	0.00	0.00	0.00	0.00	0.00	0.00
0.00	0.00	0.00	0.00	0.00	0.00	0.00
1.00	0.00	1.00	0.00	1.00	0.00	1.00
0.00	0.00	0.00	0.00	0.00	0.00	0.00

d21406_oness	d143_oness	d2143_oness	d144_oness	d2144_oness
1.00	1.00	1.00	1.00	1.00
#NULL!	#NULL!	0.00	0.00	0.00
#NULL!	1.00	0.00	1.00	0.00
#NULL!	#NULL!	0.00	0.00	0.00
#NULL!	#NULL!	0.00	0.00	0.00
#NULL!	#NULL!	0.00	0.00	0.00
#NULL!	#NULL!	0.00	0.00	0.00
#NULL!	#NULL!	0.00	0.00	0.00
#NULL!	#NULL!	0.00	0.00	0.00
#NULL!	#NULL!	0.00	0.00	0.00
#NULL!	#NULL!	0.00	0.00	0.00
#NULL!	#NULL!	0.00	0.00	0.00
#NULL!	#NULL!	0.00	0.00	0.00
#NULL!	#NULL!	0.00	0.00	0.00
1.00	#NULL!	1.00	0.00	1.00
#NULL!	#NULL!	0.00	0.00	0.00
#NULL!	#NULL!	0.00	0.00	0.00
#NULL!	#NULL!	0.00	0.00	0.00
1.00	#NULL!	1.00	0.00	1.00
1.00	1.00	1.00	1.00	1.00
#NULL!	#NULL!	0.00	0.00	0.00
#NULL!	#NULL!	0.00	0.00	0.00
#NULL!	#NULL!	0.00	0.00	0.00
#NULL!	#NULL!	0.00	0.00	0.00
#NULL!	1.00	0.00	0.00	0.00
#NULL!	#NULL!	0.00	0.00	0.00
#NULL!	#NULL!	0.00	0.00	0.00
#NULL!	#NULL!	0.00	0.00	0.00
#NULL!	#NULL!	0.00	0.00	0.00
#NULL!	#NULL!	0.00	0.00	0.00
#NULL!	#NULL!	0.00	0.00	0.00
#NULL!	#NULL!	0.00	0.00	0.00
1.00	1.00	1.00	1.00	1.00
#NULL!	#NULL!	0.00	0.00	0.00
#NULL!	#NULL!	0.00	0.00	0.00
#NULL!	#NULL!	0.00	0.00	0.00
1.00	#NULL!	1.00	0.00	1.00
#NULL!	#NULL!	0.00	0.00	0.00
#NULL!	#NULL!	0.00	0.00	0.00
#NULL!	#NULL!	0.00	0.00	0.00
#NULL!	#NULL!	0.00	0.00	0.00
#NULL!	#NULL!	0.00	0.00	0.00
#NULL!	#NULL!	0.00	0.00	0.00
#NULL!	#NULL!	0.00	0.00	0.00
#NULL!	#NULL!	0.00	0.00	0.00
#NULL!	#NULL!	0.00	0.00	0.00
#NULL!	#NULL!	0.00	0.00	0.00
1.00	#NULL!	1.00	0.00	1.00
1.00	1.00	1.00	0.00	1.00
#NULL!	#NULL!	0.00	0.00	0.00
#NULL!	#NULL!	0.00	0.00	0.00
#NULL!	#NULL!	0.00	0.00	0.00
#NULL!	#NULL!	0.00	0.00	0.00
#NULL!	#NULL!	0.00	0.00	0.00
1.00	#NULL!	1.00	0.00	1.00

#NULL!	#NULL!	0.00	0.00	0.00
#NULL!	#NULL!	0.00	0.00	0.00
#NULL!	#NULL!	0.00	0.00	0.00
1.00	1.00	0.00	1.00	0.00
#NULL!	#NULL!	0.00	0.00	0.00
#NULL!	#NULL!	0.00	0.00	0.00
#NULL!	#NULL!	0.00	0.00	0.00
#NULL!	#NULL!	0.00	0.00	0.00
1.00	#NULL!	1.00	0.00	1.00
1.00	1.00	1.00	0.00	1.00
#NULL!	#NULL!	0.00	0.00	0.00
#NULL!	#NULL!	0.00	0.00	0.00
#NULL!	#NULL!	0.00	0.00	0.00
#NULL!	#NULL!	0.00	0.00	0.00
1.00	1.00	1.00	1.00	1.00
#NULL!	#NULL!	0.00	0.00	0.00
#NULL!	#NULL!	0.00	0.00	0.00
#NULL!	#NULL!	0.00	0.00	0.00
#NULL!	#NULL!	0.00	0.00	0.00
#NULL!	#NULL!	0.00	0.00	0.00
#NULL!	#NULL!	0.00	0.00	0.00
#NULL!	#NULL!	0.00	0.00	0.00
1.00	#NULL!	1.00	0.00	1.00
1.00	#NULL!	1.00	0.00	1.00
1.00	1.00	1.00	1.00	1.00
#NULL!	#NULL!	0.00	0.00	0.00
#NULL!	1.00	0.00	0.00	0.00
#NULL!	#NULL!	0.00	0.00	0.00
1.00	1.00	1.00	1.00	1.00
#NULL!	#NULL!	0.00	0.00	0.00
#NULL!	#NULL!	0.00	0.00	0.00
1.00	1.00	1.00	1.00	1.00
#NULL!	#NULL!	0.00	0.00	0.00
#NULL!	#NULL!	0.00	0.00	0.00
#NULL!	#NULL!	0.00	0.00	0.00
#NULL!	#NULL!	0.00	0.00	0.00
#NULL!	#NULL!	0.00	0.00	0.00
#NULL!	#NULL!	0.00	0.00	0.00
1.00	1.00	1.00	1.00	1.00
#NULL!	#NULL!	0.00	0.00	0.00
#NULL!	#NULL!	0.00	0.00	0.00
#NULL!	#NULL!	0.00	0.00	0.00
1.00	1.00	1.00	1.00	1.00
#NULL!	#NULL!	0.00	0.00	0.00
#NULL!	1.00	0.00	1.00	0.00
1.00	1.00	1.00	1.00	1.00
1.00	#NULL!	1.00	0.00	1.00
#NULL!	#NULL!	0.00	0.00	0.00
#NULL!	#NULL!	0.00	0.00	0.00
#NULL!	#NULL!	0.00	0.00	0.00
#NULL!	#NULL!	0.00	0.00	0.00
#NULL!	#NULL!	0.00	0.00	0.00
#NULL!	#NULL!	0.00	0.00	0.00
#NULL!	#NULL!	0.00	0.00	0.00
#NULL!	#NULL!	0.00	0.00	0.00
1.00	#NULL!	1.00	0.00	1.00

#NULL!	#NULL!	0.00	0.00	0.00
#NULL!	#NULL!	0.00	0.00	0.00
1.00	#NULL!	1.00	0.00	1.00
1.00	1.00	1.00	1.00	1.00
#NULL!	#NULL!	0.00	0.00	0.00
#NULL!	#NULL!	0.00	0.00	0.00
#NULL!	#NULL!	0.00	0.00	0.00
#NULL!	#NULL!	0.00	0.00	0.00
#NULL!	#NULL!	0.00	0.00	0.00
1.00	1.00	1.00	1.00	1.00
#NULL!	#NULL!	0.00	0.00	0.00
#NULL!	1.00	0.00	1.00	0.00
#NULL!	#NULL!	0.00	0.00	0.00
#NULL!	#NULL!	0.00	0.00	0.00
#NULL!	#NULL!	0.00	0.00	0.00
1.00	1.00	1.00	1.00	1.00
#NULL!	#NULL!	0.00	0.00	0.00
#NULL!	#NULL!	0.00	0.00	0.00
#NULL!	#NULL!	0.00	0.00	0.00
#NULL!	#NULL!	0.00	0.00	0.00
#NULL!	#NULL!	0.00	0.00	0.00
1.00	#NULL!	0.00	0.00	0.00
1.00	1.00	1.00	1.00	1.00
#NULL!	#NULL!	0.00	0.00	0.00
#NULL!	1.00	0.00	1.00	0.00
1.00	1.00	1.00	1.00	1.00
#NULL!	#NULL!	0.00	0.00	0.00
#NULL!	1.00	0.00	1.00	0.00
#NULL!	#NULL!	0.00	0.00	0.00
1.00	1.00	1.00	1.00	1.00
#NULL!	#NULL!	0.00	0.00	0.00
#NULL!	#NULL!	0.00	0.00	0.00
#NULL!	#NULL!	0.00	0.00	0.00
1.00	#NULL!	1.00	0.00	1.00
1.00	#NULL!	1.00	0.00	1.00
#NULL!	#NULL!	0.00	0.00	0.00
#NULL!	#NULL!	0.00	0.00	0.00
#NULL!	1.00	0.00	1.00	0.00
1.00	1.00	1.00	1.00	1.00
#NULL!	#NULL!	0.00	0.00	0.00
#NULL!	#NULL!	0.00	0.00	0.00
1.00	1.00	1.00	1.00	1.00
1.00	1.00	1.00	1.00	1.00
#NULL!	1.00	0.00	1.00	0.00
#NULL!	#NULL!	0.00	0.00	0.00
#NULL!	1.00	0.00	1.00	0.00
1.00	#NULL!	1.00	0.00	1.00
#NULL!	#NULL!	0.00	0.00	0.00
#NULL!	#NULL!	0.00	0.00	0.00
#NULL!	#NULL!	0.00	0.00	0.00
#NULL!	#NULL!	0.00	0.00	0.00
#NULL!	1.00	0.00	0.00	0.00
#NULL!	#NULL!	0.00	0.00	0.00
#NULL!	#NULL!	0.00	0.00	0.00
#NULL!	1.00	0.00	1.00	0.00
#NULL!	#NULL!	0.00	0.00	0.00

#NULL!	1.00	0.00	0.00	0.00
#NULL!	#NULL!	0.00	0.00	0.00
#NULL!	#NULL!	0.00	0.00	0.00
1.00	1.00	1.00	1.00	1.00
1.00	#NULL!	1.00	0.00	1.00
#NULL!	#NULL!	0.00	0.00	0.00
#NULL!	#NULL!	0.00	0.00	0.00
#NULL!	#NULL!	0.00	0.00	0.00
1.00	#NULL!	1.00	0.00	1.00
#NULL!	#NULL!	0.00	0.00	0.00
1.00	#NULL!	0.00	0.00	0.00
1.00	#NULL!	1.00	0.00	1.00
#NULL!	1.00	0.00	1.00	0.00
#NULL!	#NULL!	0.00	0.00	0.00
1.00	1.00	1.00	1.00	1.00
1.00	1.00	1.00	1.00	1.00
1.00	1.00	1.00	1.00	1.00
#NULL!	1.00	0.00	1.00	0.00
#NULL!	#NULL!	0.00	0.00	0.00
#NULL!	#NULL!	0.00	0.00	0.00
#NULL!	#NULL!	0.00	0.00	0.00
#NULL!	#NULL!	0.00	0.00	0.00
#NULL!	#NULL!	0.00	0.00	0.00
#NULL!	#NULL!	0.00	0.00	0.00
#NULL!	#NULL!	0.00	0.00	0.00
#NULL!	#NULL!	0.00	0.00	0.00
#NULL!	#NULL!	0.00	0.00	0.00
#NULL!	#NULL!	0.00	0.00	0.00
#NULL!	1.00	0.00	1.00	0.00
#NULL!	#NULL!	0.00	0.00	0.00
#NULL!	#NULL!	0.00	0.00	0.00
1.00	1.00	1.00	1.00	1.00
1.00	1.00	1.00	1.00	1.00
#NULL!	#NULL!	0.00	0.00	0.00
1.00	1.00	1.00	1.00	1.00
#NULL!	#NULL!	0.00	0.00	0.00
#NULL!	#NULL!	0.00	0.00	0.00
1.00	1.00	1.00	1.00	1.00
1.00	1.00	1.00	1.00	1.00
#NULL!	#NULL!	0.00	0.00	0.00
#NULL!	#NULL!	0.00	0.00	0.00
#NULL!	#NULL!	0.00	0.00	0.00
1.00	1.00	1.00	1.00	1.00
#NULL!	#NULL!	0.00	0.00	0.00
#NULL!	#NULL!	0.00	0.00	0.00
1.00	#NULL!	1.00	0.00	1.00
#NULL!	#NULL!	0.00	0.00	0.00
#NULL!	1.00	0.00	1.00	0.00
#NULL!	#NULL!	0.00	0.00	0.00
1.00	1.00	1.00	1.00	1.00
#NULL!	#NULL!	0.00	0.00	0.00
#NULL!	#NULL!	0.00	0.00	0.00
#NULL!	#NULL!	0.00	0.00	0.00
#NULL!	#NULL!	0.00	0.00	0.00
#NULL!	#NULL!	0.00	0.00	0.00
#NULL!	#NULL!	0.00	0.00	0.00

1.00	1.00	1.00	1.00	1.00
#NULL!	#NULL!	0.00	0.00	0.00
#NULL!	#NULL!	0.00	0.00	0.00
#NULL!	#NULL!	0.00	0.00	0.00
1.00	1.00	1.00	1.00	1.00
1.00	1.00	1.00	1.00	1.00
#NULL!	#NULL!	0.00	0.00	0.00
#NULL!	#NULL!	0.00	0.00	0.00
#NULL!	#NULL!	0.00	0.00	0.00
#NULL!	#NULL!	0.00	0.00	0.00
#NULL!	#NULL!	0.00	0.00	0.00
#NULL!	#NULL!	0.00	0.00	0.00
#NULL!	#NULL!	0.00	0.00	0.00
1.00	1.00	1.00	1.00	1.00
1.00	1.00	1.00	0.00	1.00
1.00	#NULL!	1.00	0.00	1.00
#NULL!	1.00	0.00	1.00	0.00
#NULL!	#NULL!	0.00	0.00	0.00
#NULL!	#NULL!	0.00	0.00	0.00
#NULL!	#NULL!	0.00	0.00	0.00
#NULL!	#NULL!	0.00	0.00	0.00
#NULL!	#NULL!	0.00	0.00	0.00
#NULL!	#NULL!	0.00	0.00	0.00
#NULL!	#NULL!	0.00	0.00	0.00
#NULL!	#NULL!	0.00	0.00	0.00
1.00	1.00	1.00	1.00	1.00
#NULL!	#NULL!	0.00	0.00	0.00
#NULL!	1.00	0.00	0.00	0.00
1.00	#NULL!	1.00	0.00	1.00
#NULL!	#NULL!	0.00	0.00	0.00
1.00	1.00	1.00	1.00	1.00
#NULL!	#NULL!	0.00	0.00	0.00
1.00	#NULL!	1.00	0.00	1.00
#NULL!	#NULL!	0.00	0.00	0.00
#NULL!	#NULL!	0.00	0.00	0.00
#NULL!	#NULL!	0.00	0.00	0.00
#NULL!	#NULL!	0.00	0.00	0.00
#NULL!	#NULL!	0.00	0.00	0.00
#NULL!	#NULL!	0.00	0.00	0.00
#NULL!	#NULL!	0.00	0.00	0.00
#NULL!	#NULL!	0.00	0.00	0.00
1.00	1.00	1.00	1.00	1.00
#NULL!	1.00	0.00	1.00	0.00
#NULL!	#NULL!	0.00	0.00	0.00
#NULL!	1.00	0.00	1.00	0.00
#NULL!	#NULL!	0.00	0.00	0.00
#NULL!	#NULL!	0.00	0.00	0.00
#NULL!	#NULL!	0.00	0.00	0.00
#NULL!	#NULL!	0.00	0.00	0.00
#NULL!	#NULL!	0.00	0.00	0.00
#NULL!	#NULL!	0.00	0.00	0.00
1.00	#NULL!	1.00	0.00	1.00
#NULL!	#NULL!	0.00	0.00	0.00
#NULL!	1.00	0.00	1.00	0.00
1.00	1.00	1.00	1.00	1.00
#NULL!	#NULL!	0.00	0.00	0.00

#NULL!	#NULL!	0.00	0.00	0.00
#NULL!	#NULL!	0.00	0.00	0.00
#NULL!	#NULL!	0.00	0.00	0.00
#NULL!	#NULL!	0.00	0.00	0.00
#NULL!	1.00	0.00	1.00	0.00
#NULL!	#NULL!	0.00	0.00	0.00
#NULL!	#NULL!	0.00	0.00	0.00
#NULL!	#NULL!	0.00	0.00	0.00
#NULL!	1.00	0.00	1.00	0.00
1.00	#NULL!	1.00	0.00	1.00
1.00	#NULL!	1.00	0.00	1.00
#NULL!	#NULL!	0.00	0.00	0.00
#NULL!	#NULL!	0.00	0.00	0.00
#NULL!	#NULL!	0.00	0.00	0.00
#NULL!	#NULL!	0.00	0.00	0.00
#NULL!	#NULL!	0.00	0.00	0.00
1.00	#NULL!	1.00	0.00	1.00
#NULL!	#NULL!	0.00	0.00	0.00
